# Diterpene Biosynthesis in *Catenulispora acidiphila*: On the Mechanism of Catenul‐14‐en‐6‐ol Synthase

**DOI:** 10.1002/anie.202014180

**Published:** 2020-12-10

**Authors:** Geng Li, Yue‐Wei Guo, Jeroen S. Dickschat

**Affiliations:** ^1^ Kekulé-Institute of Organic Chemistry and Biochemistry University of Bonn Gerhard-Domagk-Strasse 1 53121 Bonn Germany; ^2^ State Key Laboratory of Drug Research Shanghai Institute of Materia Medica Chinese Academy of Sciences 555 Zu Chong Zhi Road, Zhangjiang Hi-Tech Park 201203 Shanghai China; ^3^ University of Chinese Academy of Sciences No. 19A Yuquan Road Beijing 100049 China

**Keywords:** biosynthesis, enzyme mechanisms, isotopes, substrate analogues, terpenoids

## Abstract

A new diterpene synthase from the actinomycete Catenulispora acidiphila was identified and the structures of its products were elucidated, including the absolute configurations by an enantioselective deuteration approach. The mechanism of the cationic terpene cyclisation cascade was deeply studied through the use of isotopically labelled substrates and of substrate analogues with partially blocked reactivity, resulting in derailment products that gave further insights into the intermediates along the cascade. Their chemistry was studied, leading to the biomimetic synthesis of a diterpenoid analogue of a brominated sesquiterpene known from the red seaweed Laurencia microcladia.

## Graphical abstract

A new diterpene synthase (CaCS) from *Catenulispora acidiphila* and its products were identified. The enzyme mechanism was studied by isotopic labelling experiments and usage of substrate analogues with blocked reactivity, resulting in a series of derailment products. Their chemistry was studied, leading to the biomimetic synthesis of a diterpenoid analogue of a brominated sesquiterpene known from the red seaweed *Laurencia microcaldia*.

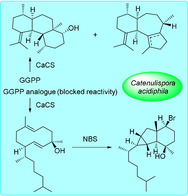

The fascinating skeletal diversity of terpenes is generated by terpene synthases (TSs) from a limited number of oligoprenyl diphosphates.[^1^, ^2^, ^3^] These intriguing biocatalysts perform some of the mechanistically most complex transformations in Nature, yet the basic principle of TS catalysis is remarkably simple at the same time. The initiation of terpene cyclisations always proceeds by substrate ionisation, either through diphosphate abstraction from or by protonation of the substrate,[^4^, ^5^] while the subsequent events are determined by the substrate conformation that is controlled by the shape of the enzyme's hydrophobic active site.^[6]^ After cation formation, a multistep cascade with cyclisations by intramolecular attack of double bonds to cationic centres or—rarely—ring openings or fragmentations by the reverse process,^[7]^ Wagner–Meerwein or dyotropic rearrangements,[^8^, ^9^, ^10^] hydride or proton shifts, attack of water or alcohol groups to a cation in the formation of terpene alcohols or ethers, and a terminal deprotonation is promoted. Intriguingly, all steps are performed with precise stereo‐, but very limited direct enzyme control, that is almost restricted to the stabilisation of cationic intermediates through cation–π interactions. The overall process results in the installation of usually polycyclic skeletons with multiple stereogenic centres. Mechanistic investigations of TSs can be performed by structure‐based site‐directed mutagenesis,^[11]^ usage of isotopically labelled substrates^[12]^ or substrate analogues,^[13]^ and DFT calculations,^[6]^ but the cationic intermediates along the cascade cannot be observed experimentally. During the past few years several di‐ and sesterterpene synthases (DTSs/StTSs) were identified from all kingdoms of life.[^2^, ^12^, ^14^, ^15^, ^16^, ^17^] As a result of the multiple reactive double bonds in their substrates geranylgeranyl (GGPP) and geranylfarnesyl diphosphate (GFPP) the cyclisation cascades for these enzymes are usually more complex and interesting to study than the cyclisations of geranyl (GPP) and farnesyl diphosphate (FPP) by mono‐ and sesquiterpene synthases (MTSs/STSs). We envisioned that substrate analogues with saturated double bonds would allow for interesting insights into diterpene cyclisation cascades, because they cannot undergo certain cyclisation steps as observed for GGPP and could lead to derailment products that may reflect early cyclisation events. Here we report on the identification of a new DTS from *Catenulispora acidiphila* and its mechanistic interrogation with isotopically labelled probes and with such GGPP analogues of limited reactivity.

The actinomycete *C. acidiphila* DSM 44928 caught our interest to study its TSs, because this strain was found to emit traces of a volatile diterpene hydrocarbon (Figure S1), suggesting that one of the TSs encoded in its genome should have DTS activity. From the two candidate enzymes returned by a BLAST search one appeared within a phylogenetic tree constructed from 3267 bacterial TS homologues close to several known STSs, while the second showed close phylogenetic relation to known DTSs and exhibited all highly conserved motifs expected for a functional enzyme (Figures S2 and S3), and was thus selected for further study. Gene cloning and expression followed by incubation of the purified protein (Figure S4) with GGPP resulted in the efficient formation of several diterpenes (Figures 1 and S5), while GPP, FPP, and GFPP were not converted. Three major products obtained from GGPP were isolated by silica gel chromatography followed by HPLC purification, and their structures were elucidated by one‐ and two‐dimensional NMR spectroscopy (Tables S2–S4, Figures S6–S26), resulting in the structures of the main product catenul‐14‐en‐6‐ol (**1**) with a 6‐6‐6 tricyclic system, and two hydrocarbons with the skeleton of a 6‐7‐5 tricyclic system, named isocatenula‐2,14‐diene (**2**) and isocatenula‐2(6),14‐diene (**3**). This identified the enzyme as *Catenulispora acidiphila* Catenul‐14‐en‐6‐ol Synthase (CaCS). A BLAST search and amino acid sequence alignment with the four closest hits suggests that enzymes with the same function may be present in a few other actinomycetes (Figure S27).

**Figure 1:**
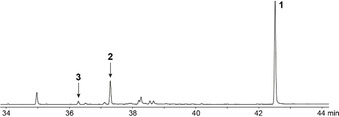
Total ion chromatogram of the products obtained from GGPP with CaCS.

The proposed cyclisation cascade for **1**–**3** (Scheme 1) starts from GGPP by 1,10‐cyclisation to **A**, followed by a 1,3‐hydride shift to **B**, 1,14‐cyclisation to **C** and deprotonation to the neutral intermediate **D**. Its reprotonation at C3 can induce another 2,7‐cyclisation with attack of water that may be concerted to avoid a secondary cation as intermediate, yielding **1**. Alternatively, the protonation at C3 of **D** can induce a 2,6‐cyclisation to **E** that upon a 1,2‐hydride migration to **F** and deprotonation gives access to **3**. Branching out from **F**, another 1,2‐hydride transfer to **G** and deprotonation result in **2**. This mechanism was investigated in a series of incubation experiments with isotopically labelled terpene precursors (Table S5). The conversion of all 20 isotopomers of (^13^C)GGPP, partly synthesised as reported previously and partly made accessible through enzymatic reactions by GGPP synthase (GGPPS) from *Streptomyces cyaneofuscatus* ^[15]^ from the corresponding isotopomers of GPP, FPP, or isopentenyl diphosphate (IPP), with CaCS resulted in the labelled compounds **1**–**3** carrying the ^13^C‐label in the expected positions from all labelled substrates (Figure S28, for an example cf. Figure 2). These experiments i) supported the GGPP fold leading to **1**–**3** and ii) revealed a strict stereochemical course for the geminal methyl groups C16 and C17 that did not show any scrambling of ^13^C‐labelling between these positions.

**Figure 2:**
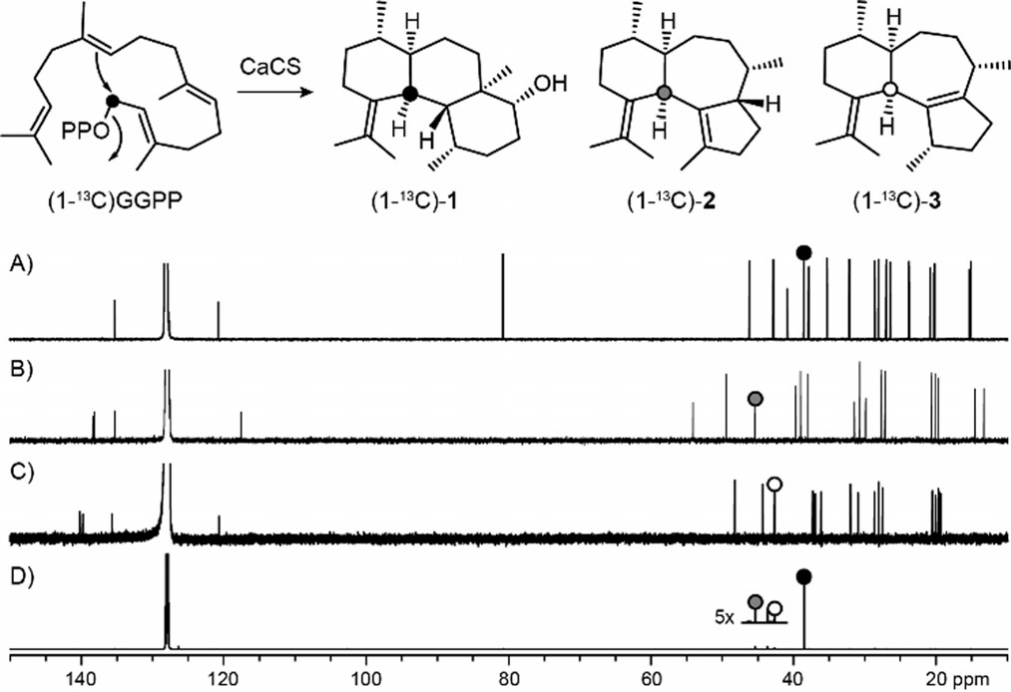
Labelling experiment with (1‐^13^C)GGPP and CaCS. ^13^C NMR spectra of A) **1**, B) **2**, C) **3**, and D) product mixture obtained from (1‐^13^C)GGPP with CaCS. Dots represent ^13^C‐labelled carbons.

**Scheme 1:**
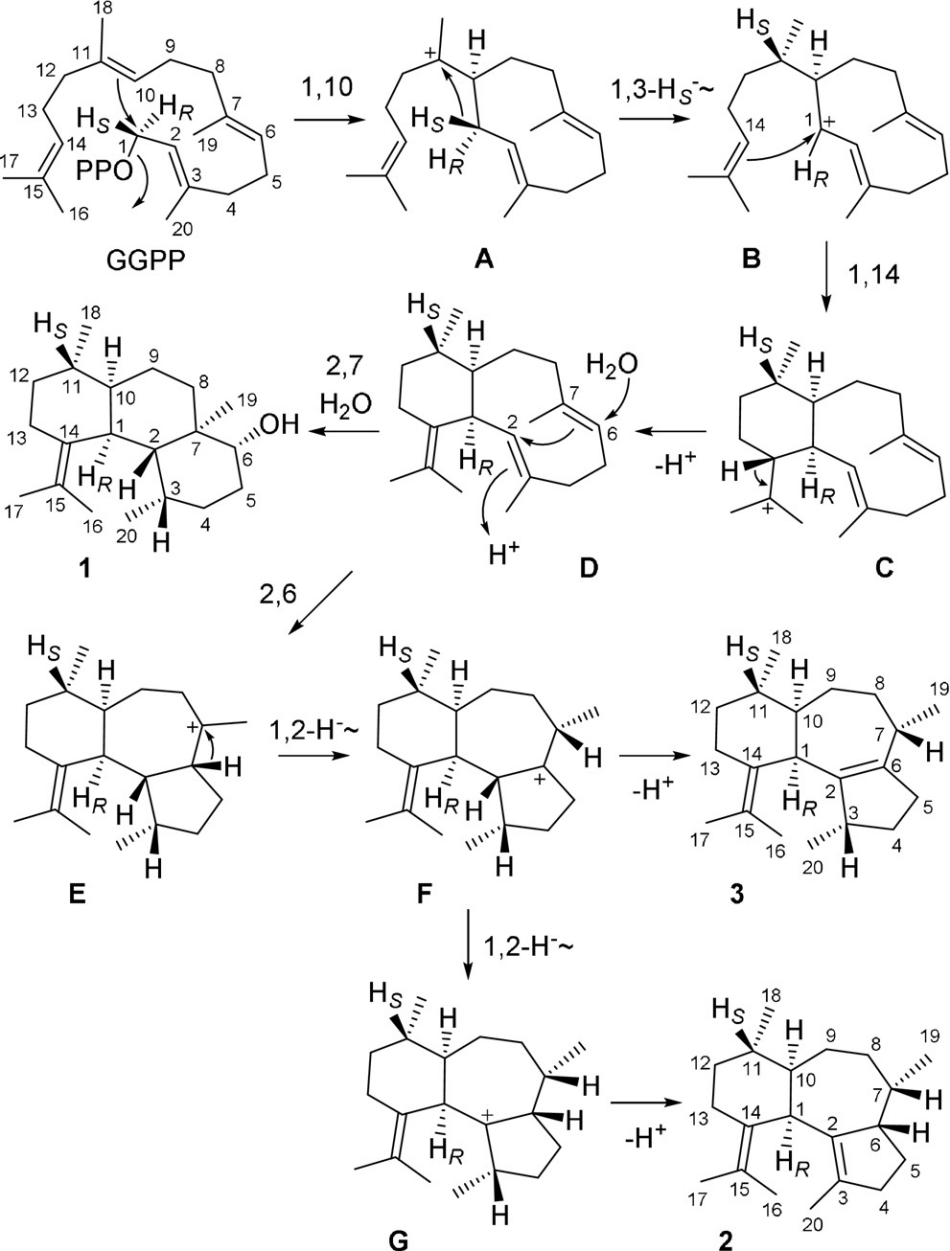
Cyclisation cascade from GGPP to the diterpenes **1**–**3** by CaCS. Carbon numberings of **1**–**3** were assigned so that the origin for each carbon from GGPP can be followed by the same number.

The 1,3‐hydride shift from C1 to C11 was followed by using (*R*)‐ and (*S*)‐(1‐^2^H)IPP,^[18]^ which were used to elongate (7‐^13^C)FPP with GGPPS to (*R*)‐ and (*S*)‐(1‐^2^H,11‐^13^C)GGPP, followed by cyclisation with CaCS (Figure S29). The hydride shift resulted in a direct ^13^C−^2^H connection in **1** when using (*S*)‐(1‐^2^H)IPP, as indicated by an upfield shifted triplet peak (−0.47 ppm, ^1^ *J*_C,D_=19.0 Hz) for C11 in the ^13^C NMR, while a singlet with a minor upfield shift (−0.01 ppm) was obtained with (*R*)‐(1‐^2^H)IPP, in agreement with a deuterium located two positions away. The alternative of two sequential 1,2‐hydride shifts from **A** to **B** was excluded by incubation of (3‐^13^C,2‐^2^H)GPP^[20]^ and IPP with GGPPS and CaCS, showing a singlet with an upfield shift (−0.08 ppm) in the ^13^C NMR as a result of deuterium bound at a carbon next to C11, indirectly giving further evidence for the 1,3‐hydride shift. We have previously shown that the analogous hydride shift occuring in the germacradienyl cation in the biosynthesis of many sesquiterpenes can be taken as an indicator for the absolute configurations of the products, as for 10*S* configuration the specific migration of the 1‐*pro*‐*S* hydrogen (H_*S*_) was observed, while for 10*R* configuration the 1‐*pro*‐*R* hydrogen (H_*R*_) was shifted.^[19]^ Along similar lines, the specific migration of H_*S*_ in **A** may be taken as an indicator for 10*S* configuration and thus for the absolute configurations of **1**–**3** as shown in Scheme 1 (for confirmation of absolute configurations see below).

The deprotonation at C14 from **C** to **D** was evident from an experiment with (2‐^2^H)DMAPP^[21]^ that was elongated with IPP and GGPPS to (14‐^2^H)GGPP, followed by cyclisation through CaCS. The mass spectra of **1**–**3** (Figure S30) demonstrated the deprotonation step at C14 from **C** to **D** by the observed molecular ions at *m*/*z=*290 and 272, respectively. The reprotonation of **D** at C3 with an external proton from the medium was demonstrated by incubation of FPP and (3‐^13^C)IPP^[15]^ with GGPPS and CaCS in D_2_O buffer, resulting in the typical upfield shifted triplet peak in the ^13^C NMR of **1** indicating a direct ^13^C−^2^H connection (Figure S31). Both experiments together also ruled out the hypothetical alternative of an intramolecular proton transfer from C14 to C3 in **C** that would bypass the neutral intermediate **D**. GC/MS analysis of the products obtained in the latter experiment simultaneously demonstrated the loss of the same deuterium in the deprotonation from **G** to **2** as taken up in the C3 reprotonation of **D** by a molecular ion at *m*/*z=*273 (Figure S32).

The 1,2‐hydride shift from **E** to **F** was demonstrated using the labeled substrate (2‐^2^H,3‐^13^C)FPP^[22]^ together with IPP, GGPPS and CaCS to yield (7‐^13^C,7‐^2^H)‐**2** for which a triplet peak was observed in the ^13^C NMR (Figure S33). Along similar lines, the 1,2‐hydride shift from **F** to **G** was evident from an upfield‐shifted triplet for C6 of **2** from substrates (2‐^13^C)FPP^[23]^ and (*S*)‐(2‐^2^H)IPP, generated in situ from (2‐^2^H)DMAPP with IDI from *Escherichia coli*,[^24^, ^25^] with GGPPS and CaCS (Figure S34). Furthermore, GC/MS analysis of the products from the same experiment demonstrated the loss of deuterium in the deprotonation from C2 in **F** to yield **3** by a molecular ion at *m*/*z=*273 (Figure S35). If (2‐^13^C)FPP was replaced by (3‐^13^C)FPP,^[23]^ an upfield‐shifted singlet (−0.08 ppm) for C7 occurred in the ^13^C NMR spectrum (Figure S36), which further supported the 1,2‐hydride shift and ruled out a direct 1,3‐hydride shift from **E** to **G** in the biosynthesis of **2**.

The absolute configurations of **1**–**3** were determined using the substrate DMAPP together with the stereoselectively deuterated probes (*E*)‐ and (*Z*)‐(4‐^13^C,4‐^2^H)IPP,^[26]^ which were enzymatically converted with the combination of FPP synthase (FPPS) from *S. coelicolor* ^[27]^ and GGPPS for higher efficiency into stereoselectively deuterated and ^13^C‐labelled GGPP (Figures S37, S39, and S41). This reaction proceeds through a known stereochemical course with attack at C4 of IPP from the *Si* face.^[28]^ Subsequent cyclisation by CaCS yielded labelled **1**–**3** in which the deuterated carbon atoms serve as configurationally well defined stereogenic centres. The substitution of one of the two diastereotopic hydrogens by deuterium can be monitored with high sensitivity through HSQC spectroscopy. As a result of the ^13^C‐labelling, the signal for the remaining hydrogen is strongly enhanced, while the signal of the hydrogen replaced by deuterium has vanished. Together with the assignment of the relative orientation of the diastereotopic hydrogens with respect to the naturally present stereogenic centres in **1**–**3** by NOESY the absolute configurations of all three compounds could be concluded. Similar experiments were performed with (*R*)‐ and (*S*)‐(1‐^13^C,1‐^2^H)IPP,^[25]^ which were converted with IDI, FPPS, GGPPS, and CaCS into labelled **1**–**3** (Figures S38, S40, and S42). Herein, the attack at C1 of prenyl diphosphates is known to proceed with inversion of configuration.^[28]^ Through these experiments every methylene group of **1**–**3** (C4, C5, C8, C9, C12, and C13) was stereoselectively deuterated, and all experiments simultaneously pointed to the same absolute configurations as shown in Scheme 1. Moreover, the absolute configuration as already suspected from the specific 1,3‐migration of the 1‐*pro*‐*S* hydrogen in **A**, discussed above as a potential indicator of a 10*S* configuration, was confirmed.

To further explore the cyclisation mechanism of CaCS, incubation experiments with substrate analogues with partially blocked reactivity were performed. For this purpose, 6,7‐dihydro‐GPP was synthesised (Scheme S1), elongated twice with IPP using GGPPS, and cyclised by CaCS. While the 1,10‐cyclisation to **A′** and the subsequent 1,3‐hydride shift to **B′**, the analogues of the natural intermediates **A** and **B**, are still possible, the next 1,14‐cyclisation cannot proceed as a consequence of the saturated C14−C15 bond (Scheme 2). Therefore, we assumed that this substrate could yield 1,10‐cyclised derailment products, thus giving insights into the early cyclisation events. Extraction of the products, drying of the organic layer with MgSO_4_, and chromatographic purification yielded two compounds that were characterised by NMR spectroscopy (Tables S6 and S7, Figures S43–S56) as biflora‐4,10(19)‐diene (**5**), the dihydro‐analogue of the natural diterpene biflora‐4,10(19),15‐triene (**5 a**) from *Cubitermes umbratus* soldiers,^[29]^ and biflora‐4,10‐diene (**6**). Their structures supported an initial 1,10‐cyclisation, but also showed an additional 1,6‐cyclisation not observed in the native enzyme products **1**–**3** obtained from GGPP. The cyclisation mechanisms for **5** and **6** were investigated by isotopic labelling experiments with 6,7‐dihydro‐GPP and all five isotopomers of (^13^C)IPP (Table S8), yielding doubly labelled isotopomers of **5** and **6** with GGPPS and CaCS. Similarly, the usage of 10,11‐dihydro‐FPP^[30]^ with all five (^13^C)IPPs, and of synthetic (1‐^13^C)‐ and (2‐^13^C)‐6,7‐dihydro‐GPP (Scheme S1) with IPP gave seven more singly labelled isotopomers of **5** and **6**. Their ^13^C NMR spectra (Figure S57) indicated the origin for each carbon in the C1–C10+ C19 and C20 portion. Moreover, the ^2^ *J*_C,C_ couplings observed in the double labelling experiments with (1‐^13^C)IPP (labelling at C1/C5) and with (2‐^13^C)IPP (labelling at C2/C6) gave further evidence for the 1,6‐ring closure in **5** and **6**.

**Scheme 2:**
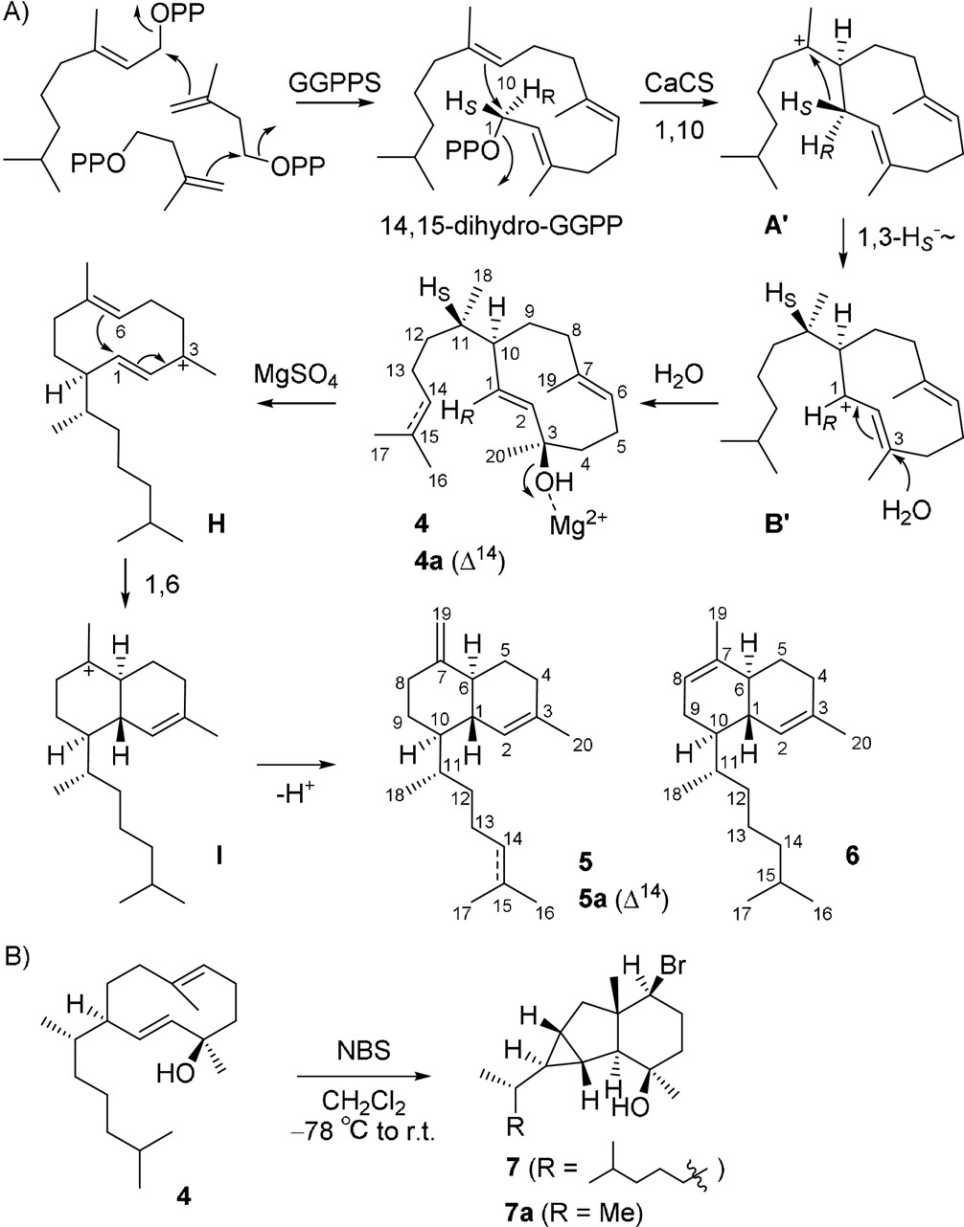
Interception of the diterpene cyclisation by CaCS with 14,15‐dihydro‐GGPP. A) Cyclisation mechanism to **4** and its MgSO_4_ dependent degradation, B) derivatisation of **4** with *N*‐bromosuccinimide (NBS). Carbon numberings deviate from that for **5 a** in the literature and indicate the origin of each carbon from 14,15‐dihydro‐GGPP by the same number.

The absolute configurations of **5** and **6** were determined by the enantioselective labelling strategy using 6,7‐dihydro‐GPP with (*R*)‐ and (*S*)‐(1‐^13^C,1‐^2^H)IPP and with (*E*)‐ and (*Z*)‐(4‐^13^C,4‐^2^H)IPP, or (*R*)‐ and (*S*)‐(1‐^13^C,1‐^2^H)‐6,7‐dihydro‐GPP (synthesised as in Scheme S2 in high enantiomeric purity, Figure S58) with IPP (Figures S59–S64). The experiments with (*R*)‐ and (*S*)‐(1‐^13^C,1‐^2^H)IPP also demonstrated the specific migration of the 1‐*pro*‐*S* hydrogen from **A′** to **B′**. The absolute configurations of **5** and **6** are analogous to that of natural **5 a**.^[31]^

Compound **5 a** is also known as a degradation product of obscuronatin (**4 a**), a known diterpene alcohol from the soft coral *Xenia obscuronata* ^[32]^ that shares the skeleton with the hypothetical intermediate 14,15‐dihydroobscuronatin (**4**). Therefore, we assumed **5** and **6** may likewise be degradation products of **4**, and indeed their formation depended on the usage of MgSO_4_ during workup (Figures S65 and S66), suggesting that the 1,6‐cyclisation in **5** and **6** is not under enzyme control. Their formation can be explained by water elimination through cations **H** and **I**. Workup without drying the organic extract with MgSO_4_ allowed the isolation of **4**, followed by structure elucidation through NMR spectroscopy (Table S9, Figures S67–S73), revealing the structure of an only 1,10‐cyclised enzyme product and thus further supporting the 1,10‐cyclisation as an early event of CaCS catalysis. Labelling of the carbons of the core structure strengthened the structural assignment, this time giving no ^2^ *J*_C,C_ couplings in the double labelling experiments with 6,7‐dihydro‐GPP and (1‐^13^C)IPP or (2‐^13^C)IPP in agreement with the macrocyclic ring, and allowed to follow the biosynthetic origin of the labelled carbons (Figure S74). However, the relative configuration of **4** with its distant stereogenic centres at C3 and C10/C11 could not independently be determined by NOESY. Therefore, the skeleton was rigidified in a reaction with NBS, resulting in the formation of **7** for which the full relative configuration could be determined by NMR‐based structure elucidation (Table S10, Figures S75–S81). Assuming that the stereogenic centres in **4** are not affected in the conversion to **7**, these results allowed to deduce the full relative configuration for **4**. Notably, this reaction may have some biosynthetic significance, as vanadium‐dependent haloperoxidases can catalyse similar transformations, which is especially important in the marine environment.[^33^, ^34^, ^35^] The brominated sesquiterpene **7 a** is structurally closely related to **7** and is a known natural product from the seaweed *Laurencia microcladia* ^[36]^ that potentially arises by such an enzymatic reaction. The absolute configuration of **4** was determined through enantioselective deuteration (Figures S82–S84).

We also envisioned that the protonation‐induced cyclisation of **D** to **E** (Scheme 1) could be blocked by use of 6,7‐dihydro‐GGPP, and its derailment products could give further evidence for the cyclisation mechanism by CaCS. Therefore, 6,7‐dihydro‐GGPP was also synthesised (Scheme S3) and enzymatically converted with CaCS. However, this substrate was not converted efficiently and despite the usage of 220 mg of substrate only some trace compounds could be observed by GC/MS (Figure S85).

In summary, the diterpene synthase CaCS from *C. acidiphila* was functionally characterised and found to produce the novel 6‐6‐6 tricyclic diterpene alcohol catenul‐14‐en‐6‐ol (**1**) and two 6‐7‐5 tricyclic compounds, isocatenula‐2,14‐diene (**2**) and isocatenula‐2(6),14‐diene (**3**). Extensive labelling studies gave detailed insights into the cyclisation cascade and the absolute configurations of all three products. Furthermore, substrate analogues with a saturated double bond were synthesised and enzymatically converted, leading for 14,15‐dihydro‐GGPP to a series of derailment products that further supported the mechanistic model for the cyclisation cascade. As substrate analogues may adopt a different conformation in the enzyme than the native substrate, the absolute configurations and cyclisation mechanisms to all derailment products were also investigated by labelling experiments, demonstrating that all compounds are formed from a common substrate fold. Some of the obtained derailment compounds showed structural similarities to known natural products from termites and soft corals and also allowed for a deeper understanding of their chemistry. However, the starting point of our study was the observed diterpene production by *C. acidiphila*, and although by the present work a diterpene synthase with interesting products has been discovered, none of these diterpenes showed an identical mass spectrum to the compounds observed in headspace extracts from *C. acidiphila* (compare Figures S1 and S5), suggesting that a different DTS may be responsible for its biosynthesis. Its discovery will be on the list of our future research.

## Conflict of interest

The authors declare no conflict of interest.

## Supporting information

As a service to our authors and readers, this journal provides supporting information supplied by the authors. Such materials are peer reviewed and may be re‐organized for online delivery, but are not copy‐edited or typeset. Technical support issues arising from supporting information (other than missing files) should be addressed to the authors.

Supplementary
