# Supplementary material for: Diterpene Biosynthesis in Catenulispora acidiphila: On the Mechanism of Catenul‐14‐en‐6‐ol Synthase
Source: Angew Chem Int Ed Engl. 2020 Dec 10;60(3):1488–92. doi: 10.1002/anie.202014180 (PMC7839432; doi:10.1002/anie.202014180)
Supplement: Supplementary file 1 — Supplementary [file ANIE-60-1488-s001.pdf]

Angewandte Chemie
Eine Zeitschrift der Gesellschaft Deutscher Chemiker

Supporting Information

Diterpene Biosynthesis in *Catenulispora acidiphila*: On the Mechanism of Catenul-14-en-6-ol Synthase

Geng Li, Yue-Wei Guo, and Jeroen S. Dickschat**

anie_202014180_sm_miscellaneous_information.pdf

Strains and culture conditions

Catenulispora acidiphila DSM 44928 was obtained from the Leibniz Institute DSMZ – German Collection of Microorganisms and Cell Cultures GmbH. The strain was cultivated in 65 GYM medium (4.0 g glucose, 4.0 g yeast extract, 10.0 g malt extract, dissolved in 1 L distilled water, pH 5.5) at 28 °C. For agar plates $CaCO_3$ (2.0 g/L) and agar (12.0 g/L) were added to the medium.

CLSA headspace extraction

The volatile compounds were obtained from an agar plate culture of *C. acidiphila* by a closed loop stripping apparatus (CLSA).[1] The emitted compounds were collected by charcoal after 24 h, eluted with dichloromethane (30μL) and analysed by GC/MS immediately.

GC/MS and GC/MS-QToF analyses

An Agilent (Santa Clara, CA, USA) 7890B GC, using a HP5-MS fused silica capillary column (30 m, 0.25 mm i. d., 0.50 μm film) was connected to a 5977A mass detector to record GC/MS data. GC parameters were 1) inlet pressure: 77.1 kPa, He at 23.3 mL/min, 2) injection volume: 2 μL or 1 μL, 3) temperature program: 5 min at 50 °C, then increasing at 5 °C min^{-1} or 10 °C min^{-1} to 320 °C, 4) 60 s valve time, and 5) carrier gas: He at 1.2 mL/min. MS parameters were 1) source: 230 °C, 2) transfer line: 250 °C, 3) quadrupole: 150 °C and 4) electron energy: 70 eV.

To record high resolution MS data, a 7890B GC equipped with a HP5-MS fused silica capillary column (30 m, 0.25 mm i. d., 0.50 μm film) was connected to a 7200 accurate-mass Q-TOF detector (Agilent). GC parameters were 1) temperature program: 5 min at 50 °C increasing at 5 °C min^{-1} to 320 °C, 2) injection volume: 1 μL, 3) split ratio: 100:1, 60 s valve time, and 4) carrier gas: He at 1 mL/min. MS parameters were 1) inlet pressure: 83.2 kPa, He at 24.6 mL/min, 2) transfer line: 250 °C, 3) electron energy 70 eV.

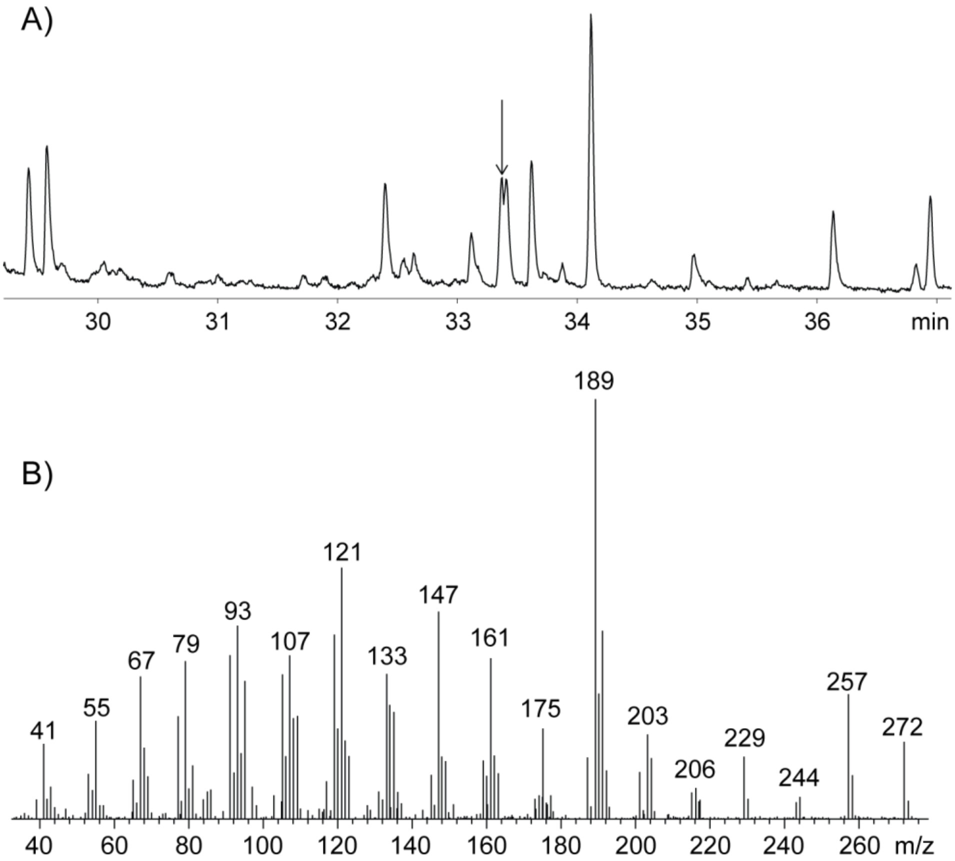

Figure S1. GC/MS analysis of the volatiles from *Catenulispora acidiphila*. A) Total ion chromatogram. B) MS of an unknown diterpene.

Construction of phylogenetic tree

The phylogenetic tree was constructed from 3267 amino acid sequences of bacterial terpene synthase homologs that were identified from sequenced bacteria through a BLAST search and verified by individual inspection for the presence of the highly conserved motifs in type I terpene synthases.[2-5] The tree was constructed using the tree builder function of Geneious (alignment type: global alignment with free end gaps, cost matrix: Blosum45, genetic distance model: Jukes-Cantor, tree build method: neighbor-joining, gap open panelty: 8, gap extension penalty: 2). The largest branches in the tree representing functionally characterised enzymes[4,6-17] and their close homologs that likely have the same function are shown in green and pink. The blue arrow points to catenul-14-en-6-ol synthase from *C. acidiphila* (CaCS).

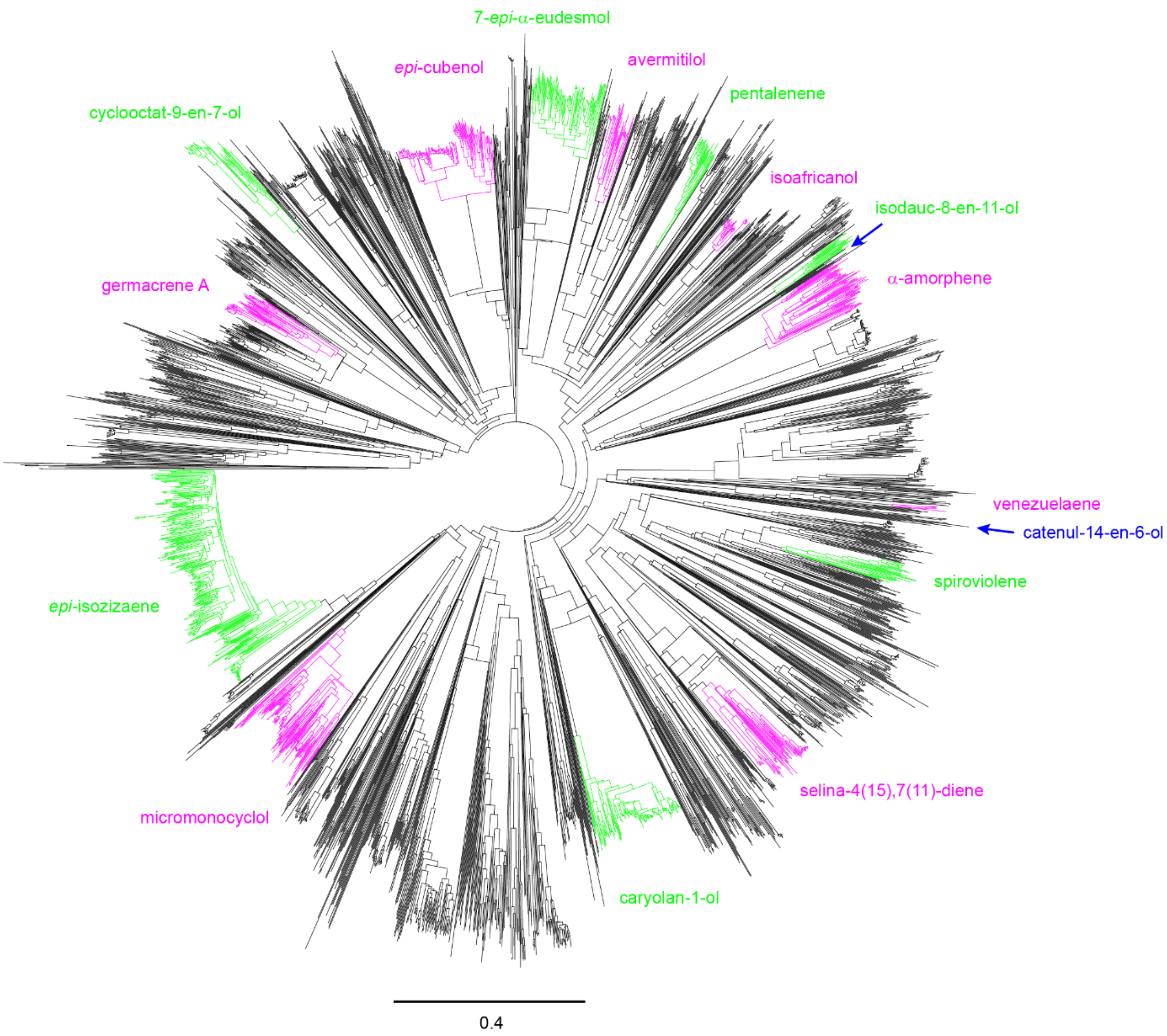

Figure S2. Phylogenetic tree of bacterial terpene synthase homologs. Blue arrows point to terpene synthases from *C. acidiphila*. The scale bar indicates substitutions per site.

```
MPADEITRLFDLPEFRLLPGERRAAWGPELDDHVIDFACRTGLLTTPAARRYYASQRIGTMC
AYVVPGALSPRRYEIYGELMTWFFIYDDWAEQLGNHLSPQQVSGVTDVVHTWFAEDERDVVM
LDAPLAQSMRGIWAKLQEDTSLEWRRRLLAETDGYLRTAAEEAILVSTGRVNSFGKASELRP
TATAAAPVFMMAEHSYGIEIPMDVVRHPLVRKAMRAAAAAIAYGNDIIGLKSDLLRGIRDNL
VLSLQQEYGGDLQLNVERAAEHYQRAAGTLTALREDPEAGGREDVAVFFQILEDWVYEGVKW
QLRDTDRYSSTVRLTQEENPNQLLALAASSALADC
```

Figure S3. Amino acid sequence of the type I terpene synthase from *C. acidiphila* investigated in this study (accession number WP_015792165, gene locus tag CACI_RS17545). Highly conserved motifs are highlighted in yellow.[2-5]

Isolation of genomic DNA[18]

Bacterial cells from a 65. GYM (pH 5.5) liquid culture (200 mL) were harvested by centrifugation, resuspended in SET buffer (5 mL, 75 mM NaCl, 25 mM EDTA, 20 mM Tris/HCl, pH 8.0) and incubated with lysozyme solution (1 mg/mL) for 30 min at 37 °C. Proteinase K solution (100 μL, 1 mg/mL) was added and the solution was mixed. The reaction mixture was incubated for 1 h at 55 °C, followed by addition of 10% SDS (600 μL) and mixing by inversion. Phenol/chloroform (5 mL) was added followed by mixing and centrifugation for 1 h at 14600 g. The aqueous layer was transferred to a fresh tube and ethanol (60% vol.) was added for precipitation of gDNA. After centrifugation and washing with 70% ethanol the gDNA was re-dissolved in distilled water.

Gene cloning

PCRs were performed with Q5 polymerase (New England Biolabs, Ipswich, MA, USA) and ligations were performed with In-Fusion HD cloning kit (Clontech, Saint-Germain-en-Laye, France) according to the manuals provided by the manufacturers. The fragment ACU_72436 was amplified from gDNA of *C. acidiphila* DSM 44928 using primers ACU72436_pYE_Fw and ACU72436_pYE_Rv, and cloned into a linearised pYE-Express fragment that was obtained from the cyclic plasmid by PCR using primers pYE-Fw and pYE-Rv. The obtained plasmid pYE-ACU72436 was used for protein expression in *E. coli*.

Cells were plated on LB agar plates followed by incubation at 37 °C overnight. Single colonies were selected and used to inoculate LB medium (6 mL) liquid cultures with kanamycin (6 μL; 50 mg/mL). After 24 h of growth plasmid DNA was isolated and checked for correct insertion of the desired gene by analytical digest with XhoI and PvuII and by sequencing. The obtained plasmid was named pYE-ACU72436.

Table S1. Primers used in this study.

Primer	Sequence
ACU72436_pYE_Fw	AGCATGACTGGTGGAATGCCGGCCGATGAGATCAC
ACU72436_pYE_Rv	GGTGGTGCTCGAGTGTCAGCAGTCCGCGAGCGC
pYE-Fw	CACTCGAGCACCACCACCAC
pYE-Rv	TCCACCAGTCATGCTAGCCATATGG

Gene expression and protein purification

A preculture of *E. coli* BL21(DE3) transformed with pYE-ACU72436 was grown in LB medium with kanamycin (50 μg/mL) overnight with shaking at 37 °C. Gene expression cultures were inoculated with the preculture (1/1000) and grown in LB medium containing kanamycin (50 μg/mL) with shaking at 37 °C until OD_{600} = 0.4 – 0.6 was reached. After cooling the cultures to 18 °C, enzyme expression was induced

by the addition of aqueous IPTG solution (400 mM, 1/1000). The cultures were shaken at 18 °C overnight. The cells were harvested via centrifugation (3600 rpm, 30 min, 4 °C), resuspended in binding buffer (10 mL/L culture; 20 mM Na_2HPO_4, 500 mM NaCl, 20 mM imidazole, 1 mM $MgCl_2$, pH = 7.4, 4 °C) and lysed by ultrasonication (3x 1 min) on ice. The cell debris was removed by centrifugation (14600 g, 7 min, 4 °C) and the soluble protein fraction was loaded on a Ni^{2+}-NTA superflow affinity chromatography column (Qiagen, Venlo, Netherlands) equilibrated with binding buffer. The column was washed with binding buffer (2x 10 mL/L culture) and the desired protein was eluted with elution buffer (2x 10 mL/L culture; 20 mM Na_2HPO_4, 500 mM NaCl, 500 mM imidazole, 1 mM $MgCl_2$, pH = 7.4, 4 °C).

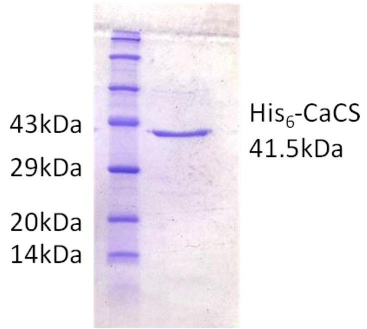

Figure S4. SDS-PAGE analysis of recombinant CaCS from *C. acidiphila*.

Incubation experiments with recombinant CaCS

Test incubations were carried out to identify the substrate scope of recombinant CaCS with GPP, FPP, GGPP or GFPP (1 mg) dissolved in substrate buffer (1.0 mL; 25 mM NH_4HCO_3) and diluted with binding buffer (1.5 mL) and incubation buffer (3.0 mL; 50 mM Tris/HCl, 10 mM $MgCl_2$, 20% glycerol, pH = 8.2). A protein preparation (0.5 mL x 4) obtained from 400 mL expression culture was added to each substrate, followed by incubation with shaking at 28 °C overnight. The products were extracted with hexane (100 μL), the extracts were dried with $MgSO_4$ and analysed by GC/MS. Large scale diterpene preparations were done by dissolving GGPP (trisammonium salt, 100 mg, 0.2 mmol) in substrate buffer (20 mL). This solution was slowly added within 1 h to a stirred mixture of protein preparation of recombinant CaCS (80 mL; from 8 L expression culture, 1.3 mg/mL), binding buffer (120 mL) and incubation buffer (200 mL). The reaction mixtures were incubated overnight at 28 °C. The reaction mixtures were extracted with pentane (3x 100 mL), and the extracts were dried with $MgSO_4$ and concentrated in vacuo (600 mbar, 35 °C). Column chromatography on silica gel with pentane yielded the hydrocarbons **2** and **3**, then changing to pentane/Et_2O (2:1) elution yielded pure **1**.

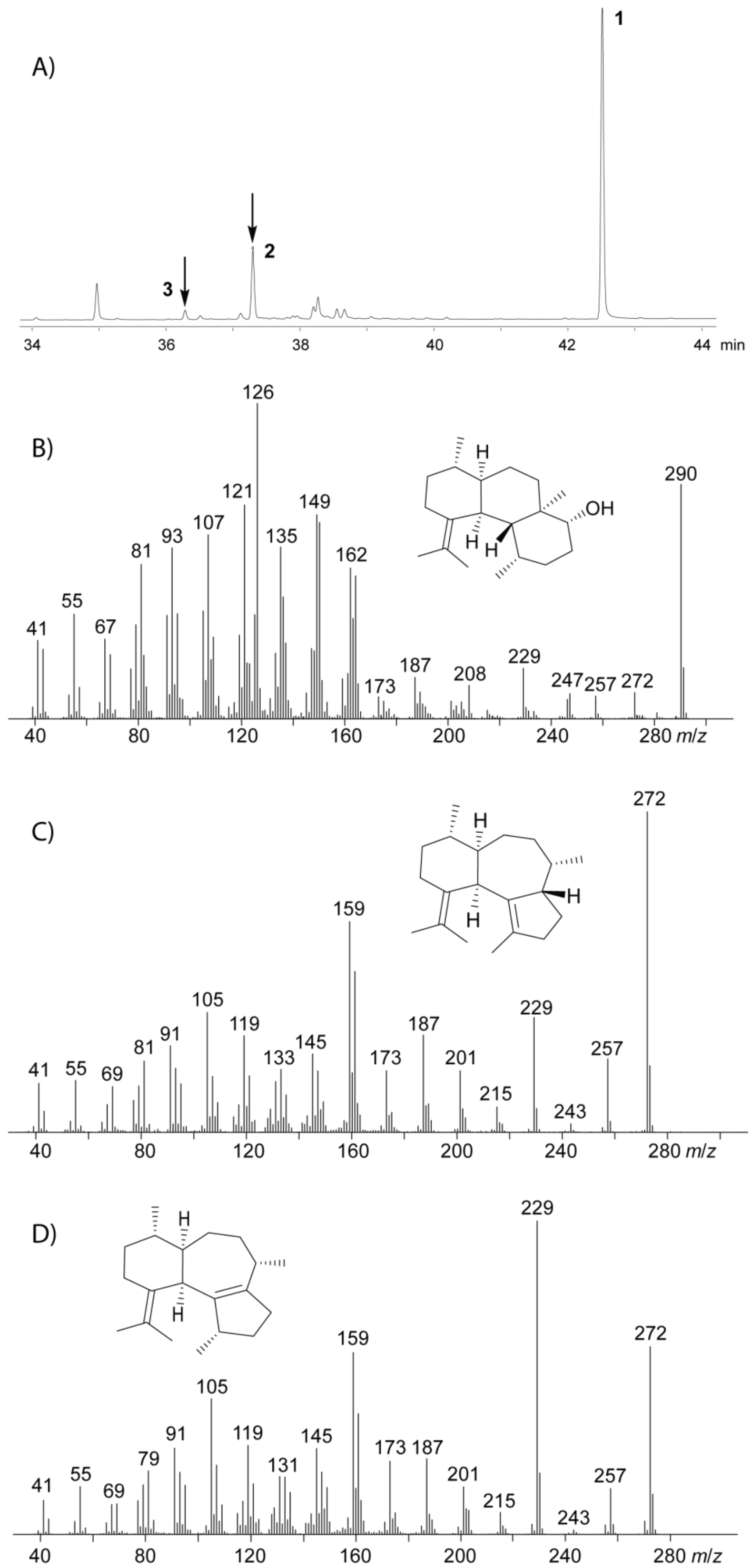

Figure S5. A) Total ion chromatogram of the products obtained from GGPP with CaCS. B) EI mass spectrum of the main product **1**. C) EI mass spectrum of the side product **2**. D) EI mass spectrum of the side product **3**.

HPLC purifications

For analytical separations a PLATINblue-series UHPLC system (Knauer, Berlin, Germany) was used, equipped with a photo diode array detector PDA-1 (190-1000 nm). Separation of **2** and **3** was performed using a Nucleodur 110-1.8 Gravity C18 column (1,8 µm; 2.0 x 100 mm, Macherey-Nagel, Düren, Germany) and an isocratic solvent mixture of acetonitrile/water (95:5) with a flow of 0.5 mL min^{-1} (301 bar). Separation of **5** and **6** (prepared as on page 53 of SI) was performed using a Knauer Eurospher II 100-3 C18P column (3.0 µm; 2.0 x 100 mm) and an isocratic solvent mixture of methanol/water (95:5) with a flow of 0.5 mL min^{-1} (215 bar). The UV-Vis absorption was monitored at 205 nm. Observed elution times were 8.39 min (**2**), 9.82 min (**3**), 6.55 min (**5**) and 7.22 min (**6**).
Semi-preparative scale HPLC purifications were performed on the same system with a Nucleodur 110-5 Gravity C18 column (5 µm; 250 x 10 mm) for compounds **2** and **3**. For elution an isocratic mixture of acetonitrile/water (95/5) at 8 mL min^{-1} (103 bar) was used. For compounds **5** and **6** a Knauer Eurospher II 100-5 C18P column (5.0 µm; 8.0 x 250 mm) with a pre-column (30 x 8 mm) was used. Elution was performed with an isocratic mixture of methanol/water (95/5) at 5 mL min^{-1} (141 bar).

NMR spectroscopy

NMR spectra were recorded on a Bruker (Billerica, MA, USA) Avance I (500 MHz), Avance III HD Prodigy (500 MHz) or an Avance III HD Cryo (700 MHz) NMR spectrometer. Spectra were referenced against solvent signals (^{1}H-NMR, residual proton signals: $CDCl_3$ δ = 7.26 ppm, C_6D_6 δ = 7.16, D_2O δ = 4.79; ^{13}C-NMR: $CDCl_3$ δ = 77.16 ppm, C_6D_6 δ = 128.06 ppm).[19]

Table S2. NMR data of catenul-14-en-6-ol (**1**) in C_6D_6 recorded at 298 K.

C[a]	type	^{1}H[b]	^{13}C[b]
1	CH	2.94 (dd, *J* = 11.9, 4.0)	38.6
2	CH	1.59 (m)	42.9
3	CH	1.50 (m)	28.6
4	CH_2	1.39 (m, H_β)	32.2
		1.39 (m, H_α)	
5	CH_2	1.45 (m, H_β)	27.0
		1.61 (m, H_α)	
6	CH	3.12 (dt, *J* = 10.7, 3.6)	80.8
7	C_q	–	40.9
8	CH_2	1.18 (td, *J* = 13.1, 12.5, 3.1, H_β)	35.3
		1.58 (m, H_α)	
9	CH_2	1.68 (m, H_β)	23.8
		1.54 (m, H_α)	
10	CH	1.25 (m)	46.2
11	CH	1.81 (m)	28.1
12	CH_2	1.68 (m, H_β)	37.9
		0.95 (m, H_α)	
13	CH_2	1.63 (m, H_β)	26.4
		2.43 (d, *J* = 13.0, H_α)	
14	C_q	–	135.4
15	C_q	–	120.8
16	CH_3	1.76 (d, *J* = 1.4)	20.8
17	CH_3	1.65 (s)	20.3
18	CH_3	0.82 (d, *J* = 6.2)	20.2
19	CH_3	1.03 (s)	15.1
20	CH_3	0.81 (d, *J* = 7.3)	15.3

[a] Carbon numbering as shown in main text. [b] Chemical shifts δ in ppm, multiplicity: s = singlet, d = doublet, t = triplet, m = multiplet, coupling constants *J* are given in Hertz.

Catenul-14-en-6-ol (1). Yield: 4.2 mg (14.5 µmol, 7%), from 100 mg (200 µmol) GGPP trisammonium salt. TLC (pentane/Et_2O = 4/1): R_f = 0.25. IR (diamond ATR): $\tilde{\nu}$ = 3384 (w), 2964 (m), 2923 (s), 2863 (m), 1455 (m), 1382 (w), 1116 (w), 1060 (m), 1022 (w), 987 (w) cm^{-1}. HR-MS (Q-TOF, 70 eV): calc. for $[C_{20}H_{34}O]^+$ *m*/*z* = 290.2604; found: *m*/*z* = 290.2067. Optical rotary power: $[\alpha]_D^{20}$ = –36.7 (*c* 0.15, C_6H_6).

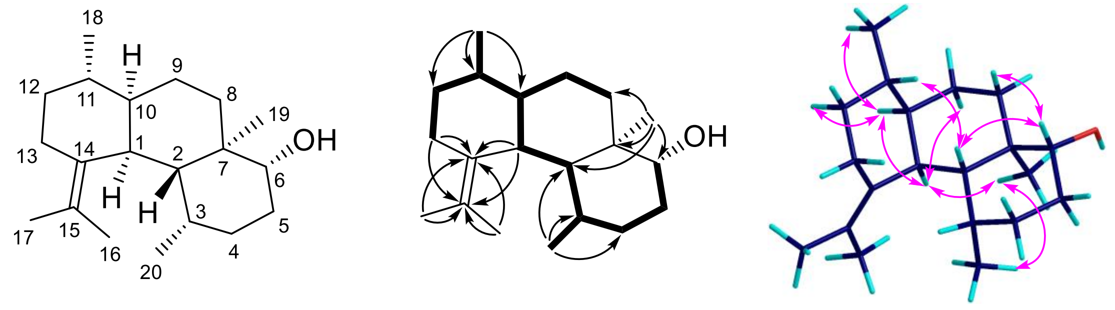

Figure S6. Carbon numbering and structure elucidation of **1**. Bold lines represent $^{1}H,^{1}H$-COSY correlations, selected HMBC correlations are represented by single-headed arrows and key NOESY correlations are indicated by double headed arrows.

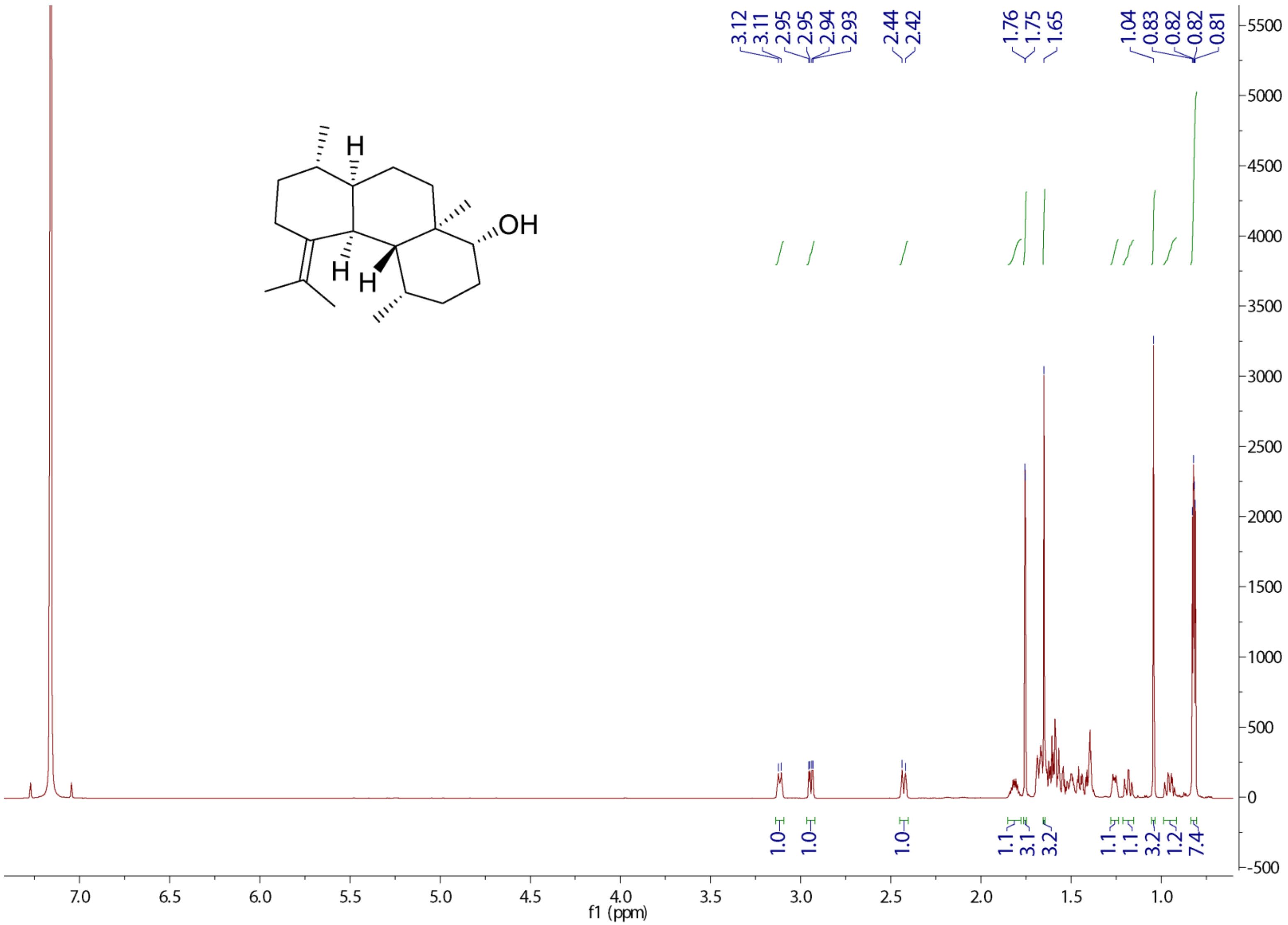

Figure S7. ^{1}H-NMR spectrum of **1** (700 MHz, C_6D_6).

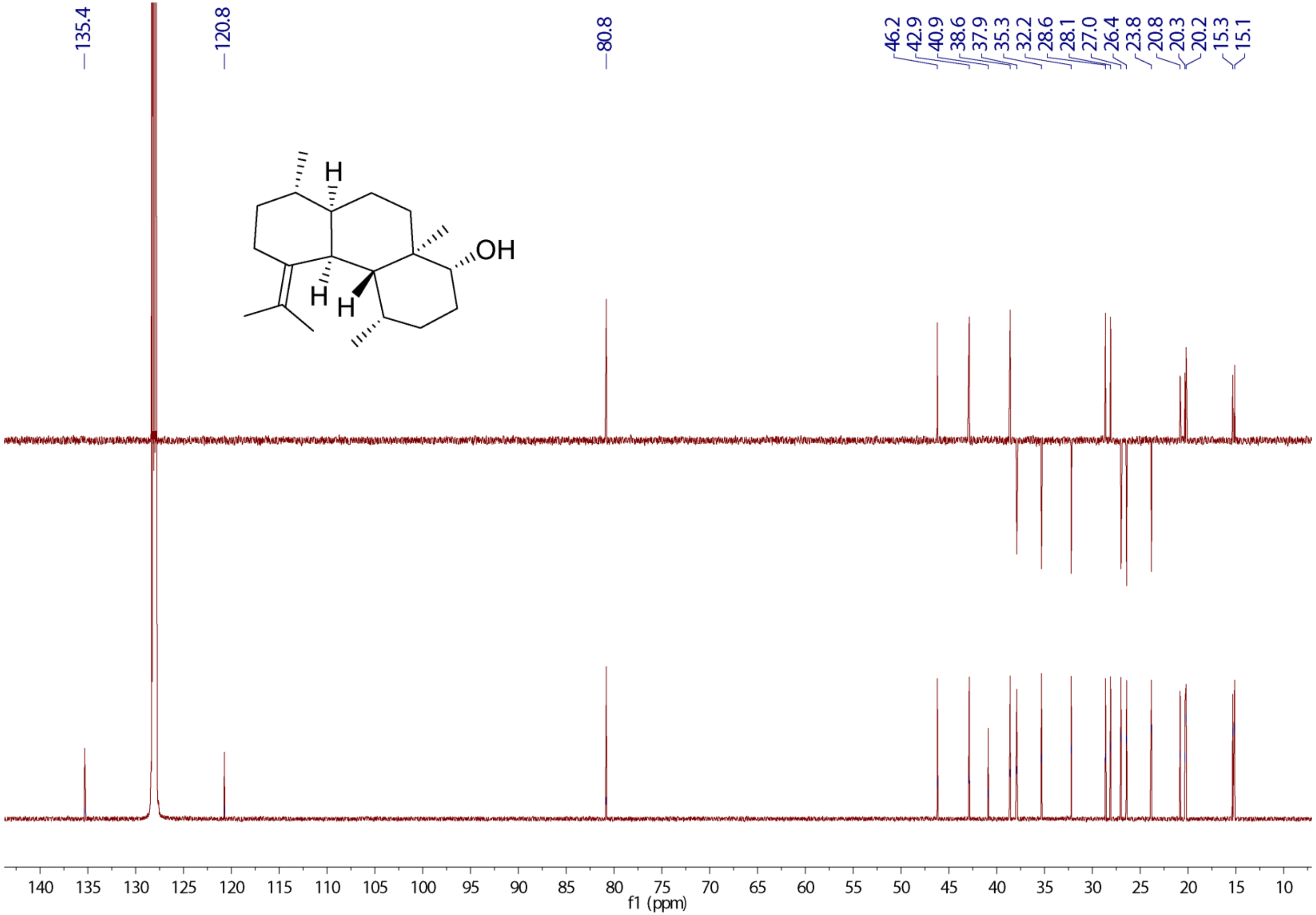

Figure S8. ^{13}C-NMR and ^{13}C-DEPT-135 spectra of **1** (175 MHz, C_6D_6).

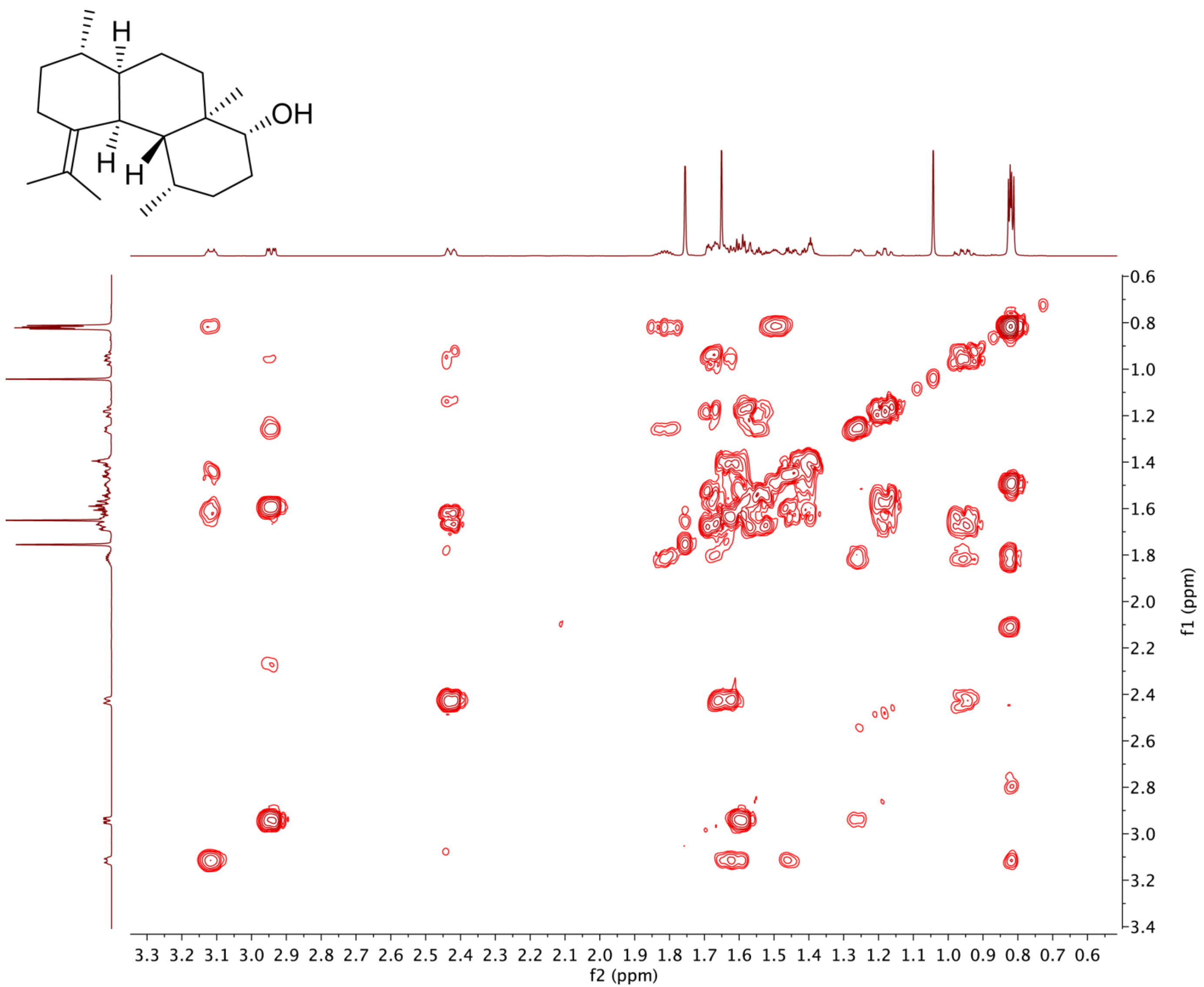

Figure S9. $^1H,^1H$-COSY spectrum of **1** (C_6D_6).

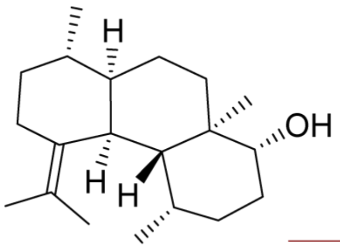

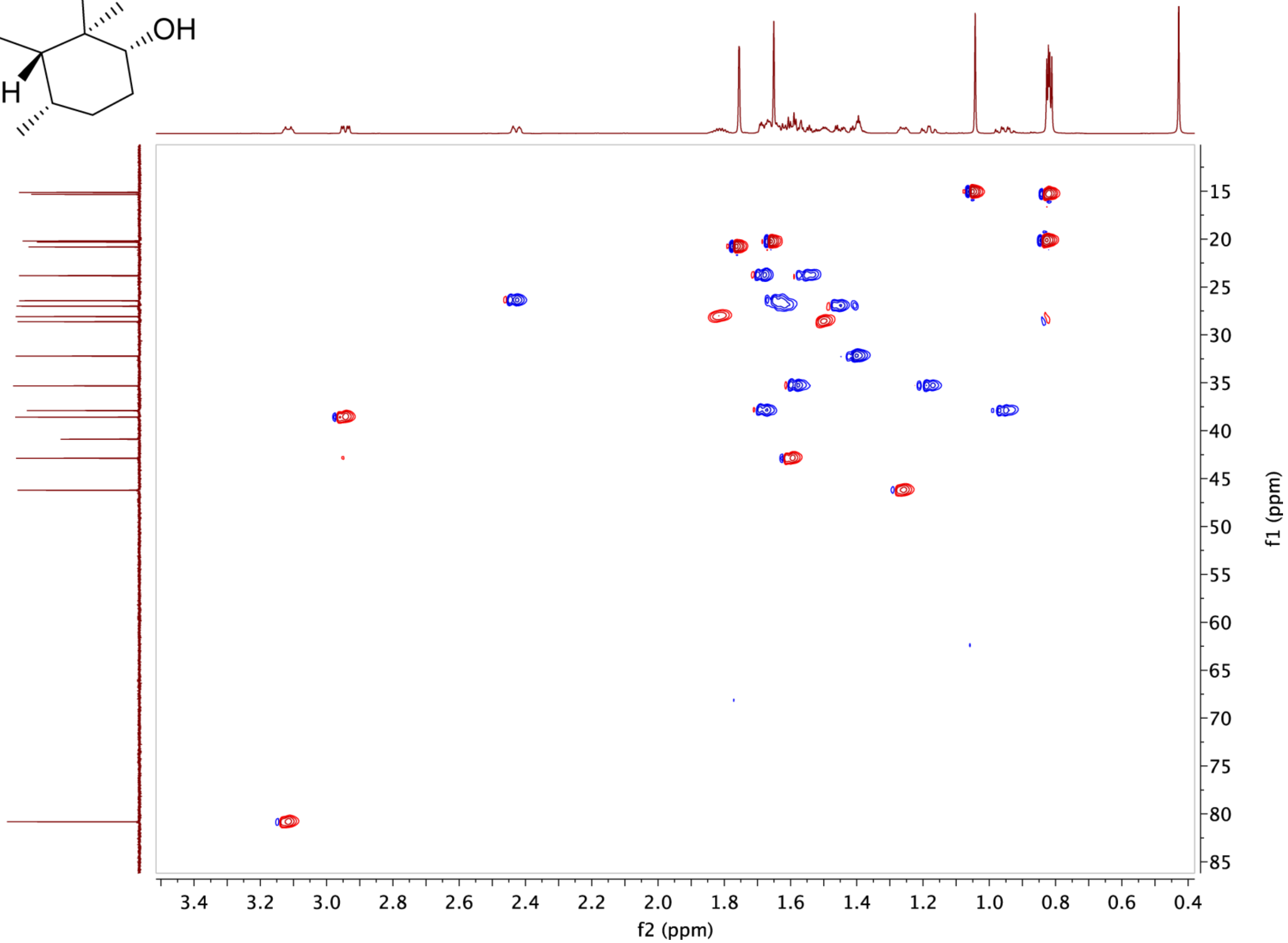

Figure S10. HSQC spectrum of **1** (C_6D_6).

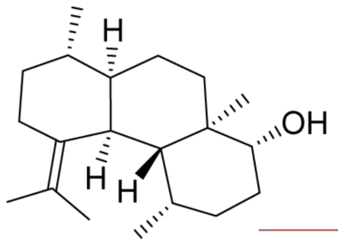

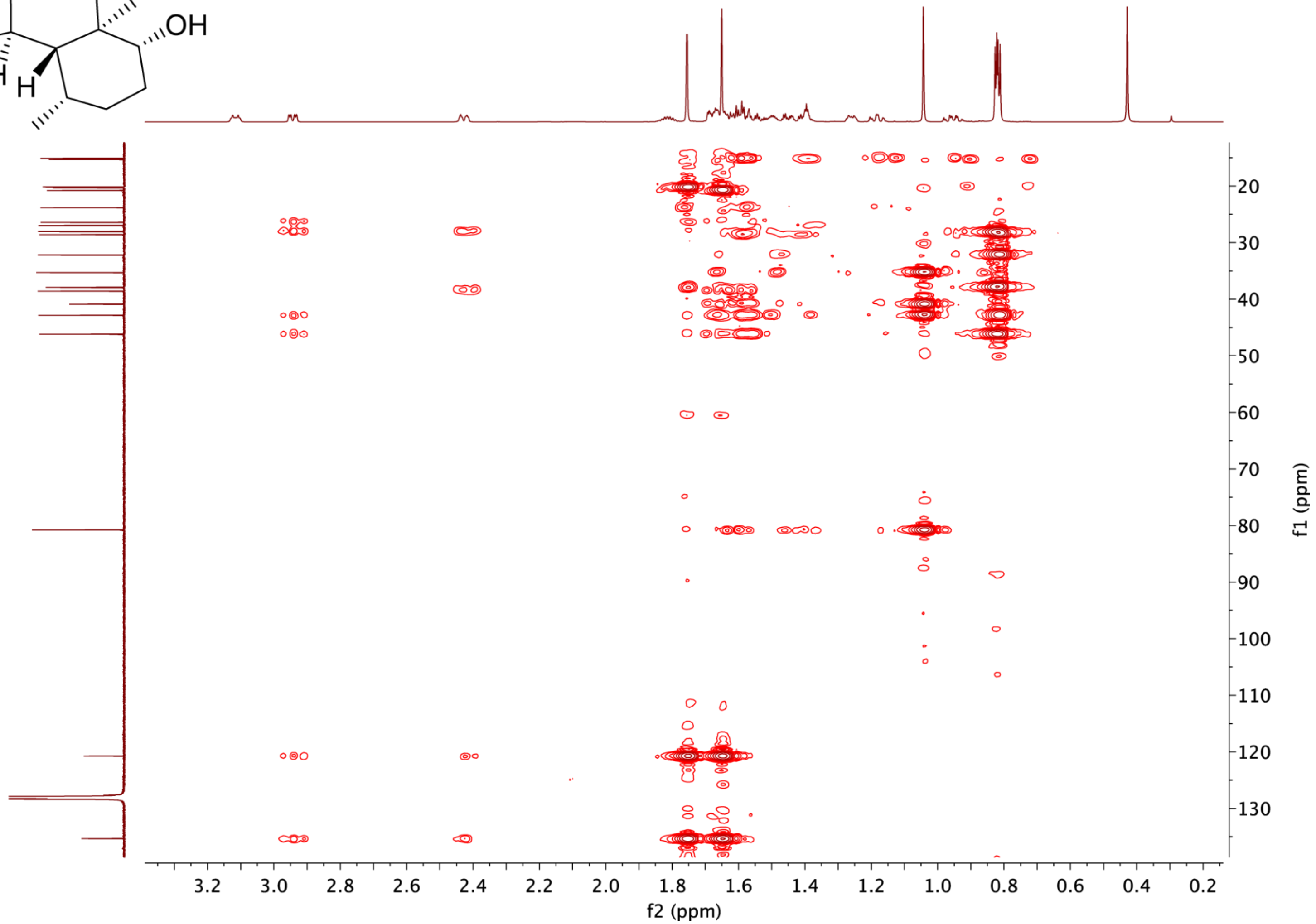

Figure S11. HMBC spectrum of **1** (C_6D_6).

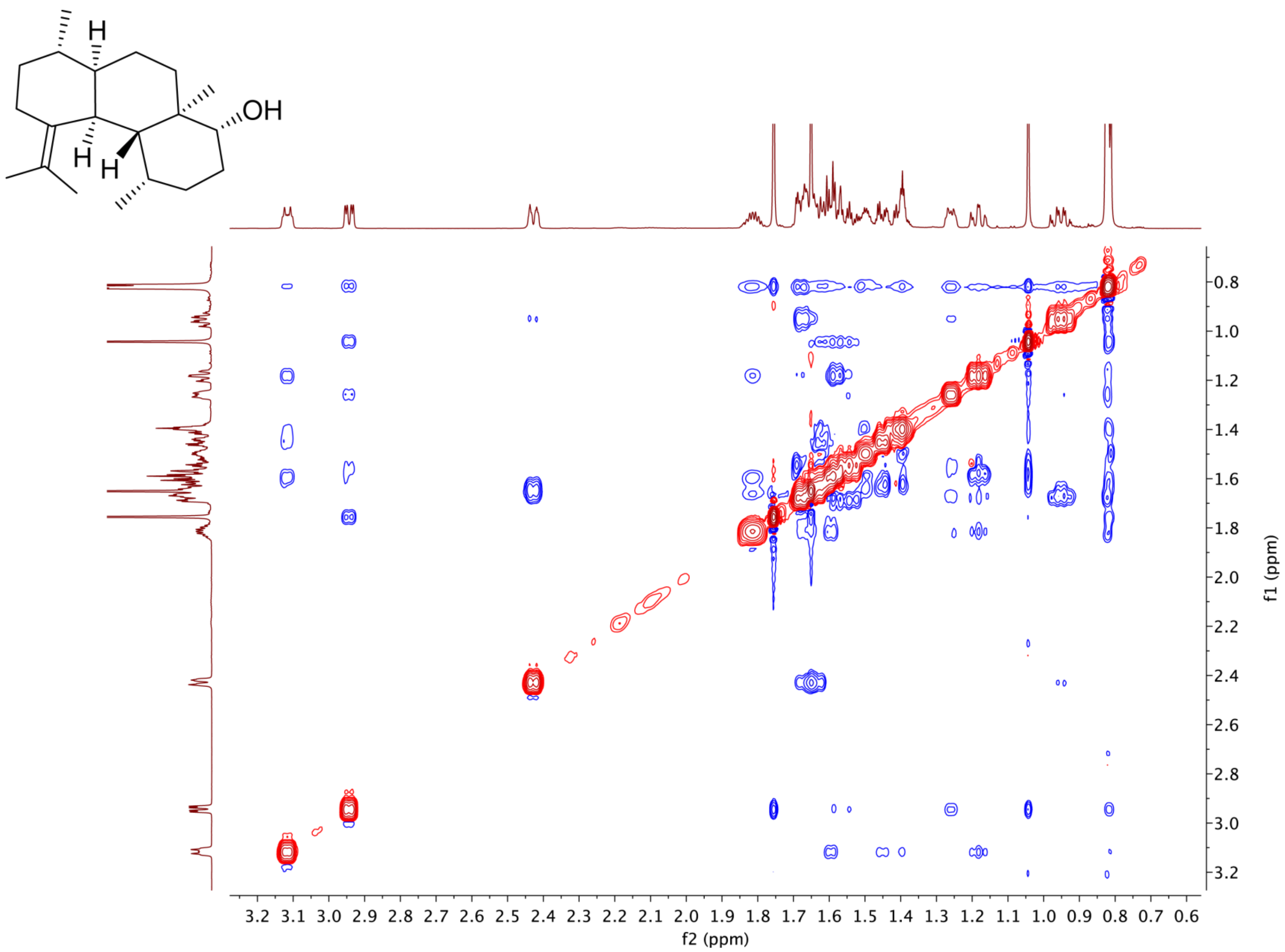

Figure S12. NOESY spectrum of **1** (C_6D_6).

Table S3. NMR data of isocatenula-2,14-diene (**2**) in C_6D_6 recorded at 298 K.

C[a]	type	^{1}H[b]	^{13}C[b]
1	CH	3.53 (s)	45.5
2	C_q	–	138.5
3	C_q	–	135.4
4	CH_2	2.20 (m, H_β)	39.1
		2.33 (m, H_α)	
5	CH_2	2.09 (m, H_β)	29.9
		1.49 (m, H_α)	
6	CH	3.17 (d, *J* = 8.8)	54.2
7	CH	1.85 (m)	39.8
8	CH_2	1.74 (m, H_β)	30.8
		1.33 (m, H_α)	
9	CH_2	1.81 (m, H_β)	27.2
		1.52 (m, H_α)	
10	CH	1.34 (m)	49.5
11	CH	1.95 (m)	31.5
12	CH_2	1.75 (m, H_β)	38.1
		0.99 (m, H_α)	
13	CH_2	1.80 (m, H_β)	27.7
		2.42 (m, H_α)	
14	C_q	–	138.3
15	C_q	–	117.7
16	CH_3	1.75 (d, *J* = 1.2)	20.7
17	CH_3	1.66 (s)	19.7
18	CH_3	0.84 (d, *J* = 7.7)	20.1
19	CH_3	0.86 (d, *J* = 8.4)	13.3
20	CH_3	1.62 (t, *J* = 1.4)	14.6

[a] Carbon numbering as shown in main text. [b] Chemical shifts δ in ppm, multiplicity: s = singlet, d = doublet, t = triplet, m = multiplet, coupling constants *J* are given in Hertz.

Isocatenula-2,14-diene (2). Yield: 0.8 mg (2.9 mmol, 1%), from 100 mg (200 µmol) GGPP trisammonium salt. TLC (pentane): R_f = 0.95. IR (diamond ATR): ṽ = 2959 (s), 2922 (s), 2858 (m), 1456 (m), 1377 (w), 1260 (m), 1094 (m), 1019 (m), 800 (m) cm^{-1}. HR-MS (Q-TOF, 70 eV): calc. for $[C_{20}H_{32}]^+$ *m*/*z* = 272.2499, found: *m*/*z* = 272.2501. Optical rotary power: $[\alpha]_D^{20}$ = –57.8 (*c* 0.09, C_6H_6).

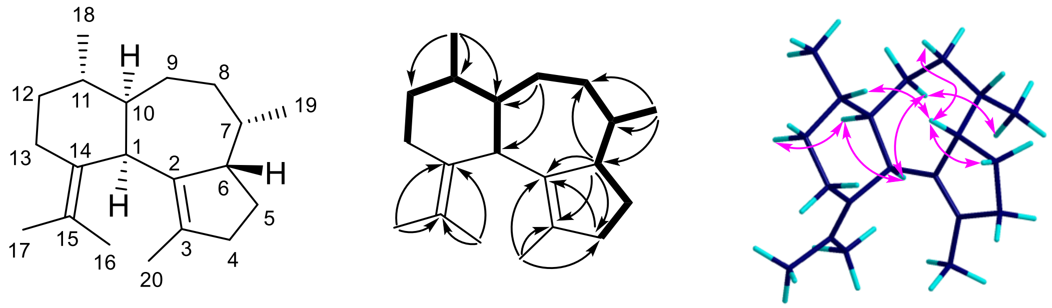

2

Figure S13. Carbon numbering and structure elucidation of **2**. Bold lines represent ^{1}H,^{1}H-COSY correlations, selected HMBC correlations are represented by single-headed arrows and key NOESY correlations are indicated by double headed arrows.

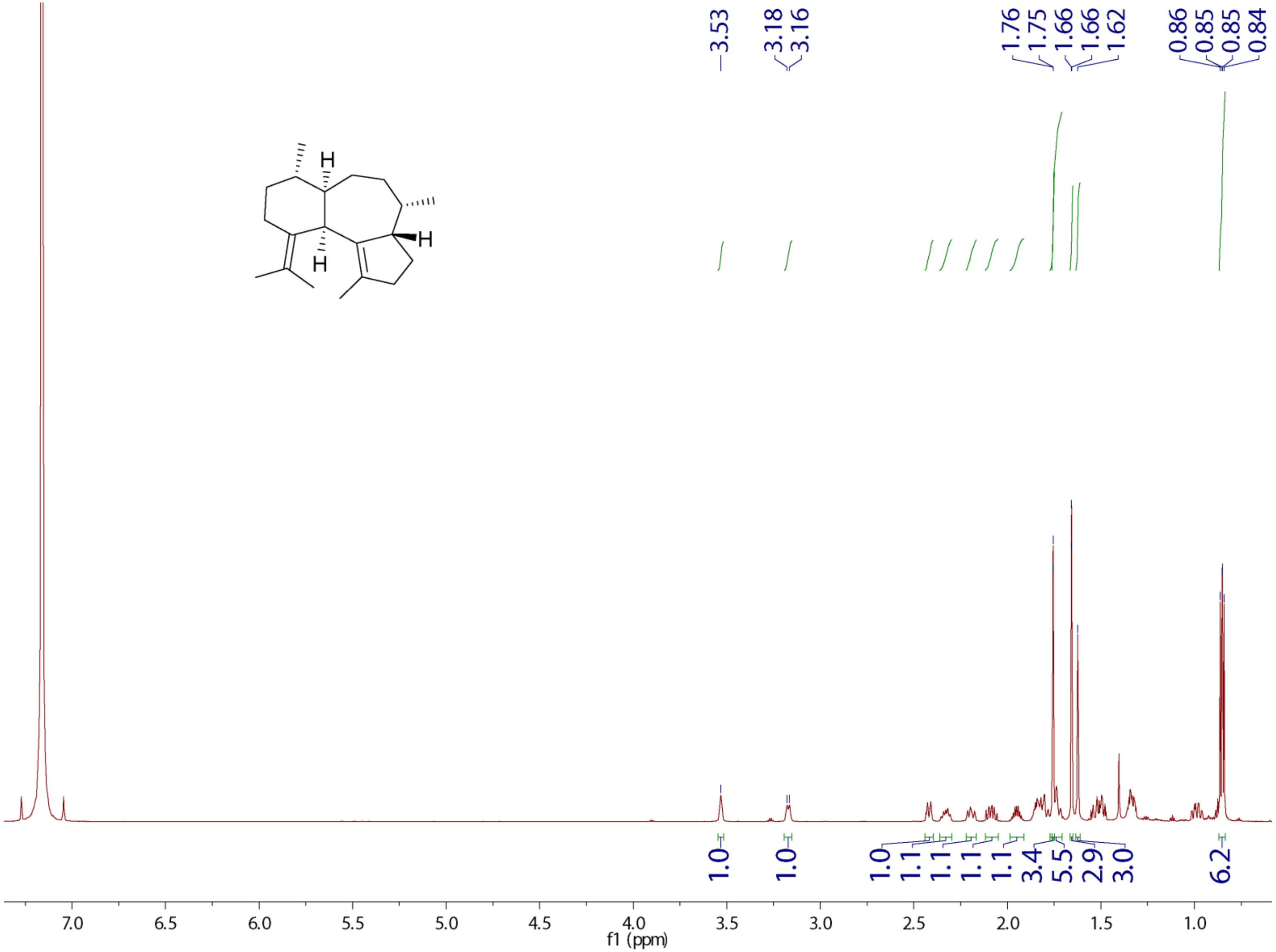

Figure S14. ^{1}H-NMR spectrum of **2** (700 MHz, C_6D_6).

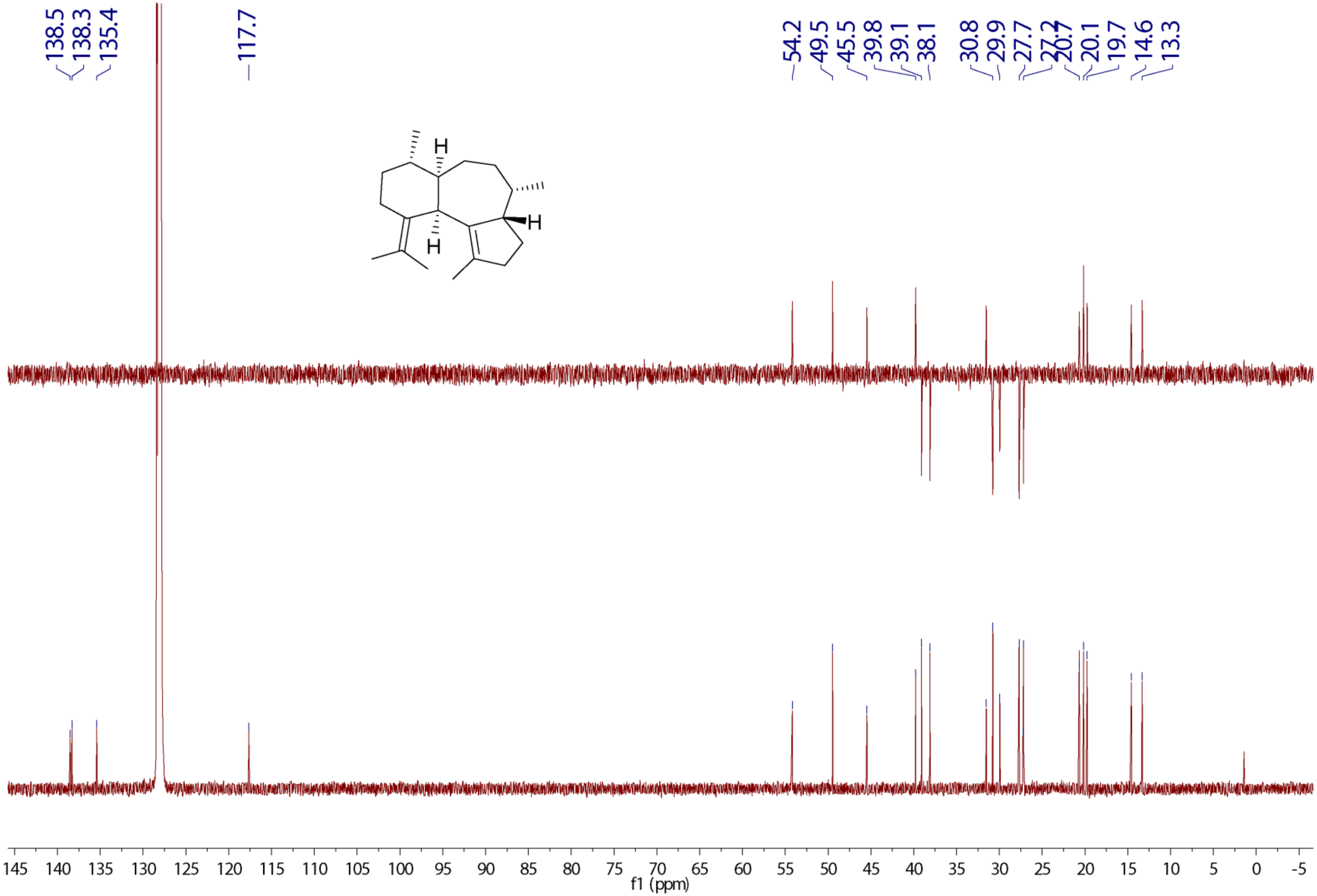

Figure S15. ^{13}C-NMR and ^{13}C-DEPT-135 spectra of **2** (175 MHz, C_6D_6).

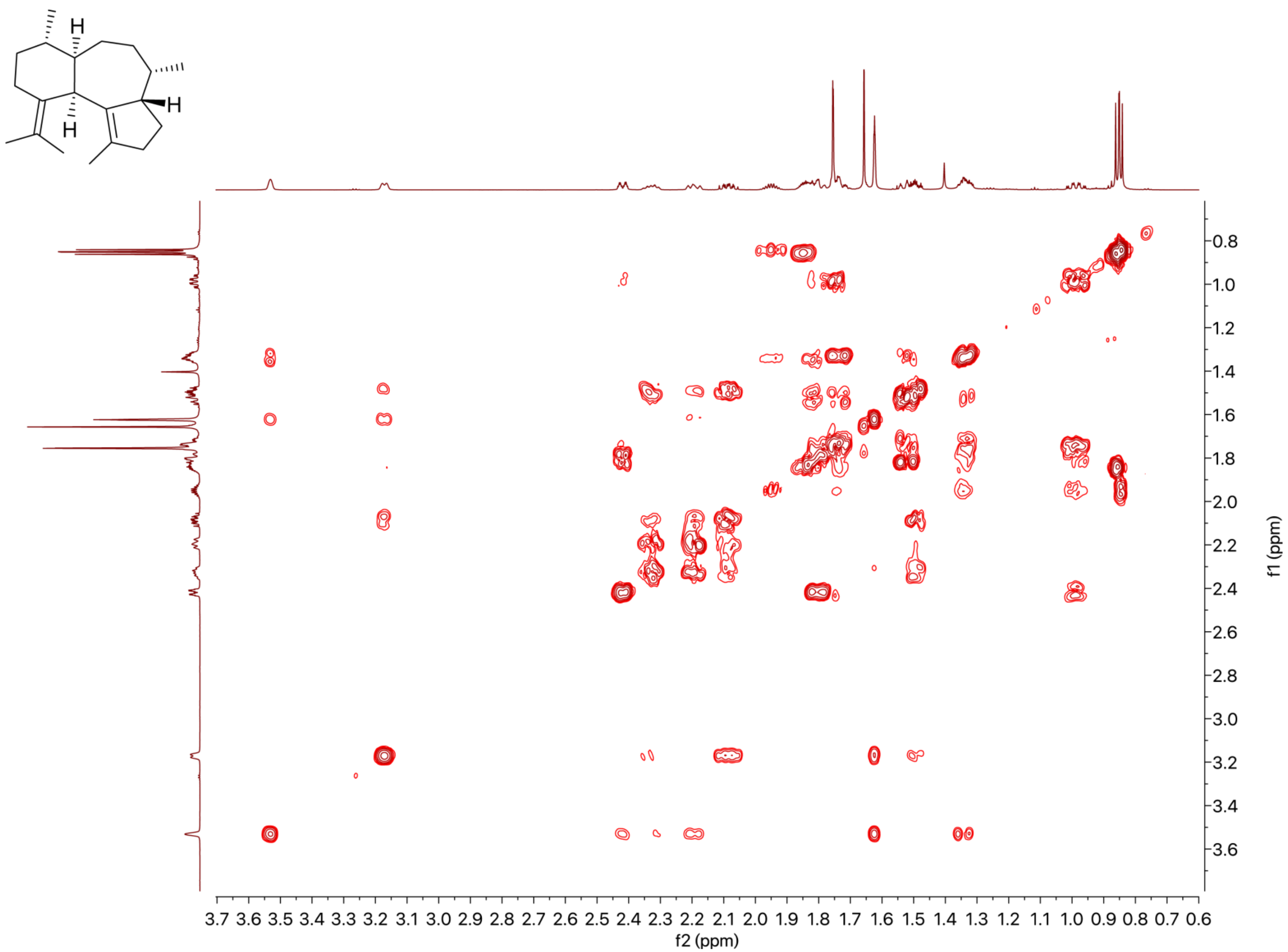

Figure S16. ^{1}H,^{1}H-COSY spectrum of **2** (C_6D_6).

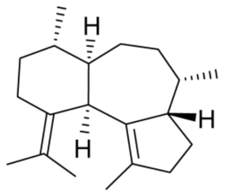

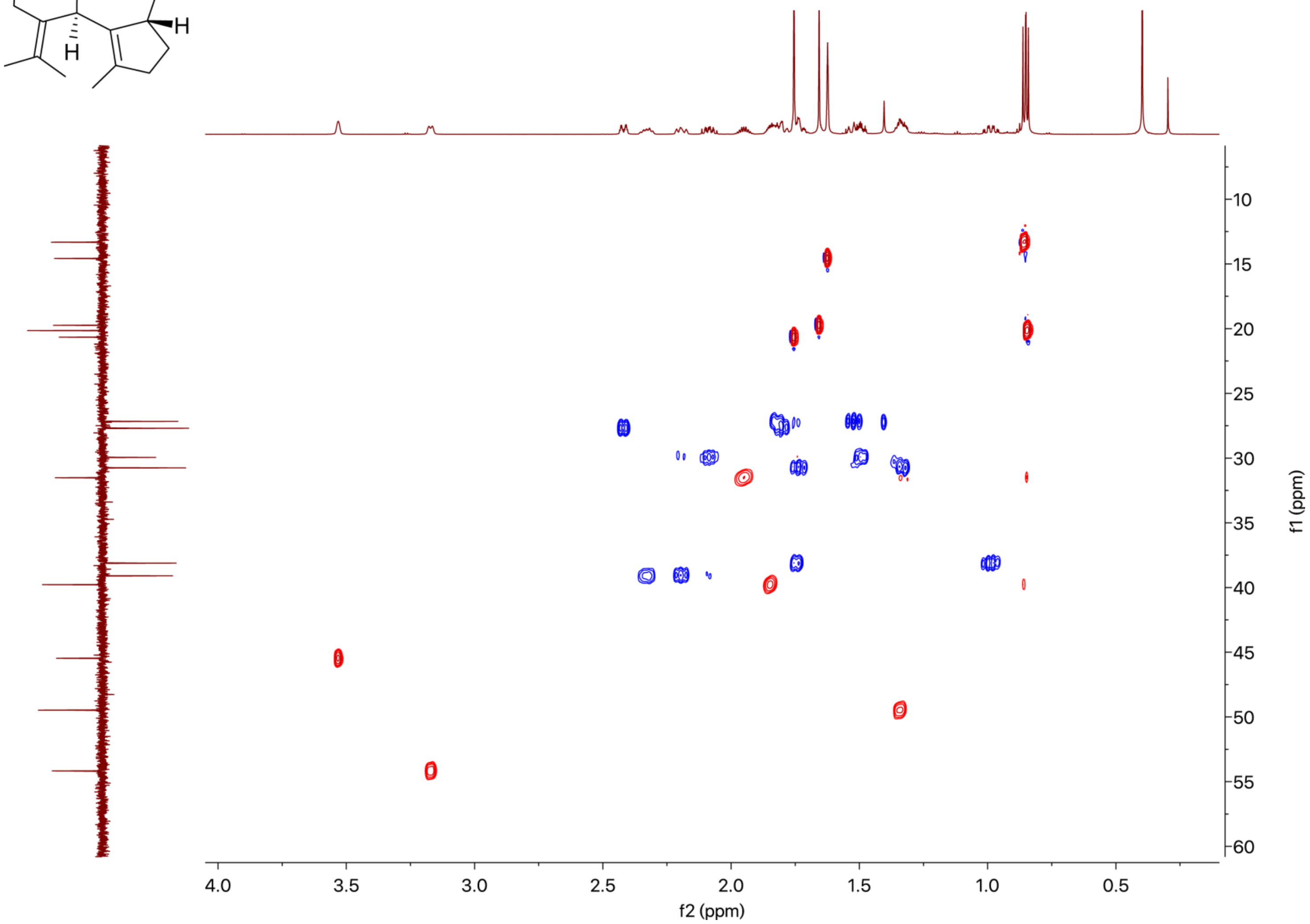

Figure S17. HSQC spectrum of **2** (C_6D_6).

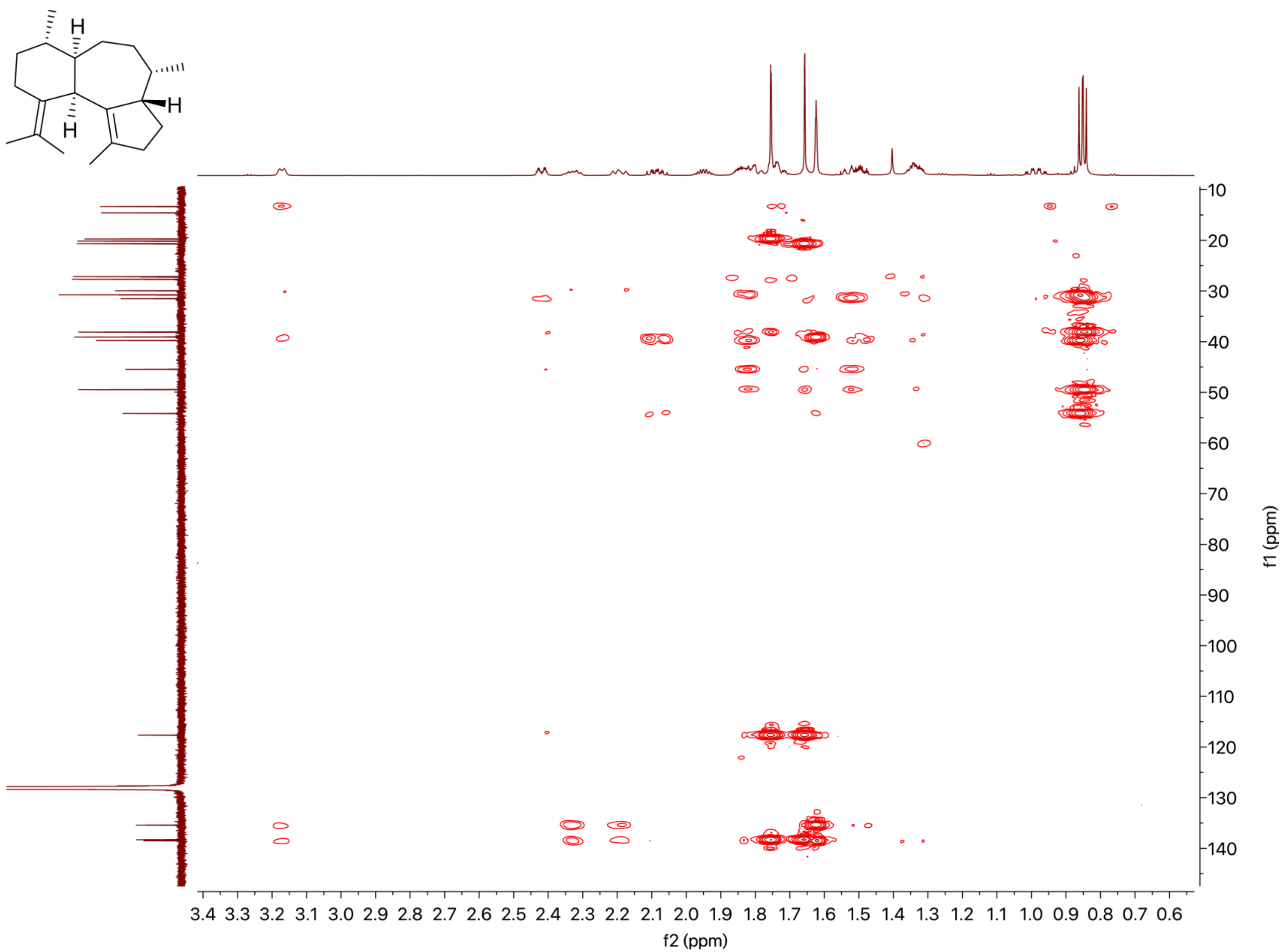

Figure S18. HMBC spectrum of **2** (C_6D_6).

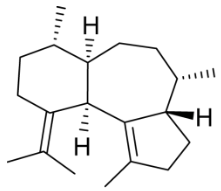

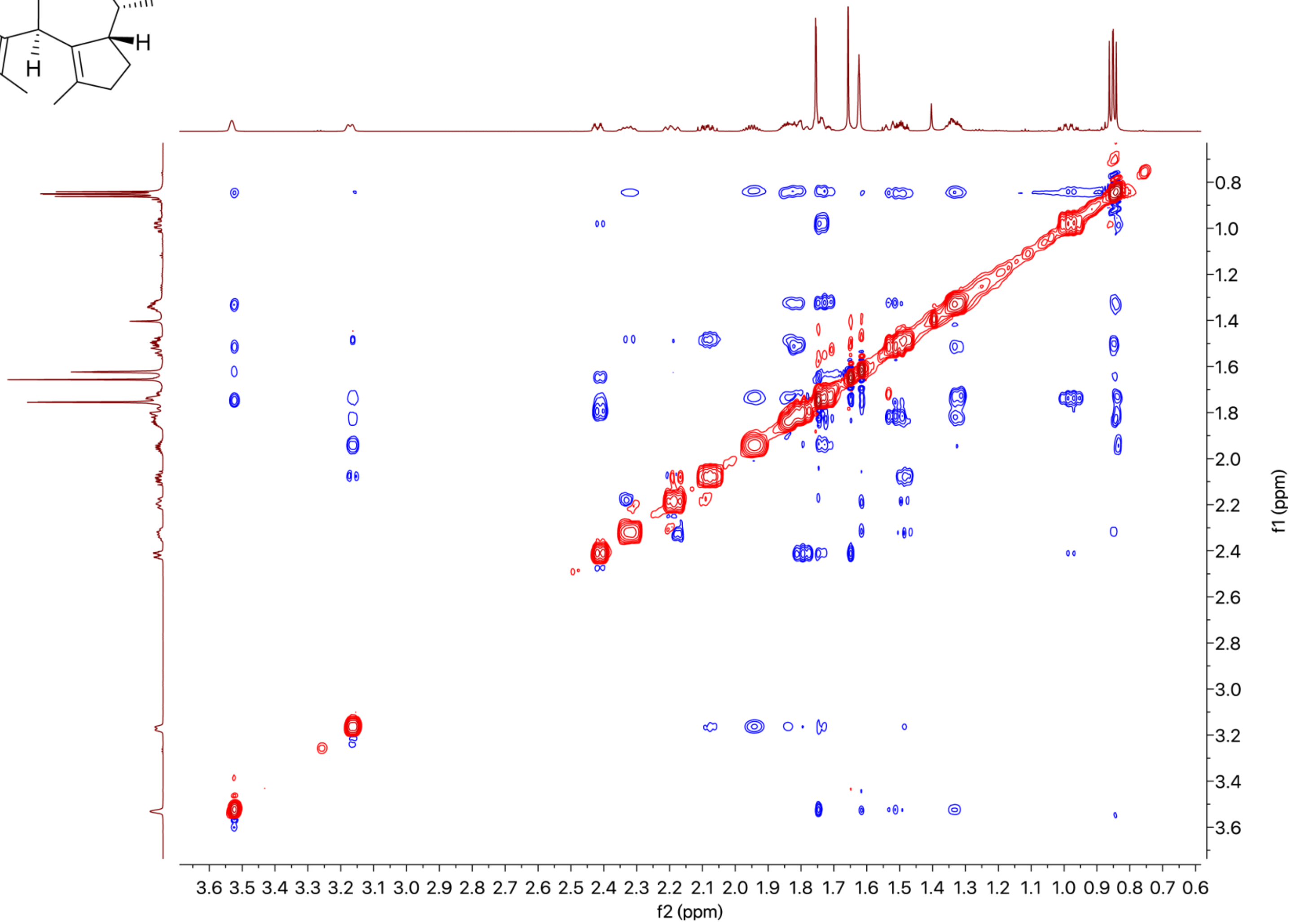

Figure S19. NOESY spectrum of **2** (C_6D_6).

Table S4. NMR data of isocatenula-2(6),14-diene (**3**) in C_6D_6 recorded at 298 K.

C[a]	type	^{1}H[b]	^{13}C[b]
1	CH	3.74 (s)	42.7
2	C_q	–	139.9
3	CH	2.36 (m)	36.2
4	CH_2	1.80 (m, H_β)	28.1
		1.36 (m, H_α)	
5	CH_2	2.05 (ddd, *J* = 15.4, 9.2, 1.9, H_α)	37.0
		2.66 (m, H_β)	
6	C_q	–	140.3
7	CH	2.62 (m)	44.4
8	CH_2	1.96 (m, H_β)	32.1
		1.44 (dd, *J* = 11.9, 7.5, H_α)	
9	CH_2	1.68 (m, H_α)	27.6
		1.80 (m, H_β)	
10	CH	1.30 (m)	48.4
11	CH	1.71 (m)	31.0
12	CH_2	1.68 (m, H_β)	37.4
		0.99 (m, H_α)	
13	CH_2	1.90 (t, *J* = 13.0, H_β)	28.7
		2.61 (m, H_α)	
14	C_q	–	135.8
15	C_q	–	120.8
16	CH_3	1.72 (d, *J* = 1.7)	20.6
17	CH_3	1.66 (d, *J* = 1.1)	20.1
18	CH_3	0.85 (d, *J* = 6.5)	19.8
19	CH_3	0.95 (d, *J* = 6.7)	19.6
20	CH_3	1.06 (d, *J* = 7.1)	19.3

[a] Carbon numbering as shown in main text. [b] Chemical shifts δ in ppm, multiplicity: s = singlet, d = doublet, t = triplet, m = multiplet, coupling constants *J* are given in Hertz.

Isocatenula-2(6),14-diene (3). Yield: 0.5 mg (1.8 μmol, 1%), from 100 mg (200 μmol) GGPP trisammonium salt. TLC (pentane): R_f = 0.95. IR (diamond ATR): ṽ = 2959 (m), 2921 (s), 2852 (m), 1447 (w), 1366 (s), 1217 (m), 1093 (m), 1021 (m), 800 (m) cm^{-1}. HR-MS (Q-TOF, 70 eV): calc. for $[C_{20}H_{32}]^+$ *m*/*z* = 272.2499, found: *m*/*z* = 272.2503. Optical rotary power: $[\alpha]_D^{20}$ = –16.0 (*c* 0.05, C_6H_6).

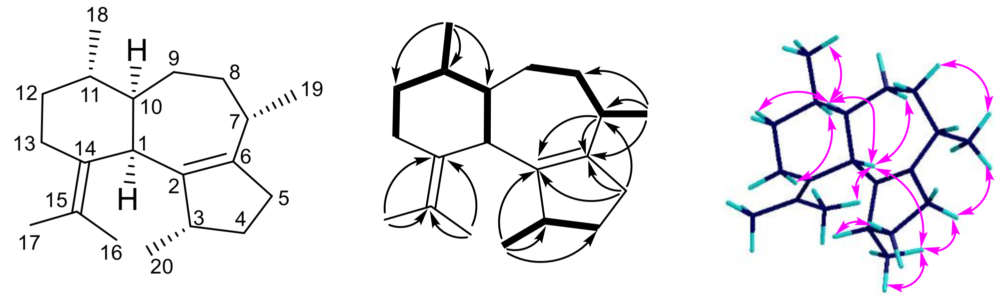

3

Figure S20. Carbon numbering and structure elucidation of **3**. Bold lines represent ^{1}H,^{1}H-COSY correlations, selected HMBC correlations are represented by single-headed arrows and key NOESY correlations are indicated by double headed arrows.

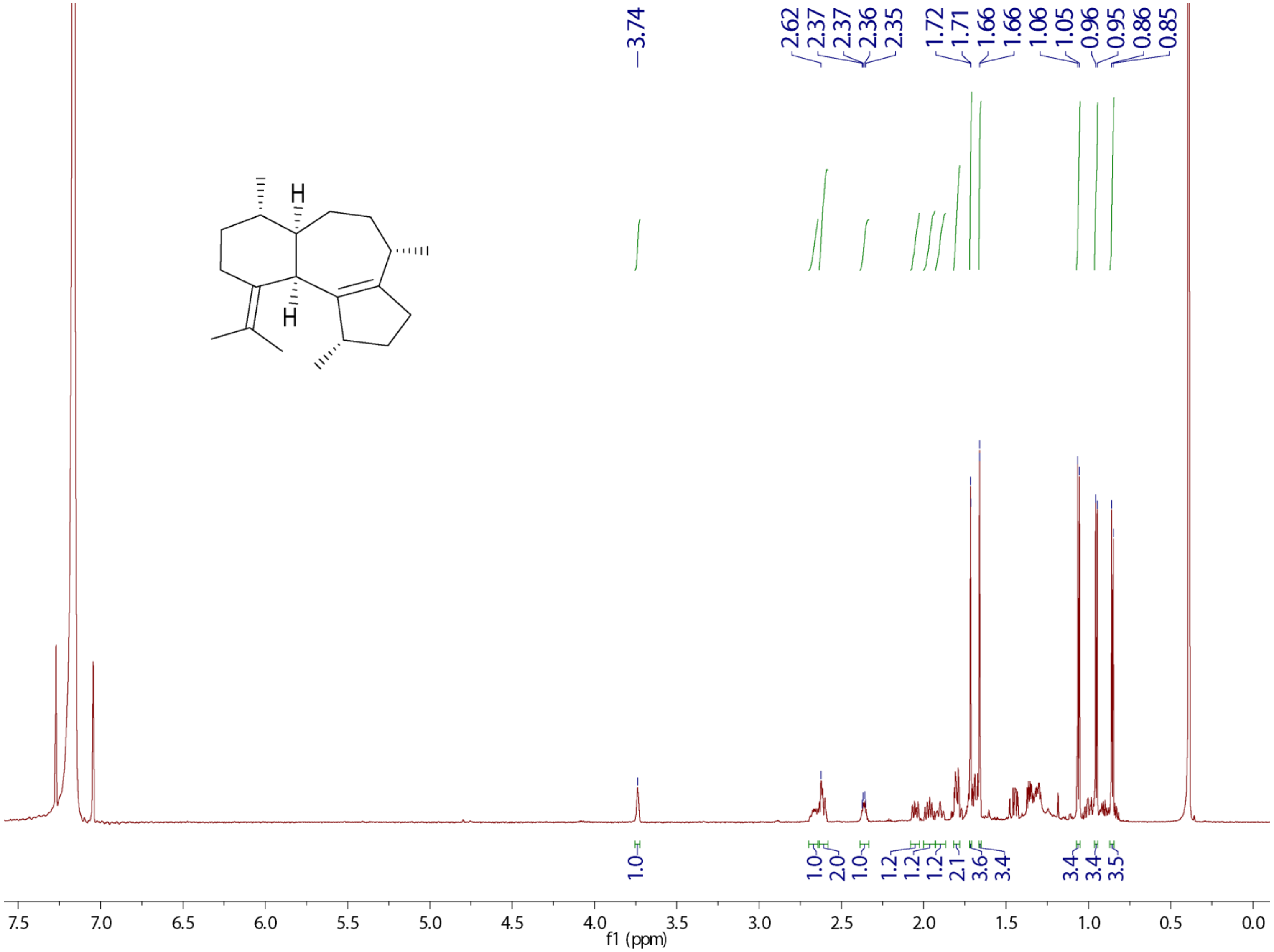

Figure S21. ^{1}H-NMR spectrum of **3** (700 MHz, C_6D_6).

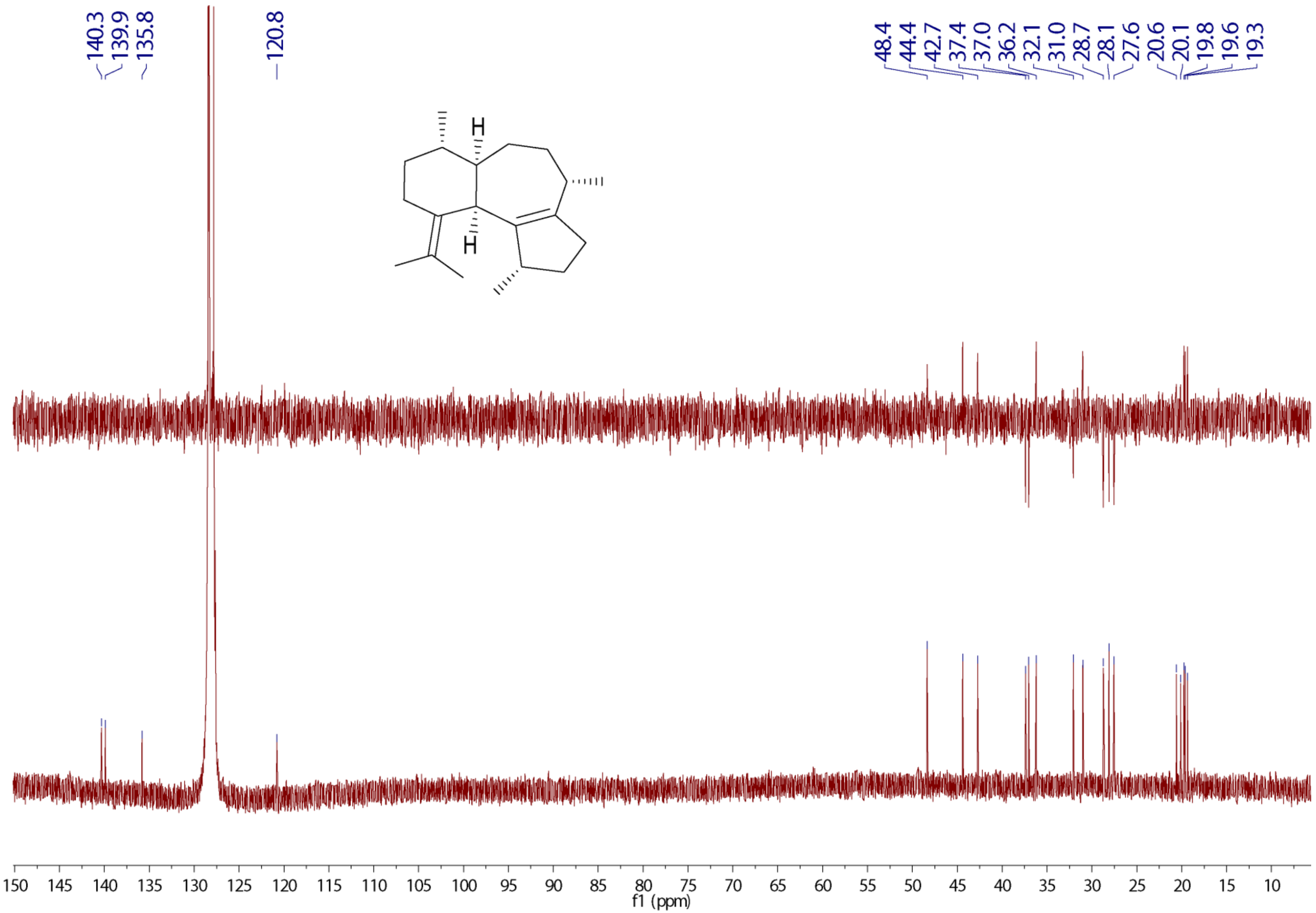

Figure S22. ^{13}C-NMR and ^{13}C-DEPT-135 spectra of **3** (175 MHz, C_6D_6).

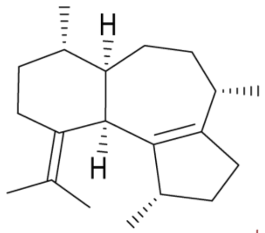

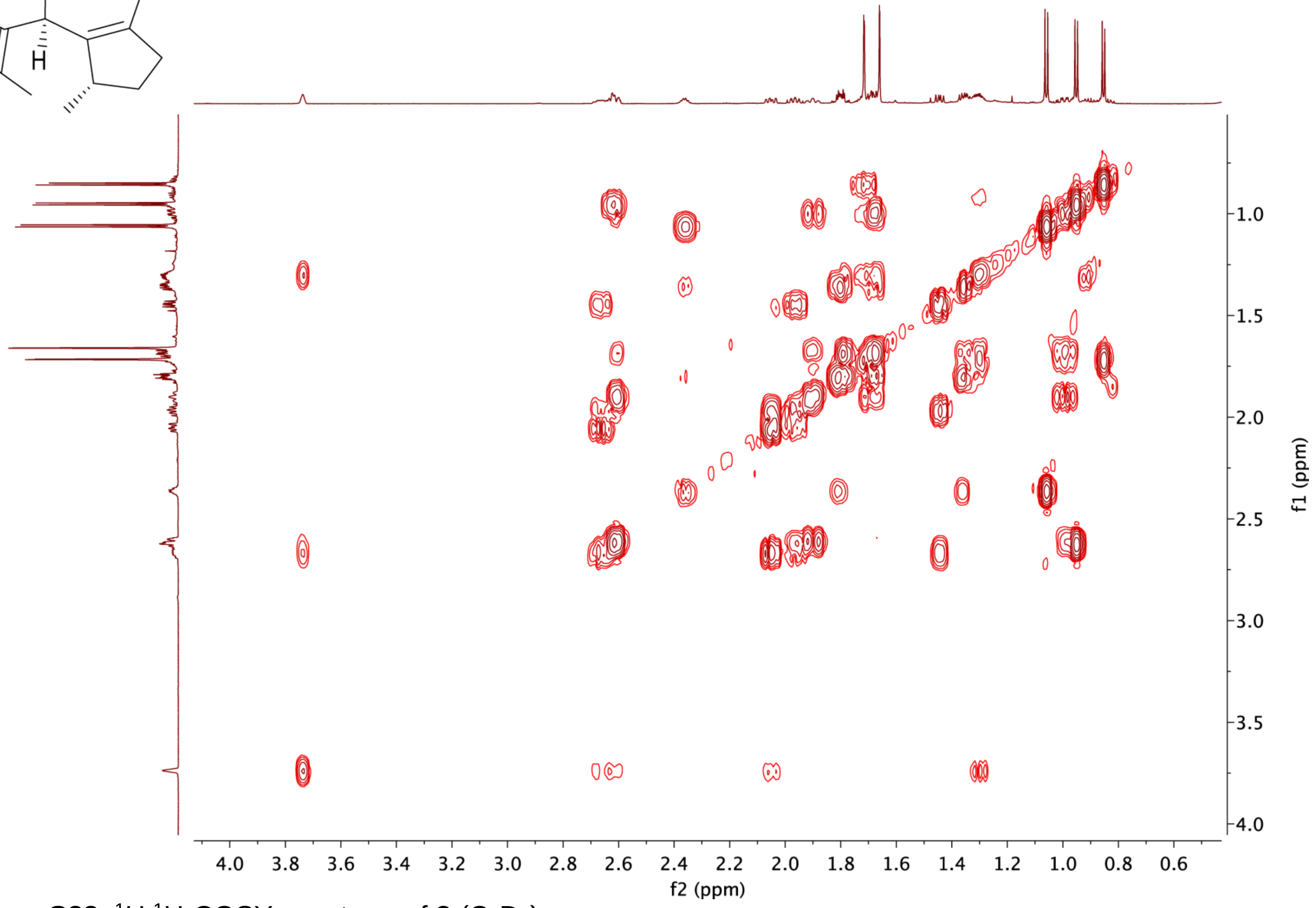

Figure S23. ^{1}H,^{1}H-COSY spectrum of **3** (C_6D_6).

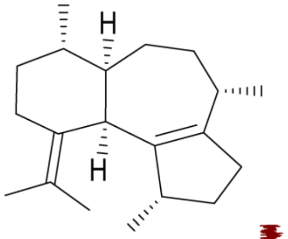

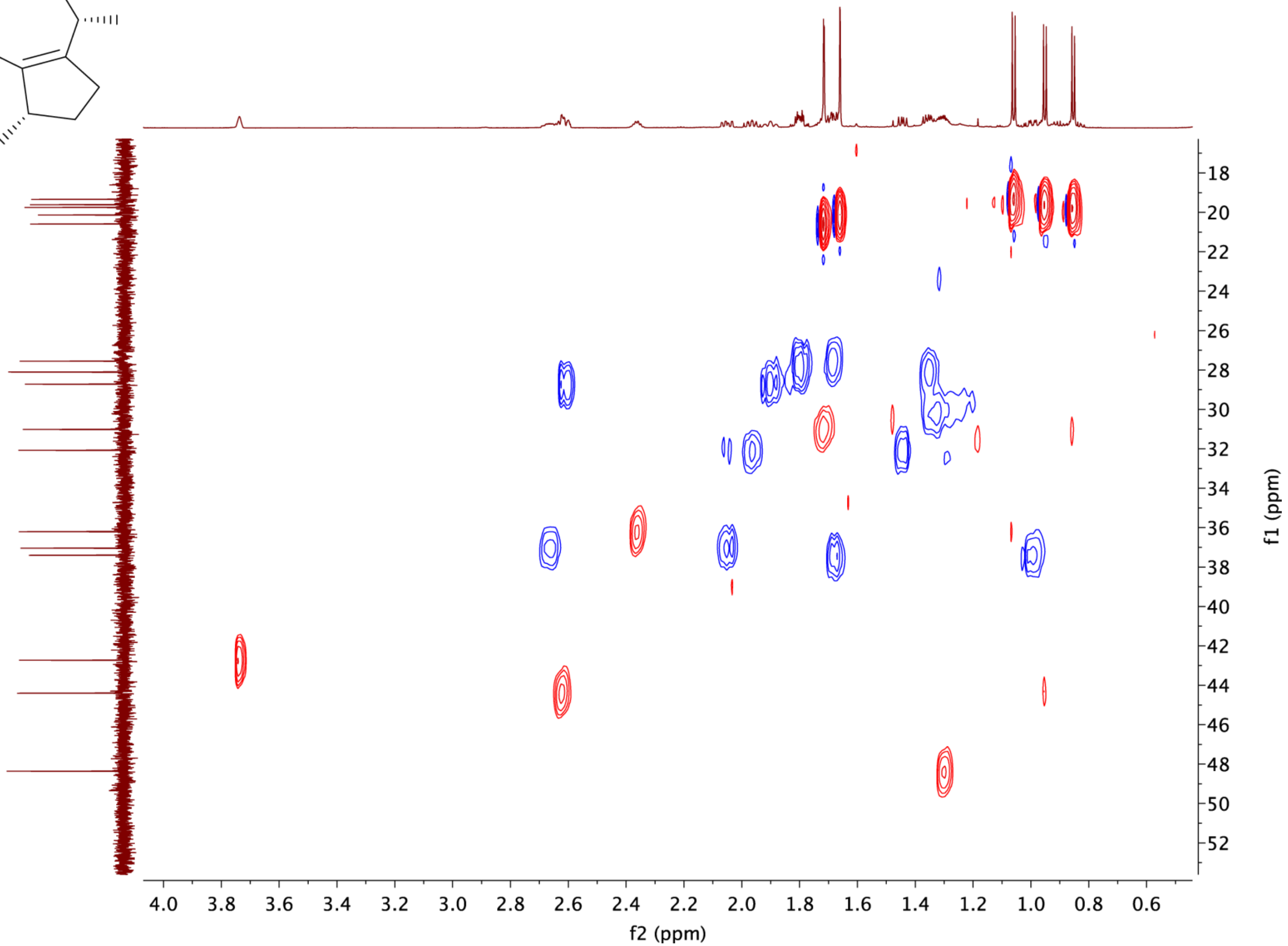

Figure S24. HSQC spectrum of **3** (C_6D_6).

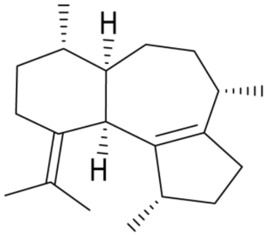

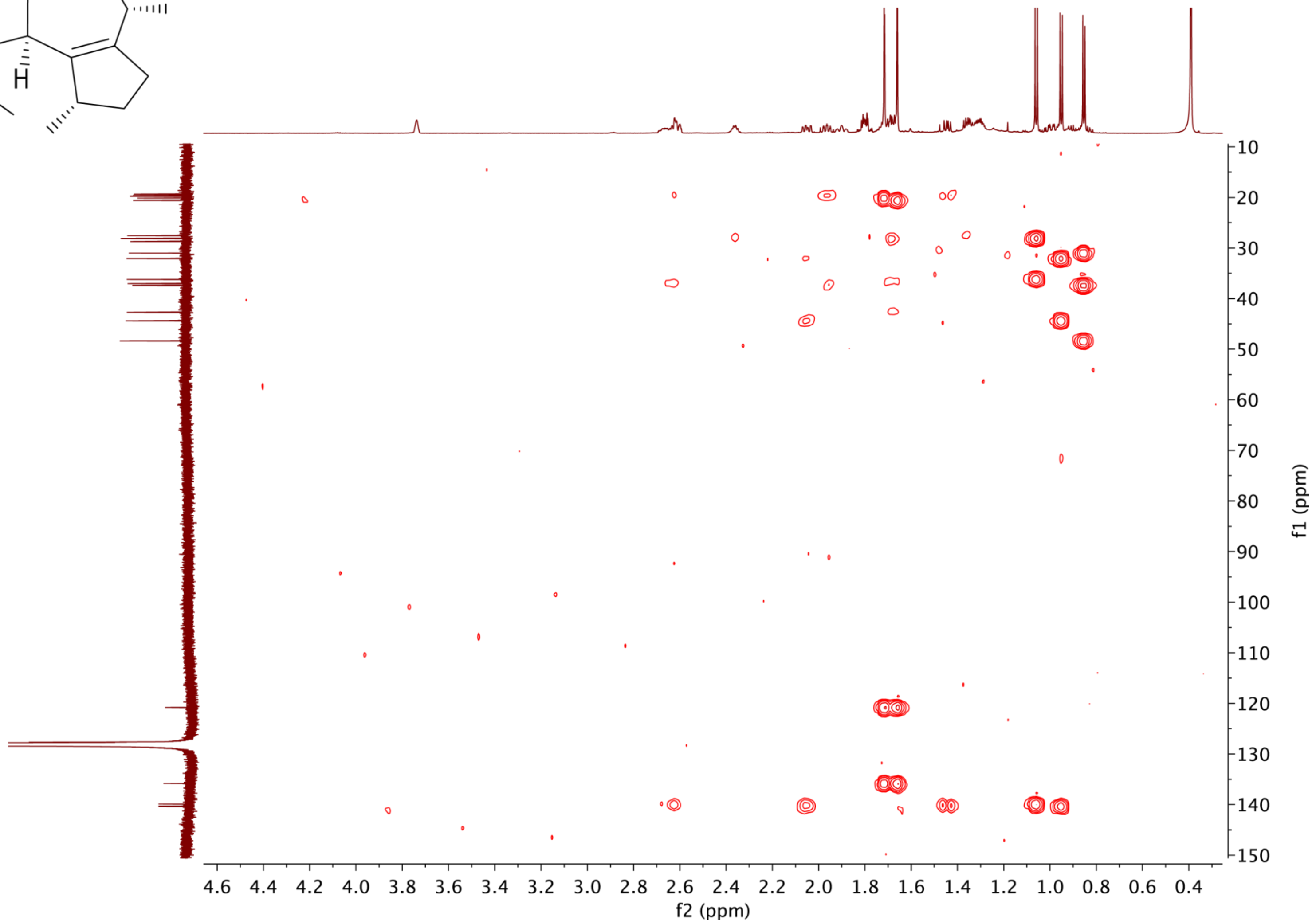

Figure S25. HMBC spectrum of **3** (C_6D_6).

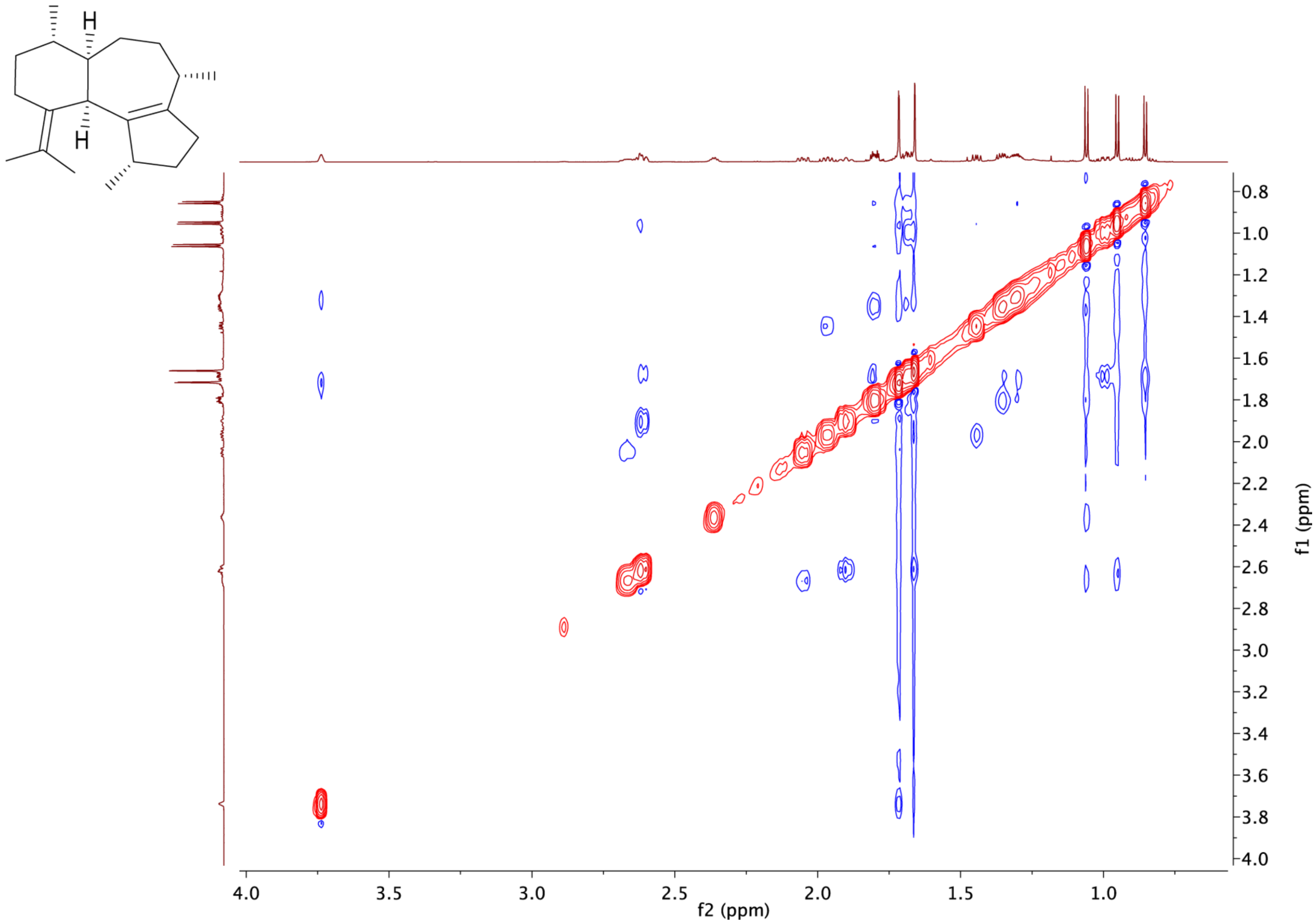

Figure S26. NOESY spectrum of **3** (C_6D_6).

```
Consensus                                          MPVIDPTRLFDLPELQLIPGDRKAPWGPDLDGVVAAFARETGLLTTETAXRYYAAQXIGT    60
WP_015792165 (Catenulispora acidiphila DSM 44928)  MPADEITRLFDLPEFRLLPGERRAAWGPELDDHVIDFACRTGLLTTPAARRYYASQRIGT    60
WP_034260409 (Actinospica robiniae DSM 44927)      MSATEITHLFDLPELQLIPGERAAPWGPQLDASLIAFVRATGLLTTETALDYYTTQHFGT    60
WP_137245160 (Herbidospora galbida NEAU-GS14)      MPVIDPTRLFDLPELQLIPGDRKASWGPDLDGVVAAFARETGLLTTETADRYYAAQRIGT    60
WP_066367595 (Herbidospora mongoliensis NBRC 10588) MPVIDPTRLFDLPELPLIPGDRKAPWGPDLDGVLAAFVRETGLLTTETAERYYAAQHIGT    60
WP_110698172 (Streptosporangium sp. caverna)       MPVIDPTRLFDLPELQLIPGDRKAPWGPDLDGVVAAFARETGLLTTETANHYYTKQNIGT    60

Consensus                                          MCAYVVPGAVSRXRREIYGKLQTWFFIYDDWAEQLGRFLLREDVAGVTDIVLTWFAESED   120
WP_015792165 (Catenulispora acidiphila DSM 44928)  MCAYVVPGALSPRRYEIYGELMTWFFIYDDWAEQLGNHLSPQQVSGVTDVVHTWFAEDER   120
WP_034260409 (Actinospica robiniae DSM 44927)      MCAYVVPGAVTQARREIYGKLMAWFFIYDDWAEQLGRHLLPEDVAGMVDDIQTWFADDED   120
WP_137245160 (Herbidospora galbida NEAU-GS14)      MCAYVIPGAVSRDRREIYGKLQTWLFIYDDWAEQLGRFLNREDVARVTDIVLTWFAESEK   120
WP_066367595 (Herbidospora mongoliensis NBRC 10588) MCAYVAPGAVSRARREIYGKLQTWFFIYDDWAEQLGRFLQREDVADVTDIVLTWFAESED   120
WP_110698172 (Streptosporangium sp. caverna)       MCAYVVPGAVSRDRRETYGKLQTWLFIYDDWAEQLGRFLLREDVTGVTDIVLTWFAESEW   120

Consensus                                          DCRRLDLAAARSMREIWAQIQQDTSPDWRHRLREELGVYLRTAVEEAELVRSGRVNPFGK   180
WP_015792165 (Catenulispora acidiphila DSM 44928)  DVVMLDAPLAQSMRGIWAKLQEDTSLEWRRRLLAETDGYLRTAAEEAILVSTGRVNSFGK   180
WP_034260409 (Actinospica robiniae DSM 44927)      DVRRFDLPAARSMRTIWAQIREDTSPQWRDRLREELGMYLRTAVEEARLVRSGRVNPLGA   180
WP_137245160 (Herbidospora galbida NEAU-GS14)      DCRRLDLAVARGLREIWGQIQQNSSPDWRHRLREELGVYLRTAVEEAELVRSGRVNPFGK   180
WP_066367595 (Herbidospora mongoliensis NBRC 10588) DCRRLDLAAARSMREIWAQIQQDSSPDWRHRLREELGVYLRTAVEEAELVRSGRVNPFGK   180
WP_110698172 (Streptosporangium sp. caverna)       ECRRLDLAAARSMREIWAQIQQDTSLDWRHRLREELGVYLRTAVEEAELVRSGRVNPFGK   180

Consensus                                          ASELRPLATAAQAVFTMSEFAYGIELPRELIRHPFLRRISRASTIAIAYANDIIGLKADL   240
WP_015792165 (Catenulispora acidiphila DSM 44928)  ASELRPTATAAAPVFMMAEHSYGIEIPMDVVRHPLVRKAMRAAAAAIAYGNDIIGLKSDL   240
WP_034260409 (Actinospica robiniae DSM 44927)      ASELRPLASAARPVYTMAEYAYGIELTPETVRHPLLRKADSVGTAAIAYANDIIGLKADL   240
WP_137245160 (Herbidospora galbida NEAU-GS14)      ASELRPLATAAQAVFTQSEFAYGIELPRELIRHPFLRRISRASTIAIAYANDIIGLKADL   240
WP_066367595 (Herbidospora mongoliensis NBRC 10588) ASELRPLATAAQAVFTLSEFAYGIELPRELIRHPFLRRISRATTIAIAYANDIIGLKADL   240
WP_110698172 (Streptosporangium sp. caverna)       ASELRPLATAAQAVFTMSEFAYGIELPRELIRHPFLRRISRASTIAIAYANDIIGLKADL   240

Consensus                                          LRGIRDNLVLSLQEEYGGDLQTNVERAAKXFQRVAGEFXDLQSQFRSGGGLCDHEIAGRP   300
WP_015792165 (Catenulispora acidiphila DSM 44928)  LRGIRDNLVLSLQQEYGGDLQLNVERAAEHYQRAAGTLTALRE---------DPEAGGRE   291
WP_034260409 (Actinospica robiniae DSM 44927)      LRGIRDNLVLSLQHEHGGTLQANVERAAAKFHQKAAEFLDVQEQFRSGSGLCDATIAGRA   300
WP_137245160 (Herbidospora galbida NEAU-GS14)      LRGIRDNLVLSLQEEYGGDLQTNVERAAQNFQRVAGEFADLQSQFRSGGGLCDHEIAGRP   300
WP_066367595 (Herbidospora mongoliensis NBRC 10588) LRGIRDNLVLSLQEEYGGDLQTNVERAAKGFQRVAGEFTDLQSQFRSGGGLCDHEIAGRP   300
WP_110698172 (Streptosporangium sp. caverna)       LRGIRDNLVLSLQEEYGGDLQTNVERAAKDFHRVAGEFADLQSQFRSGGGLCDHEIAGRP   300

Consensus                                          DVEVYIQILEDWLYEGIKWQLLDTNRYQTTVRLTQXEHPNQLLSLA--EALPPE         352
WP_015792165 (Catenulispora acidiphila DSM 44928)  DVAVFFQILEDWVYEGVKWQLRDTDRYSSTVRLTQEENPNQLLALAASSALADC         345
WP_034260409 (Actinospica robiniae DSM 44927)      DVATYLQILEDWLFEGVKWQLRETDRYDTTVRLTDRENPNQLLLLSGAAA----         350
WP_137245160 (Herbidospora galbida NEAU-GS14)      DVEVYIQILEDWLYEGIKWQLLDTNRYQTTVRLTQQEHPNQLLSLA--EALPPE         352
WP_066367595 (Herbidospora mongoliensis NBRC 10588) DVEVYIQILEDWIYEGIKWQLLDTNRYQTTVRLTQQEHPNQLLSLT--EALPPE         352
WP_110698172 (Streptosporangium sp. caverna)       DVEVYIQILEDWMYEGIKWQLLDTNRYETTVRLTQREHPNQLLSLA--EALQPE         352
```

Figure S27. Amino acid sequence alignment of CaCS with four closely related enzymes identified by a BLAST search against sequenced bacteria. All four homologs have a pairwise identity with CaCS of >61% suggesting they may have the same function. The next closest hit returned by the BLAST search (WP_168873020 from *Chitinophaga varians* Ae27) had a pairwise identity of only 24.5% and likely has a different function. Amino acid residues that are conserved in all five sequences of the alignment are highlighted in green, positions in which functionally similar amino acid residues are conserved are highlighted in yellow.

Incubation experiments with isotopically labelled substrates

Isotopic labelling experiments were performed with ca. 1 mg labelled GGPP (or its precursors) in substrate buffer (1 mL), incubation buffer (5 mL), enzyme elution fractions (1 mL each, protein concentrations of ca. 1 mg/mL) and binding buffer (to 10 mL total volume) with the substrates and enzyme preparations as listed in Table S5. After incubation with shaking at 28 °C for 4 h, the products were extracted with C_6D_6 (700 μL and 300 μL), the extracts were dried with $MgSO_4$ and analysed by NMR and/or GC/MS.

Table S5. Labeling experiments with CaCS for compounds **1-3**.

entry	substrate	enzyme(s)	results shown in
1	FPP + (1-^{13}C)IPP[14]	CaCS, GGPPS[14]	Figure S28
2	(2-^{13}C)GGPP[14]	CaCS	Figure S28
3	(3-^{13}C)IPP[14] + FPP	CaCS, GGPPS[14]	Figure S28
4	(4-^{13}C)IPP[14] + FPP	CaCS, GGPPS[14]	Figure S28
5	(1-^{13}C)FPP[20] + IPP	CaCS, GGPPS[14]	Figure S28
6	(2-^{13}C)FPP[20] + IPP	CaCS, GGPPS[14]	Figure S28
7	(3-^{13}C)FPP[20] + IPP	CaCS, GGPPS[14]	Figure S28
8	(4-^{13}C)FPP[20] + IPP	CaCS, GGPPS[14]	Figure S28
9	(5-^{13}C)FPP[20] + IPP	CaCS, GGPPS[14]	Figure S28
10	(6-^{13}C)FPP[20] + IPP	CaCS, GGPPS[14]	Figure S28
11	(7-^{13}C)FPP[20] + IPP	CaCS, GGPPS[14]	Figure S28
12	(8-^{13}C)FPP[20] + IPP	CaCS, GGPPS[14]	Figure S28
13	(9-^{13}C)FPP[20] + IPP	CaCS, GGPPS[14]	Figure S28
14	(10-^{13}C)FPP[20] + IPP	CaCS, GGPPS[14]	Figure S28
15	(11-^{13}C)FPP[20] + IPP	CaCS, GGPPS[14]	Figure S28
16	(12-^{13}C)FPP[20] + IPP	CaCS, GGPPS[14]	Figure S28
17	(9-^{13}C)GPP[21] + IPP	CaCS, GGPPS[14]	Figure S28
18	(10-^{13}C)GPP[22] + IPP	CaCS, GGPPS[14]	Figure S28
19	(15-^{13}C)FPP[20] + IPP	CaCS, GGPPS[14]	Figure S28
20	(20-^{13}C)GGPP[14]	CaCS	Figure S28
21	(7-^{13}C)FPP[20] + (*R*)-(1-^{2}H)IPP[23]	CaCS, GGPPS[14]	Figure S29
22	(7-^{13}C)FPP[20] + (*S*)-(1-^{2}H)IPP[23]	CaCS, GGPPS[14]	Figure S29
23	(3-^{13}C,2-^{2}H)GPP[24] + IPP	CaCS, GGPPS[14]	Figure S29
24	(2-^{2}H)DMAPP[25] + IPP	CaCS, FPPS,[26] GGPPS[14]	Figure S30
25	(3-^{13}C)IPP[14] + FPP + D_2O	CaCS, GGPPS[14]	Figure S31 and S32
26	IPP + (3-^{13}C,2-^{2}H)FPP[27]	CaCS, GGPPS[14]	Figure S33
27	(2-^{2}H)DMAPP[25] + (2-^{13}C)FPP[20]	CaCS, IDI,[24] GGPPS[14]	Figure S34 and S35
28	(2-^{2}H)DMAPP[25] + (3-^{13}C)FPP[20]	CaCS, IDI,[24] GGPPS[14]	Figure S36
29	DMAPP + (*E*)-(4-^{13}C,4-^{2}H)IPP[28]	CaCS, FPPS,[26] GGPPS[14]	Figure S37, S39 and S41
30	DMAPP + (*Z*)-(4-^{13}C,4-^{2}H)IPP[28]	CaCS, FPPS,[26] GGPPS[14]	Figure S37, S39 and S41
31	(*R*)-(1-^{13}C,1-^{2}H)IPP[6]	CaCS, IDI,[24] FPPS,[26] GGPPS[14]	Figure S38, S40 and S42
32	(*S*)-(1-^{13}C,1-^{2}H)IPP[6]	CaCS, IDI,[24] FPPS,[26] GGPPS[14]	Figure S38, S40 and S42

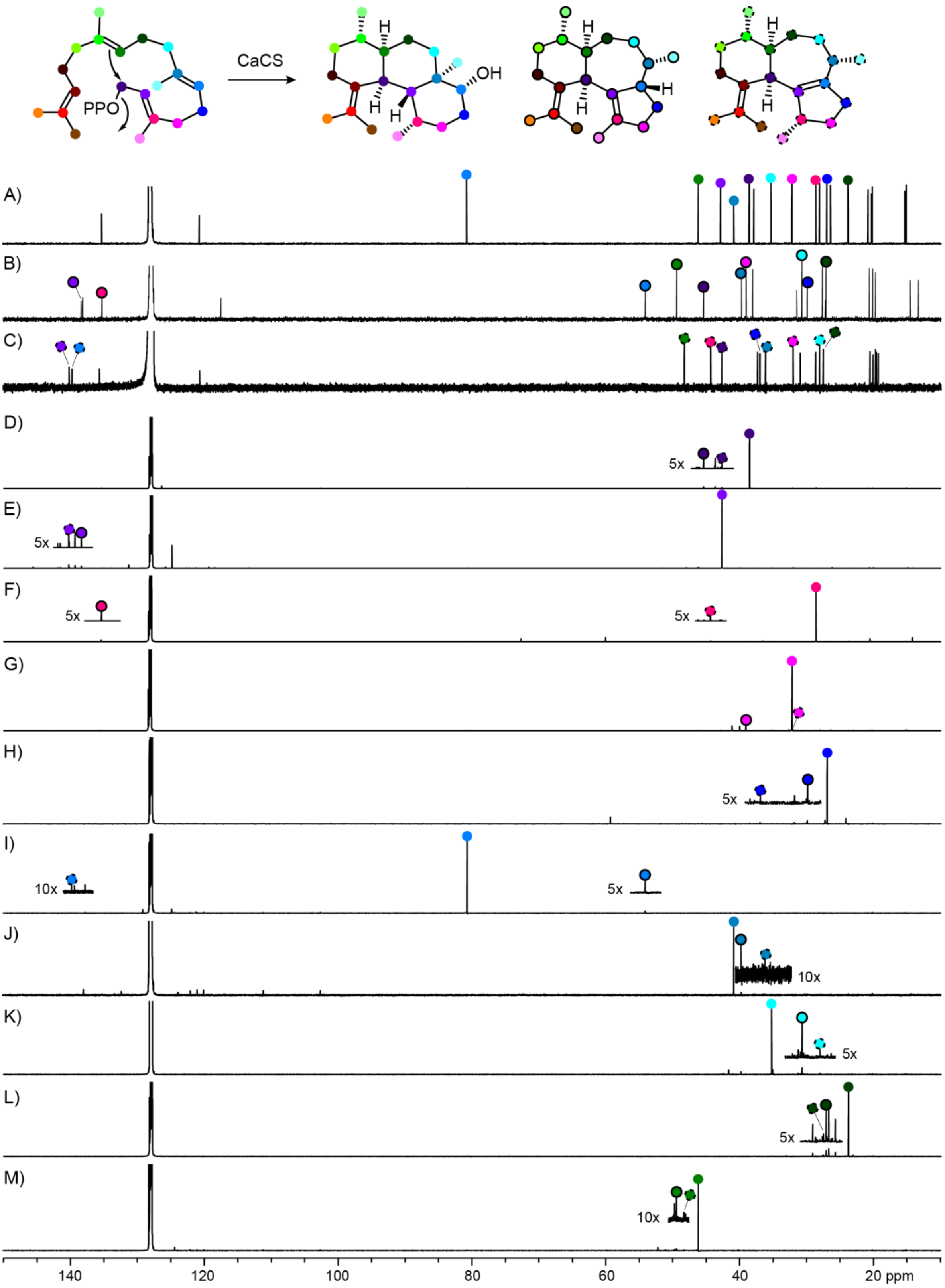

Figure S28. ^{13}C-NMR spectra of A) unlabelled **1**, B) unlabelled **2**, C) unlabelled **3** and D) – M) ^{13}C-labelled **1** – **3** obtained from the 10 isotopomers of ($^{13}C_1$)GGPP labelled at carbons C1 – C10 with CaCS. Colored dots correlate the observed ^{13}C signals in the labelling experiments to the individual carbon signals of **1**, **2** and **3**.

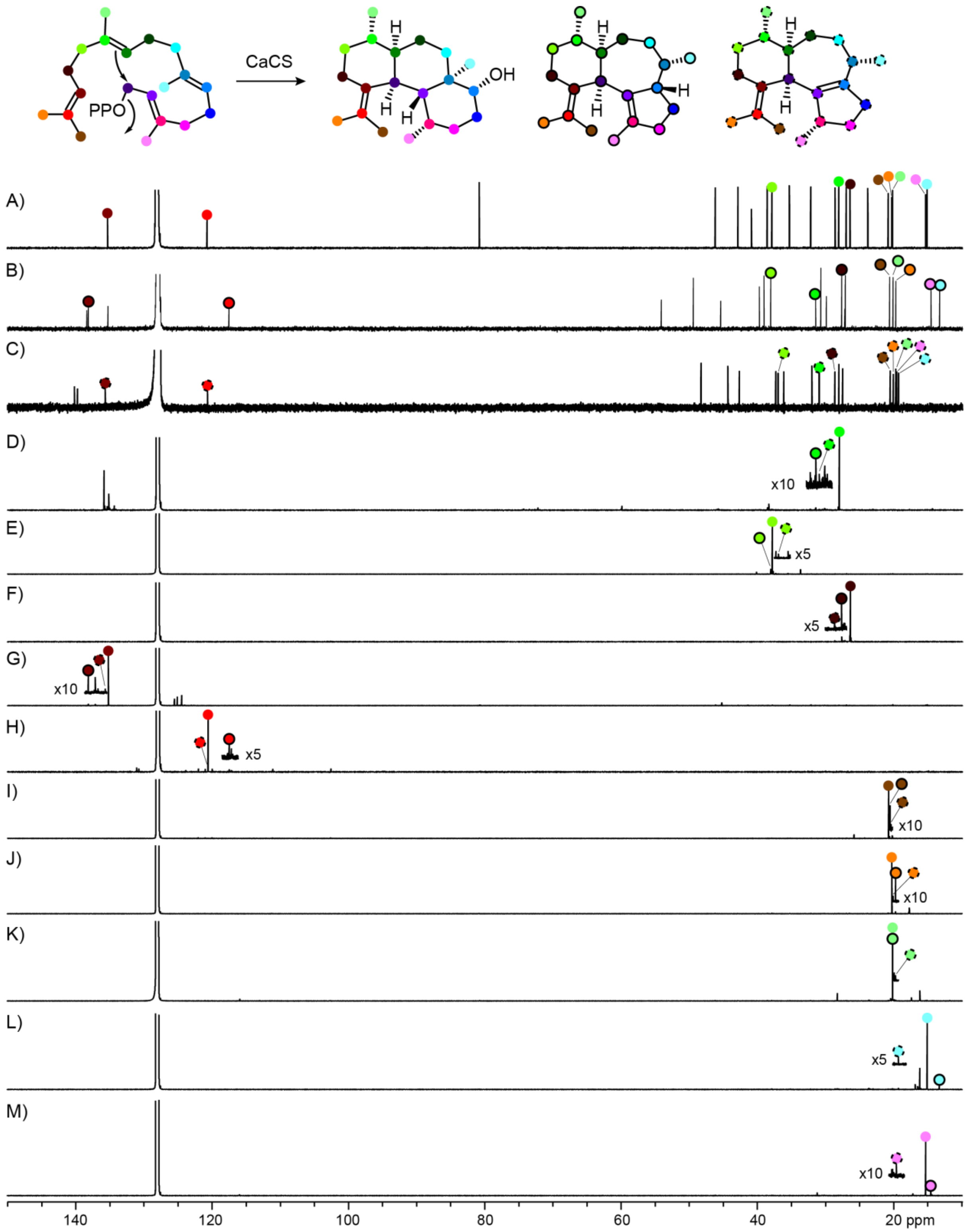

Figure S28. ^{13}C-NMR spectra of A) unlabelled **1**, B) unlabelled **2**, C) unlabelled **3** and D) – M) ^{13}C-labelled **1** – **3** obtained from the 10 isotopomers of ($^{13}C_1$)GGPP labelled at carbons C11 – C20 with CaCS. Colored dots correlate the observed ^{13}C signals in the labelling experiments to the individual carbon signals of **1**, **2** and **3**.

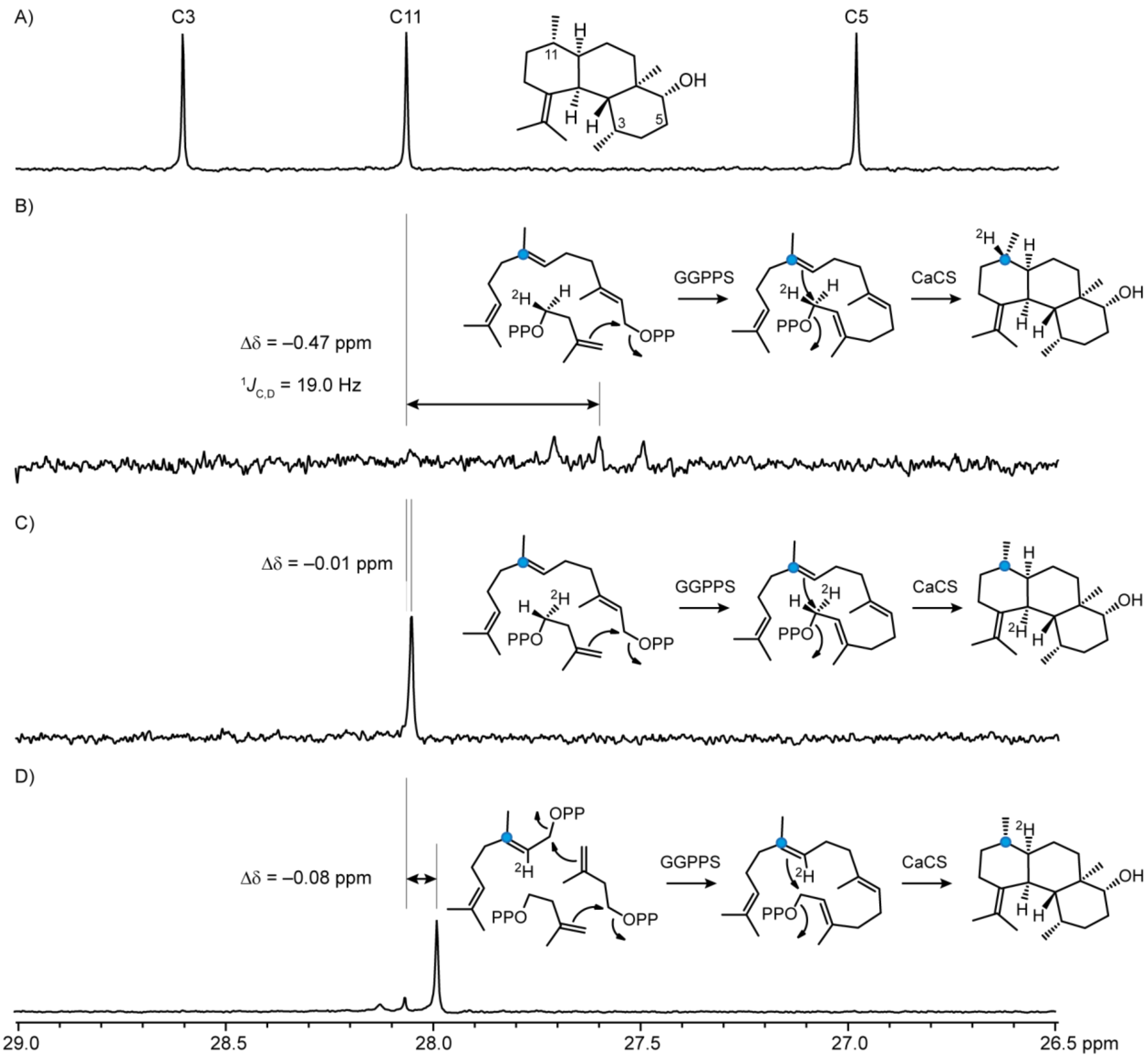

Figure S29. The 1,3-hydride shift from **A** to **B** in the biosynthesis of **1** (Scheme 1 of main text). A) Partial ^{13}C-NMR spectrum of **1**. B) ^{13}C-NMR spectrum of (11-^{13}C,11-^{2}H)-**1** obtained from (*S*)-(1-^{2}H)IPP and (7-^{13}C)FPP with GGPPS and CaCS. The upfield shifted triplet for C11 indicates a deuterium directly bound to ^{13}C. C) ^{13}C-NMR spectrum of the product (11-^{13}C,1-^{2}H)-**1** obtained from (*R*)-(1-^{2}H)IPP and (7-^{13}C)FPP with GGPPS and CaCS. The minor upfield shift for C11 is a result of deuterium bound to C1. D) ^{13}C-NMR spectrum of the product (11-^{13}C,10-^{2}H)-**1** obtained from (3-^{13}C,2-^{2}H)GPP and IPP with GGPPS and CaCS. The upfield shift for C11 is a result of deuterium bound to the neighbouring carbon C10. Blue dots represent ^{13}C-labelled carbons.

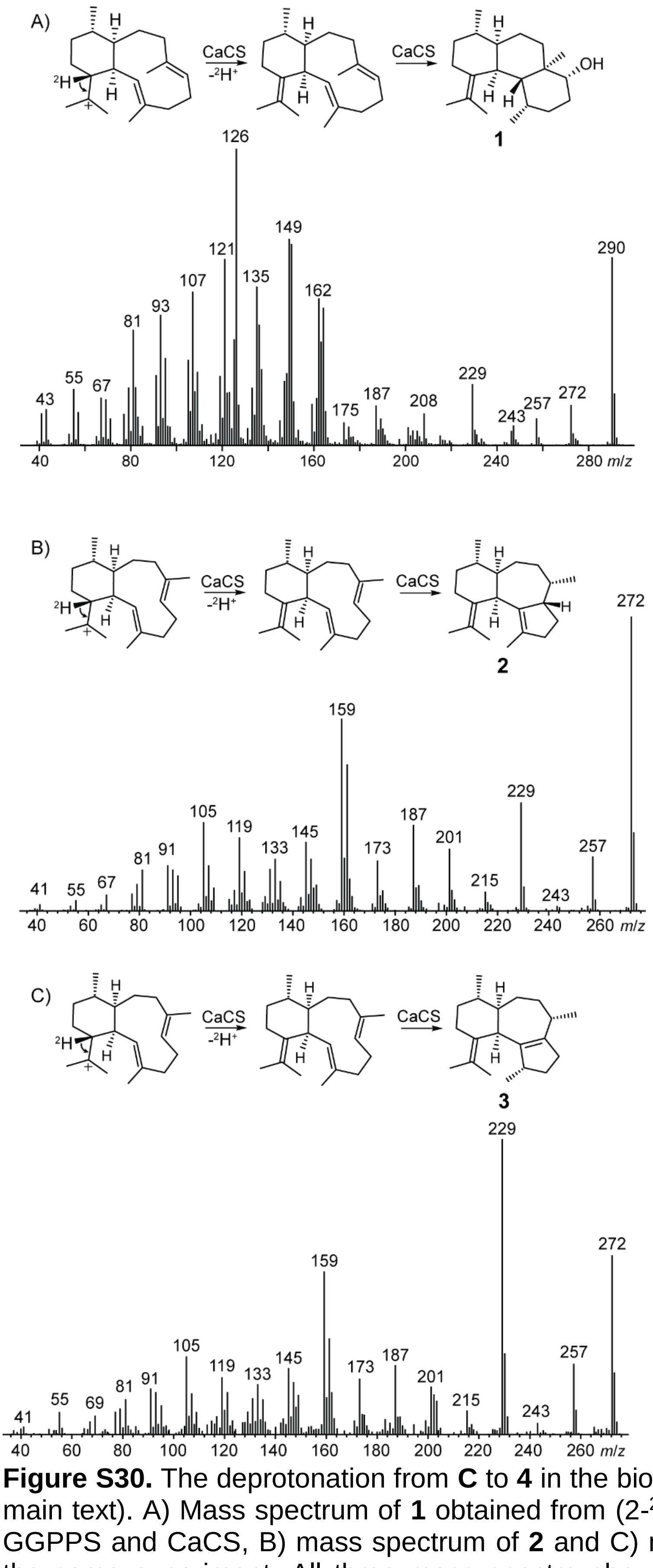

Figure S30. The deprotonation from **C** to **4** in the biosynthesis of **1** – **3** (Scheme 1 of main text). A) Mass spectrum of **1** obtained from (2-^{2}H)DMAPP and IPP with FPPS, GGPPS and CaCS, B) mass spectrum of **2** and C) mass spectrum of **3** obtained in the same experiment. All three mass spectra show a molecular ion at *m*/*z* = 272 indicating no incorporation of deuterium.

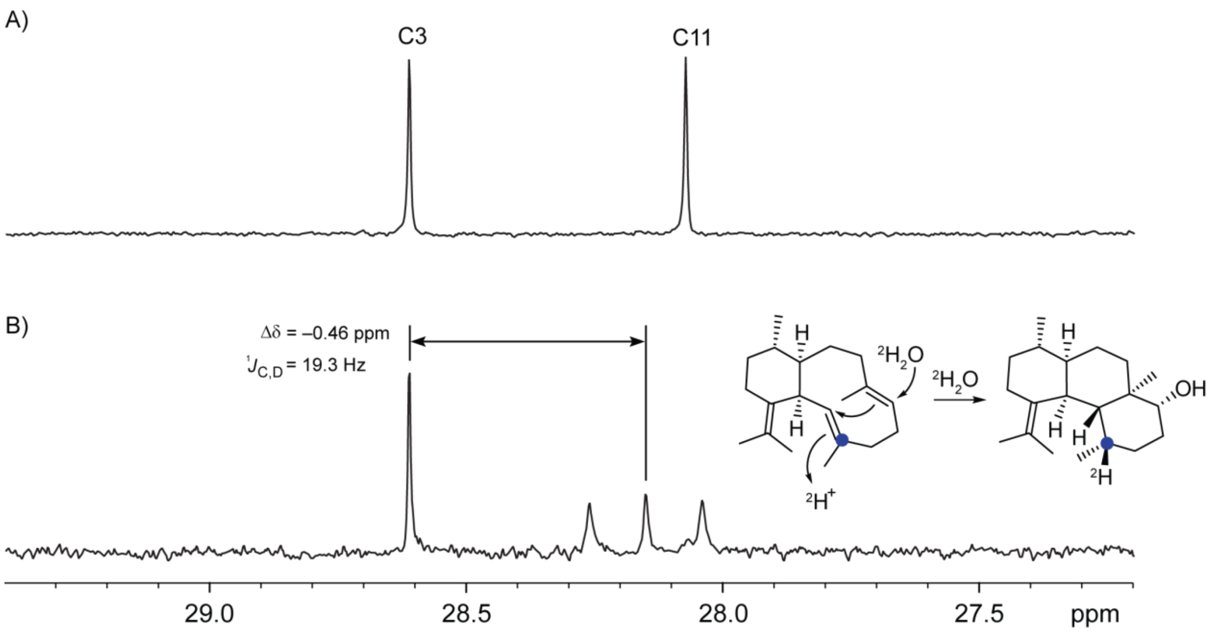

Figure S31. Reprotonation at C3 of **4** in the biosynthesis of **1** (Scheme 1 of main text). A) Partial ^{13}C-NMR spectrum of **1**. B) ^{13}C-NMR spectrum of the product (3-^{13}C,3-^{2}H)-**1** obtained from (3-^{13}C)IPP and FPP with GGPPS and CaCS in D_2O. The upfield shifted triplet for C3 indicates a deuterium directly bound to ^{13}C. The additionally observed singlet for C3 is a result of incomplete deuteration of the substrate by residual water. Deuterium from the hydroxyl group was lost during work-up. Blue dots represent ^{13}C-labelled carbons.

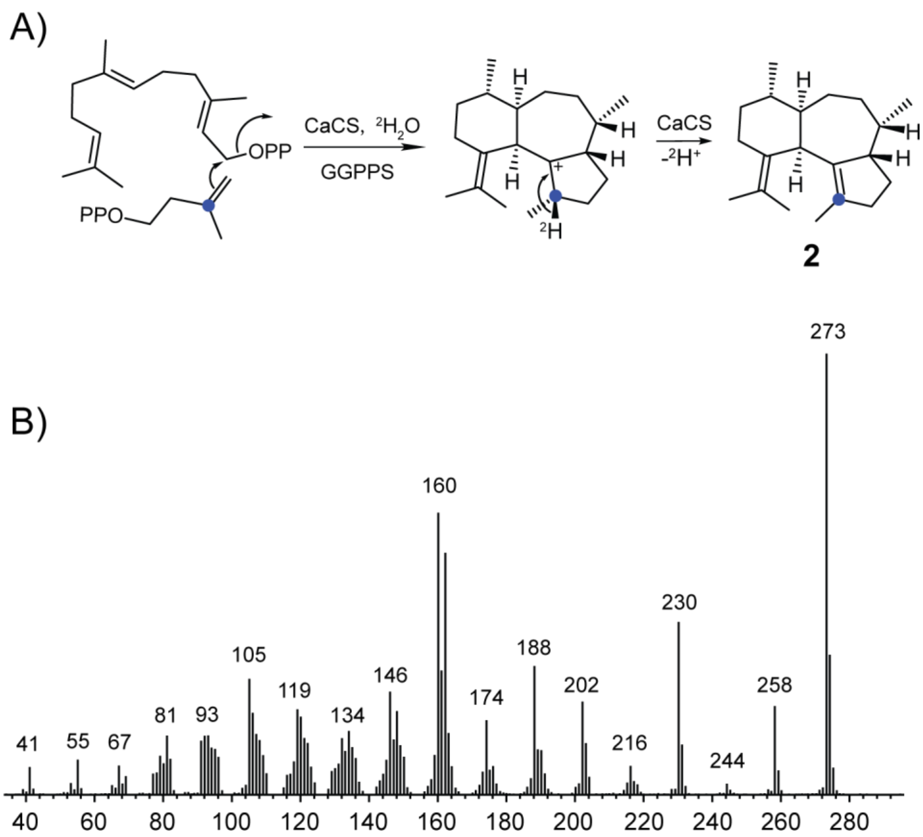

Figure S32. Deprotonation from C3 of **F** in the biosynthesis of **2** (Scheme 1 of main text). A) Enzymatic conversion of (3-^{13}C)IPP and FPP with GGPPS and CaCS in D_2O. B) Mass spectrum of the product (3-^{13}C)-**2** obtained from (3-^{13}C)IPP and FPP with GGPPS and CaCS in D_2O, showing incorporation of ^{13}C and loss of deuterium. Blue dots represent ^{13}C-labelled carbons.

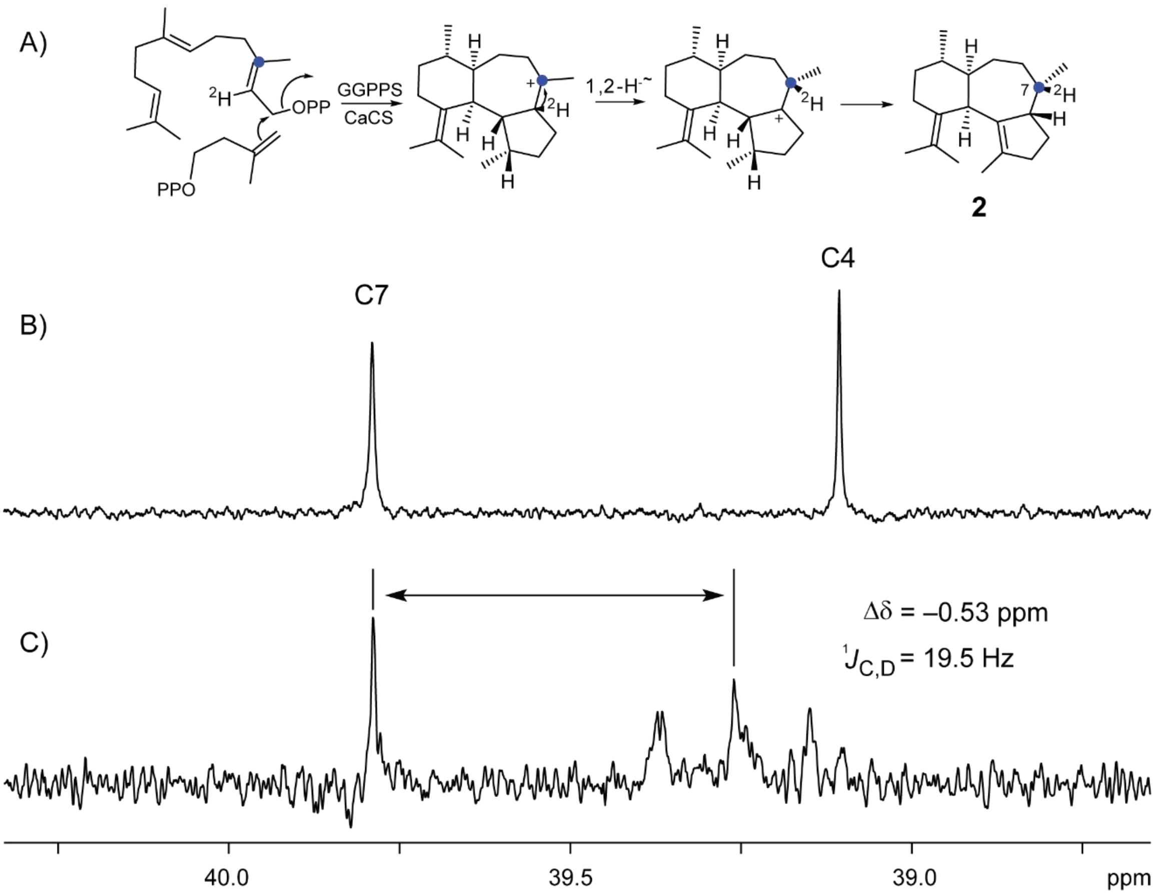

Figure S33. The 1,2-hydride shift from **D** to **E** in the biosynthesis of **2** (Scheme 1 of main text). A) Enzymatic conversion of (2-^{2}H,3-^{13}C)FPP and IPP with GGPPS and CaCS. B) Partial ^{13}C-NMR spectrum of **2**. C) ^{13}C-NMR spectrum of the product (7-^{13}C,7-^{2}H)-**2** obtained from IPP and (2-^{2}H,3-^{13}C)FPP with GGPPS and CaCS. Blue dots represent ^{13}C-labelled carbons.

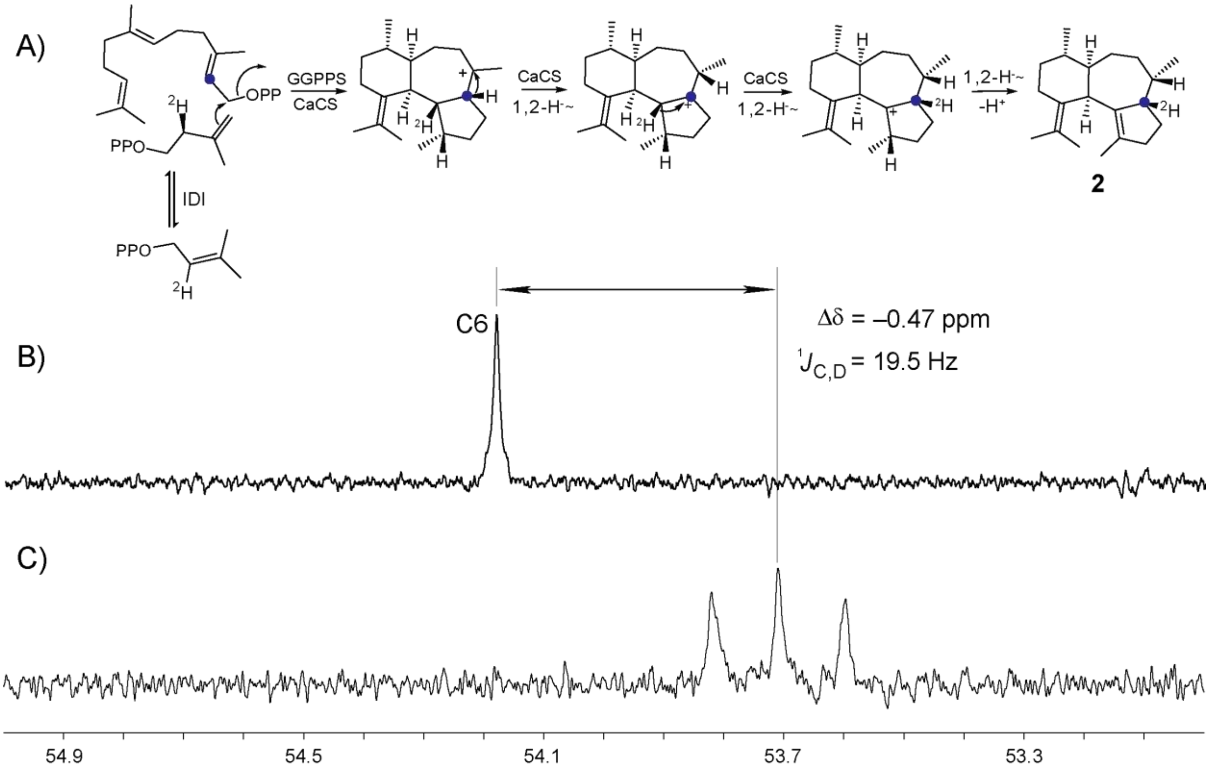

Figure S34. The 1,2-hydride migration from **E** to **F** in the biosynthesis of **2** (Scheme 1 of main text). A) Enzymatic conversion of (2-^{2}H)DMAPP and (2-^{13}C)FPP with IDI, GGPPS and CaCS. B) Partial ^{13}C-NMR spectrum of **2**. C) ^{13}C-NMR spectrum of the product (6-^{13}C,6-^{2}H)-**2** obtained from (2-^{2}H)DMAPP and (2-^{13}C)FPP with IDI, GGPPS and CaCS. The upfield shifted triplet for C6 indicates a direct ^{13}C-^{2}H bond. Blue dots represent ^{13}C-labelled carbons.

A)

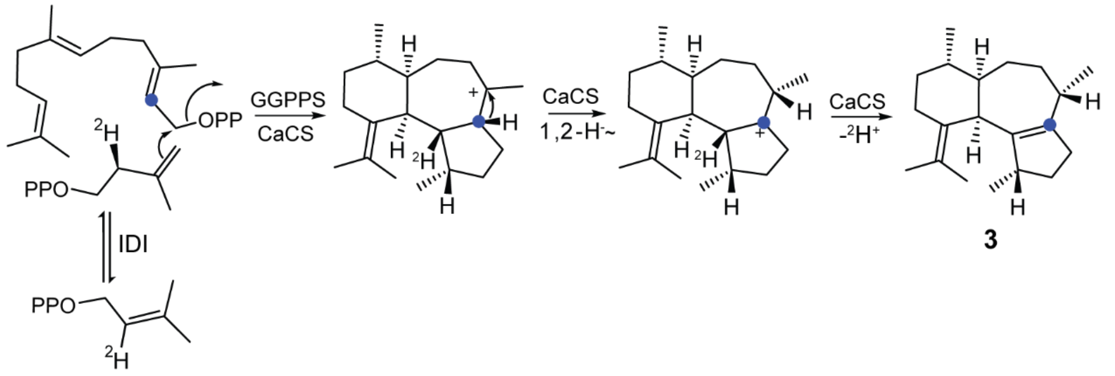

B)

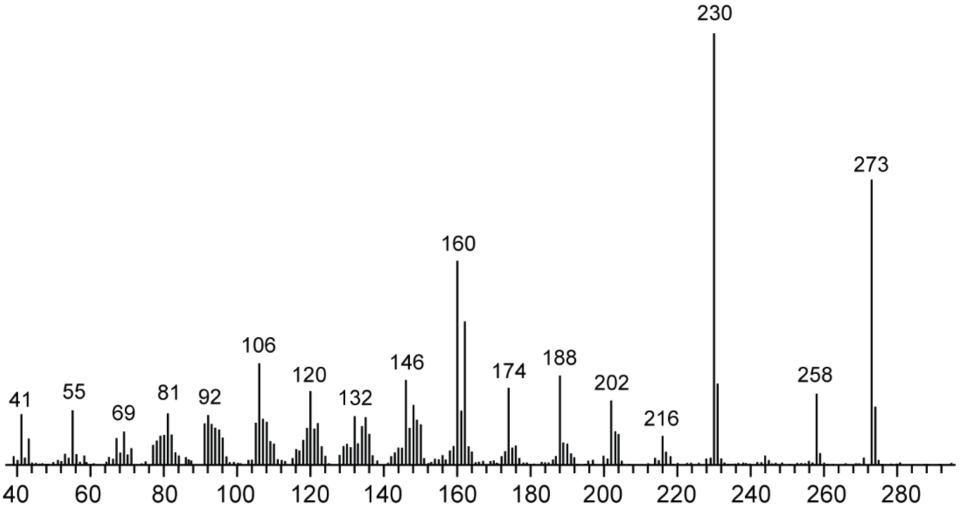

Figure S35. The deprotonation from **E** to **3**. (Scheme 1 of main text). A) Enzymatic conversion of (2-^{13}C)FPP and (2-^{2}H)DMAPP with IDI, GGPPS and CaCS. B) EI mass spectrum of labelled **3** from (2-^{2}H)DMAPP and (2-^{13}C)FPP with IDI, GGPPS and CaCS. The molecular ion at *m*/*z* = 273 indicates incorporation of ^{13}C and loss of deuterium. Blue dots represent ^{13}C-labelled carbons.

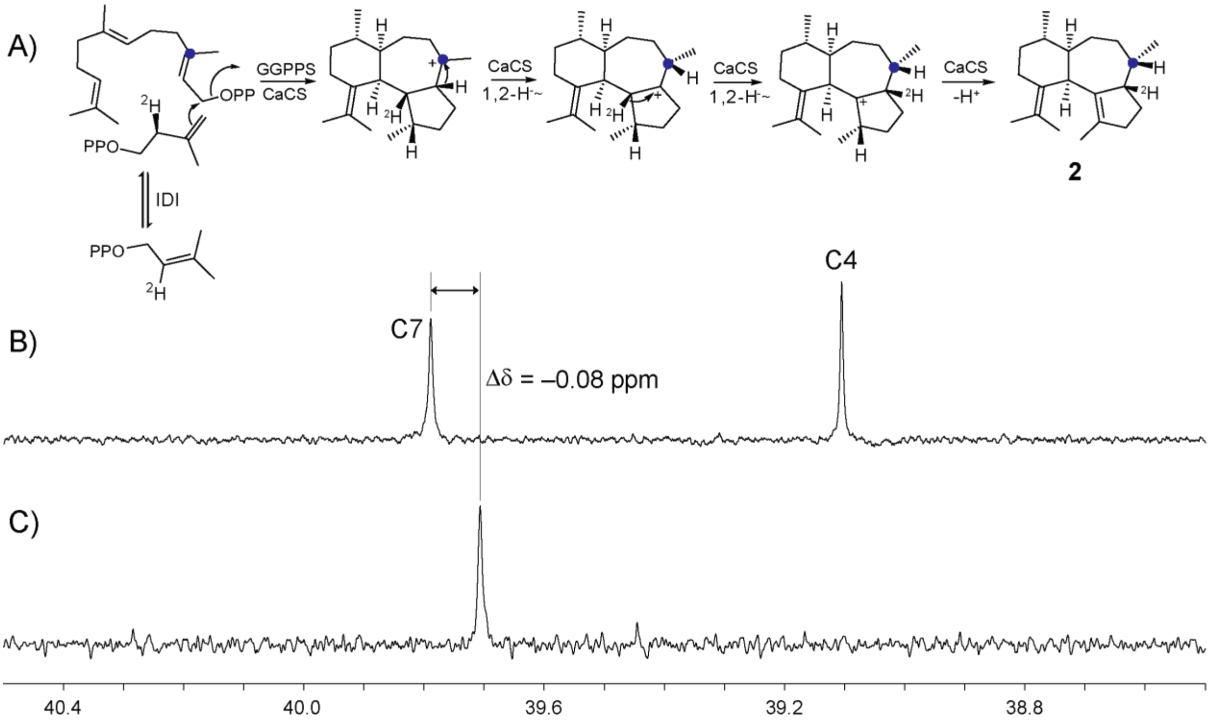

Figure S36. A) Enzymatic conversion of (2-^{2}H)DMAPP and (3-^{13}C)FPP with IDI, GGPPS and CaCS. (Scheme 1 of main text). B) Partial ^{13}C-NMR spectrum of **2**. C) ^{13}C-NMR spectrum of the product (7-^{13}C,6-^{2}H)-**2** obtained from (2-^{2}H)DMAPP and (3-^{13}C)FPP with IDI, GGPPS and CaCS. The upfield shift for C7 is a result of deuterium bound to the neighbouring carbon C6. Blue dots represent ^{13}C-labelled carbons.

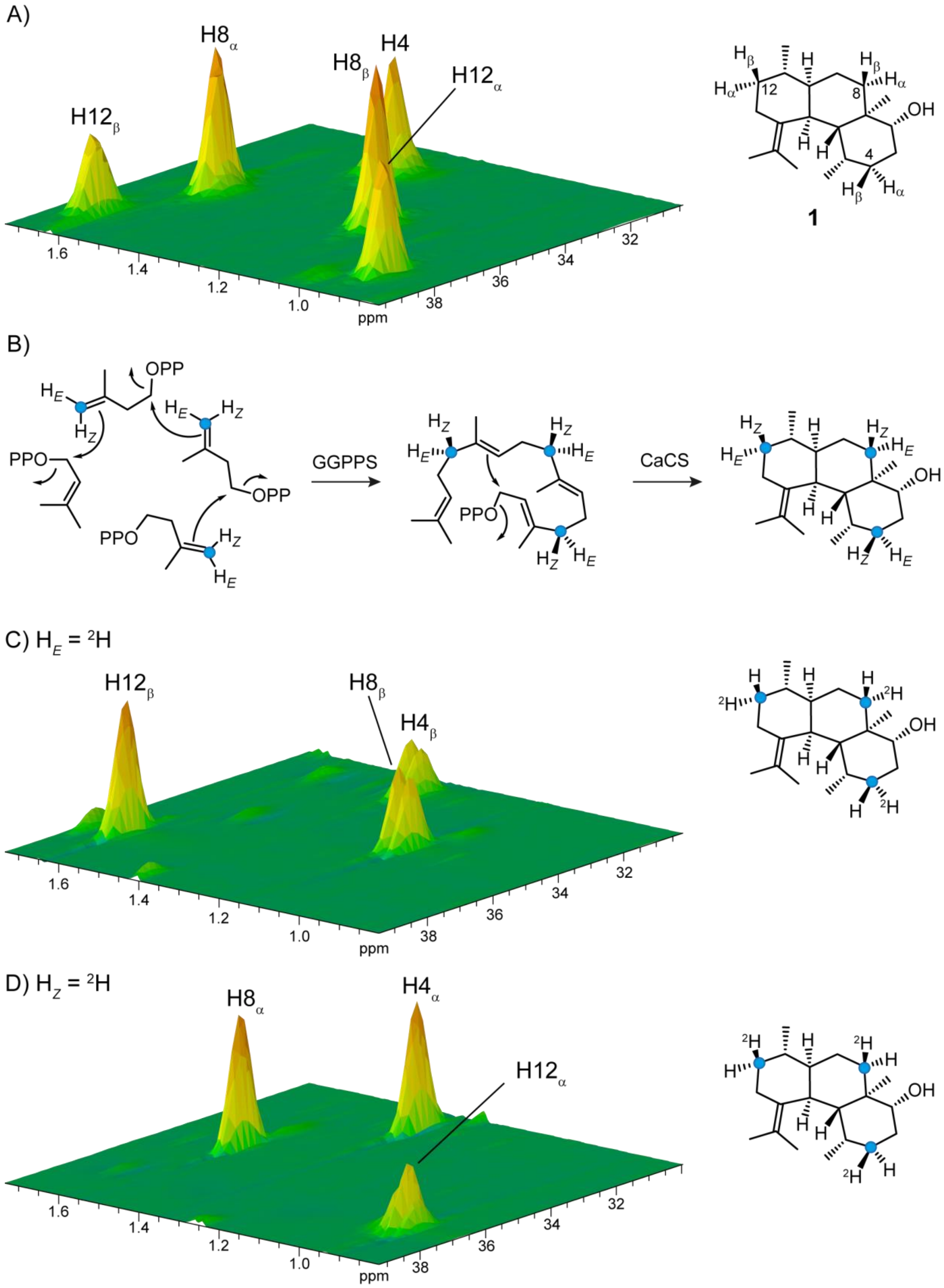

Figure S37. Determination of the absolute configuration of **1** using the substrates DMAPP and (*E*)- or (*Z*)-(4-^{13}C,4-^{2}H)IPP. A) HSQC spectrum of unlabelled **1**. B) Formation of labelled **1** in the enzyme reaction with GGPPS and CaCS. C) HSQC spectrum of labelled **1** obtained from (*E*)-(4-^{13}C,4-^{2}H)IPP. D) HSQC spectrum of labelled **1** obtained from (*Z*)-(4-^{13}C,4-^{2}H)IPP. In both labelling experiments the signals for the CH-correlations of the labelled carbons are strongly enhanced, but the signal for one of the diastereotopic hydrogens is vanished because of the substitution with deuterium. The known configurations at the labelled carbons allow to conclude on the absolute configuration of **1**.

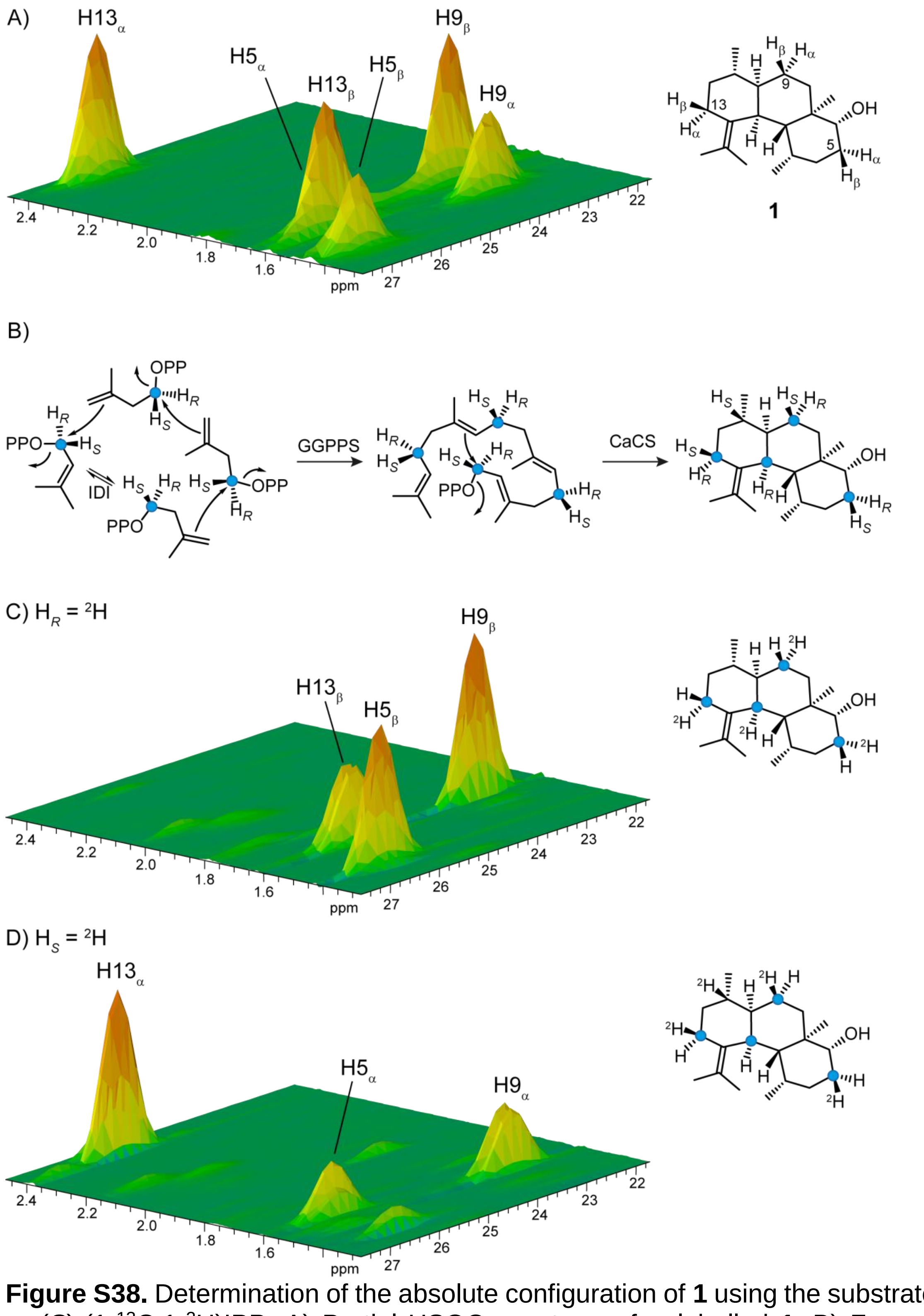

Figure S38. Determination of the absolute configuration of **1** using the substrates (*R*)- or (*S*)-(1-^{13}C,1-^{2}H)IPP. A) Partial HSQC spectrum of unlabelled **1**. B) Formation of labelled **1** in the enzyme reaction with IDI, GGPPS and CaCS. C) HSQC spectrum of labelled **1** obtained from (*R*)-(1-^{13}C,1-^{2}H)IPP. D) HSQC spectrum of labelled **1** obtained from (*S*)-(1-^{13}C,1-^{2}H)IPP. In both labelling experiments the signals for the CH-correlations of the labelled carbons are strongly enhanced, but the signal for one of the diastereotopic hydrogens is vanished because of the substitution with deuterium. The known configurations at the labelled carbons allow to conclude on the absolute configuration of **1**.

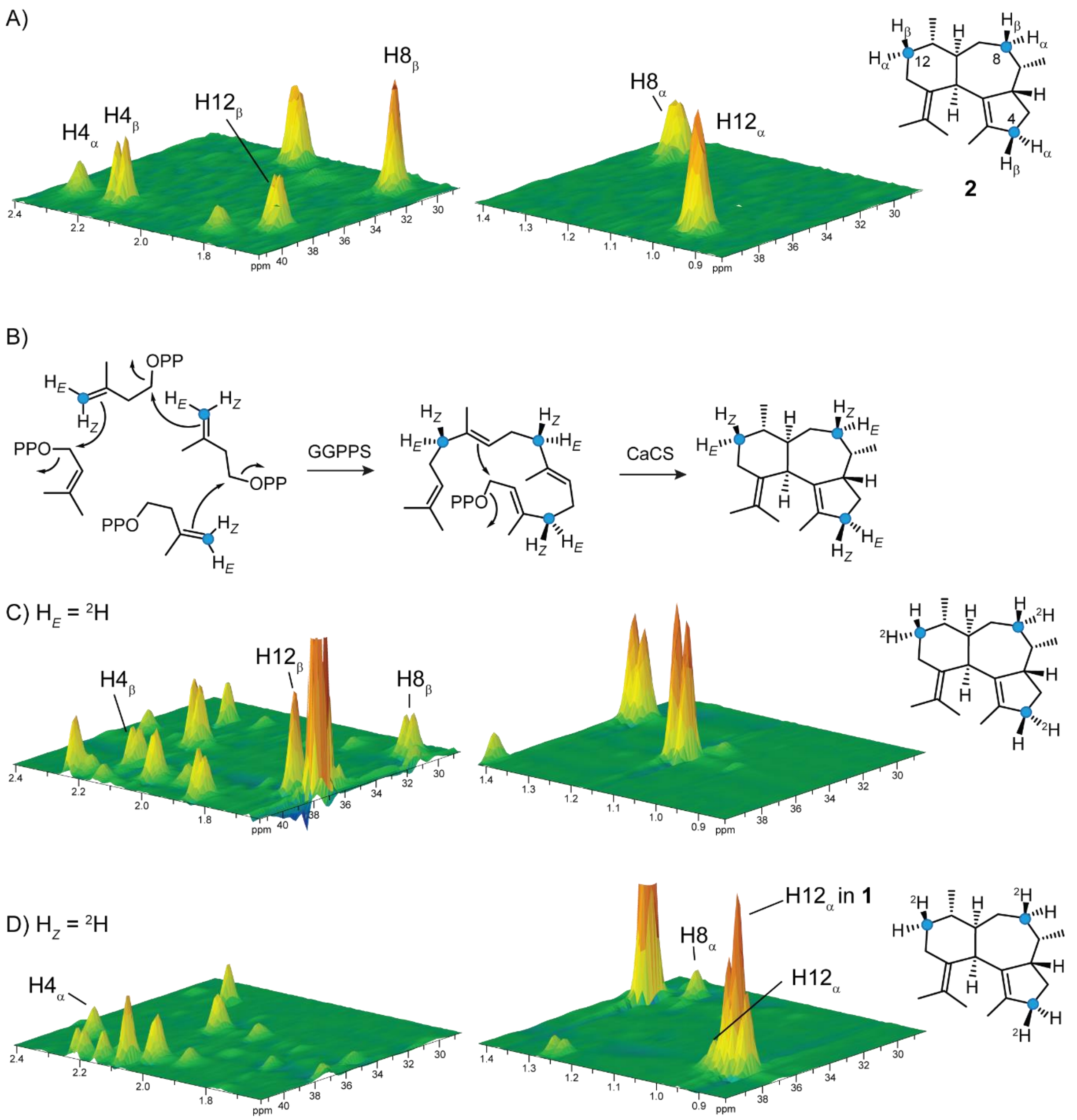

Figure S39. Determination of the absolute configuration of **2** using the substrates DMAPP and (*E*)- or (*Z*)-(4-^{13}C,4-^{2}H)IPP. A) HSQC spectrum of unlabelled **2**. B) Formation of labelled **2** in the enzyme reaction with GGPPS and CaCS. C) HSQC spectrum of labelled **2** obtained from (*E*)-(4-^{13}C,4-^{2}H)IPP. D) HSQC spectrum of labelled **2** obtained from (*Z*)-(4-^{13}C,4-^{2}H)IPP. In both labelling experiments the signals for the CH-correlations of the labelled carbons are strongly enhanced, but the signal for one of the diastereotopic hydrogens is vanished because of the substitution with deuterium. The known configurations at the labelled carbons allow to conclude on the absolute configuration of **2**.

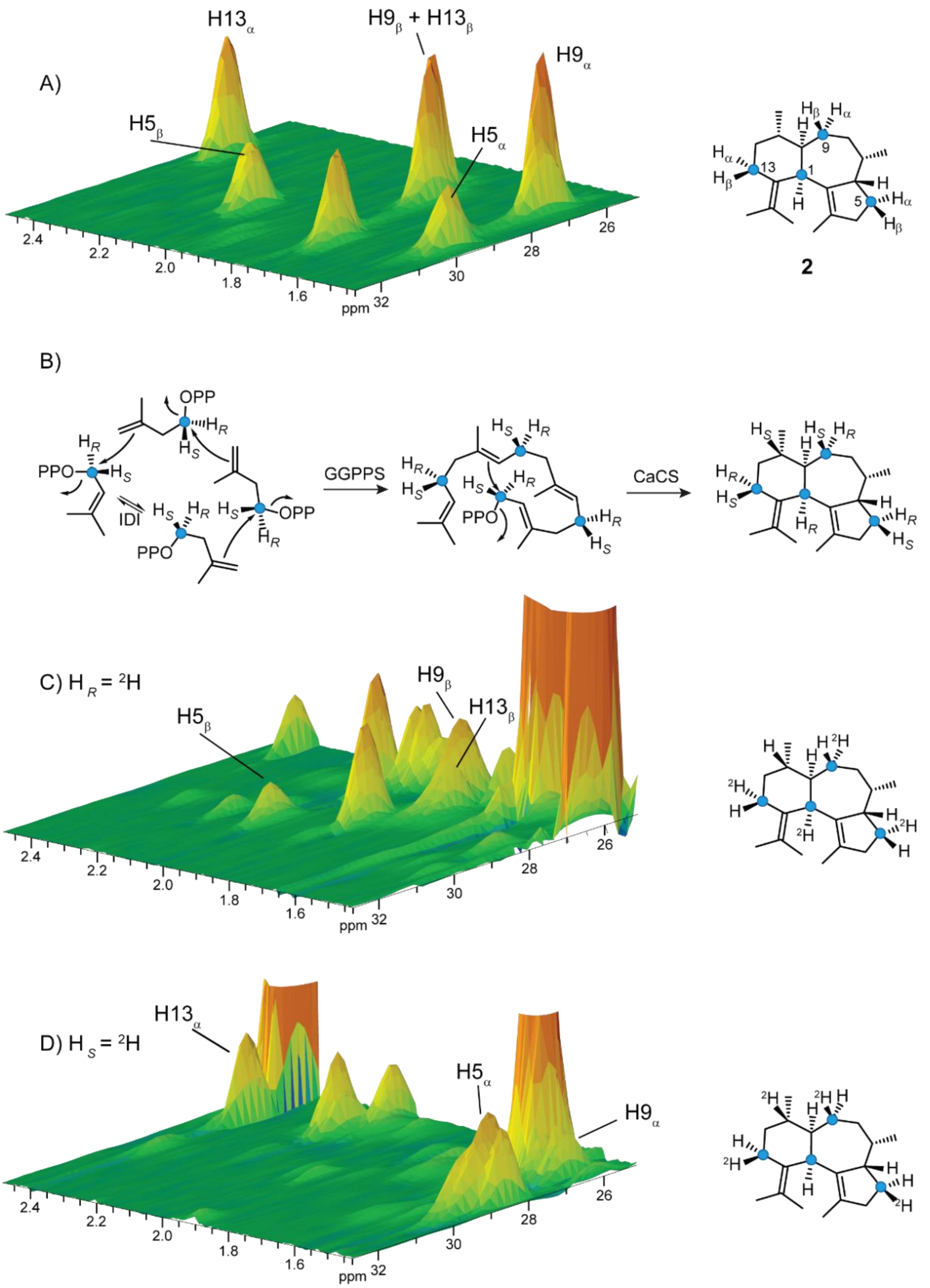

Figure S40. Determination of the absolute configuration of **2** using the substrates (*R*)- or (*S*)-(1-^{13}C,1-^{2}H)IPP. A) Partial HSQC spectrum of unlabelled **2**. B) Formation of labelled **2** in the enzyme reaction with IDI, GGPPS and CaCS. C) HSQC spectrum of labelled **2** obtained from (*R*)-(1-^{13}C,1-^{2}H)IPP. D) HSQC spectrum of labelled **2** obtained from (*S*)-(1-^{13}C,1-^{2}H)IPP. In both labelling experiments the signals for the CH-correlations of the labelled carbons are strongly enhanced, but the signal for one of the diastereotopic hydrogens is vanished because of the substitution with deuterium. The known configurations at the labelled carbons allow to conclude on the absolute configuration of **2**.

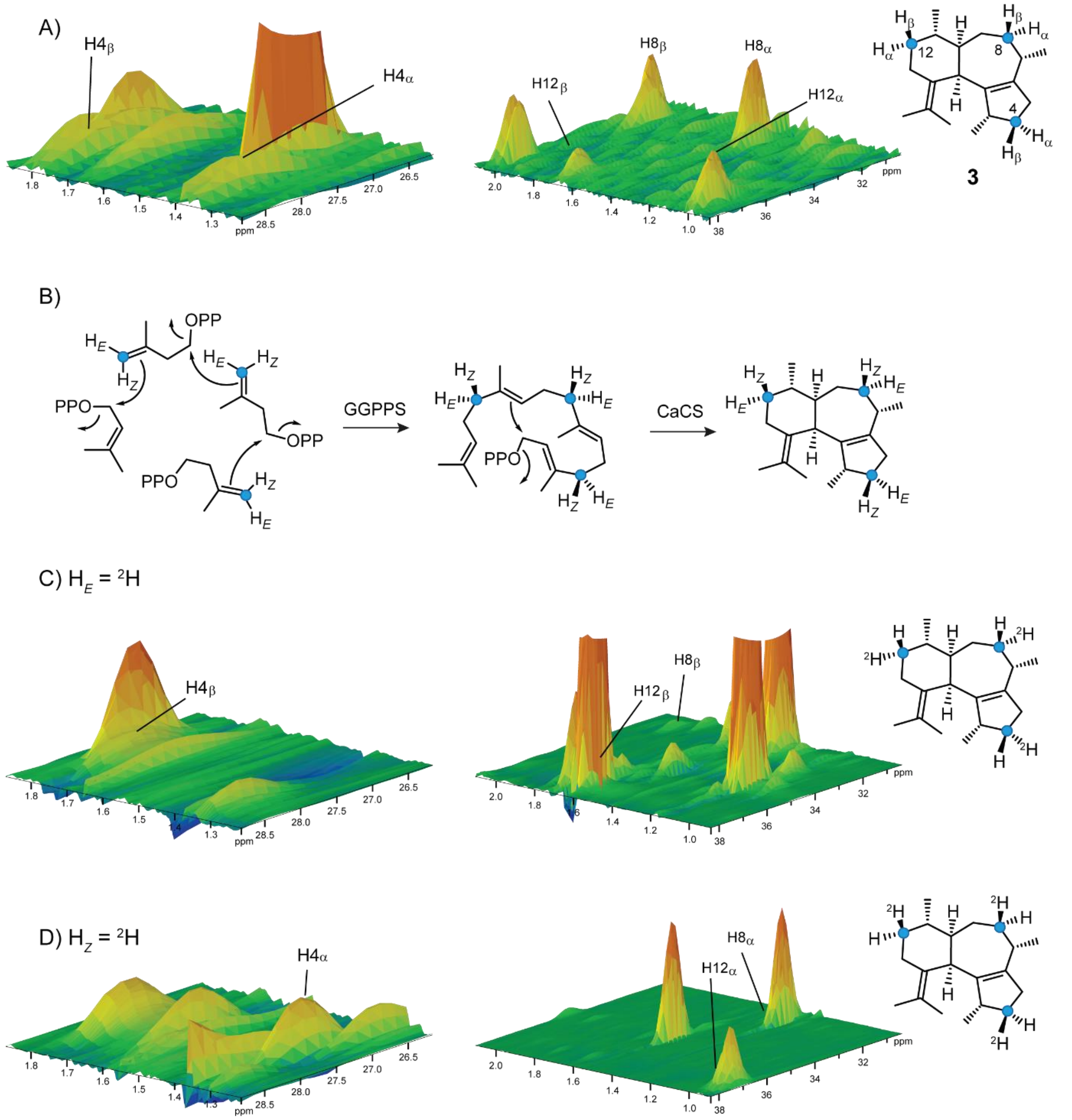

Figure S41. Determination of the absolute configuration of **3** using the substrates DMAPP and (*E*)- or (*Z*)-(4-^{13}C,4-^{2}H)IPP. A) HSQC spectrum of unlabelled **3**. B) Formation of labelled **3** in the enzyme reaction with GGPPS and CaCS. C) HSQC spectrum of labelled **3** obtained from (*E*)-(4-^{13}C,4-^{2}H)IPP. D) HSQC spectrum of labelled **3** obtained from (*Z*)-(4-^{13}C,4-^{2}H)IPP. In both labelling experiments the signals for the CH-correlations of the labelled carbons are strongly enhanced, but the signal for one of the diastereotopic hydrogens is vanished because of the substitution with deuterium. The known configurations at the labelled carbons allow to conclude on the absolute configuration of **3**.

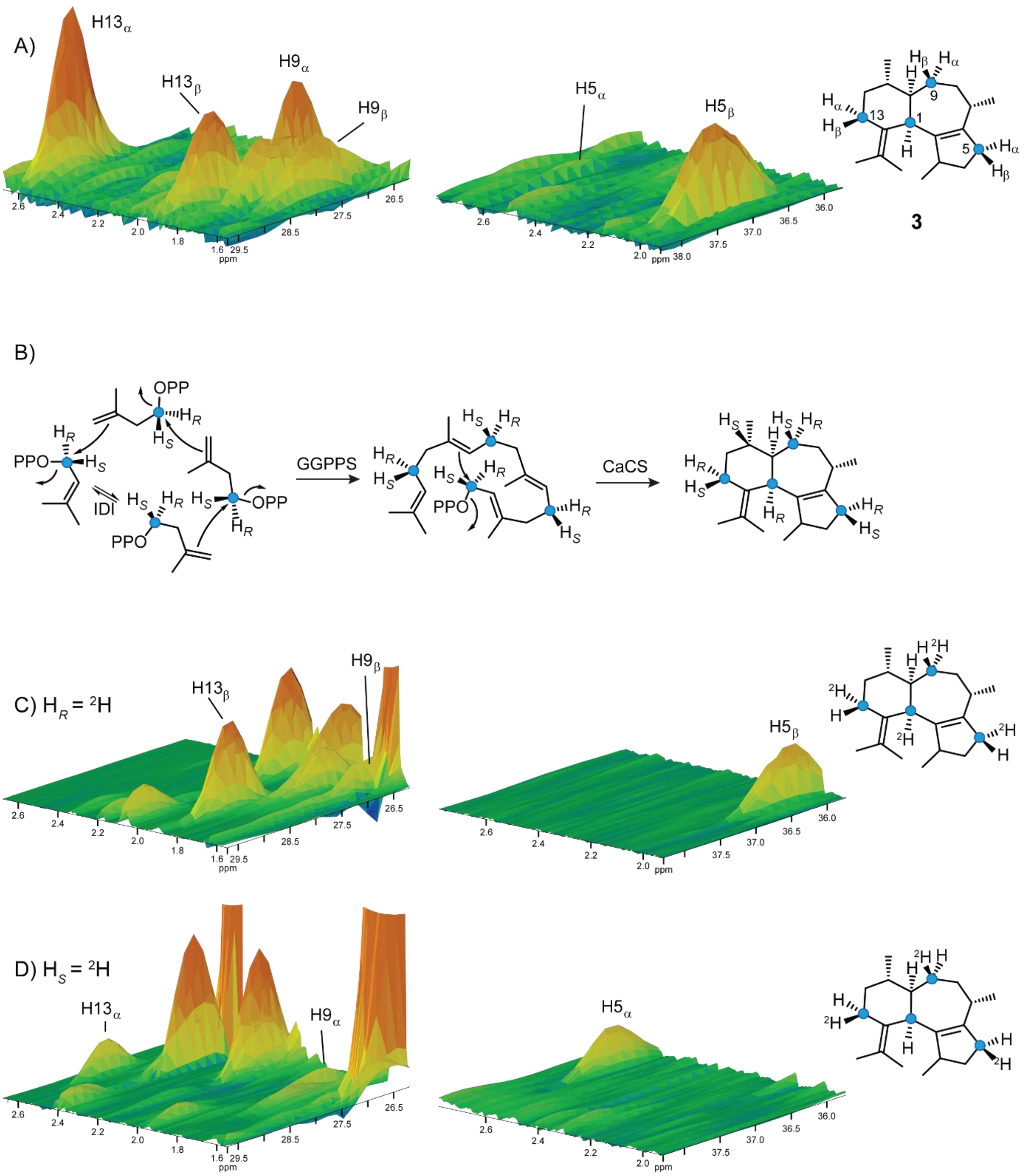

Figure S42. Determination of the absolute configuration of **3** using the substrates (*R*)- or (*S*)-(1-^{13}C,1-^{2}H)IPP. A) Partial HSQC spectrum of unlabelled **3**. B) Formation of labelled **3** in the enzyme reaction with IDI, GGPPS and CaCS. C) HSQC spectrum of labelled **3** obtained from (*R*)-(1-^{13}C,1-^{2}H)IPP. D) HSQC spectrum of labelled **3** obtained from (*S*)-(1-^{13}C,1-^{2}H)IPP. In both labelling experiments the signals for the CH-correlations of the labelled carbons are strongly enhanced, but the signal for one of the diastereotopic hydrogens is vanished because of the substitution with deuterium. The known configurations at the labelled carbons allow to conclude on the absolute configuration of **3**.

General synthetic methods

All chemicals were used without further purification. Silica gel Geduran Si 60 (40 – 63 μm) (Merck, Darmstadt, Germany) was used for flash column chromatography. Solvents for column chromatography were distilled before use. Flame dried flasks were used for reactions with dried solvents or reagents. Argon was used as inert gas. Reaction solvents were dried according to standard procedures. All reactions and chromatographic steps were monitored by TLC (silica, Polygram SIL G/UV254, Macherey-Nagel, Düren, Germany). A solution of phosphomolybdic acid in ethanol (0.1 g mL^{-1}) was used for TLC plate staining.

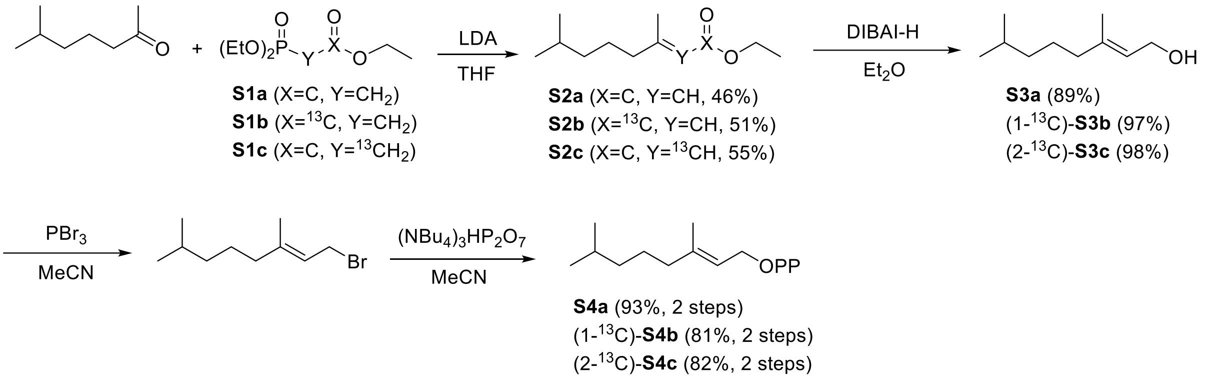

Scheme S1. Synthesis of 6,7-dihydro-GPP, (1-^{13}C)-6,7-dihydro-GPP and (2-^{13}C)-6,7-dihydro-GPP.

Preparation of ethyl (*E*)-6,7-dihydrogeranate (S2a), ethyl (*E*)-(1-^{13}C)-6,7-dihydrogeranate (S2b) and ethyl (*E*)-(2-^{13}C)-6,7-dihydrogeranate (S2c)

A solution of diisopropylamine (1.05 eq.) dissolved in dry THF (5 mL $mmol^{-1}$) was cooled to 0 °C. ^{n}BuLi (1.6 M in hexane, 1.05 eq.) was added dropwise and the reaction was stirred for 1 h at 0 °C. The reaction mixture was cooled to –78 °C and triethyl phosphonoacetate (1.00 eq.) (**S1a**, **S1b** or **S1c**) was added. After stirring the reaction mixture for 1 h at –78 °C, 6-methylhept-2-one (1.00 eq.) was added. The reaction mixture was stirred overnight at 0 °C, warmed to room temperature and quenched with water (5 mL $mmol^{-1}$). The aqueous phase was extracted with diethyl ether for three times. The combined organic layers were dried with $MgSO_4$ and concentrated under reduced pressure. Purification by repeated column chromatography (cyclohexane/EtOAc = 50/1) yielded pure **S2a**, **S2b** and **S2c** as colorless oils.

S2a. Yield: 1.01 g (5.10 mmol, 46%). TLC (cyclohexane/EtOAc = 50/1): R_f = 0.20. HR-MS (APCI): calc. for $[C_{12}H_{23}O_2]^+$ m/z = 199.1693; found m/z = 199.1690. IR (diamond ATR): $\tilde{\nu}$ = 2955 (m), 2935 (m), 2870 (w), 1716 (s), 1649 (m), 1222 (m), 1147 (s), 1045 (m) cm^{-1}. ^{1}H-NMR ($CDCl_3$, 700 MHz): δ = 5.65 (m, 1H, CH), 4.14 (q, $^3J_{H,H}$ = 7.1 Hz, 2H, CH_2), 2.15 (d, $^4J_{H,H}$ = 1.3 Hz, 3H, CH_3), 2.11 (m, 2H, CH_2), 1.54 (m, 1H, CH), 1.46 (m, 2H, CH_2), 1.28 (t, $^3J_{H,H}$ = 7.1 Hz, 3H, CH_3), 1.16 (m, 2H, CH_2), 0.88 (d, $^3J_{H,H}$ = 6.6 Hz, 6H, 2x CH_3) ppm. ^{13}C-NMR ($CDCl_3$, 175 MHz): δ = 167.1 (C_q), 160.5 (C_q), 115.6 (CH), 59.6 (CH_2), 41.3 (CH_2), 38.6 (CH_2), 28.0 (CH), 25.4 (CH_2), 22.7 (2x CH_3), 18.9 (CH_3), 14.5 (CH_3) ppm. EI-MS (70 eV): m/z (%) = 198 (8), 183 (4), 153 (64), 141 (25), 128 (90), 113 (55), 100 (54), 95 (53), 82 (65), 69 (100), 55 (61), 43 (76), 41 (71). GC (HP-5MS): I = 1364.

S2b. Yield: 1.13 g (5.68 mmol, 51%). TLC (cyclohexane/EtOAc = 50/1): R_f = 0.20. HR-MS (APCI): calc. for $[C_{11}{}^{13}C_1H_{23}O_2]^+$ *m*/*z* = 200.1726; found *m*/*z* = 200.1726. IR (diamond ATR): ṽ = 2955 (m), 2936 (m), 2871 (w), 1676 (s), 1648 (m), 1211 (m), 1135 (s), 1045 (m) cm^{-1}. ^{1}H-NMR ($CDCl_3$, 700 MHz): δ = 5.65 (m, 1H, CH), 4.14 (qd, $^3J_{H,H}$ = 7.1 Hz, $^3J_{C,H}$ = 3.0 Hz, 2H, CH_2), 2.15 (dd, $^4J_{H,H}$ = 1.3 Hz, $^4J_{C,H}$ = 1.3 Hz, 3H, CH_3), 2.11 (t, $^3J_{H,H}$ = 8.1 Hz, 2H, CH_2), 1.54 (non, $^3J_{H,H}$ = 6.6 Hz, 1H, CH), 1.46 (m, 2H, CH_2), 1.28 (t, $^3J_{H,H}$ = 7.1 Hz, 3H, CH_3), 1.16 (m, 2H, CH_2), 0.88 (d, $^3J_{H,H}$ = 6.6 Hz, 6H, 2x CH_3) ppm. ^{13}C-NMR ($CDCl_3$, 175 MHz): δ = 167.1 ($^{13}C_q$), 160.5 (d, $^2J_{C,C}$ = 2.1 Hz, C_q), 115.6 ($^1J_{C,C}$ = 75.6 Hz, CH), 59.6 ($^3J_{C,C}$ = 2.3 Hz, CH_2), 41.3 ($^2J_{C,C}$ = 7.0 Hz, CH_2), 38.6 (CH_2), 28.0 (CH), 25.3 (CH_2), 22.7 (2x CH_3), 18.9 (d, $^3J_{C,C}$ = 1.5 Hz, CH_3), 14.5 (d, $^3J_{C,C}$ = 2.3 Hz, CH_3) ppm. EI-MS (70 eV): *m*/*z* (%) = 199 (7), 184 (4), 154 (60), 142 (23), 129 (83), 114 (48), 110 (44), 101 (49), 95 (39), 83 (76), 69 (77), 55 (57), 43 (100), 41 (90), 39 (41). GC (HP-5MS): *I* = 1376.
S2c. Yield: 220 mg (1.11 mmol, 55%). TLC (cyclohexane/EtOAc = 50/1): R_f = 0.20. HR-MS (APCI): calc. for $[C_{11}{}^{13}C_1H_{23}O_2]^+$ *m*/*z* = 200.1726; found *m*/*z* = 200.1724. IR (diamond ATR): ṽ = 2955 (m), 2936 (m), 2870 (w), 1717 (s), 1622 (m), 1222 (m), 1147 (s), 1043 (m) cm^{-1}. ^{1}H-NMR ($CDCl_3$, 500 MHz): δ = 5.65 (dq, $^1J_{C,H}$ = 159.4 Hz, $^4J_{H,H}$ = 1.3 Hz, 1H, CH), 4.14 (q, $^3J_{H,H}$ = 7.1 Hz, 2H, CH_2), 2.15 (dd, $^3J_{C,H}$ = 4.6, $^4J_{H,H}$ = 1.3 Hz, 3H, CH_3), 2.10 (m, 2H, CH_2), 1.54 (m, 1H, CH), 1.46 (m, 2H, CH_2), 1.28 (t, $^3J_{H,H}$ = 7.1 Hz, 3H, CH_3), 1.16 (m, 2H, CH_2), 0.87 (d, $^3J_{H,H}$ = 6.6 Hz, 6H, 2x CH_3) ppm. ^{13}C-NMR ($CDCl_3$, 125 MHz): δ = 167.1 ($^1J_{C,C}$ = 75.7 Hz, C_q), 160.5 ($^1J_{C,C}$ = 72.1 Hz, C_q), 115.6 (^{13}CH), 59.6 ($^3J_{C,C}$ = 1.6 Hz, CH_2), 41.3 ($^2J_{C,C}$ = 3.4 Hz, CH_2), 38.6 (CH_2), 28.0 (CH), 25.3 ($^3J_{C,C}$ = 2.8 Hz, CH_2), 22.7 (2x CH_3), 18.9 (CH_3), 14.5 (CH_3) ppm. EI-MS (70 eV): *m*/*z* (%) = 199 (13), 184 (6), 154 (81), 142 (30), 129 (100), 114 (60), 101 (57), 95 (42), 83 (73), 70 (62), 55 (45), 43 (80). GC (HP-5MS): *I* = 1379.

Preparation of 6,7-dihydrogeraniol (S3a), (1-^{13}C)-6,7-dihydrogeraniol (S3b) and (2-^{13}C)-6,7-dihydrogeraniol (S3c)
Compound **S2a**, **S2b** or **S2c** (1.0 eq.) was dissolved in dry Et_2O (8 mL $mmol^{-1}$). The solution was cooled to –78 °C and a solution of DIBAl-H (1 M in hexane, 2.1 eq.) was added dropwise. After stirring for 1 h, the mixture was allowed to warm to room temperature, before a saturated aqueous solution of Na-K-tartrate (10 mL $mmol^{-1}$) was added. The mixture was extracted with Et_2O for three times and the combined organic layers were dried with $MgSO_4$ and concentrated under reduced pressure. The crude product was purified by column chromatography (pentane/Et_2O = 7:3) to yield **S3a**, **S3b** and **S3c** as colorless oils.
S3a. Yield: 710 mg (4.55 mmol, 89%). TLC (pentane/Et_2O = 1/1): R_f = 0.45. HR-MS (APCI): calc. for $[C_{10}H_{21}O]^+$ *m*/*z* = 157.1587; found *m*/*z* = 157.1586. IR (diamond ATR): ṽ = 3318 (m), 2954 (s), 2931 (s), 2869 (s), 1466 (m), 1383 (m), 1366 (m), 1000 (s) cm^{-1}. ^{1}H-NMR ($CDCl_3$, 700 MHz): δ = 5.37 (m, 1H, CH), 4.11 (d, $^3J_{H,H}$ = 7.0 Hz, 2H, CH_2), 1.96 (m, 2H, CH_2), 1.63 (s, 3H, CH_3), 1.51 (non, $^3J_{H,H}$ = 6.7 Hz, 1H, CH), 1.39 (m, 2H, CH_2), 1.13 (m, 2H, CH_2), 0.85 (d, $^3J_{H,H}$ = 6.6 Hz, 6H, 2x CH_3) ppm. ^{13}C-NMR ($CDCl_3$, 175 MHz): δ = 140.2 (C_q), 123.2 (CH), 59.4 (CH_2), 39.9 (CH_2), 38.7 (CH_2), 28.0 (CH), 25.6 (CH_2), 22.7 (2x CH_3), 16.2 (CH_3) ppm. EI-MS (70 eV): *m*/*z* (%) = 156 (0.6), 138 (3), 123 (4), 109 (3), 95 (10), 81 (17), 71 (100), 68 (23), 55 (18), 41 (24). GC (HP-5MS): *I* = 1229.
S3b. Yield: 115 mg (0.73 mmol, 97%). TLC (pentane/Et_2O = 1/1): R_f = 0.45. HR-MS (APCI): calc. for $[C_9{}^{13}C_1H_{21}O]^+$ *m*/*z* = 158.1620; found *m*/*z* = 158.1620. IR (diamond ATR): ṽ = 3354 (m), 2954 (s), 2931 (s), 2870 (s), 1467 (m), 1384 (m), 980 (m) cm^{-1}.

^{1}H-NMR ($CDCl_3$, 500 MHz): δ = 5.40 (m, 1H, CH), 4.15 (dd, $^1J_{C,H}$ = 141.8, $^3J_{H,H}$ = 7.0 Hz, 2H, CH_2), 1.99 (m, 2H, CH_2), 1.67 (s, 3H, CH_3), 1.53 (non, $^3J_{H,H}$ = 6.7 Hz, 1H, CH), 1.41 (m, 2H, CH_2), 1.15 (m, 2H, CH_2), 0.87 (d, $^3J_{H,H}$ = 6.6 Hz, 6H, 2x CH_3) ppm. ^{13}C-NMR ($CDCl_3$, 125 MHz): δ = 140.4 ($^2J_{C,C}$ = 1.4 Hz, C_q), 123.2 ($^1J_{C,C}$ = 47.4 Hz, CH), 59.6 ($^{13}CH_2$), 39.9 ($^3J_{C,C}$ = 4.8 Hz, CH_2), 38.8 (CH_2), 28.0 (CH), 25.6 (CH_2), 22.8 (2x CH_3), 16.3 ($^3J_{C,C}$ = 4.3 Hz, CH_3) ppm. EI-MS (70 eV): *m*/*z* (%) = 157 (0.4), 139 (4), 124 (5), 109 (2), 96 (9), 82 (18), 72 (100), 69 (31), 56 (17), 41 (36). GC (HP-5MS): *I* = 1230.

S3c. Yield: 170 mg (1.08 mmol, 98%). TLC (pentane/Et_2O = 1/1): R_f = 0.45. HR-MS (APCI): calc. for $[C_9{}^{13}C_1H_{21}O]^+$ *m*/*z* = 158.1620; found *m*/*z* = 158.1618. IR (diamond ATR): ṽ = 3352 (m), 2954 (s), 2931 (s), 2870 (s), 1467 (m), 1384 (m), 1366 (m), 979 (s) cm^{-1}. ^{1}H-NMR ($CDCl_3$, 500 MHz): δ = 5.41 (dtq, $^1J_{C,H}$ = 153.0 Hz, $^3J_{H,H}$ = 7.0 Hz, $^4J_{H,H}$ = 1.3 Hz, 1H, CH), 4.15 (ddd, $^3J_{H,H}$ = 7.0 Hz, $^2J_{C,H}$ = 4.0 Hz, $^5J_{H,H}$ = 0.5 Hz, 2H, CH_2), 1.99 (m, 2H, CH_2), 1.67 (brd, $^3J_{C,H}$ = 4.9 Hz, 3H, CH_3), 1.54 (non, $^3J_{H,H}$ = 6.7 Hz, 1H, CH), 1.41 (m, 2H, CH_2), 1.15 (m, 2H, CH_2), 0.85 (d, $^3J_{H,H}$ = 6.6 Hz, 6H, 2x CH_3) ppm. ^{13}C-NMR ($CDCl_3$, 125 MHz): δ = 140.4 ($^1J_{C,C}$ = 72.7 Hz, C_q), 123.2 (^{13}CH), 59.6 ($^1J_{C,C}$ = 47.5 Hz, CH_2), 39.9 ($^2J_{C,C}$ = 2.5 Hz, CH_2), 38.8 (CH_2), 28.0 (CH), 25.6 ($^3J_{C,C}$ = 2.9 Hz, CH_2), 22.8 (2x CH_3), 16.3 ($^2J_{C,C}$ = 1.6 Hz, CH_3) ppm. EI-MS (70 eV): *m*/*z* (%) = 157 (1), 139 (5), 124 (6), 109 (2), 96 (10), 82 (21), 72 (100), 69 (28), 56 (16), 41 (40). GC (HP-5MS): *I* = 1228.

Preparation of trisammonium salts of 6,7-dihydro-GPP (S4a), (1-^{13}C)-6,7-dihydro-GPP (S4b) and (2-^{13}C)-6,7-dihydro-GPP (S4c)

A solution of **S3a**, **S3b** or **S3c** in THF (3 mL $mmol^{-1}$, 1.0 eq.) was cooled to 0 °C and PBr_3 (0.4 eq.) was added. The reaction mixture was stirred for 1 hour, poured on ice water and extracted with Et_2O for 3 times. The combined organic layers were dried by $MgSO_4$ and concentrated under reduced pressure. The crude bromide was dissolved in dried MeCN (2 mL $mmol^{-1}$) and a solution of $(NBu_4)_3HP_2O_7$ (1.5 eq.) in MeCN (2 mL $mmol^{-1}$) was added. The reaction was stirred over night at room temperature, concentrated under reduced pressure and dissolved in exchange buffer (100 mL, 25 mM NH_4HCO_3 solution in 2% ^{i}PrOH/H_2O). The solution was applied to a DOWEX® 50WX8 cation exchange resin (NH_4^+ form, 100-200 mesh). The salt was eluted by adding 2 column volumes of exchange buffer and the solution was lyophilised to yield **S4a**, **S4b** or **S4c** as white solids.

S4a. Yield: 1.55 g (4.2 mmol, 93%). HR-MS (ESI–): $[M–H]^-$ calc. for $[C_{10}H_{21}O_7P_2]^-$ *m*/*z* = 315.0768; found *m*/*z* = 315.0766. ^{1}H-NMR (D_2O, 500 MHz): δ = 5.42 (t, $^3J_{H,H}$ = 7.1 Hz, 1H, CH), 4.44 (dd, $^3J_{H,H}$ = 6.5 Hz, $^3J_{H,H}$ = 6.5 Hz, 2H, CH_2), 2.02 (m, 2H, CH_2), 1.68 (s, 3H, CH_3), 1.51 (m, 1H, CH), 1.41 (m, 2H, CH_2), 1.13 (m, 2H, CH_2), 0.83 (d, $^3J_{H,H}$ = 6.6 Hz, 6H, 2x CH_3) ppm. ^{13}C-NMR (D_2O, 125 MHz): δ = 143.6 (C_q), 119.4 ($^3J_{C,P}$ = 8.5 Hz, CH), 62.5 ($^2J_{C,P}$ = 5.2 Hz, CH_2), 39.1 (CH_2), 37.9 (CH_2), 27.1 (CH), 24.7 (CH_2), 21.9 (2x CH_3), 15.5 (CH_3) ppm. ^{31}P-NMR (D_2O, 203 MHz): δ = –6.72 (d, $^2J_{P,P}$ = 21.8 Hz, 1P), –10.28 (d, $^2J_{P,P}$ = 21.8 Hz, 1P) ppm.

S4b. Yield: 220 mg (0.59 mmol, 81%). HR-MS (ESI–): $[M–H]^-$ calc. for $[C_9{}^{13}C_1H_{21}O_7P_2]^-$ *m*/*z* = 316.0802; found *m*/*z* = 316.0800. ^{1}H-NMR (D_2O, 500 MHz): δ = 5.45 (t, $^3J_{H,H}$ = 6.8 Hz, 1H, CH), 4.46 (ddd, $^1J_{C,H}$ = 146.1 Hz, $^3J_{H,H}$ = 6.6 Hz, $^3J_{H,H}$ = 6.6 Hz, 2H, $^{13}CH_2$), 2.04 (t, $^3J_{H,H}$ = 7.6 Hz, 2H, CH_2), 1.70 (s, 3H, CH_3), 1.54 (m, 1H, CH), 1.44 (m, 2H, CH_2), 1.15 (m, 2H, CH_2), 0.85 (d, $^3J_{H,H}$ = 6.7 Hz, 6H, 2x CH_3) ppm. ^{13}C-NMR (D_2O, 125 MHz): δ = 143.6 (C_q), 119.5 ($^2J_{C,H}$ = 49.8, $^3J_{C,P}$ = 8.5 Hz, CH), 62.5 ($^2J_{C,P}$ = 5.2 Hz, $^{13}CH_2$), 39.1 ($^3J_{C,C}$ = 4.8 Hz, CH_2), 37.9 (CH_2), 27.1 (CH), 24.7

(CH_2), 21.9 (2x CH_3), 15.5 ($^3J_{C,C}$ = 4.3 Hz, CH_3) ppm. ^{31}P-NMR (D_2O, 203 MHz): δ = –6.45 (d, $^2J_{P,P}$ = 22.1 Hz, 1P), –10.30 (dd, $^2J_{P,P}$ = 22.1, $^2J_{C,P}$ = 5.2 Hz, 1P) ppm.
S4c. Yield: 330 mg (0.89 mmol, 82%). HR-MS (ESI–): $[M–H]^-$ calc. for $[C_9{}^{13}C_1H_{21}O_7P_2]^-$ *m*/*z* = 316.0802; found *m*/*z* = 316.0804. ^{1}H-NMR (D_2O, 500 MHz): δ = 5.42 (dt, $^1J_{C,H}$ = 155.2 Hz, $^3J_{H,H}$ = 7.2 Hz, 1H, ^{13}CH), 4.44 (m, 2H, CH_2), 2.02 (m, 2H, CH_2), 1.69 (d, $^3J_{C,H}$ = 5.0 Hz, 3H, CH_3), 1.51 (non, $^3J_{H,H}$ = 6.8 Hz, 1H, CH), 1.42 (m, 2H, CH_2), 1.13 (m, 2H, CH_2), 0.83 (d, $^3J_{H,H}$ = 6.6 Hz, 6H, 2x CH_3) ppm. ^{13}C-NMR (D_2O, 125 MHz): δ = 143.7 (d, $^1J_{C,C}$ = 72.3 Hz, 1C, C_q), 119.5 (d, $^3J_{C,P}$ = 8.6 Hz, 1C, ^{13}CH), 62.5 (dd, $^1J_{C,C}$ = 49.7, $^2J_{C,P}$ = 5.3 Hz, 1C, CH_2), 39.1 (d, $^2J_{C,C}$ = 2.7 Hz, 1C, CH_2), 38.0 (CH_2), 27.2 (CH), 24.8 (d, $^3J_{C,C}$ = 2.8 Hz, 1C, CH_2), 22.0 (2x CH_3), 15.6 (d, $^2J_{C,C}$ = 1.4 Hz, CH_3) ppm. ^{31}P-NMR (D_2O, 203 MHz): δ = –6.5 (d, $^2J_{P,P}$ = 21.9 Hz, 1P), –10.2 (dd, $^2J_{P,P}$ = 21.9, $^3J_{C,P}$ = 8.6 Hz, 1P) ppm.

Incubation experiments with substrate analogs and recombinant CaCS
Test incubations were performed with 6,7-dihydro-GPP (1 mg) and IPP (1 mg) dissolved in substrate buffer (1 mL; 25 mM NH_4HCO_3) and diluted with binding buffer (2.5 mL) and incubation buffer (4 mL; 50 mM Tris/HCl, 10 mM $MgCl_2$, 20% glycerol, pH = 8.2). Preparations of CaCS (1 mL) obtained from 200 mL expression culture and GGPPS (0.5 mL) obtained from 100 mL expression culture preparation were added, followed by incubation with shaking at 28 °C for 4 h. The products were extracted with benzene (100 μL) and the extracts were analysed by GC/MS.
For preparative isolation of **5** and **6**, large scale incubations were done by dissolving 6,7-dihydro-GPP (80 mg, 0.25 mmol) and IPP (60 mg, 0.25 mmol) in substrate buffer (10 mL). The substrate solution was split into 20 equal portions and each portion was slowly added to a mixture of protein preparations of recombinant CaCS (2.5 mL; from 300 mL expression culture, 1.0 mg/mL), GGPPS (1 mL; from 100 mL expression culture, 0.9 mg/mL), binding buffer (0.5 mL) and incubation buffer (5 mL) (the splitting into small reaction volumes gave a better conversion than one large scale reaction). The reaction mixtures were incubated overnight at 28 °C.
Each reaction mixture was extracted with benzene (2x 1 mL), the combined organic extracts were dried with $MgSO_4$ and concentrated in vacuo. Column chromatography on silica gel with pentane yielded a mixture of **5** and **6** (for HPLC separation cf. page 7 of SI). If the combined organic extracts were not dried with $MgSO_4$, pure **4** was obtained after column chromatography on silica gel with pentane/Et_2O (2:1) elution.

Table S6. NMR data of **5** in C_6D_6 recorded at 298 K.

C[a]	type	^{1}H[b]	^{13}C[b]
1	CH	1.83 (m)	45.3
2	CH	5.70 (s)	123
3	C_q		135
4	CH_2	1.87 (m, H_β)	30.8
		1.95 (m, H_α)	
5	CH_2	1.53 (m, H_β)	26.2
		1.93 (m, H_α)	
6	CH	1.80 (m)	44.7
7	C_q		153
8	CH_2	2.42 (m, H_β)	36.8
		2.06 (m, H_α)	
9	CH_2	1.19 (m, H_β)	27.2
		1.67 (m, H_α)	
10	CH	1.36 (m)	45.7
11	CH	2.01 (m)	32.1
12	CH_2	1.19 (m)	36.4
		1.19 (m)	
13	CH_2	1.30 (m)	26.1
		1.26 (m)	
14	CH_2	1.17 (m)	39.8
		1.17 (m)	
15	CH	1.51 (m)	28.3
16	CH_3	0.90 (d, J = 6.6)	22.9
17	CH_3	0.90 (d, J = 6.6)	22.9
18	CH_3	0.77 (d, J = 6.9)	13.6
19	CH_2	4.83 (s)	104
		4.71 (s)	
20	CH_3	1.65 (s)	24.1

[a] Carbon numbering as shown in main text. [b] Chemical shifts δ in ppm, multiplicity: s = singlet, d = doublet, m = multiplet, coupling constants J are given in Hertz.

Biflora-4,10(19)-diene (5). Yield: 0.7 mg (2.6 μmol, 3%), from 80 mg (220 μmol) GPP trisammonium salt and 60 mg (204 μmol) IPP trisammonium salt. TLC (pentane): R_f = 0.97. IR (diamond ATR): $\tilde{\nu}$ = 2954 (m), 2926 (s), 2858 (m), 1457 (m), 1381 (w), 1081 (w), 1026 (m), 886 (w) cm^{-1}. HR-MS (Q-TOF, 70 eV): calc. for $[C_{20}H_{34}]^+$ m/z = 274.2652; found m/z = 274.2657. Optical rotary power: $[\alpha]_D^{20}$ = +41.0 (c 0.10, C_6H_6).

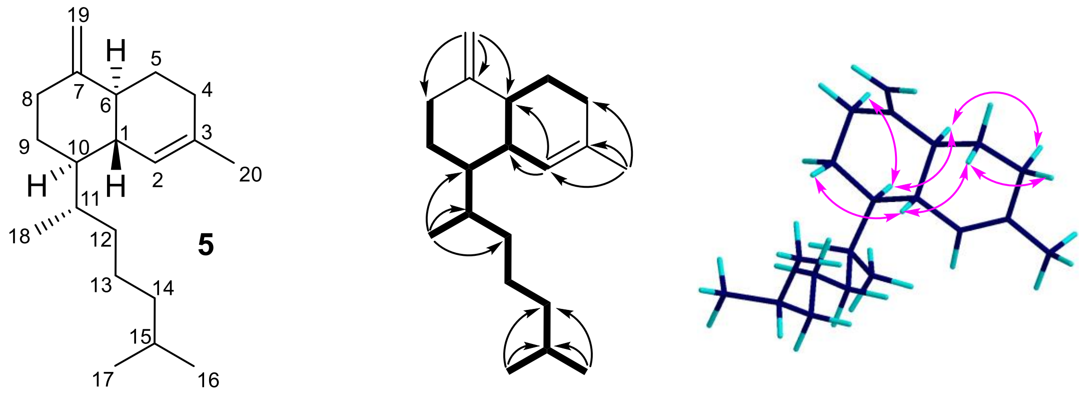

Figure S43. Carbon numbering and structure elucidation of **5**. Bold lines represent $^{1}H,^{1}H$-COSY correlations, selected HMBC signals are represented by single-headed arrows and selected NOE correlations are depicted by double headed arrows.

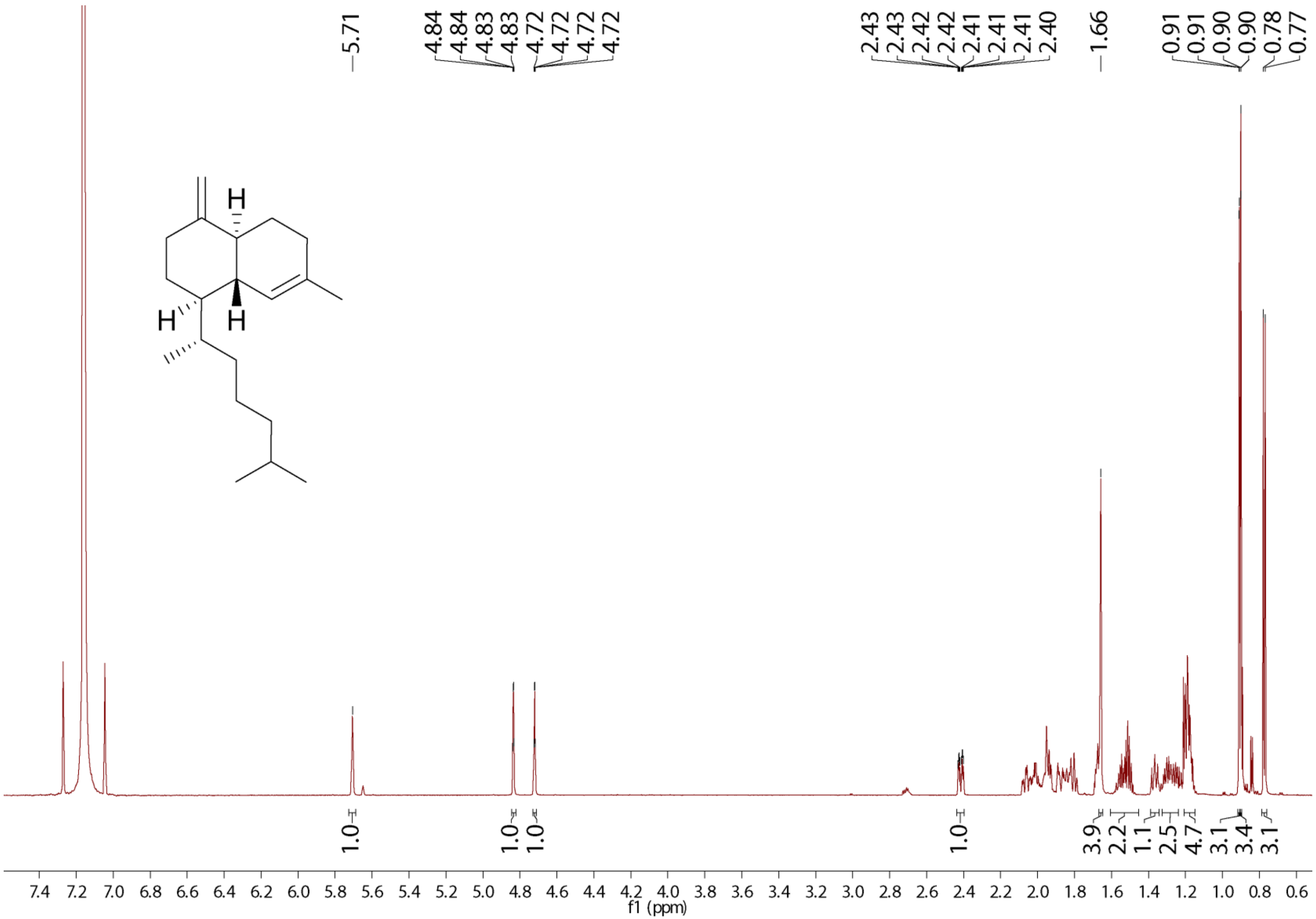

Figure S44. ^{1}H-NMR spectrum of **5** (700 MHz, C_6D_6).

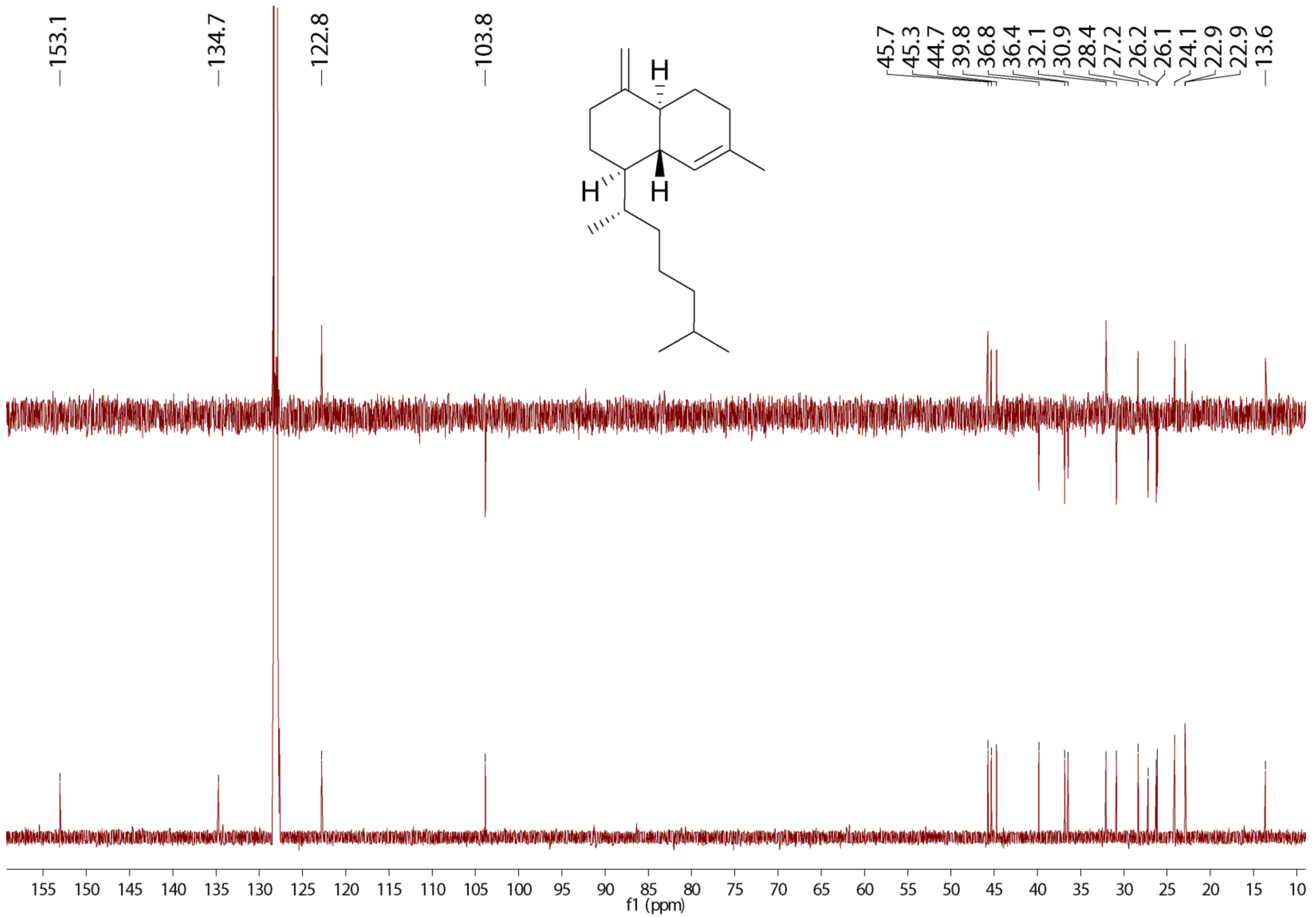

Figure S45. ^{13}C-NMR and ^{13}C-DEPT-135 spectra of **5** (175 MHz, C_6D_6).

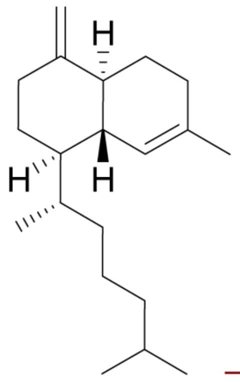

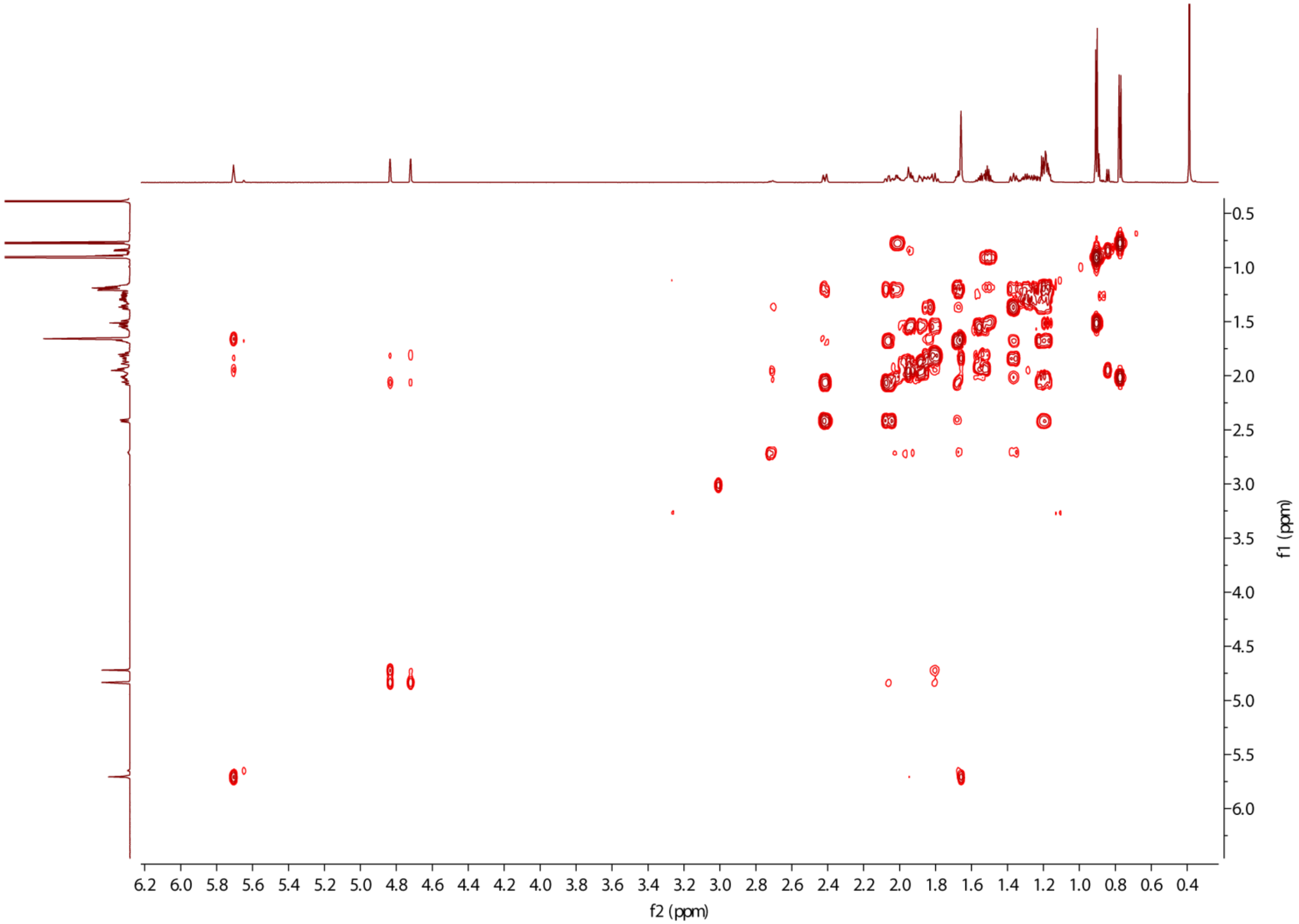

Figure S46. $^{1}H,^{1}H$-COSY spectrum of **5** (C_6D_6).

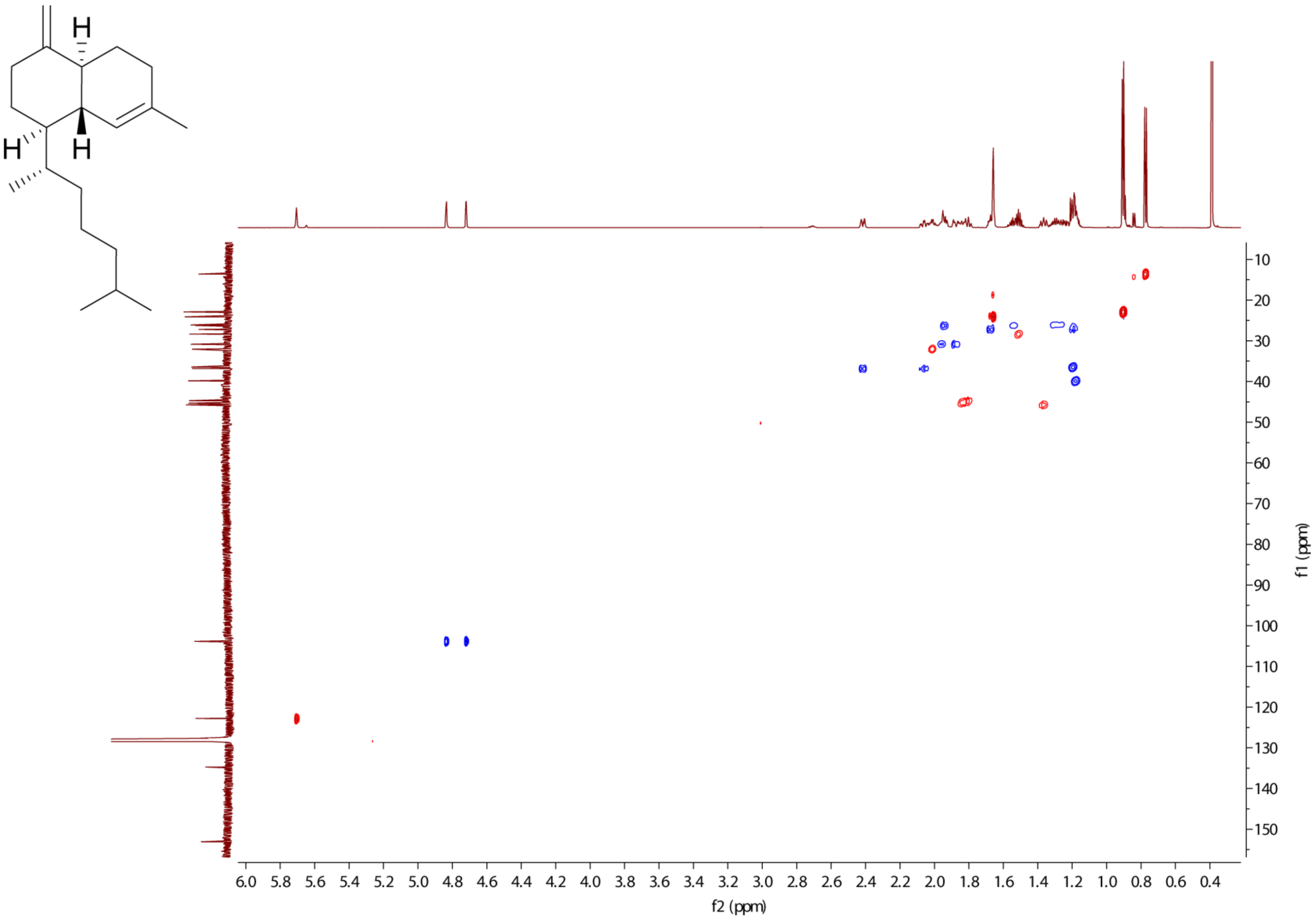

Figure S47. HSQC spectrum of **5** (C_6D_6).

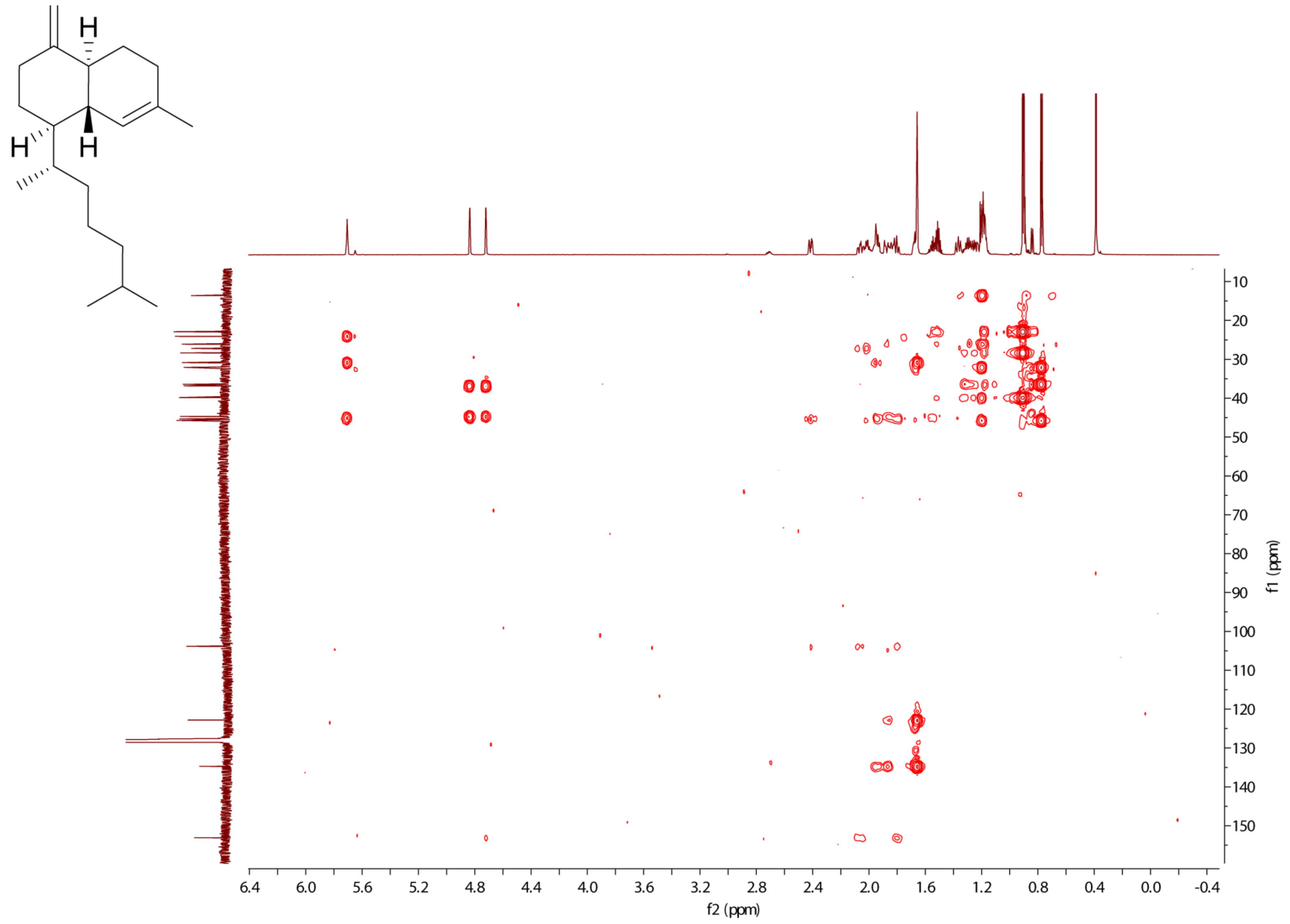

Figure S48. HMBC spectrum of **5** (C_6D_6).

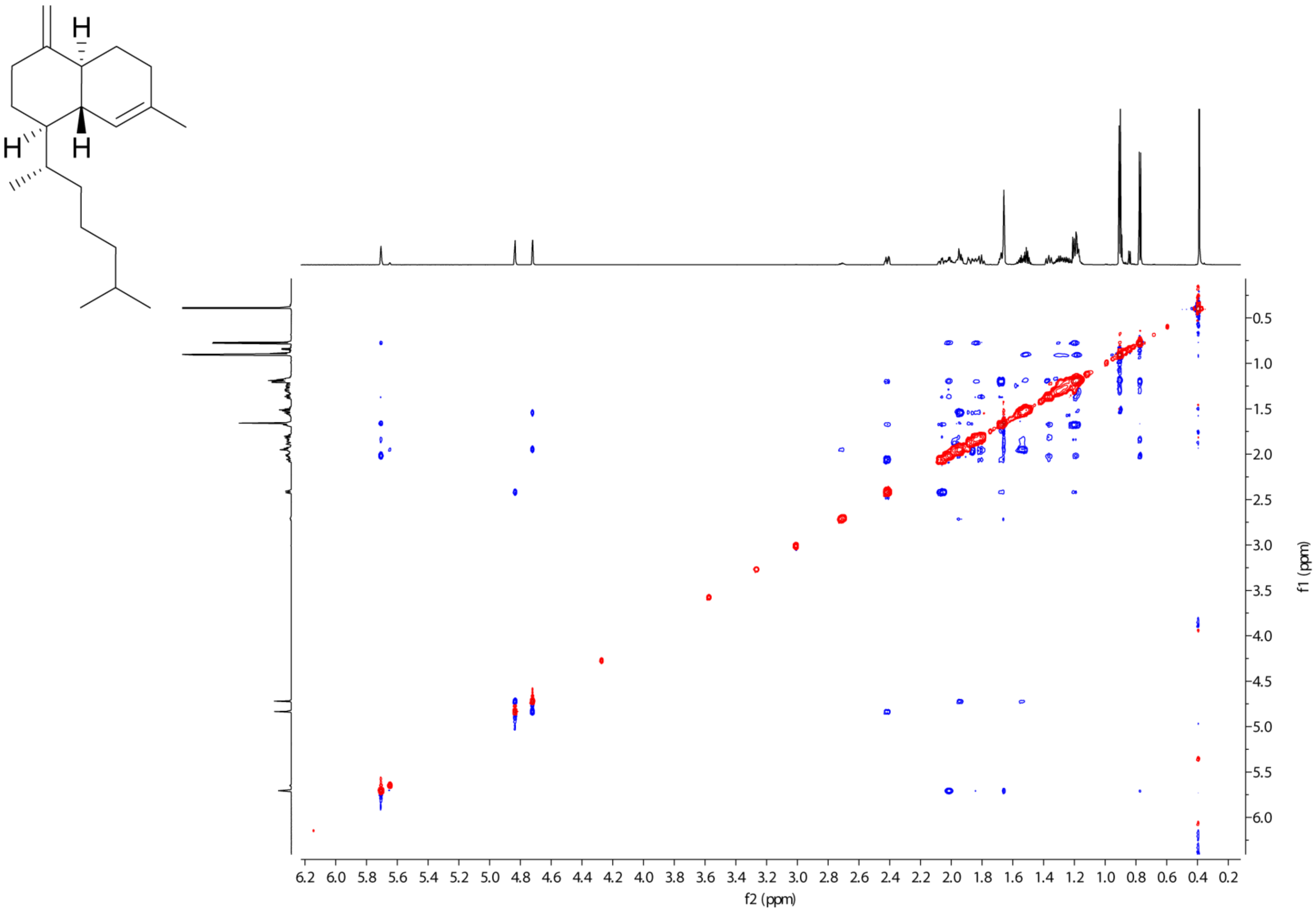

Figure S49. NOESY spectrum of **5** (C_6D_6).

Table S7. NMR data of **6** in C_6D_6 recorded at 298 K.

C[a]	type	^1H[b]	^{13}C[b]
1	CH	2.05 (m)	41.4
2	CH	5.79 (s)	122.8
3	C_q		134.8
4	CH_2	1.91 (m, H_β)<br>1.98 (m, H_α)	31.7
5	CH_2	2.03 (m, H_β)<br>1.28 (m, H_α)	27.1
6	CH	1.99 (m)	43.1
7	C_q		135.8
8	CH	5.48 (m)	122.6
9	CH_2	1.92 (m, H_β)<br>1.90 (m, H_α)	25.6
10	CH	1.69 (m)	41.0
11	CH	2.07 (m)	32.1
12	CH_2	1.20 (m, H_β)<br>1.20 (m, H_α)	35.9
13	CH_2	1.29 (m, H_β)<br>1.29 (m, H_α)	26.0
14	CH_2	1.17 (m, H_β)<br>1.17 (m, H_α)	39.9
15	CH	1.50 (hept, J = 6.6)	28.4
16	CH_3	0.90 (d, J = 6.6)	22.9
17	CH_3	0.90 (d, J = 6.6)	22.9
18	CH_3	0.85 (d, J = 6.9)	13.1
19	CH_3	1.70 (s)	21.0
20	CH_3	1.67 (s)	24.0

[a] Carbon numbering as shown in main text. [b] Chemical shifts δ in ppm, multiplicity: s = singlet, d = doublet, hept = heptet, m = multiplet, coupling constants *J* are given in Hertz.

Biflora-4,10-diene (6). Yield: 0.5 mg (1.8 μmol, 2%), from 80 mg (220 μmol) GPP trisammonium salt and 60 mg (204 μmol) IPP trisammonium salt. TLC (pentane): R_f = 0.97. IR (diamond ATR): ṽ = 2959 (w), 2920 (s), 2851 (m), 1632 (m), 1468 (w), 1421 (w), 1260 (m), 1095 (m), 1019 (m), 802 (m) cm^{-1}. HR-MS (Q-TOF, 70 eV): calc. for $[C_{20}H_{34}]^+$ m/z = 274.2652; found m/z = 274.2649. Optical rotary power: $[\alpha]_D^{20}$ = +2.5 (c 0.05, C_6H_6).

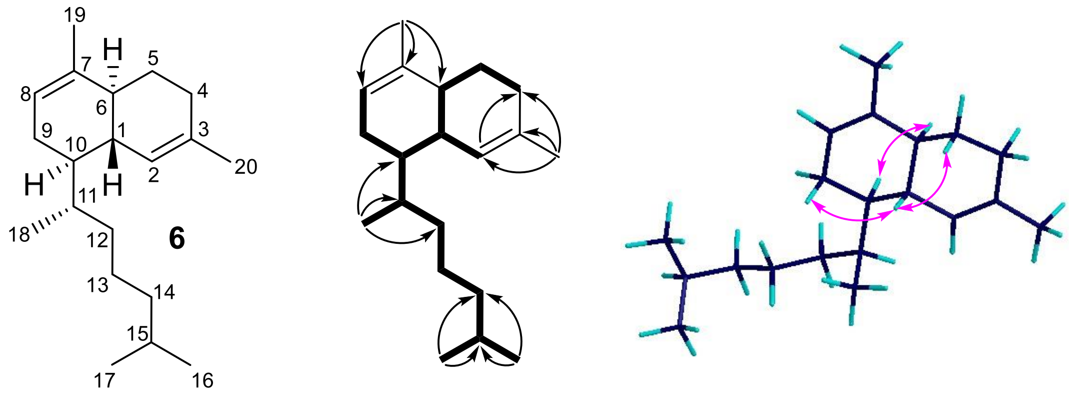

Figure S50. Carbon numbering and structure elucidation of **6**. Bold lines represent $^{1}H,^{1}H$-COSY correlations, selected HMBC signals are represented by single-headed arrows and selected NOE correlations are depicted by double headed arrows.

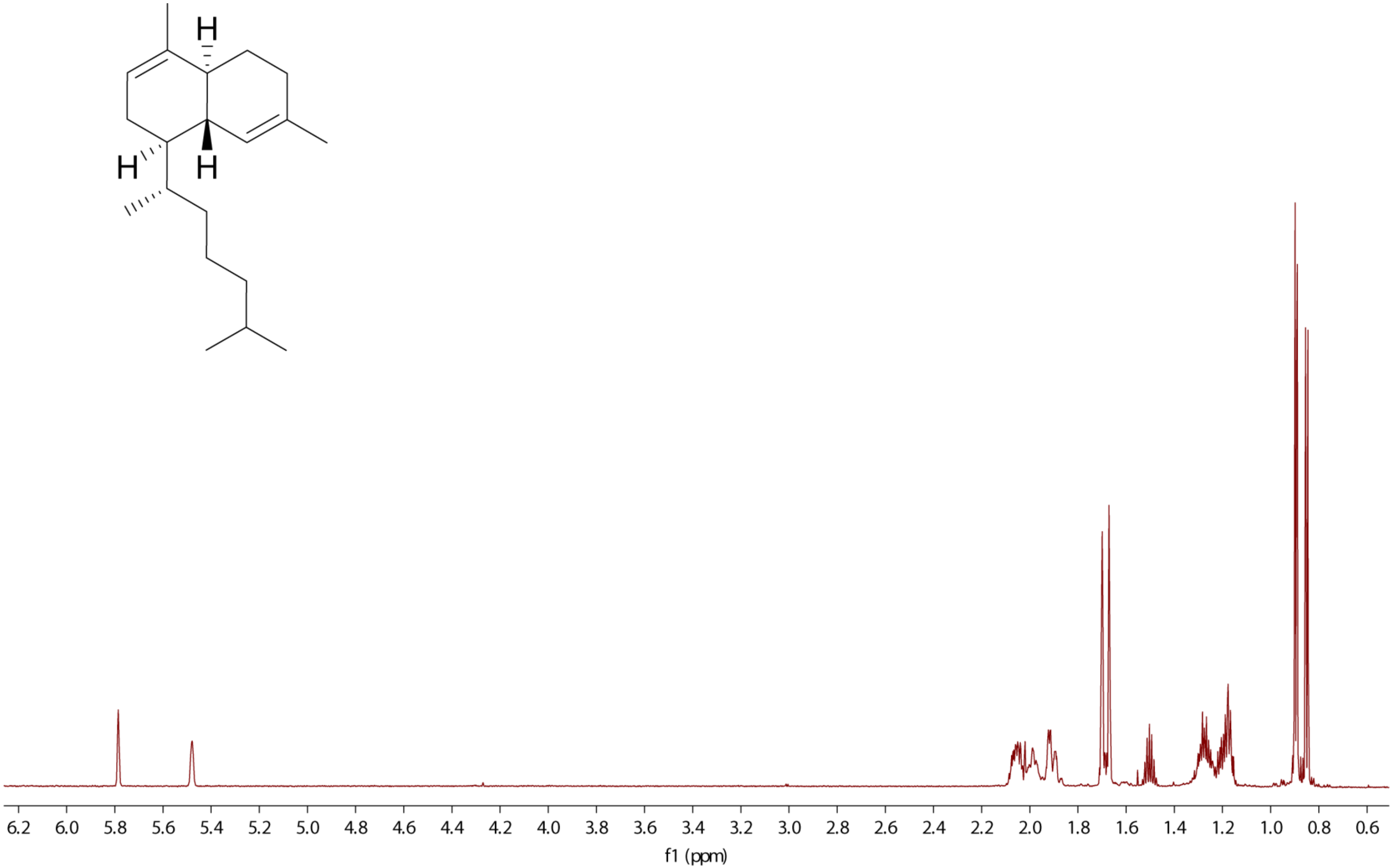

Figure S51. ^{1}H-NMR spectrum of **6** (700 MHz, C_6D_6).

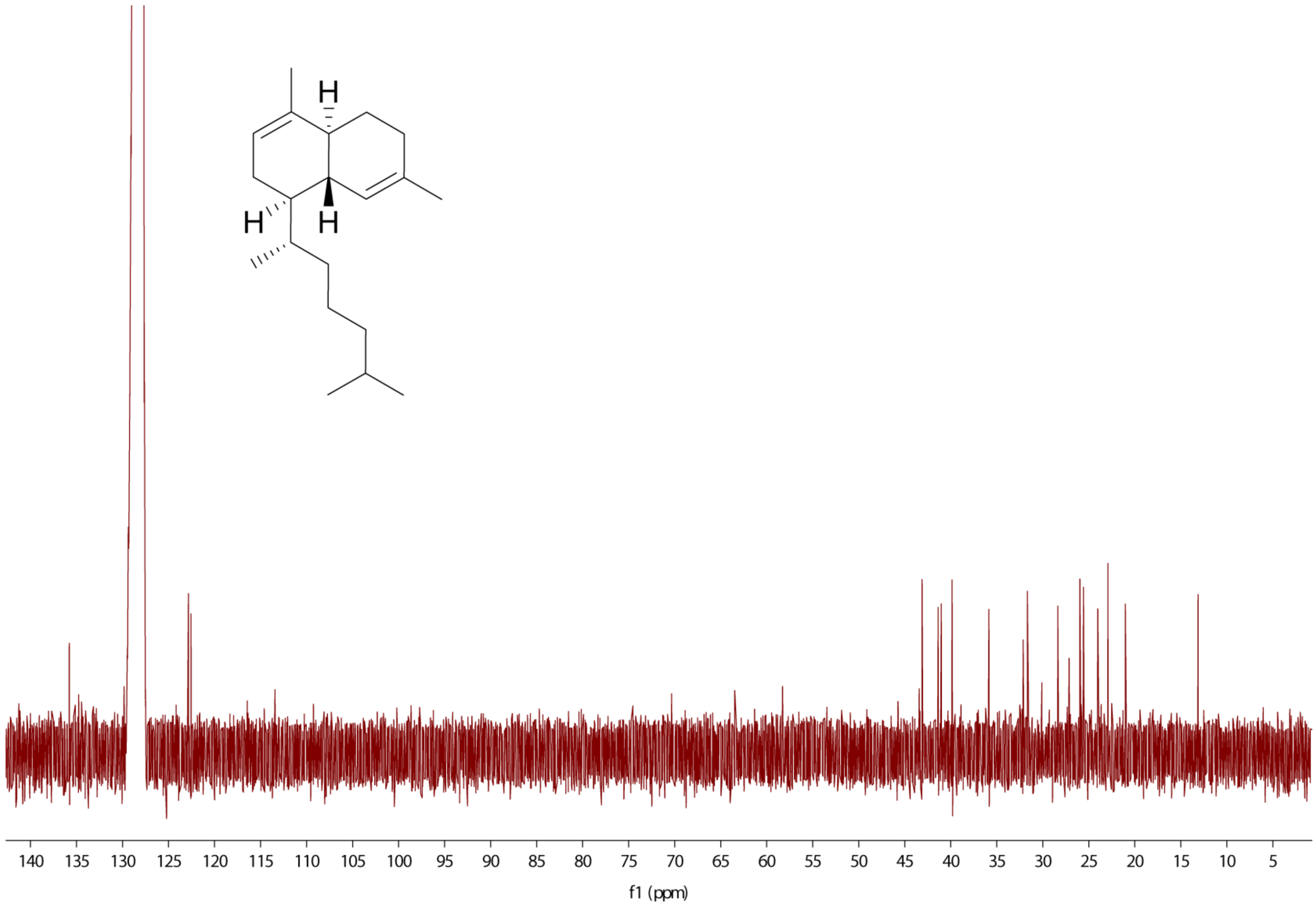

Figure S52. ^{13}C-NMR spectrum of **6** (175 MHz, C_6D_6).

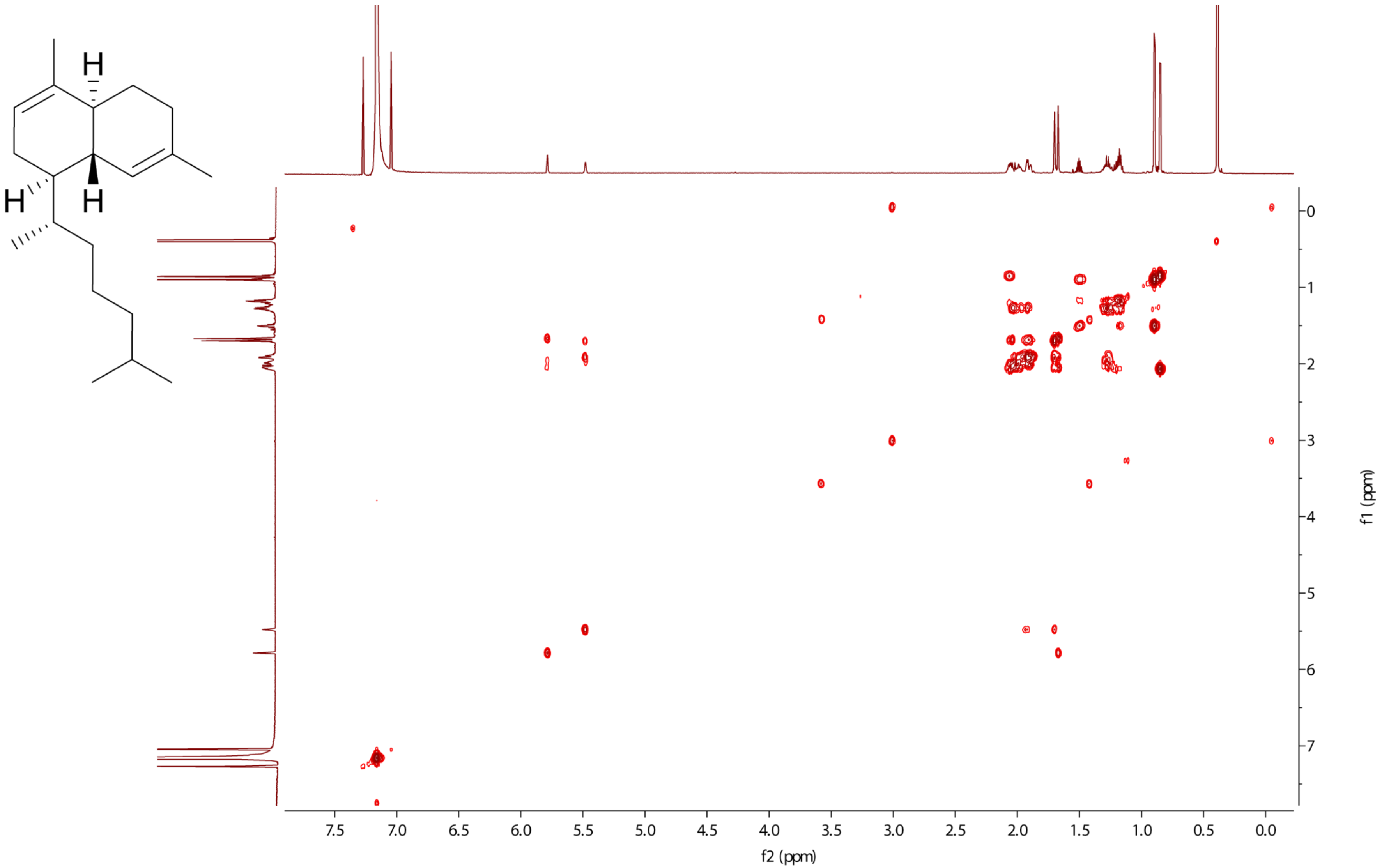

Figure S53. $^{1}H,^{1}H$ COSY spectrum of **6** (C_6D_6).

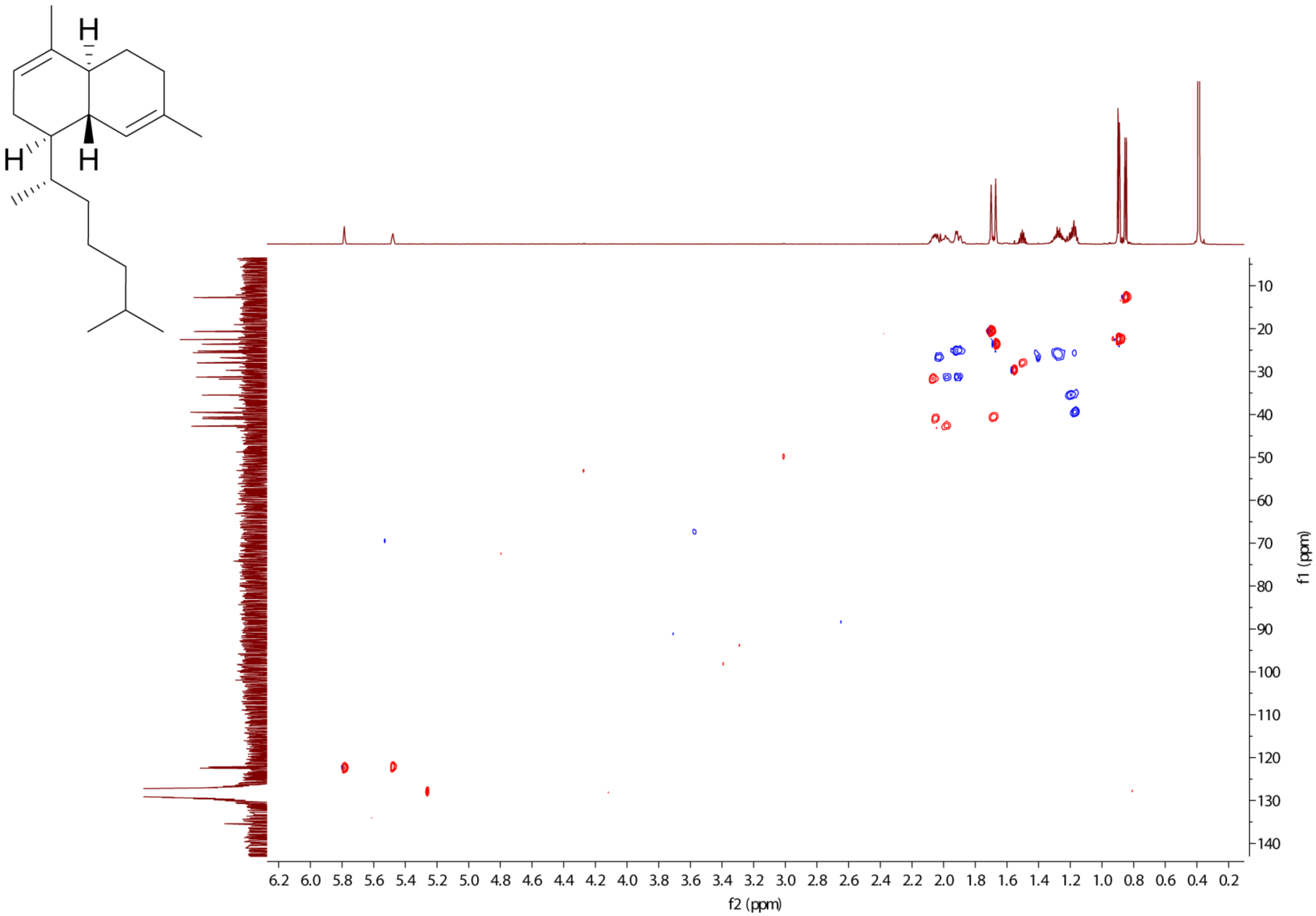

Figure S54. HSQC spectrum of **6** (C_6D_6).

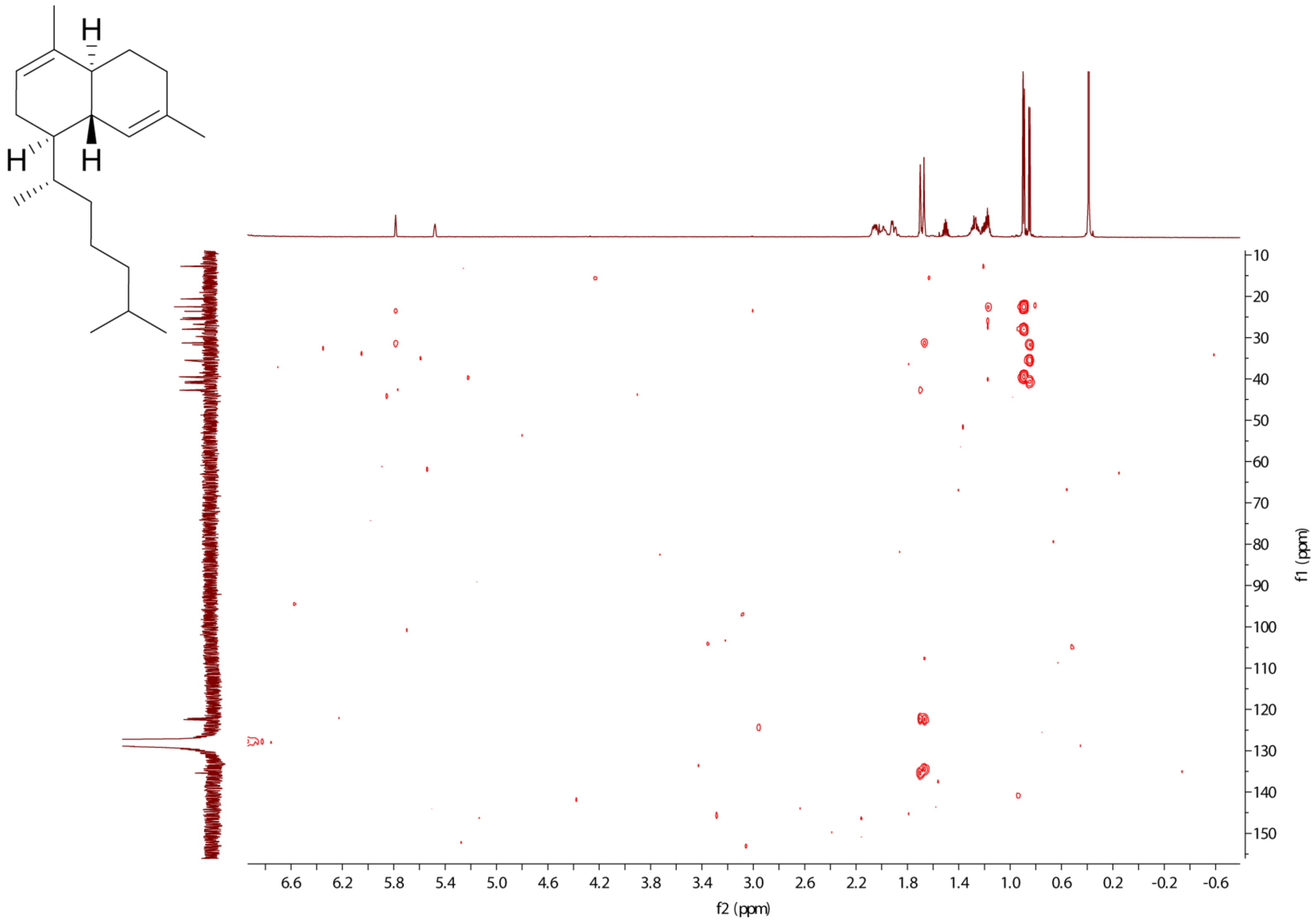

Figure S55. HMBC spectrum of **6** (C_6D_6).

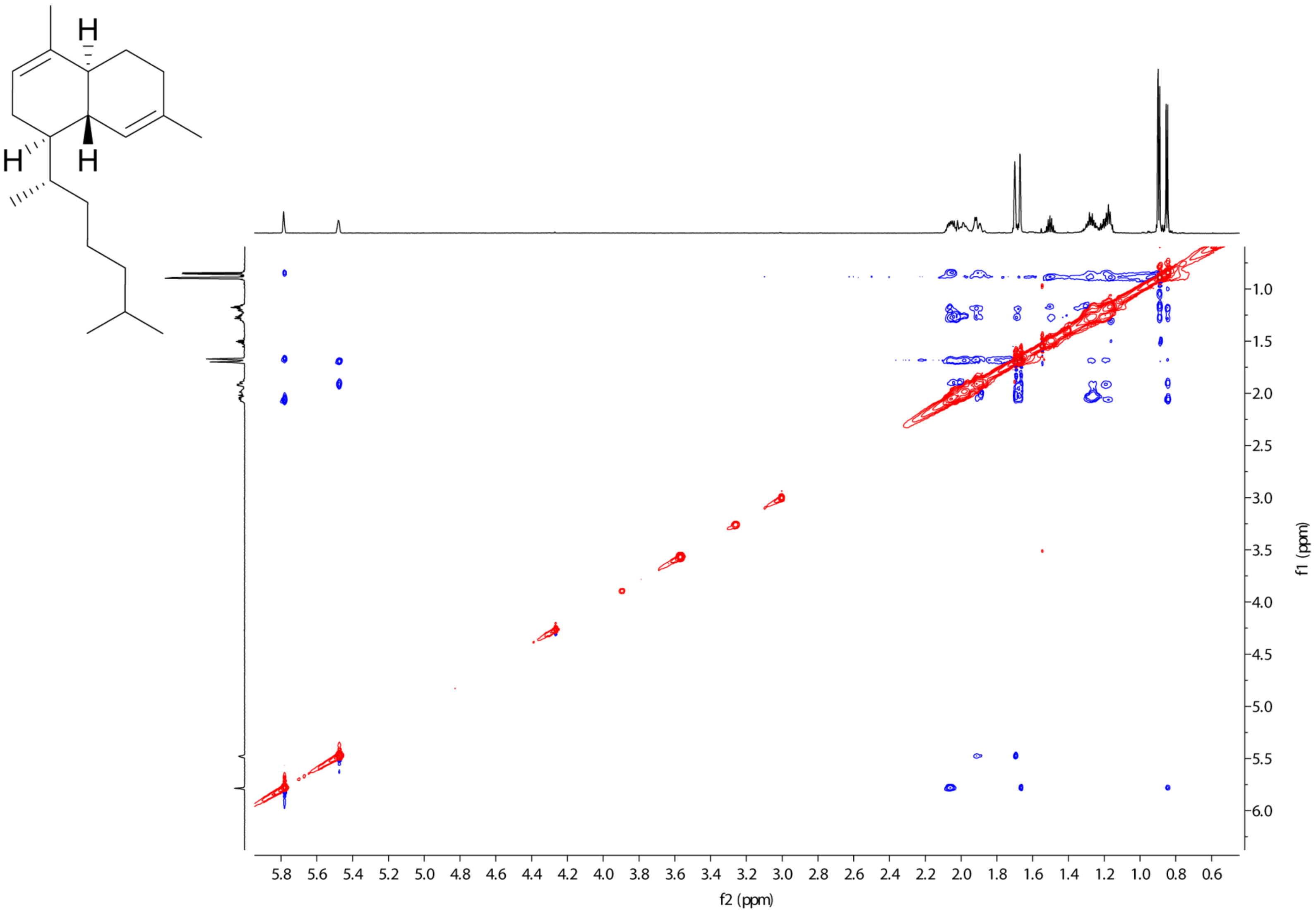

Figure S56. NOESY spectrum of **6** (C_6D_6).

Incubation experiments with isotopically labelled 14,15-dihydro-GGPP from its precursors and CaCS

Isotopic labelling experiments were performed with amounts of ca. 1 mg labelled and unlabelled substrate (as given in Table S8) in substrate buffer (1 mL), incubation buffer (5 mL), CaCS (1 mL; from 300 mL expression culture, 1.0 mg/mL), GGPPS (1 mL; from 100 mL expression culture, 0.9 mg/mL) and binding buffer (to a total volume of 10 mL). After incubation with shaking at 28 °C for 4 h, the products were extracted twice with C_6D_6 (700 μL and 300 μL), and the extracts targeting **4** were directly analysed by NMR and GC/MS. For analysis of labelled compounds **5** and **6**, the extracts were subsequently treated with $MgSO_4$ and then analysed by NMR and GC/MS again.

Table S8. Labeling experiments with CaCS for compounds **4** – **6**.

No.	Substrate	Enzyme(s)	Result shown in
1	(1-^{13}C)IPP[14] + 6,7-dihydro-GPP	CaCS, GGPPS[14]	Figure S57 and S74
2	(1-^{13}C)IPP[14] + 10,11-dihydro-FPP[29]	CaCS, GGPPS[14]	Figure S57 and S74
3	(2-^{13}C)IPP[23] + 6,7-dihydro-GPP	CaCS, GGPPS[14]	Figure S57 and S74
4	(2-^{13}C)IPP[23] + 10,11-dihydro-FPP[29]	CaCS, GGPPS[14]	Figure S57 and S74
5	(3-^{13}C)IPP[14] + 6,7-dihydro-GPP	CaCS, GGPPS[14]	Figure S57 and S74
6	(3-^{13}C)IPP[14] 10,11-dihydro-FPP[29]	CaCS, GGPPS[14]	Figure S57 and S74
7	(4-^{13}C)IPP[14] + 6,7-dihydro-GPP	CaCS, GGPPS[14]	Figure S57 and S74
8	(4-^{13}C)IPP[14] 10,11-dihydro-FPP[29]	CaCS, GGPPS[14]	Figure S57 and S74
9	(5-^{13}C)IPP[30] + 6,7-dihydro-GPP	CaCS, GGPPS[14]	Figure S57 and S74
10	(5-^{13}C)IPP[30] 10,11-dihydro-FPP[29]	CaCS, GGPPS[14]	Figure S57 and S74
11	IPP + (1-^{13}C)-6,7-dihydro-GPP	CaCS, GGPPS[14]	Figure S57 and S74
12	IPP + (2-^{13}C)-6,7-dihydro-GPP	CaCS, GGPPS[14]	Figure S57 and S74
13	(*R*)-(1-^{13}C,1-^{2}H)IPP[6] + 6,7-dihydro-GPP	CaCS, GGPPS[14]	Figure S59, S62 and S82
14	(*S*)-(1-^{13}C,1-^{2}H)IPP[6] + 6,7-dihydro-GPP	CaCS, GGPPS[14]	Figure S59, S62 and S82
15	(*E*)-(4-^{13}C,4-^{2}H)IPP[28] + 6,7-dihydro-GPP	CaCS, GGPPS[14]	Figure S60, S63 and S83
16	(*Z*)-(4-^{13}C,4-^{2}H)IPP[28] + 6,7-dihydro-GPP	CaCS, GGPPS[14]	Figure S60, S63 and S83
17	IPP + (*R*)-(1-^{13}C,1-^{2}H)-6,7-dihydro-GPP	CaCS, GGPPS[14]	Figure S61, S64 and S84
18	IPP + (*S*)-(1-^{13}C,1-^{2}H)-6,7-dihydro-GPP	CaCS, GGPPS[14]	Figure S61, S64 and S84

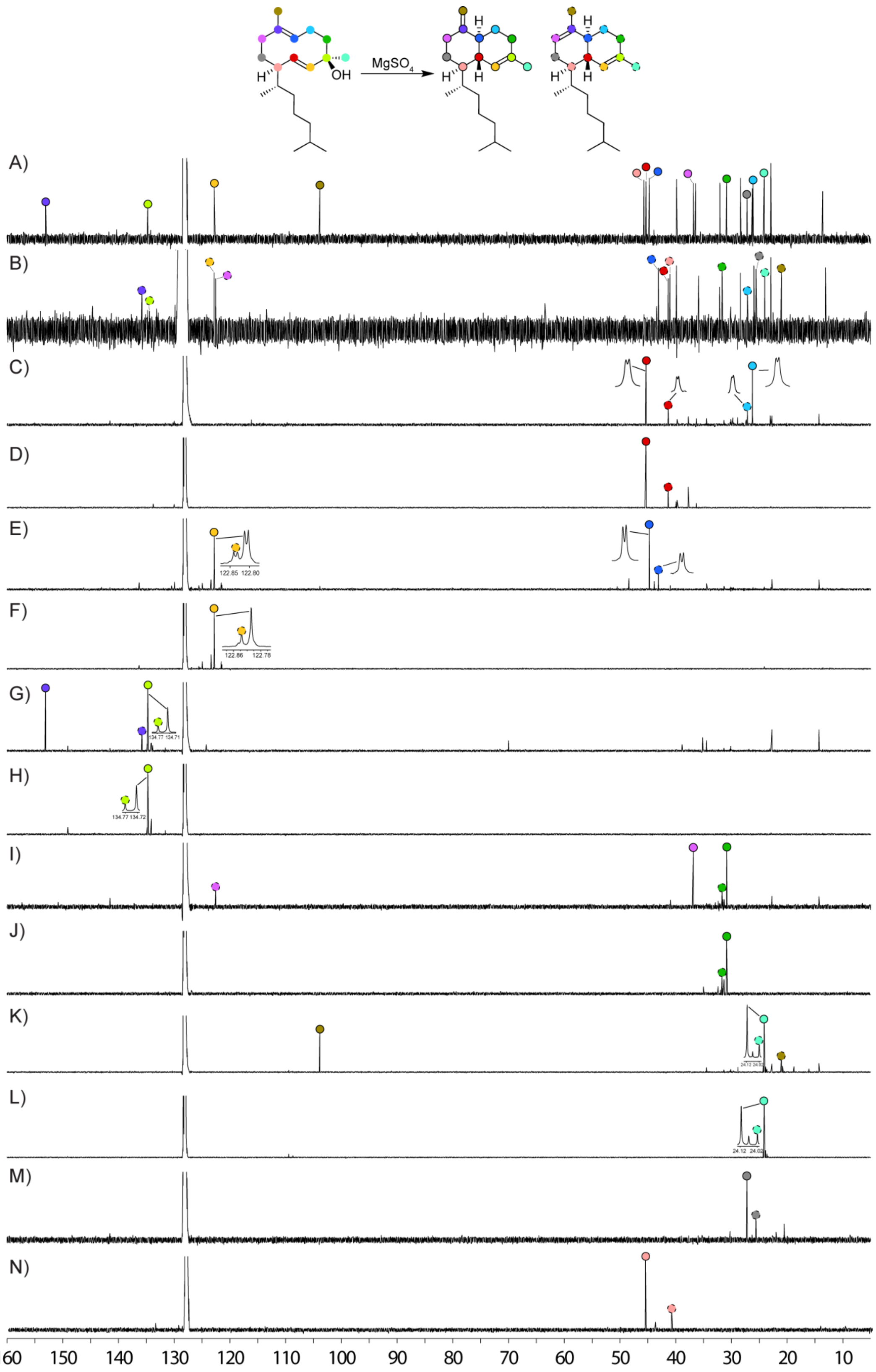

Figure S57. ^{13}C-NMR spectra of A) unlabelled **5**, B) unlabelled **6**, and mixtures of ^{13}C-labelled **5** and **6** obtained from the seven isotopomers of (^{13}C)-14,15-dihydro-GGPP after treatment with $MgSO_4$ labelled at carbons D) C1, F) C2, H) C3, J) C4, L) C20, M) C9, and N) C10. ^{13}C-NMR spectra of ^{13}C-labelled **5** and **6** obtained from the five isotopomers of ($^{13}C_2$)-14,15-dihydro-GGPP labelled C) C1 and C5, E) C2 and C6, G) C3 and C7, I) C4 and C8, and K) C19 and C20. Coloured dots correlate the observed ^{13}C-NMR signals to the carbons of **5** – **6**.

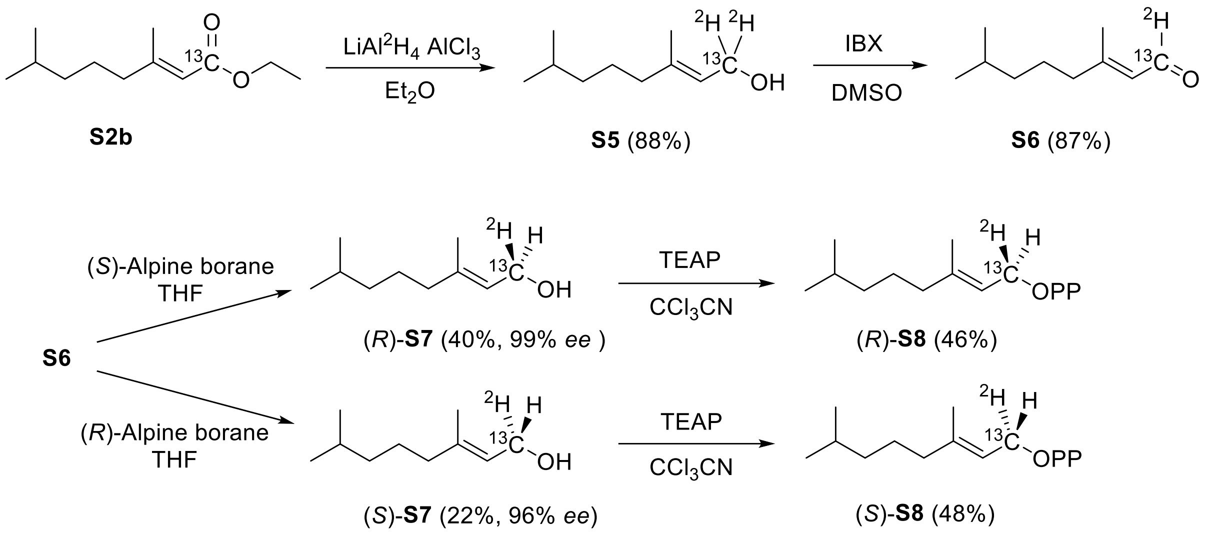

Scheme S2. Synthesis of (*R*)- and (*S*)-(1-^{13}C,1-^{2}H)-6,7-dihydro-GPP.

Synthesis of (1-^{13}C,1,1-$^{2}H_2$)-6,7-dihydrogeraniol (S5)

To a stirred suspension of $LiAl^2H_4$ (420 mg, 10.0 mmol, 2.0 eq.) in Et_2O (50 mL) $AlCl_3$ (465 mg, 3.5 mmol, 0.7 eq.) was added slowly. The mixture was stirred for 30 min and then cooled to 0 °C. (*E*)-(1-^{13}C)-Ethyl 6,7-dihydrogeranate **S2b** (1.0 g, 5.0 mmol, 1.0 eq.) was added dropwise. The mixture was warmed to room temperature and stirred overnight, followed by quenching through the slow addition of H_2O (1 mL), 2 M NaOH (3 mL), and then another portion of H_2O (3 mL). The formed colourless precipitate was removed by filtration under reduced pressure and washed three times with Et_2O. The combined organic layers were dried with $MgSO_4$ and concentrated under reduced pressure. The residue was purified by repeated column chromatography (pentane/diethyl ether = 7/3) to yield **S5** (0.70 g, 4.40 mmol, 88%) as a colourless oil. **S5.** TLC (pentane/diethyl ether = 7/3): R_f = 0.30. HR-MS (APCI): calc. for $[C_9{}^{13}C_1H_{19}D_2O]^+$ *m*/*z* = 160.1746; found *m*/*z* = 160.1743. IR (diamond ATR): ṽ = 3325 (m), 2955 (s), 2931 (s), 2870 (s), 1466 (m), 1383 (m), 1367 (m), 1068 (m), 950 (m) cm^{-1}. ^{1}H-NMR ($CDCl_3$, 500 MHz): δ = 5.40 (s, 1H, CH), 1.99 (t, $^{3}J_{H,H}$ = 7.7 Hz, 2H, CH_2), 1.67 (d, $^{4}J_{H,H}$ = 1.4 Hz, 3H, CH_3), 1.54 (non, $^{3}J_{H,H}$ = 6.7 Hz, 1H, CH), 1.41 (m, 2H, CH_2), 1.15 (m, 2H, CH_2), 0.87 (d, $^{3}J_{H,H}$ = 6.7 Hz, 6H, 2x CH_3) ppm. ^{13}C-NMR ($CDCl_3$, 125 MHz): δ = 140.6 (C_q), 123.1 (d, $^{1}J_{C,C}$ = 47.4 Hz, CH), 58.9 (quint, $^{1}J_{C,D}$ = 21.7 Hz, $^{13}C^2H_2$), 39.9 (d, $^{3}J_{C,C}$ = 4.8 Hz, CH_2), 38.8 (CH_2), 28.0 (CH), 25.6 (CH_2), 22.8 (2x CH_3), 16.3 (d, $^{3}J_{C,C}$ = 4.3 Hz, CH_3) ppm. EI-MS (70 eV): *m*/*z* (%) = 159 (0.5), 141 (5), 126 (7), 112(1), 109 (3), 98 (11), 84 (20), 74 (100), 71 (33), 56 (22), 41 (33). GC (HP-5MS): *I* = 1226.

Synthesis of (1-^{13}C,1-^{2}H)-6,7-dihydrogeranial (S6)

IBX (845 mg, 3.0 mmol, 1.2 eq.) was added to DMSO (8 mL) and stirred at room temperature for 15 min to form a clear solution. The alcohol **S5** (400 mg, 2.5 mmol, 1 eq.) was added dropwise. The reaction mixture was stirred for 1 h at room temperature until full consumption of the starting material (monitored by TLC). The reaction mixture was cooled to 0 °C and quenched by the addition saturated $NaHCO_3$ solution (20 mL). The mixture was extracted three times with Et_2O, the combined extracts were dried with $MgSO_4$ and concentrated under reduced pressure. The residue was purified by repeated column chromatography on silica gel with

(pentane/diethyl ether = 9:1) to yield the aldehyde **S6** (350 mg, 2.24 mmol, 87%) as colourless oil.

S6. TLC (pentane/Et_2O = 9:1): R_f = 0.25. HR-MS (APCI): calc. for $[C_9{}^{13}C_1H_{18}D_1O]^+$ *m*/*z* = 157.1527; found *m*/*z* = 157.1523. IR (diamond ATR): ṽ = 2955 (m), 2937 (m), 2870 (m), 1633 (s), 1467 (w), 1384 (w), 1144 (w) cm^{-1}. ^1H-NMR (C_6D_6, 500 MHz): δ = 5.82 (m, 1H, CH), 1.66 (t, $^3J_{H,H}$ = 7.5 Hz, 2H, CH_2), 1.53 (s, 3H, CH_3), 1.35 (non, $^3J_{H,H}$ = 6.7 Hz, 1H, CH), 1.12 (m, 2H, CH_2), 0.92 (m, 2H, CH_2), 0.80 (d, $^3J_{H,H}$ = 6.6 Hz, 6H, 2x CH_3) ppm. ^{13}C-NMR (C_6D_6, 125 MHz): δ = 189.6 (t, $^1J_{C,D}$ = 25.8 Hz, H-^{13}C=O), 161.9 (d, $^2J_{C,C}$ = 2.6 Hz, C_q), 127.4 (dt, $^1J_{C,C}$ = 55.2 Hz, $^2J_{C,D}$ = 3.9 Hz, CH), 40.6 (d, $^3J_{C,C}$ = 5.3 Hz, CH_2), 38.6 (CH_2), 28.1 (CH), 25.1 (CH_2), 22.6 (2x CH_3), 16.8 (d, $^3J_{C,C}$ = 4.8 Hz, CH_3) ppm. EI-MS (70 eV): *m*/*z* (%) = 156 (0.5), 141 (6), 138 (1), 123 (10), 113 (9), 99 (30), 86 (100), 84 (21), 69 (16), 55 (25), 43 (36), 41 (44), 39 (24). GC (HP-5MS): *I* = 1250.

Synthesis of (*R*)- and (*S*)-(1-^{13}C,1-^{2}H)-6,7-dihydrogeraniol ((*R*)-/(*S*)-S7)

The aldehyde **S6** (1 eq.) was added to a cooled (0 °C) and stirred solution of (*S*)- or (*R*)-Alpine borane (0.5 M in THF, 1.25 eq.). The reaction mixture was stirred for 6 h at room temperature, followed by the addition of freshly distilled acetaldehyde (50 µL $mmol^{-1}$). The reaction mixture was subjected to high vacuum for 1 h and the residue was dissolved in Et_2O (2 mL $mmol^{-1}$). The resulting solution was cooled to 0 °C. After addition of ethanolamine (1.1 eq.), stirring was continued for 10 min until a white precipitate was formed. The precipitate was filtered off and washed three times with Et_2O. The combined organic layers were washed with H_2O and brine, followed by drying with $MgSO_4$. The solvent was removed under reduced pressure and the residue was purified by repeated column chromatography on silica gel with (pentane/diethyl ether = 1/1) to yield the alcohols (*R*)-**S7** and (*S*)-**S7** as colourless oils. The enantiomeric purity was checked by conversion of a small sample (ca. 0.5 mg) into the Mosher ester with (*S*)-(+)-Mosher chloride (1 mg) and pyridine (1 µL) in $CDCl_3$ (100 µL). After a reaction time of 1 h, $CDCl_3$ (400 µL) was added and the sample was directly analysed by ^1H-NMR (Figure S58).

(*R*)-S7. Yield: 60 mg (0.38 mmol, 40%, 99% *ee*). TLC (pentane/diethyl ether = 7/3): R_f = 0.30. HR-MS (APCI): calc. for $[C_9{}^{13}C_1H_{20}D_1O]^+$ *m*/*z* = 159.1683; found *m*/*z* = 159.1678. IR (diamond ATR): ṽ = 3328 (m), 2954 (s), 2931 (s), 2870 (s), 1467 (m), 1384 (m), 1366 (m), 1010 (m), 983 (m) cm^{-1}. ^1H-NMR ($CDCl_3$, 700 MHz): δ = 5.40 (s, 1H, CH), 1.99 (t, $^3J_{H,H}$ = 7.7 Hz, 2H, CH_2), 1.67 (d, $^4J_{H,H}$ = 1.4 Hz, 3H, CH_3), 1.54 (non, $^3J_{H,H}$ = 6.7 Hz, 1H, CH), 1.41 (m, 2H, CH_2), 1.15 (m, 2H, CH_2), 0.87 (d, $^3J_{H,H}$ = 6.7 Hz, 6H, 2x CH_3) ppm. ^{13}C-NMR ($CDCl_3$, 175 MHz): δ = 140.5 (C_q), 123.2 (d, $^1J_{C,C}$ = 47.4 Hz, CH), 59.2 (t, $^1J_{C,D}$ = 21.6 Hz, $^{13}C^2H^1H$), 39.9 (d, $^3J_{C,C}$ = 4.8 Hz, CH_2), 38.8 (CH_2), 28.0 (CH), 25.6 (CH_2), 22.8 (2x CH_3), 16.3 (d, $^3J_{C,C}$ = 4.3 Hz, CH_3) ppm. EI-MS (70 eV): *m*/*z* (%) = 158 (0.4), 140 (5), 125 (6), 109 (3), 97 (13), 83 (21), 73 (100), 69 (32), 55 (21), 41 (36). GC (HP-5MS): *I* = 1230.

(*S*)-S7. Yield: 45 mg (0.28 mmol, 22%, 96% *ee*). HR-MS (APCI): calc. for $[C_9{}^{13}C_1H_{20}D_1O]^+$ *m*/*z* = 159.1683; found *m*/*z* = 159.1681. Spectroscopic data were the same as for (*R*)-**S7**.

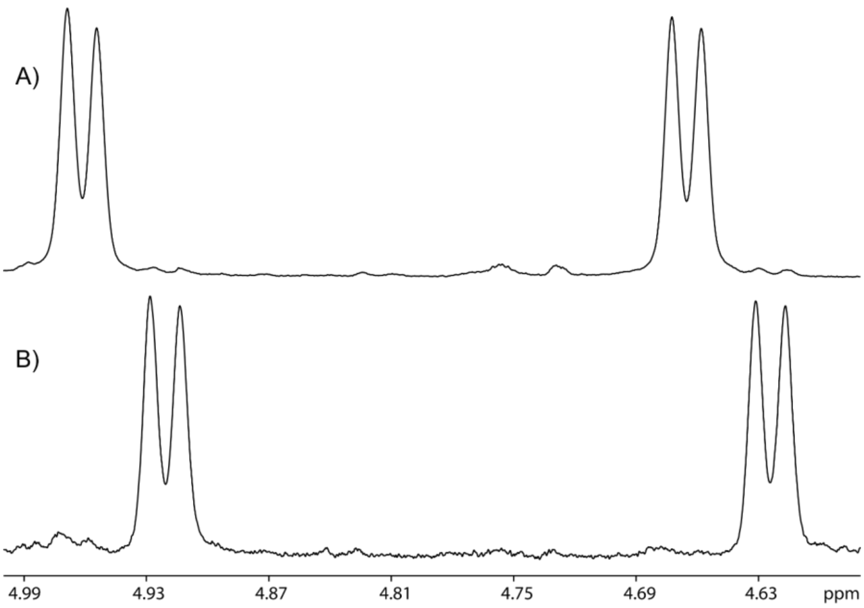

Figure S58. A) Partial ^{1}H-NMR spectra of the Mosher ester prepared from (*R*)-**S7** and (*S*)-Mosher chloride, B) partial ^{1}H-NMR spectra of the Mosher ester prepared from (*S*)-**S7** and (*S*)-Mosher chloride.

Synthesis of (*R*)- and (*S*)-(1-^{13}C,1-^{2}H)-6,7-dihydro-GPP trisammonium salt

Alcohols (*S*)- and (*R*)-**S7** (1 eq.) were dissolved in Cl_3CCN (2.5 mL mmol^{-1}) and a solution of bis-triethylammonium phosphate (TEAP; prepared by adding 3.64 mL of a solution of H_3PO_4 (2.5 mL) in MeCN (9.4 mL) to a mixture of NEt_3 (11 mL) and MeCN (10 mL); 2.5 mL mmol^{-1}) was added dropwise to the stirred solution within 1 min. After intervals of 4 mins stirring at room temperature, two more additions of the same amount of TEAP solution were performed before directly subjecting the reaction mixture to silica gel column chromatography [^{i}PrOH/25% NH_3 H_2O/H_2O (6:2.5:0.5)] to yield the title compounds as white solids after lyophilisation of collected fractions.

(*R*)-S8. Yield: 75 mg (0.20 mmol, 46%). HR-MS (ESI–): [M–H]$^-$ calc. for [$C_9{}^{13}C_1H_{20}D_1O_7P_2$]$^-$ *m*/*z* = 317.0864; found *m*/*z* = 317.0870. ^{1}H-NMR (D_2O, 500 MHz): δ = 5.46 (d, $^{3}J_{H,H}$ = 6.6 Hz, 1H, CH), 4.47 (dt, $^{1}J_{C,H}$ = 146.2 Hz, $^{1}J_{C,D}$ = 6.7 Hz, 1H, ^{2}H-^{13}C-^{1}H), 2.06 (t, $^{3}J_{H,H}$ = 7.6 Hz, 2H, CH_2), 1.72 (s, 3H, CH_3), 1.55 (hept, $^{3}J_{H,H}$ = 6.7 Hz, 1H, CH), 1.45 (m, 2H, CH_2), 1.17 (m, 2H, CH_2), 0.87 (d, $^{3}J_{H,H}$ = 6.7 Hz, 6H, 2x CH_3) ppm. ^{13}C-NMR (D_2O, 125 MHz): δ = 144.1 (1C, C_q), 119.1 (dd, $^{1}J_{C,C}$ = 49.8, $^{3}J_{C,P}$ = 8.3 Hz, 1C, CH), 62.6 (td, $^{1}J_{C,D}$ = 22.4, $^{2}J_{C,P}$ = 5.0 Hz, 1C, ^{2}H-^{13}C-^{1}H), 39.2 (d, $^{3}J_{C,C}$ = 4.8 Hz, 1C, CH_2), 38.0 (s, 1C, CH_2), 27.2 (s, 1C, CH), 24.8 (s, 1C, CH_2), 22.0 (s, 2C, 2x CH_3), 15.6 (d, $^{3}J_{C,C}$ = 4.2 Hz, 1C, CH_3) ppm. ^{31}P-NMR (D_2O, 203 MHz): δ = –9.39 (d, $^{2}J_{P,P}$ = 21.0 Hz, 1P), –10.52 (dd, $^{2}J_{P,P}$ = 21.0, $^{2}J_{C,P}$ = 5.2 Hz, 1P) ppm.

(*S*)-S8. Yield: 50 mg (0.13 mmol, 48%). HR-MS (ESI–): [M–H]$^-$ calc. for [$C_9{}^{13}C_1H_{20}D_1O_7P_2$]$^-$ *m*/*z* = 317.0864; found *m*/*z* = 317.0866. Spectroscopic data were the same as for (*R*)-**S8**.

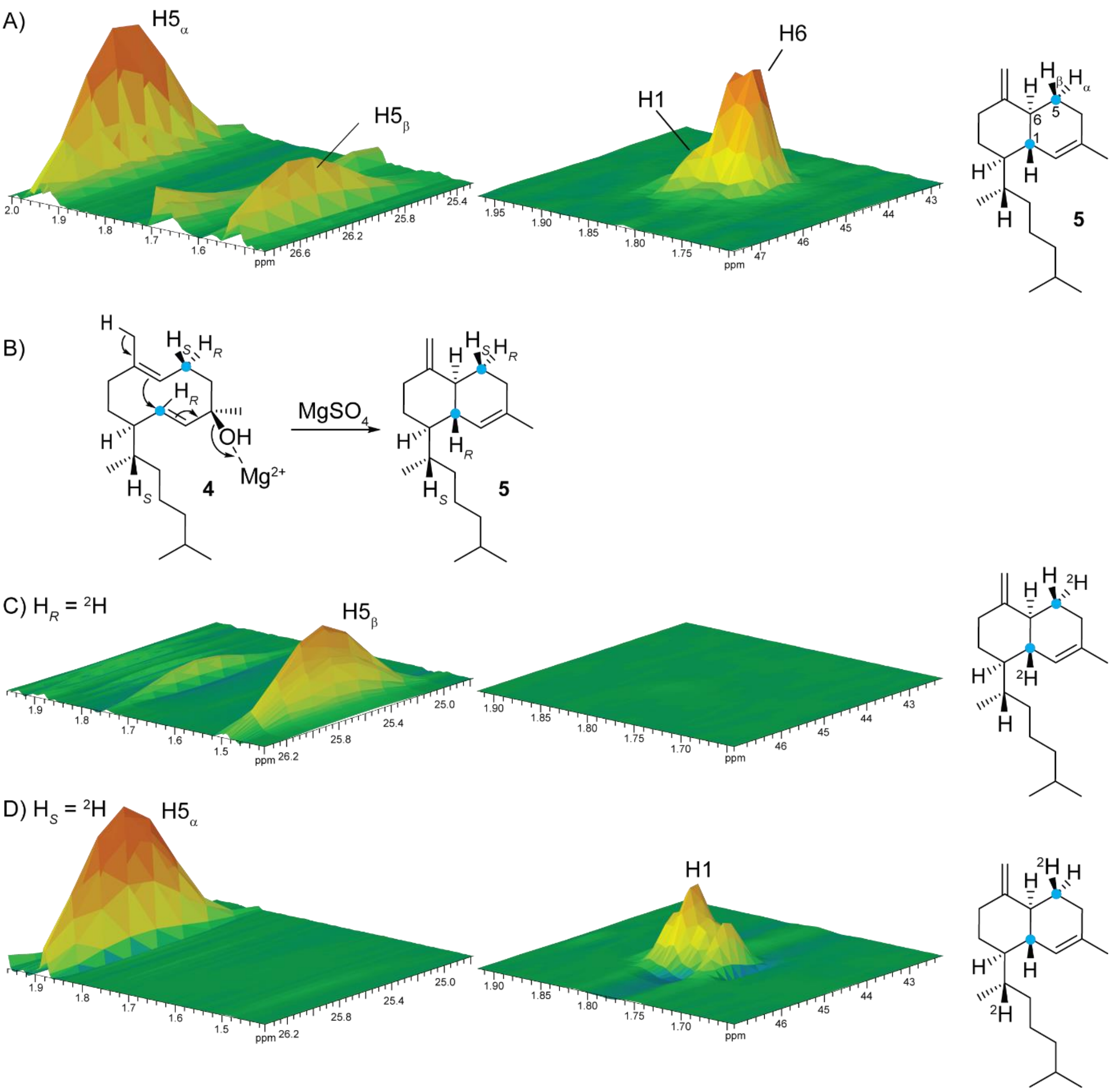

Figure S59. Determination of the absolute configuration of **5** using the substrates (*R*)- and (*S*)-(1-^{13}C,1-^{2}H)IPP and 6,7-dihydro-GPP. A) HMQC spectrum of unlabelled **5**. B) $MgSO_4$ catalysed conversion of **4** into **5**. C) HSQC spectrum of labelled **5** obtained from (*R*)-(1-^{13}C,1-^{2}H)IPP and 6,7-dihydro-GPP. D) HSQC spectrum of labelled **5** obtained from (*S*)-(1-^{13}C,1-^{2}H)IPP and 6,7-dihydro-GPP. In both labelling experiments the signals for the CH-correlations of the labelled carbons are strongly enhanced, but the signal for one of the diastereotopic hydrogens is vanished because of the substitution with deuterium.

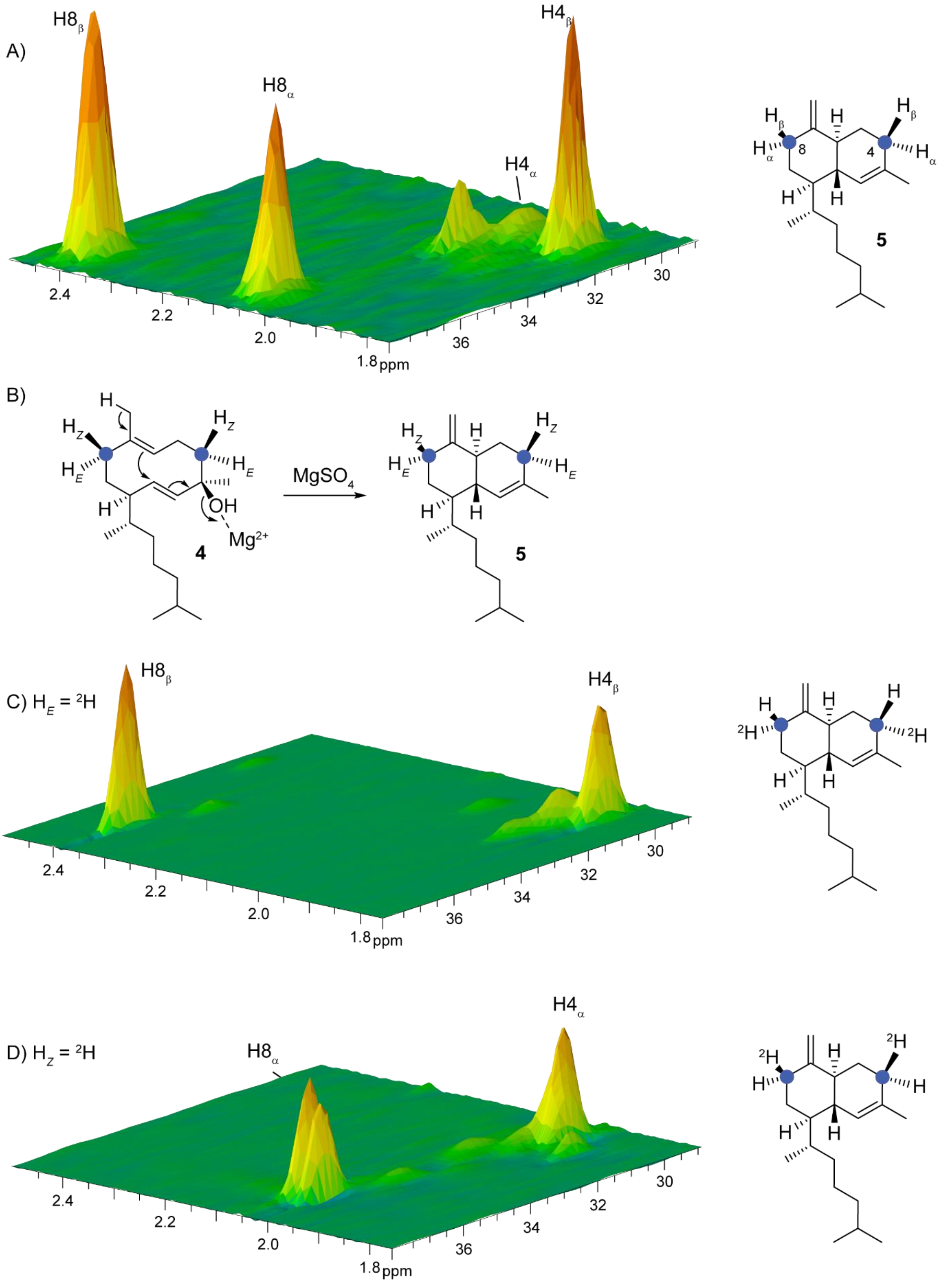

Figure S60. Determination of the absolute configuration of **5** using the substrates (*E*)- and (*Z*)-(4-^{13}C,4-^{2}H)IPP. A) HMQC spectrum of unlabelled **5**. B) $MgSO_4$ catalysed conversion of **4** into **5**. C) HSQC spectrum of labelled **5** obtained from (*E*)-(4-^{13}C,4-^{2}H)IPP. D) HSQC spectrum of labelled **5** obtained from (*Z*)-(4-^{13}C,4-^{2}H)IPP. In both labelling experiments the signals for the CH-correlations of the labelled carbons are strongly enhanced, but the signal for one of the diastereotopic hydrogens is vanished because of the substitution with deuterium.

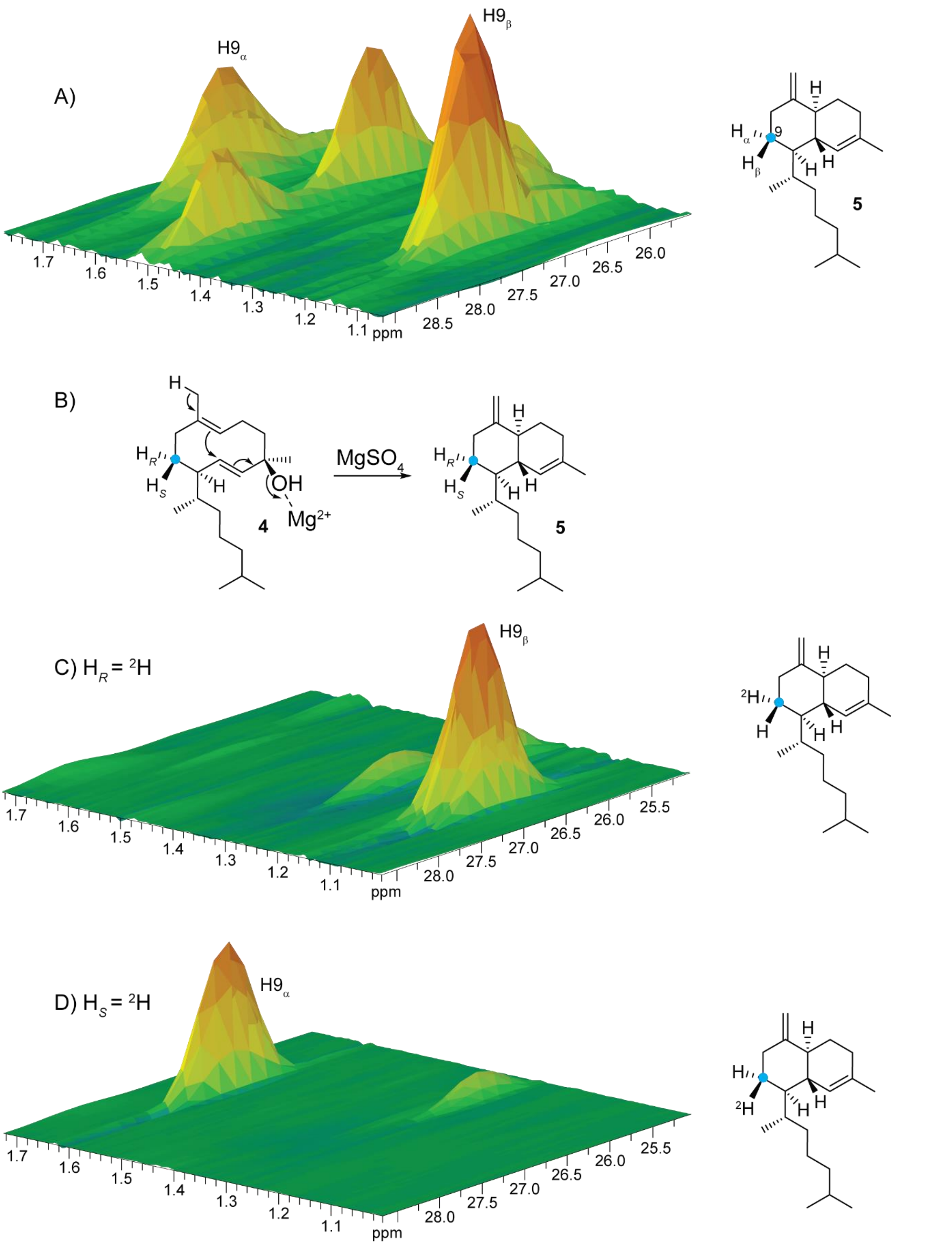

Figure S61. Determination of the absolute configuration of **5** using the substrates (*R*)- and (*S*)-(1-^{13}C,1-^{2}H)-6,7-dihydro-GPP and IPP. A) HMQC spectrum of unlabelled **5**. B) $MgSO_4$ catalysed conversion of **4** into **5**. C) HSQC spectrum of labelled **5** obtained from (*R*)-(1-^{13}C,1-^{2}H)-6,7-dihydro-GPP and IPP. D) HSQC spectrum of labelled **5** obtained from (*S*)-(1-^{13}C,1-^{2}H)-6,7-dihydro-GPP and IPP. In both labelling experiments the signals for the CH-correlations of the labelled carbons are strongly enhanced, but the signal for one of the diastereotopic hydrogens is vanished because of the substitution with deuterium.

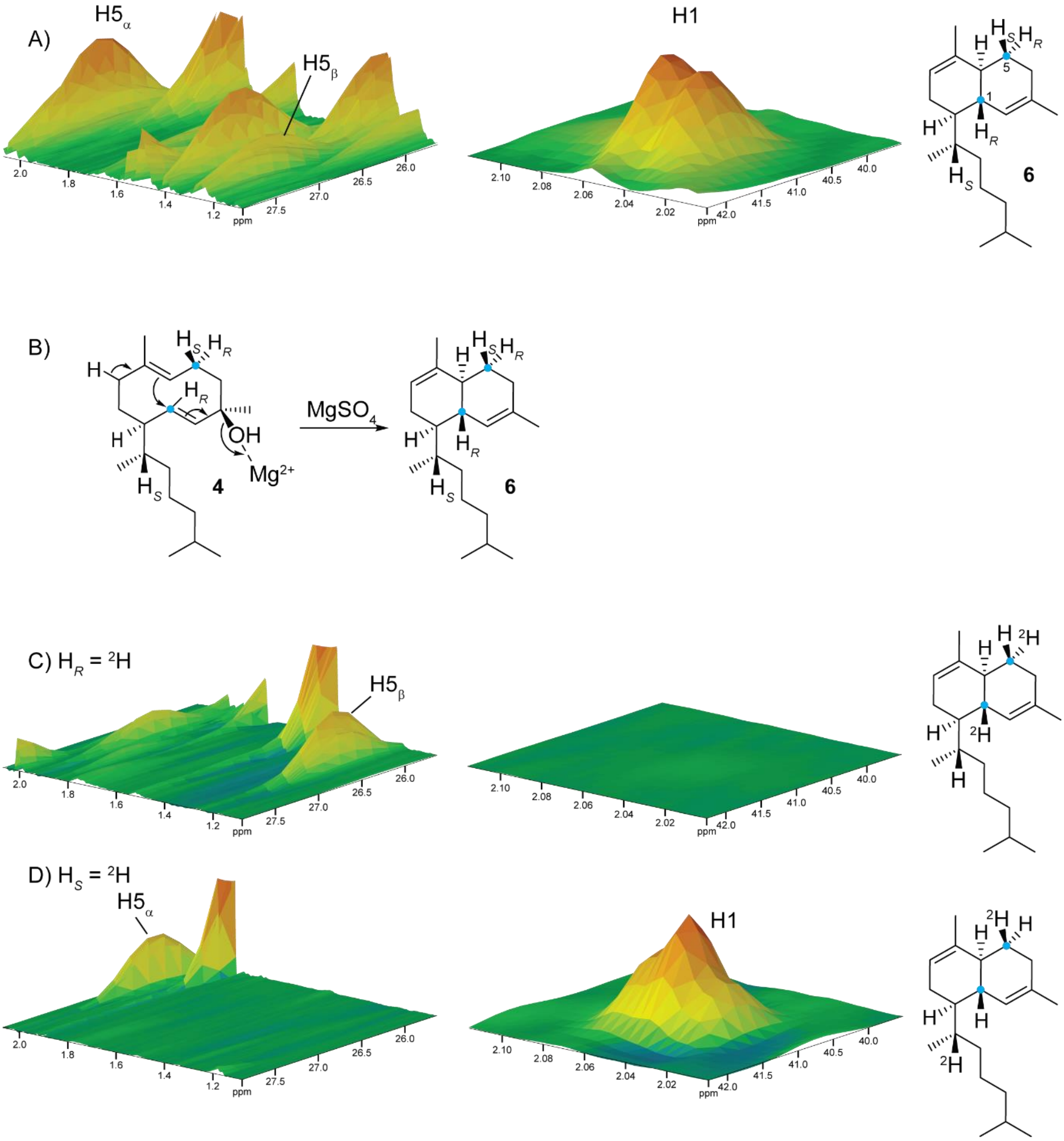

Figure S62. Determination of the absolute configuration of **6** using the substrates (*R*)- and (*S*)-(1-^{13}C,1-^{2}H)IPP and 6,7-dihydro-GPP. A) HSQC spectrum of unlabelled **6**. B) $MgSO_4$ catalysed conversion of **4** into **6**. C) HSQC spectrum of labelled **6** obtained from (*R*)-(1-^{13}C,1-^{2}H)IPP and 6,7-dihydro-GPP. D) HSQC spectrum of labelled **6** obtained from (*S*)-(1-^{13}C,1-^{2}H)IPP and 6,7-dihydro-GPP. In both labelling experiments the signals for the CH-correlations of the labelled carbons are strongly enhanced, but the signal for one of the diastereotopic hydrogens is vanished because of the substitution with deuterium.

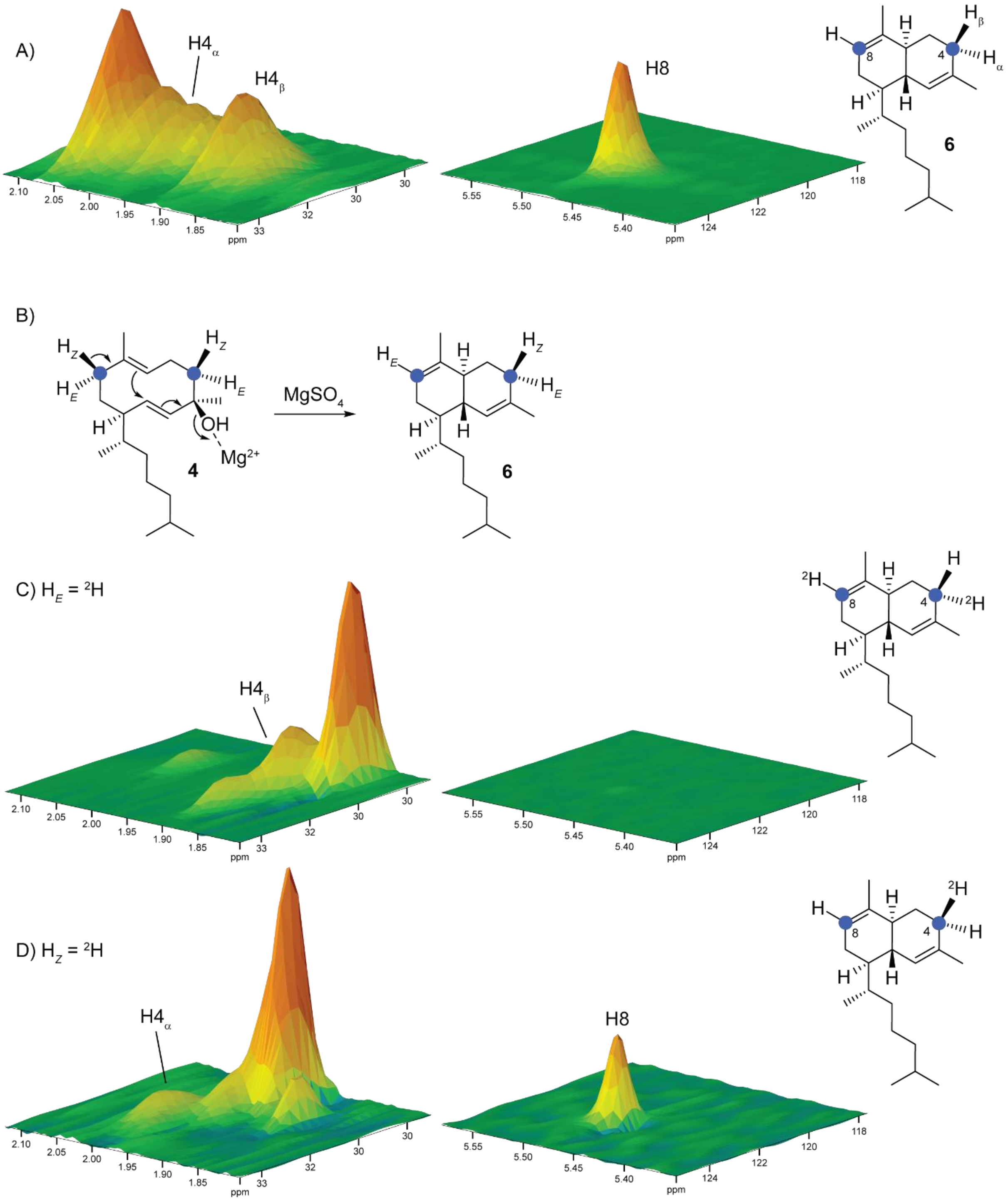

Figure S63. Determination of the absolute configuration of **6** using the substrates (*E*)- and (*Z*)- (4-^{13}C,4-^{2}H)IPP. A) HSQC spectrum of unlabelled **6**. B) $MgSO_4$ catalysed conversion of **4** into **6**. C) HSQC spectrum of labelled **6** obtained from (*E*)-(4-^{13}C,4-^{2}H)IPP. D) HSQC spectrum of labelled **6** obtained from (*Z*)-(4-^{13}C,4-^{2}H)IPP. In both labelling experiments the signals for the CH-correlations of the labelled carbons are strongly enhanced, but the signal for one of the diastereotopic hydrogens is vanished because of the substitution with deuterium.

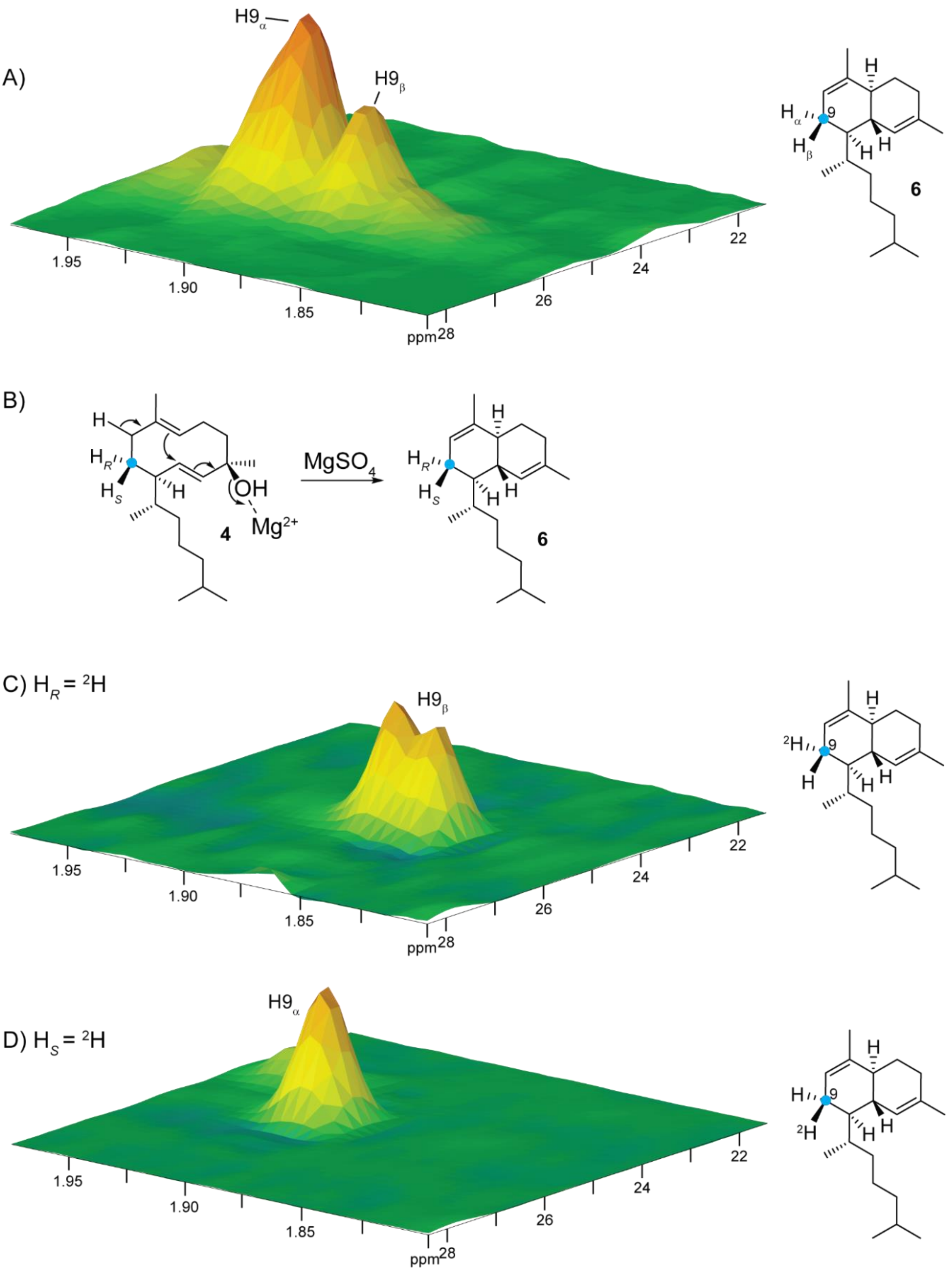

Figure S64. Determination of the absolute configuration of **6** using the substrates (*R*)- and (*S*)-(1-^{13}C,1-^{2}H)-6,7-dihydro-GPP and IPP. A) HSQC spectrum of unlabelled **6**. B) $MgSO_4$ catalysed conversion of **4** into **6**. C) HSQC spectrum of labelled **6** obtained from (*R*)-(1-^{13}C,1-^{2}H)-6,7-dihydro-GPP and IPP. D) HSQC spectrum of labelled **6** obtained from (*S*)-(1-^{13}C,1-^{2}H)-6,7-dihydro-GPP and IPP. In both labelling experiments the signals for the CH-correlations of the labelled carbons are strongly enhanced, but the signal for one of the diastereotopic hydrogens is vanished because of the substitution with deuterium.

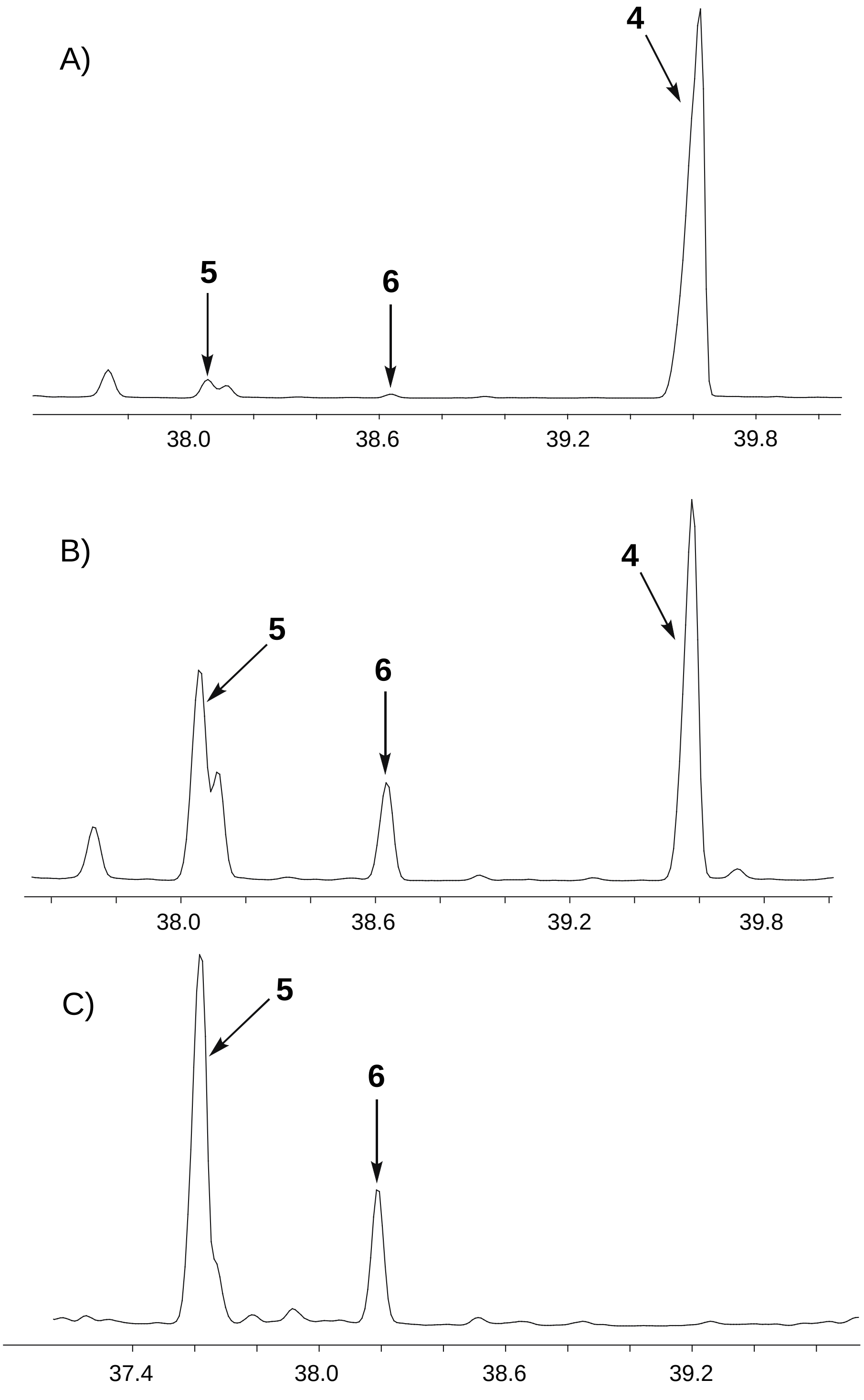

Figure S65. Total ion chromatogram of the products obtained from 14,15-dihydro-GGPP with CaCS A) without drying, B) after drying with a small amount of $MgSO_4$, and C) after drying with an excess of $MgSO_4$.

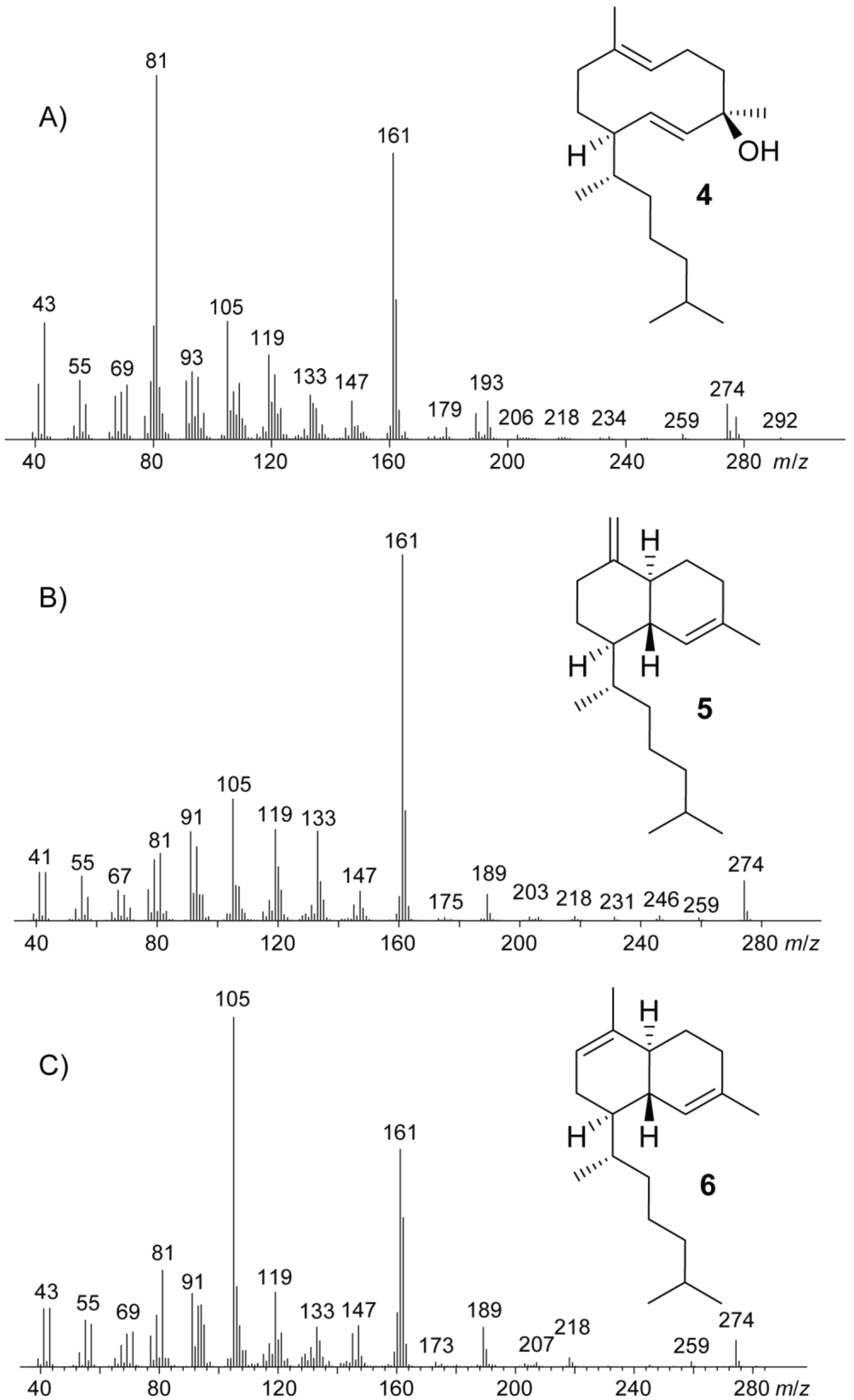

Figure S66. EI mass spectra of A) compound **4** and its degradation products B) **5** and C) **6**.

Table S9. NMR data of **4** in C_6D_6 recorded at 298 K.

C[a]	type	^{1}H[b]	^{13}C[b]
1	CH	5.34 (dd, *J* = 15.6, 10.0)	126.6
2	C_q	5.07 (d, *J* = 15.6)	140.3
3	C_q	–	72.7
4	CH_2	1.44 (m, H_β)	40.0
		1.29 (m, H_α)	
5	CH_2	2.62 (m, H_β)	24.2
		1.90 (m, H_α)	
6	CH	4.94 (d, *J* = 11.6)	129.3
7	CH	–	132.5
8	CH_2	2.27 (m, H_β)	41.8
		2.27 (m, H_α)	
9	CH_2	1.35 (m, H_β)	25.8
		1.31 (m, H_α)	
10	CH	2.06 (m)	52.3
11	CH	1.31 (m)	38.8
12	CH_2	1.45 (m)	33.9
		1.06 (m)	
13	CH_2	1.40 (m)	25.5
		1.22 (m)	
14	C_q	1.21 (m)	39.8
		1.16 (m)	
15	C_q	1.53 (m)	28.4
16	CH_3	0.92 (d, *J* = 6.6)	22.9
17	CH_3	0.92 (d, *J* = 6.6)	23.0
18	CH_3	0.93 (d, *J* = 6.8)	17.5
19	CH_3	1.56 (s)	16.9
20	CH_3	1.08 (s)	31.3

[a] Carbon numbering as shown in main text. [b] Chemical shifts δ in ppm, multiplicity: s = singlet, d = doublet, m = multiplet, coupling constants *J* are given in Hertz.

14,15-Dihydroobscuronatin (4). Yield: 4.0 mg (14.6 μmol, 14%), from 80 mg (220 μmol) 6,7-dihydro-GPP trisammonium salt and 60 mg (204 μmol) IPP trisammonium salt. TLC (pentane/Et_2O = 3/1): R_f = 0.74. IR (diamond ATR): ṽ = 3446 (w), 2954 (m), 2924 (s), 2853 (m), 1458 (m), 1376 (w), 1262 (w), 1096 (m), 1015 (w), 980 (w), 801 (w) cm^{-1}. HR-MS (Q-TOF, 70 eV): calc. for $[C_{20}H_{36}O]^+$ $[M-H_2O]^+$ *m*/*z* = 274.2655, found: *m*/*z* = 274.2652. Optical rotary power: $[\alpha]_D^{20}$ = +59.0 (*c* 0.30, C_6H_6).

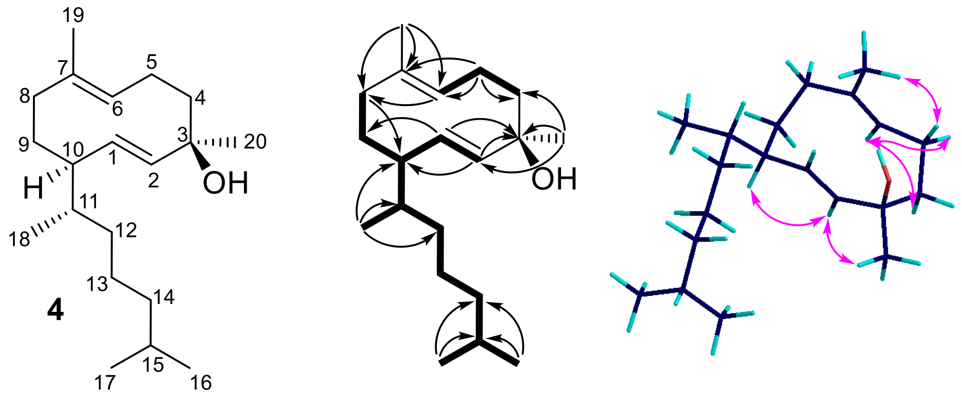

Figure S67. Carbon numbering and structure elucidation of **4**. Bold lines represent $^{1}H,^{1}H$-COSY correlations, selected HMBC signals are represented by single-headed arrows and selected NOE correlations are depicted by double headed arrows.

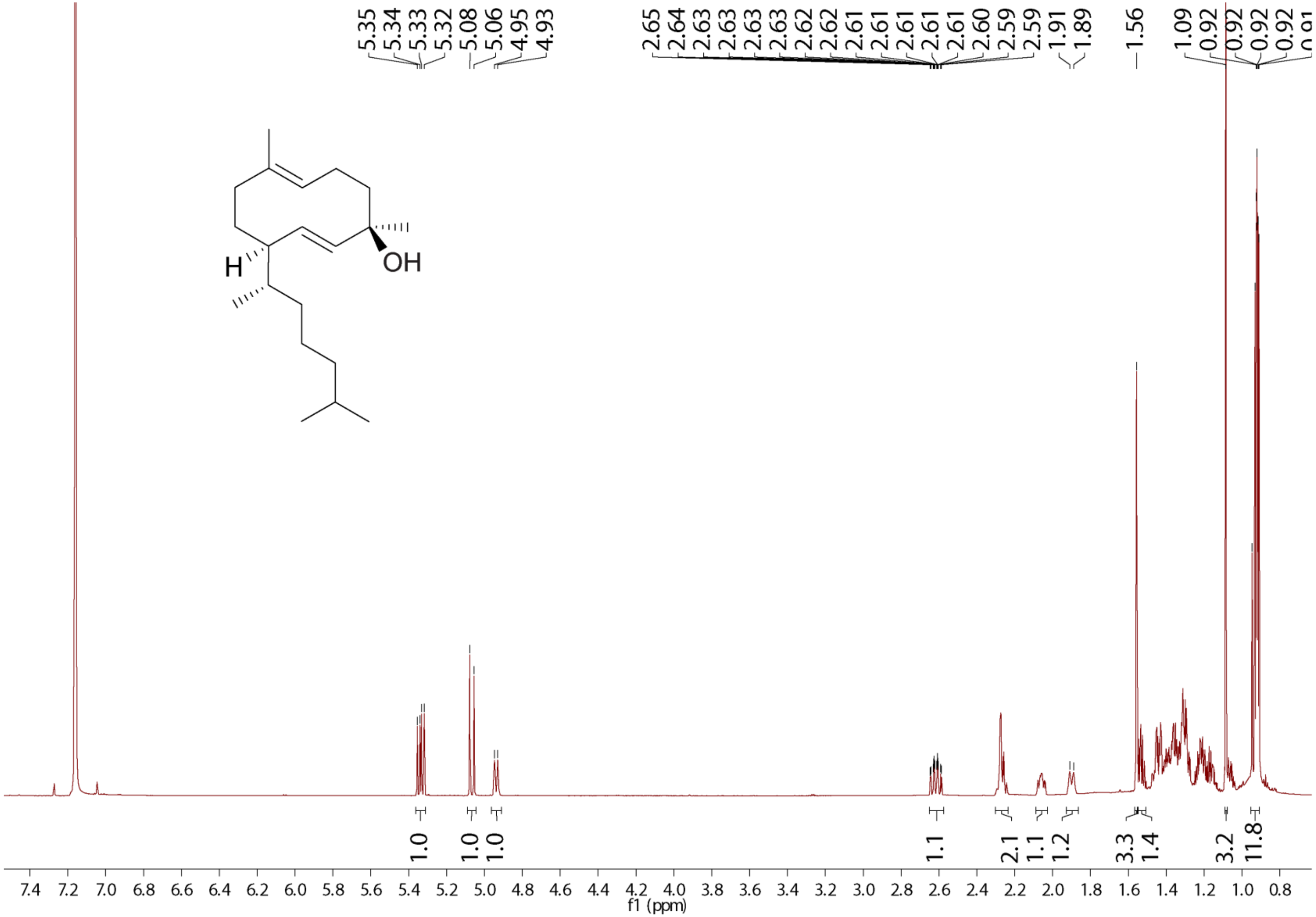

Figure S68. ^{1}H-NMR spectrum of **4** (700 MHz, C_6D_6).

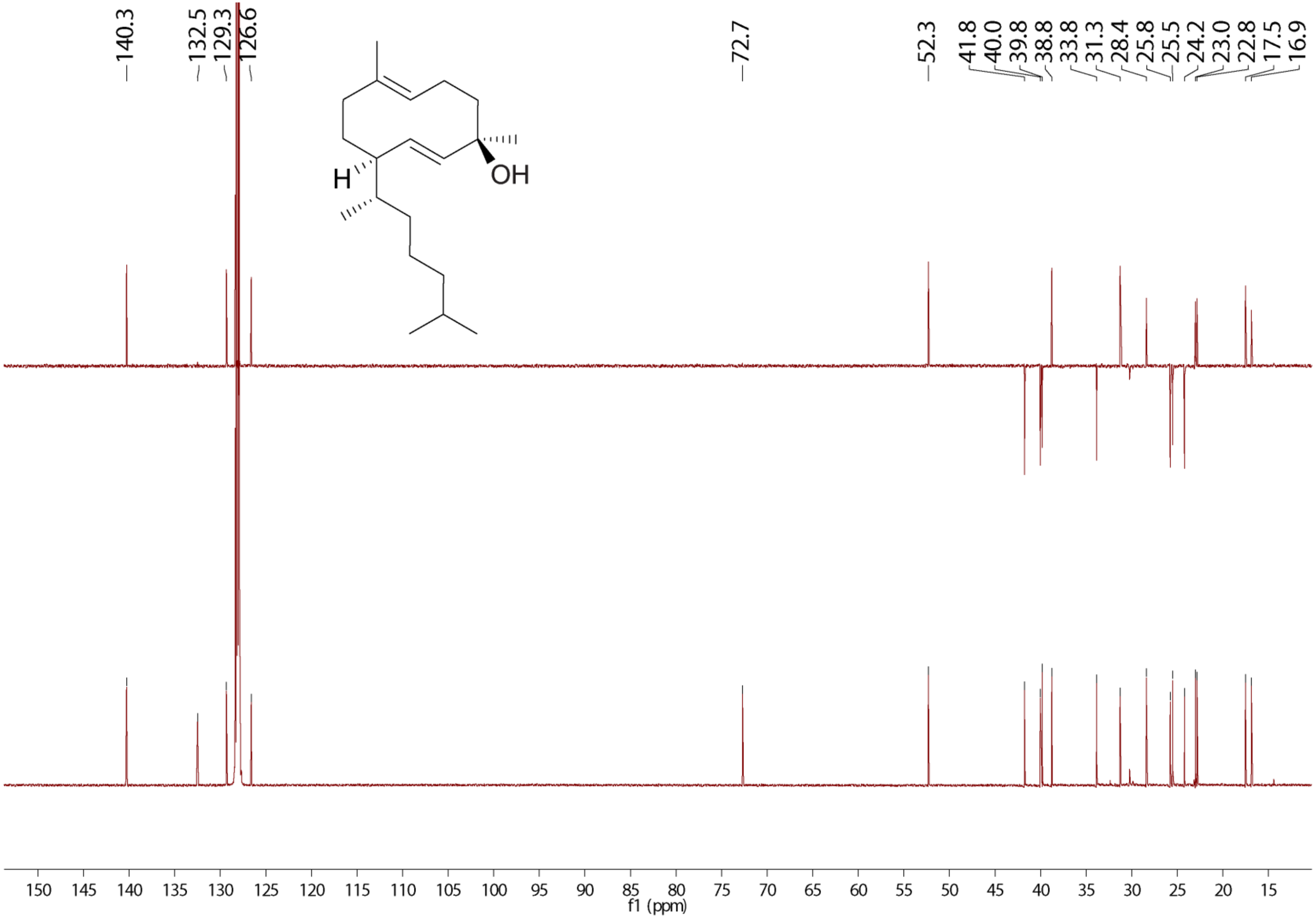

Figure S69. ^{13}C-NMR and ^{13}C-DEPT-135 spectra of **4** (175 MHz, C_6D_6).

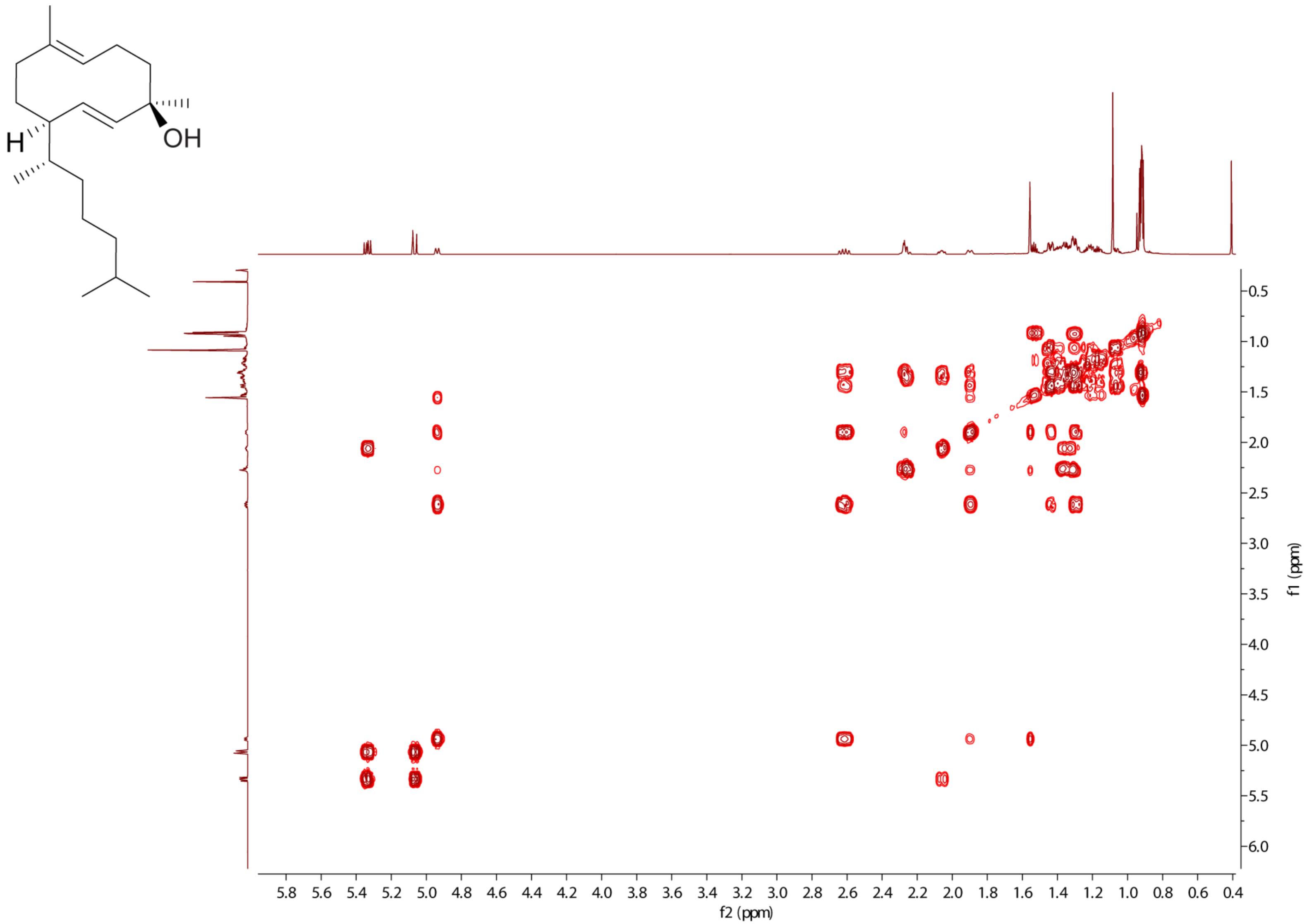

Figure S70. $^{1}H,^{1}H$ COSY spectrum of **4** (C_6D_6).

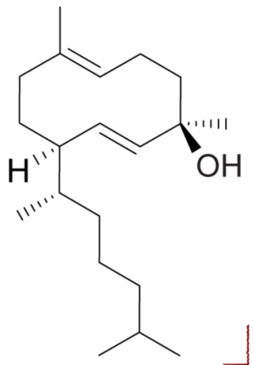

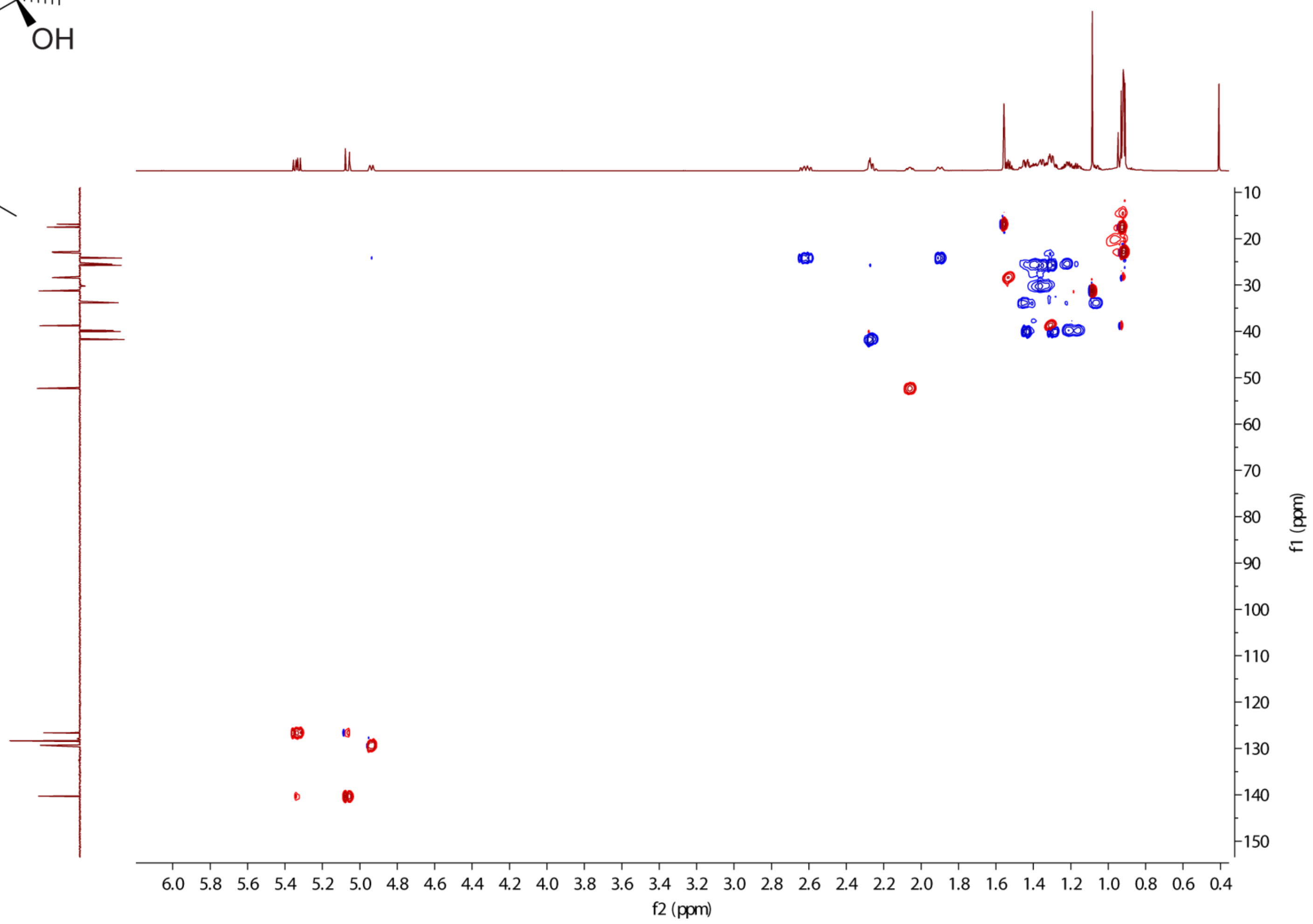

Figure S71. HSQC spectrum of **4** (C_6D_6).

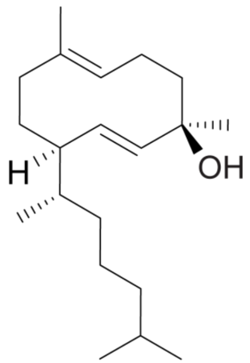

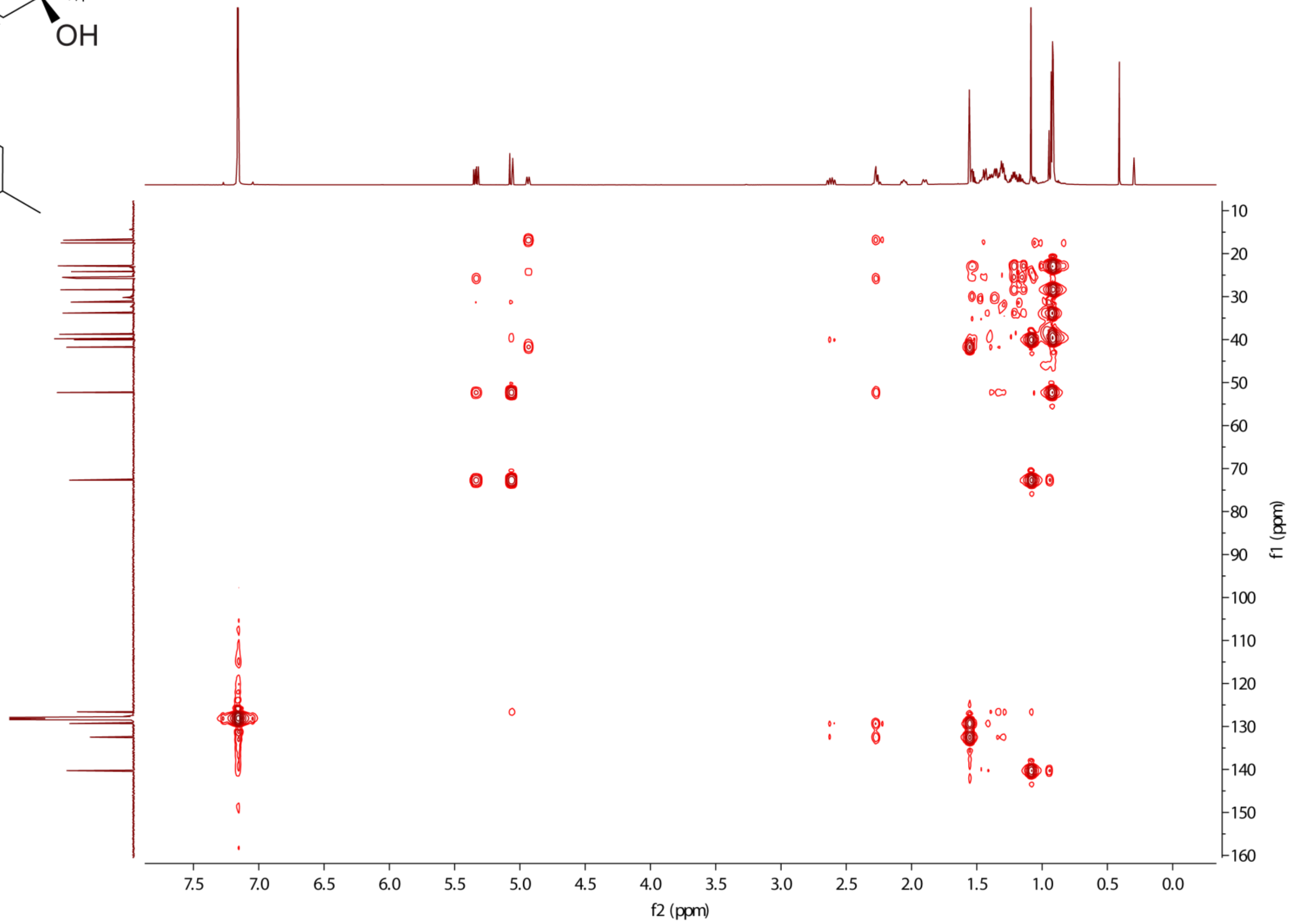

Figure S72. HMBC spectrum of **4** (C_6D_6).

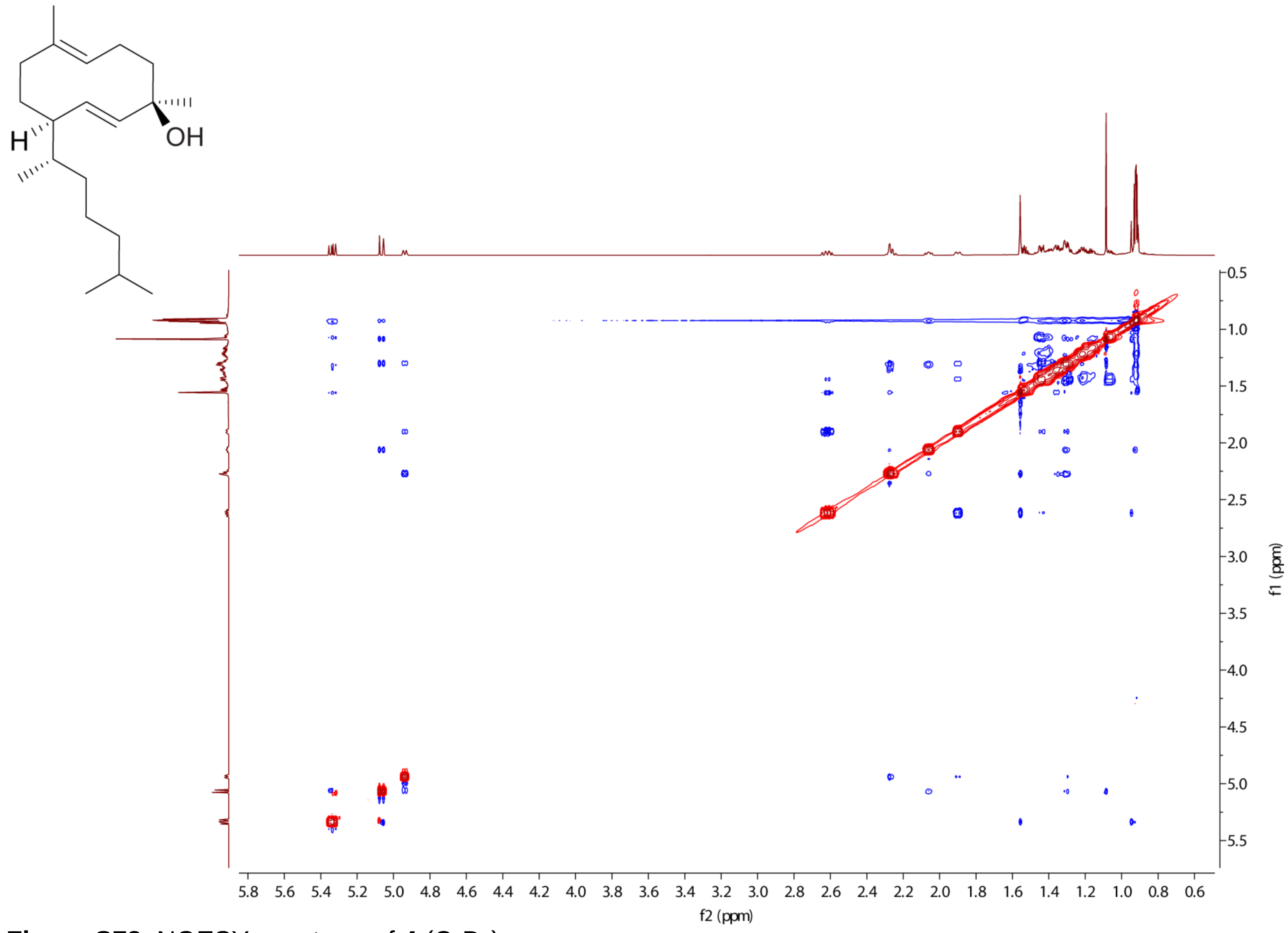

Figure S73. NOESY spectrum of **4** (C_6D_6).

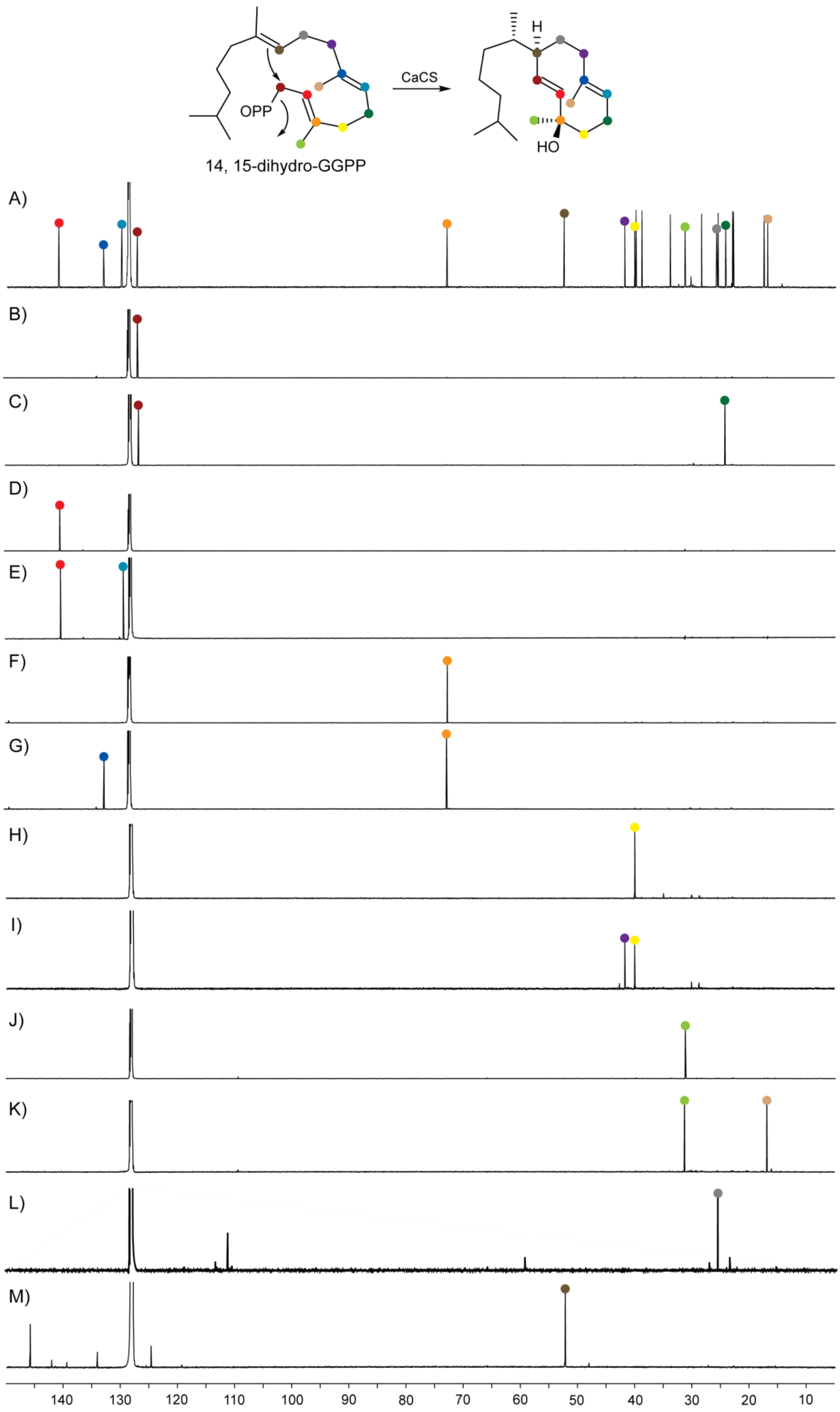

Figure S74. ^{13}C-NMR spectra of A) unlabelled **4**, and of ^{13}C-labelled **4** obtained from the seven isotopomers of (^{13}C)-14,15-dihydro-GGPP labelled at carbons B) C1, D) C2, F) C3, H) C4, J) C19, L) C9, and M) C10. ^{13}C-NMR spectra of ^{13}C-labelled **4** obtained from the five isotopomers of ($^{13}C_2$)-14,15-dihydro-GGPP labelled at C) C1 and C5, E) C2 and C6, G) C3 and C7, I) C4 and C8, and K) C19 and C20. Coloured dots correlate the observed ^{13}C signals to the carbons of **4**.

Derivatisation of 4 with NBS

N-Bromosuccinimide (NBS, 1.2 mg, 6.8 μmol, 1.0 eq) was added in one portion to a stirred solution of **4** (2.0 mg, 6.8 μmol, 1.0 eq.) in dry CH_2Cl_2 (1 mL) at –78 °C. After stirring for 10 min at –78 °C, the reaction was allowed to warm to room temperature and stirring was continued for 2 h. Another portion of NBS (1.0 eq) was added and the reaction was further stirred at room temperature for 20 min, before saturated NH_4Cl solution (2 mL) was added. The mixture was extracted with Et_2O (3x 3 mL). The combined extracts were collected and dried with $MgSO_4$ and concentrated under reduced pressure. Purification by column chromatography on silica gel [pentane/Et_2O (4:1)] yielded bromide **7** (0.6 mg, 1.6 μmol, 24%) as colorless oil.

(1*S*,1a*S*,1b*S*,2*R*,5*S*,5a*S*,6a*S*)-5-Bromo-2,5a-dimethyl-1-((*S*)-6-methylheptan-2-yl) decahydrocyclopropa[*a*]inden-2-ol (7). TLC [pentane/Et_2O (2:1)]: R_f = 0.67. IR (diamond ATR): ṽ = 3461 (w), 2954 (s), 2929 (s), 2855 (m), 1670 (w), 1460 (m), 1376 (m), 1192 (w), 1166 (w), 1110 (w), 949 (w), 791 (w), 713 (m) cm^{-1}. EI-MS (70 eV): *m*/*z* (%) = 357/355 (4) [M^+ - CH_3], 354/352(1) [M^+ - H_2O], 341 (2), 291 (2), 281 (4), 273 (19) [M-Br-H_2O]$^+$, 232 (5), 228 (4), 207 (8), 187 (9), 177 (3), 159 (11), 147 (52), 131 (21), 119 (22),. 105 (40), 91 (27), 79 (20), 71 (21), 55 (23), 43 (100). GC (HP-5MS): *I* = 2372. HR-MS (APCI): calc. for [$C_{20}H_{34}{}^{79}Br$]$^+$ [M+H-H_2O]$^+$ *m*/*z* = 353.1839; found *m*/*z* = 353.1838. Optical rotary power: $[\alpha]_D^{20}$ = +8.0 (*c* 0.05, C_6H_6).

Table S10. NMR data of **7** in C_6D_6 recorded at 298 K.

C[a]	type	^1H[b]	^{13}C[b]
1	CH	1.03 (ddd, *J* = 8.3, 5.6, 2.9)	26.4
2	CH	0.41 (m)	62.1
3	C_q		70.5
4	CH_2	1.24 (m, H_β)	41.8
		0.87 (m, H_α)	
5	CH_2	2.31 (dddd, *J* = 13.5, 13.5, 12.5, 4.5, H_β)	31.6
		1.81 (dddd, *J* = 13.5, 4.8, 4.1, 2.4, H_α)	
6	CH	3.61 (dd, *J* = 12.4, 4.0)	62.0
7	C_q		58.5
8	CH_2	1.90 (dd, *J* = 12.4, 6.9, H_β)	46.3
		0.82 (br dd, *J* = 12.4, 4.4, H_α)	
9	CH	0.89 (m)	22.9
10	CH	0.29 (m)	48.6
11	CH	0.58 (m)	37.8
12	CH_2	1.36 (m)	37.7
		1.23 (m)	
13	CH_2	1.33 (m)	25.5
		1.33 (m)	
14	CH_2	1.19 (m)	40.0
		1.19 (m)	
15	CH	1.55 (m)	28.5
16	CH_3	0.93 (d, *J* = 6.6)	22.9
17	CH_3	0.93 (d, *J* = 6.6)	22.9
18	CH_3	0.96 (d, *J* = 6.7)	19.9
19	CH_3	1.50 (d, *J* = 1.0)	18.1
20	CH_3	1.00 (s)	30.7

[a] Carbon numbering as shown in main text. [b] Chemical shifts δ in ppm, multiplicity: s = singlet, d = doublet, m = multiplet, br = broad, coupling constants *J* are given in Hertz.

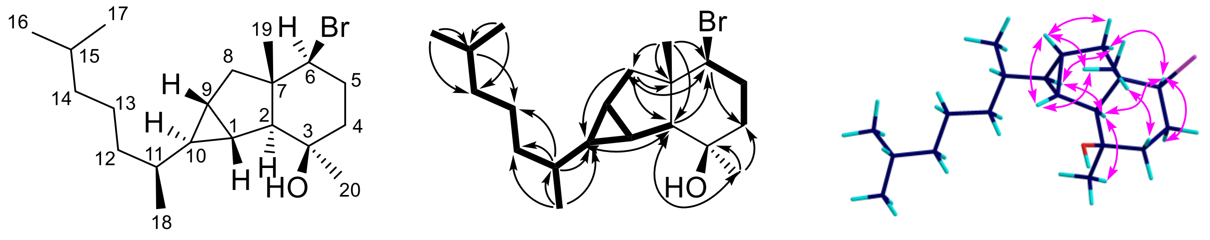

7

Figure S75. Carbon numbering and structure elucidation of **7**. Bold lines represent ^{1}H,^{1}H-COSY correlations, selected HMBC signals are represented by single-headed arrows and selected NOE correlations are depicted by double headed arrows.

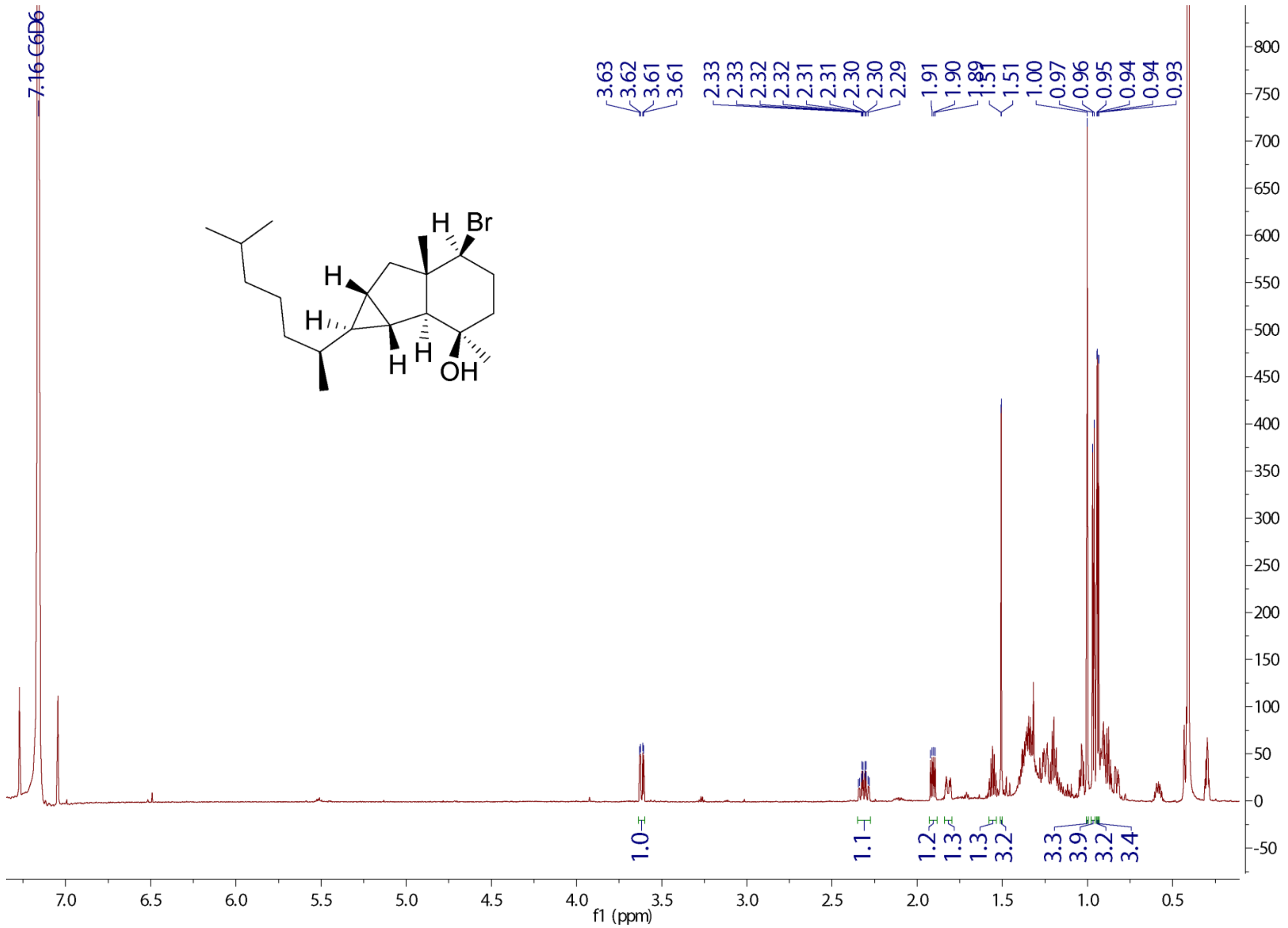

Figure S76. ^{1}H-NMR spectrum of **7** (700 MHz, C_6D_6).

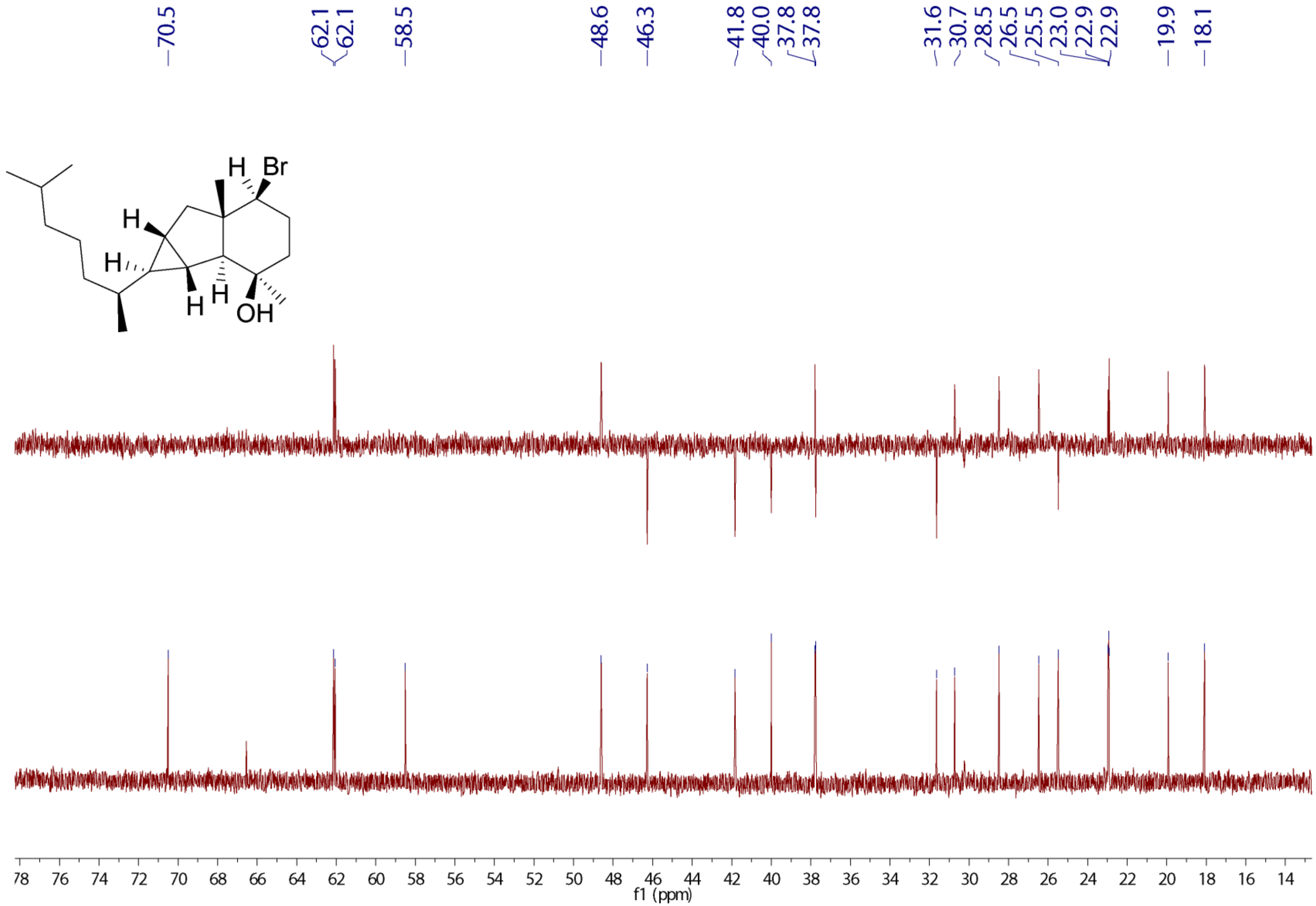

Figure S77. ^{13}C-NMR and ^{13}C-DEPT-135 spectra of **7** (175 MHz, C_6D_6).

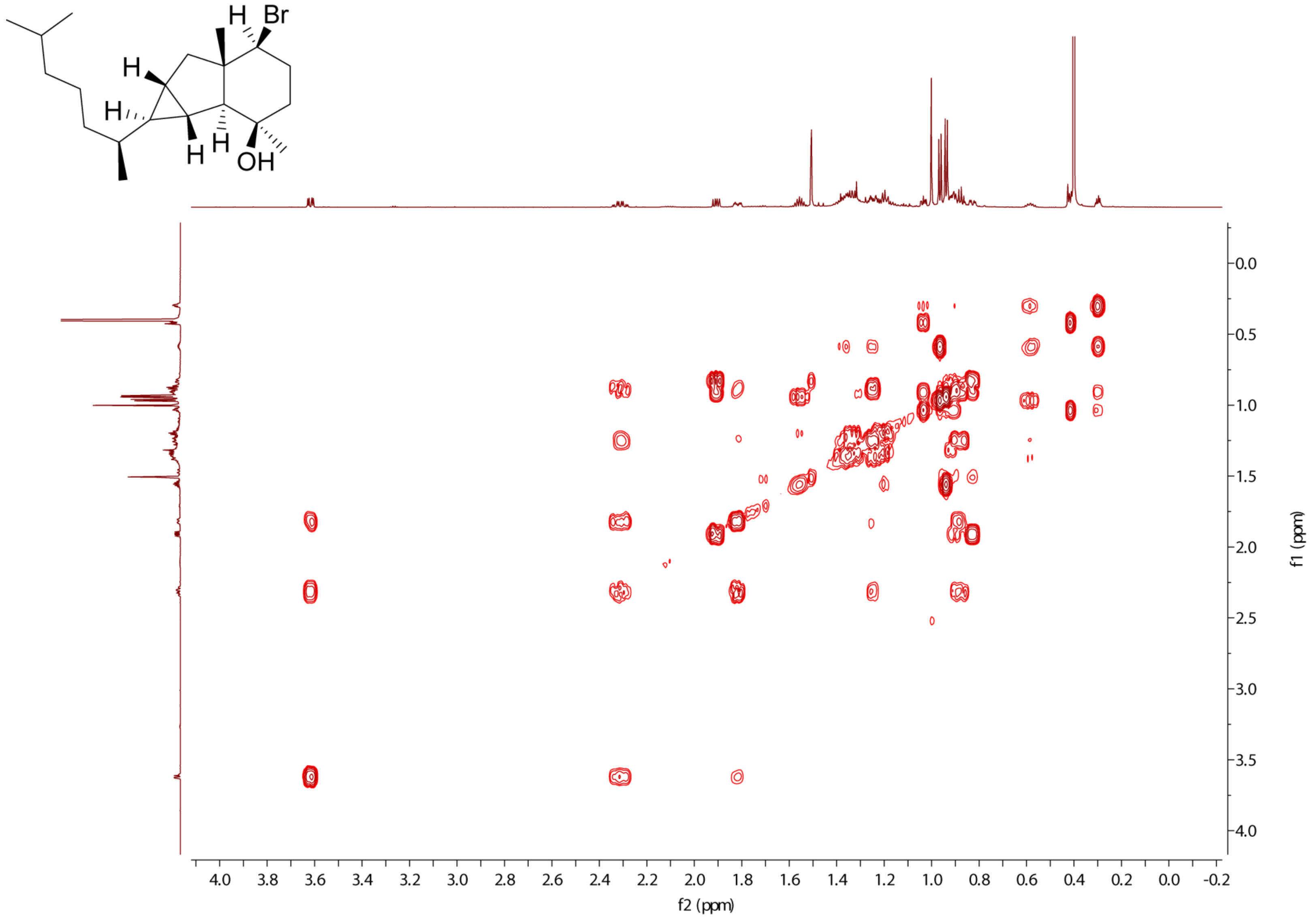

Figure S78. $^{1}H,^{1}H$-COSY spectrum of **7** (C_6D_6).

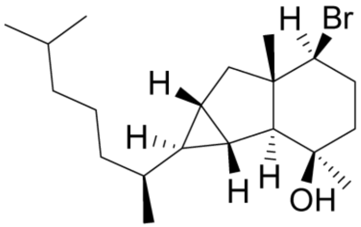

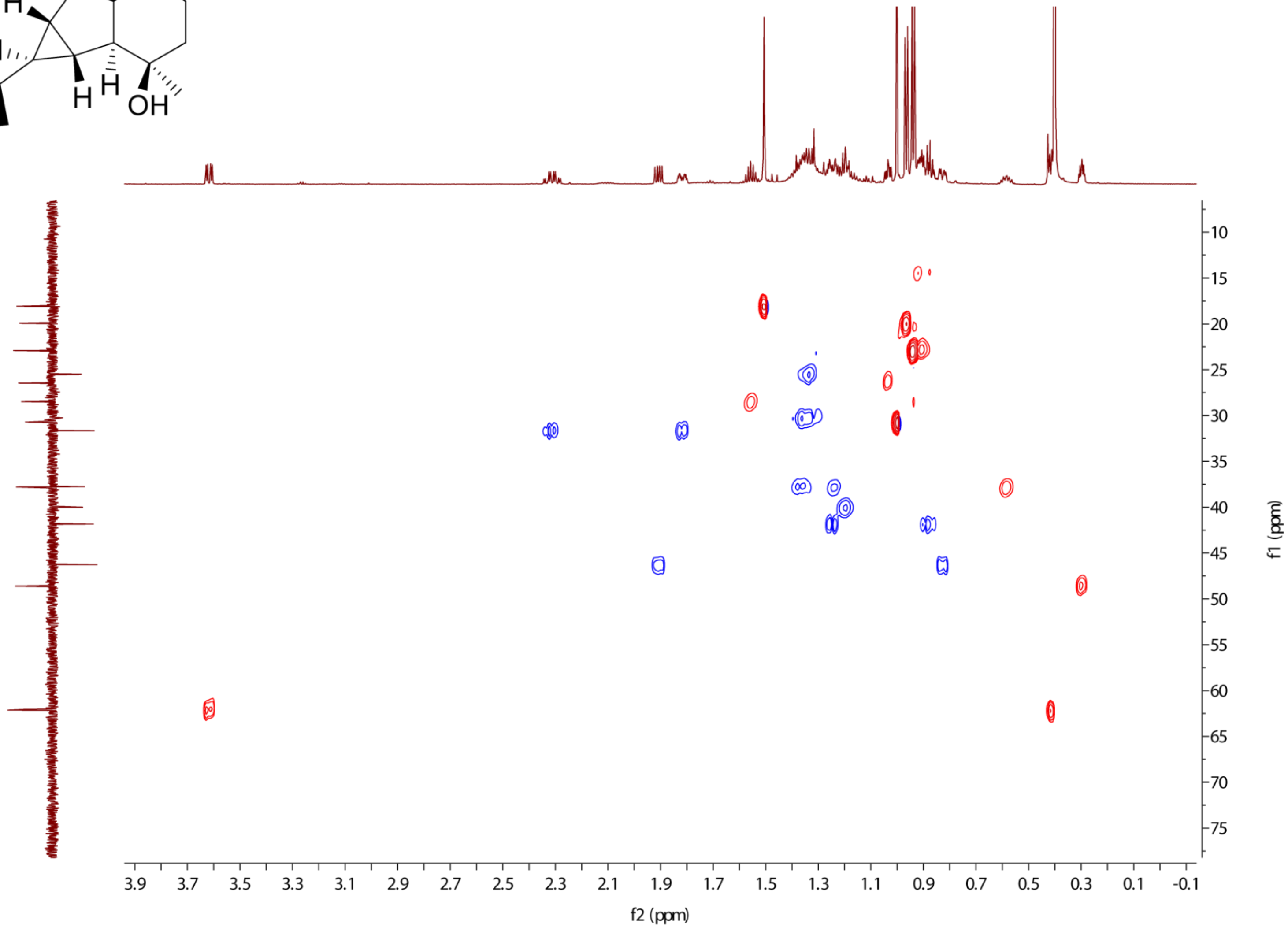

Figure S79. HSQC spectrum of **7** (C_6D_6).

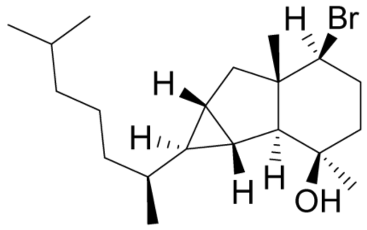

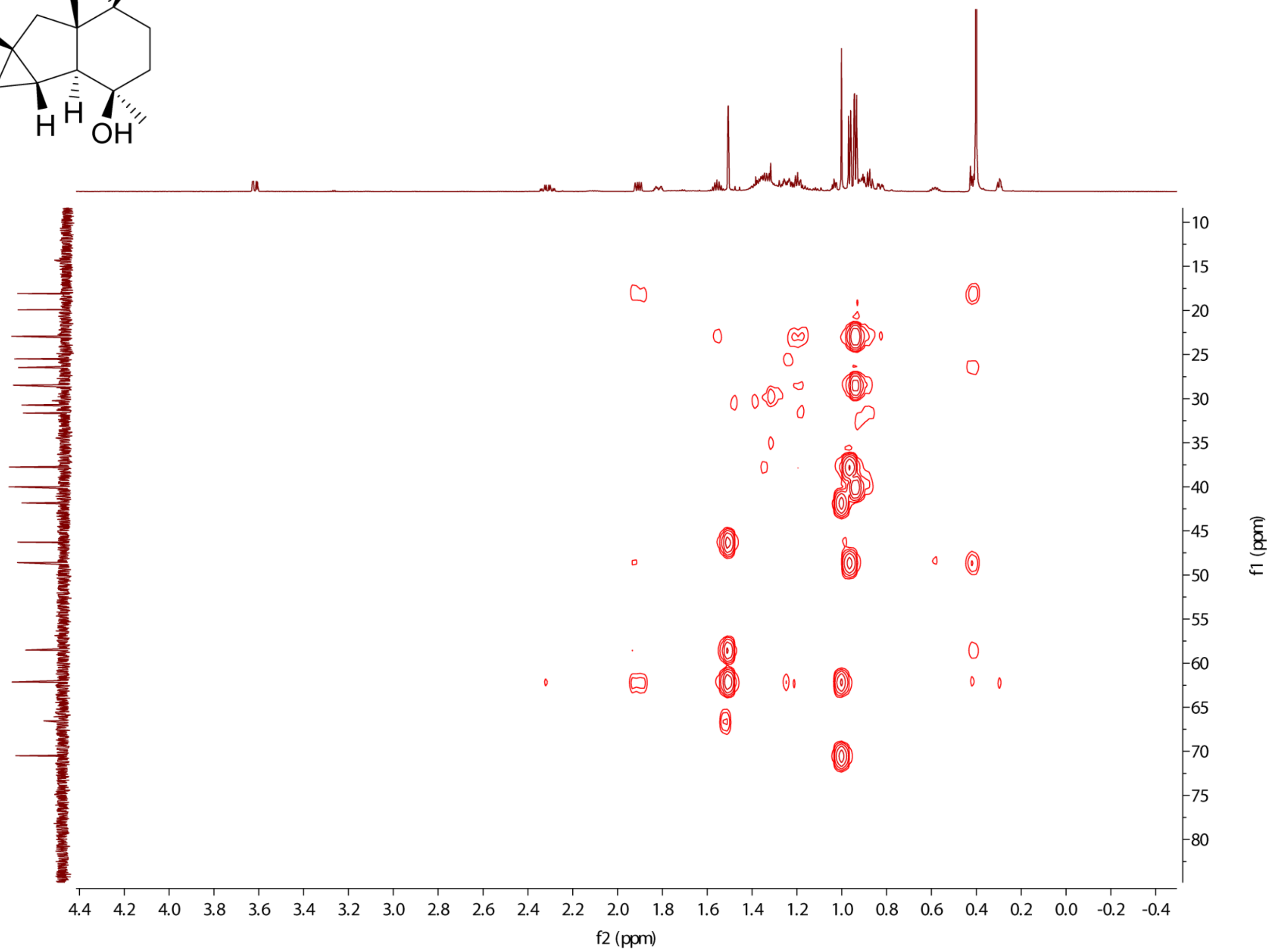

Figure S80. HMBC spectrum of **7** (C_6D_6).

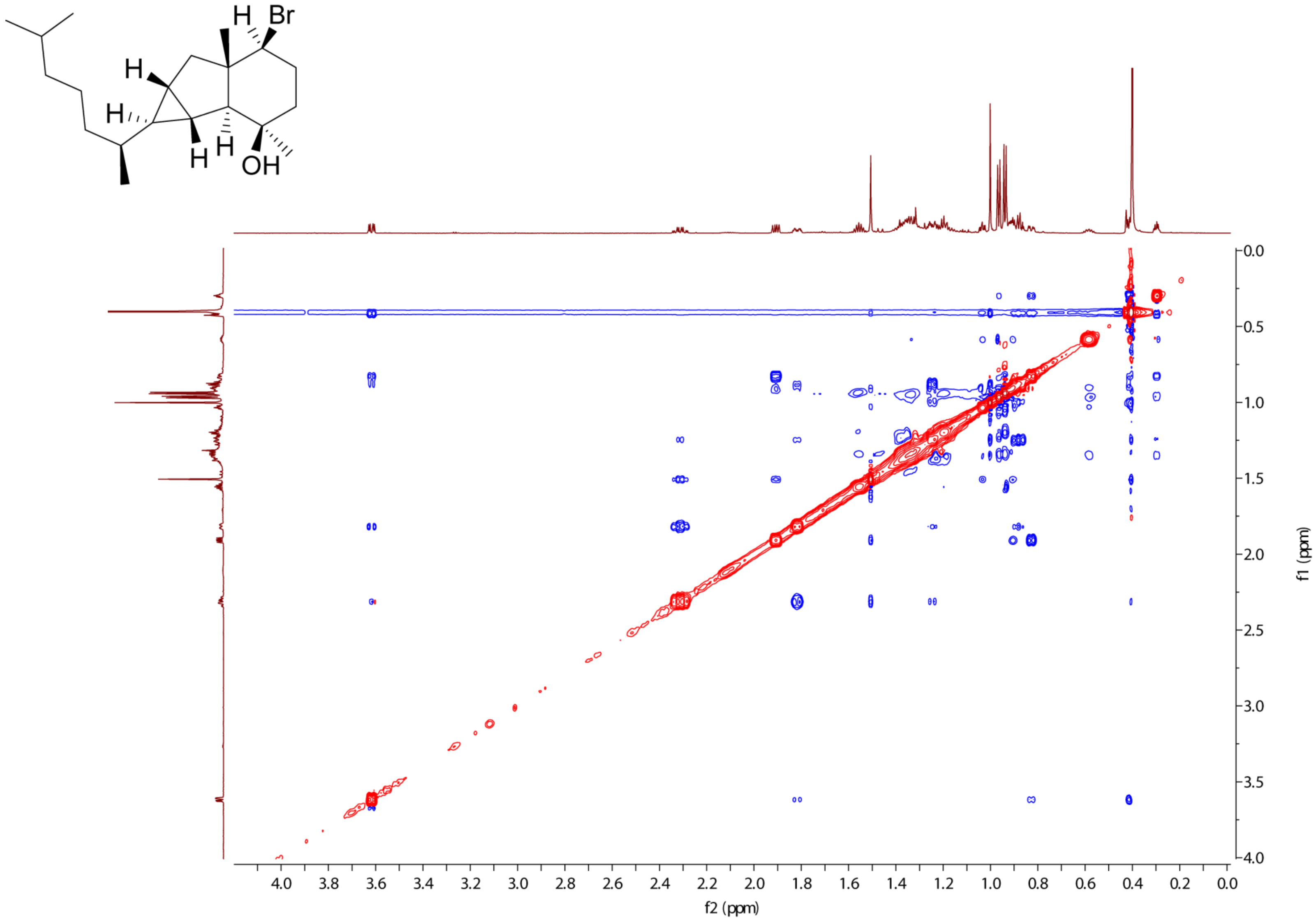

Figure S81. NOESY spectrum of **7** (C_6D_6).

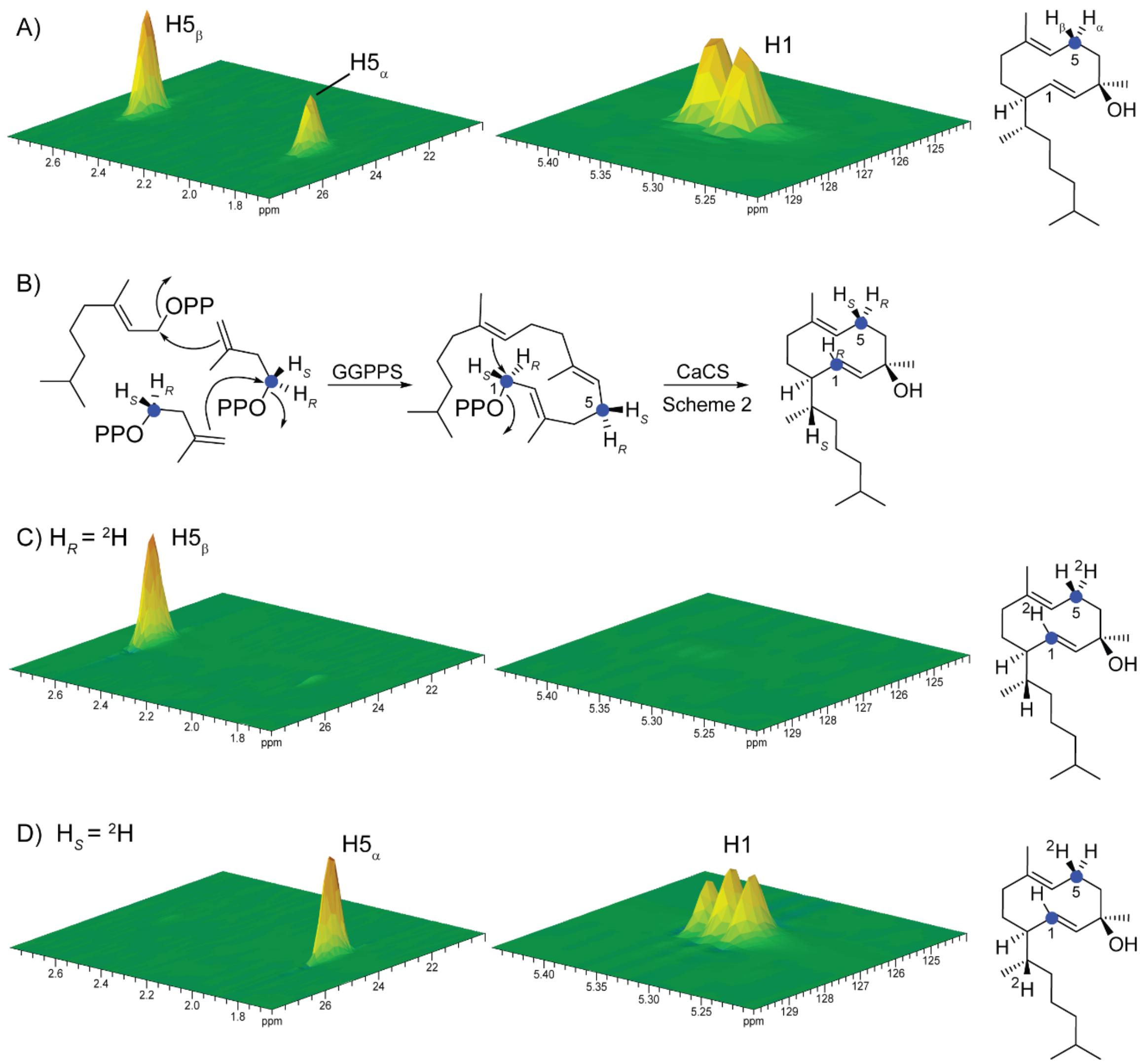

Figure S82. Determination of the absolute configuration of **4** using the substrates (*R*)- and (*S*)-(1-^{13}C,1-^{2}H)IPP and 6,7-dihydro-GPP. A) HSQC spectrum of unlabelled **4**. B) Enzymatic reaction of 6,7-dihydro-GPP with GGPPS and CaCS. C) HSQC spectrum of labelled **4** obtained from (*R*)-(1-^{13}C,1-^{2}H)IPP and 6,7-dihydro-GPP. D) HSQC spectrum of labelled **4** obtained from (*S*)-(1-^{13}C,1-^{2}H)IPP and 6,7-dihydro-GPP. In both labelling experiments the signals for the CH-correlations of the labelled carbons are strongly enhanced, but the signal for one of the diastereotopic hydrogens is vanished because of the substitution with deuterium.

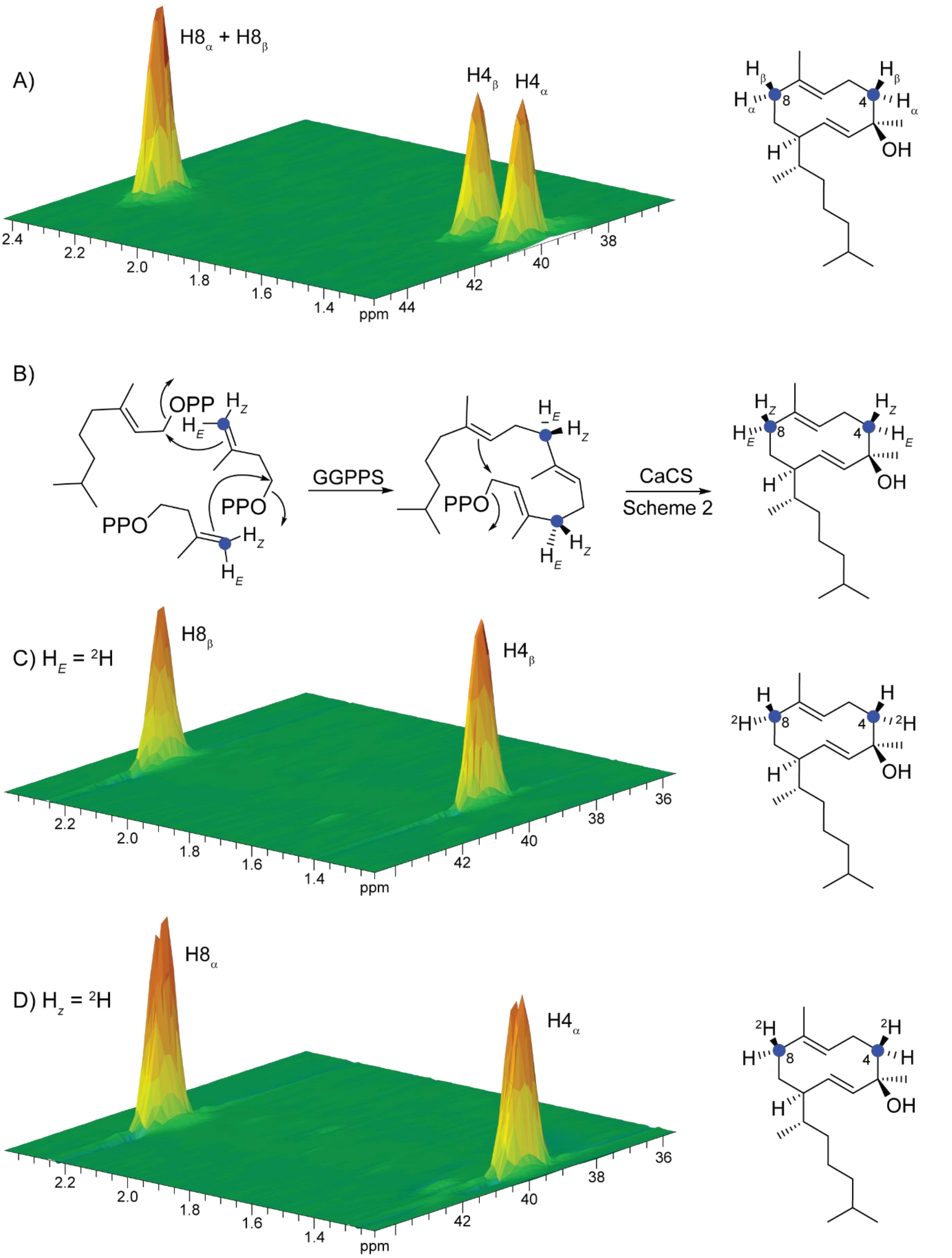

Figure S83. Determination of the absolute configuration of **4** using the substrates (*E*)- and (*Z*)-(4-^{13}C,4-^{2}H)IPP. A) HSQC spectrum of unlabelled **4**. B) Enzymatic reaction of 6,7-dihydro-GPP with GGPPS and CaCS. C) HSQC spectrum of labelled **4** obtained from (*E*)-(4-^{13}C,4-^{2}H)IPP. D) HSQC spectrum of labelled **4** obtained from (*Z*)-(4-^{13}C,4-^{2}H)IPP. In both labelling experiments the signals for the CH-correlations of the labelled carbons are strongly enhanced, but the signal for one of the diastereotopic hydrogens is vanished because of the substitution with deuterium.

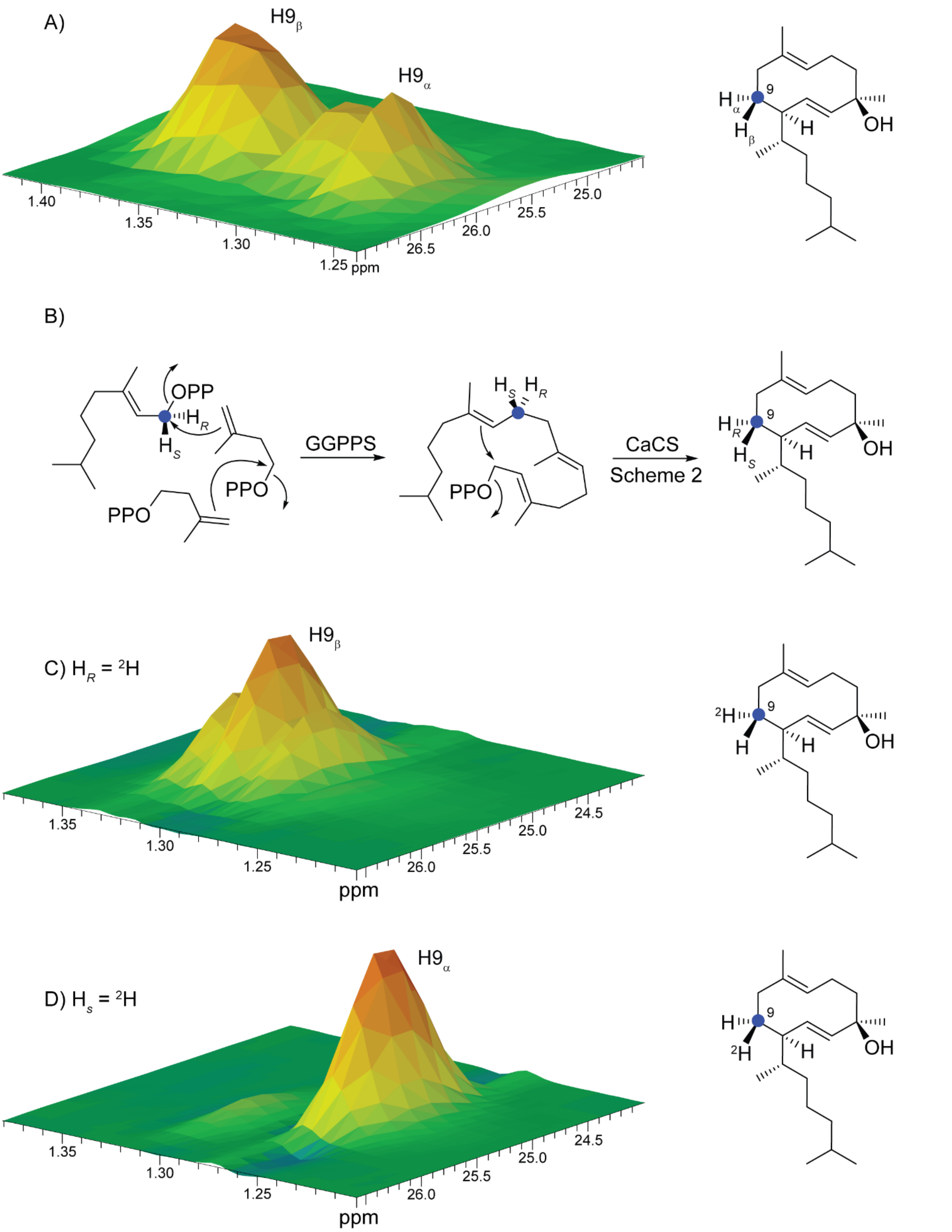

Figure S84. Determination of the absolute configuration of **4** using the substrates (*R*)- and (*S*)-(1-^{13}C,1-^{2}H)-6,7-dihydro-GPP and IPP. A) HSQC spectrum of unlabelled **4**. B) Enzymatic reaction of 6,7-dihydro-GPP with GGPPS and CaCS. C) HSQC spectrum of labelled **4** obtained from (*R*)-(1-^{13}C,1-^{2}H)-6,7-dihydro-GPP and IPP. D) HSQC spectrum of labelled **4** obtained from (*S*)-(1-^{13}C,1-^{2}H)-6,7-dihydro-GPP and IPP. In both labelling experiments the signals for the CH-correlations of the labelled carbons are strongly enhanced, but the signal for one of the diastereotopic hydrogens is vanished because of the substitution with deuterium.

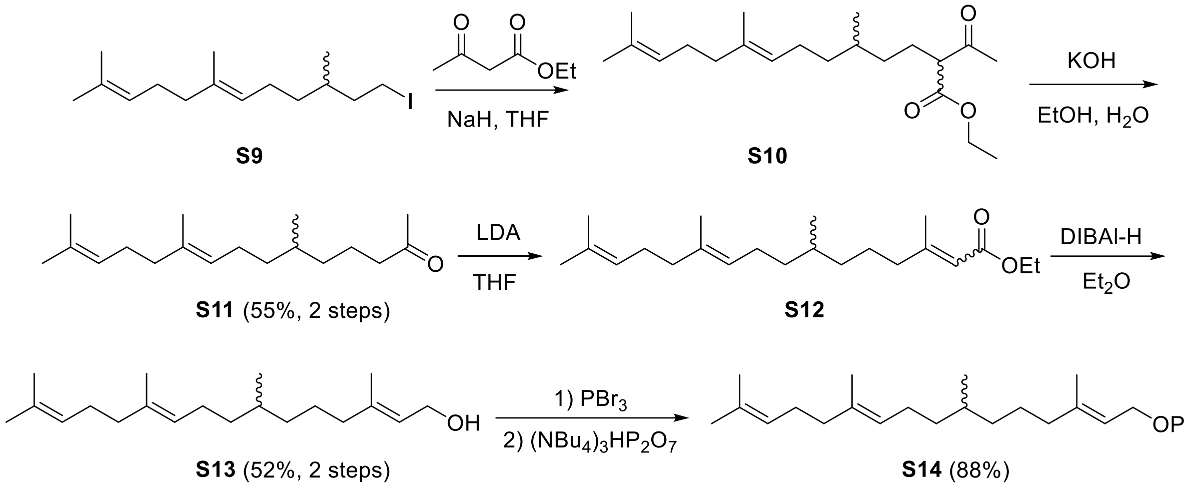

Scheme S3. Synthesis of 6,7-dihydro-GGPP.

Preparation of (*rac*)-(*E*)-6,10,14-trimethylpentadeca-9,13-dien-2-one (S11)

The synthesis of **S11** started from **S9**.[4] Ethyl acetoacetate (244 mg, 1.88 mmol, 2.0 eq.) was dissolved in THF (10 mL) under argon. After cooling to 0 °C, NaH (60%, 75 mg, 1.88 mmol, 2.0 eq.) was added into the reaction in several portions. The reaction mixture was allowed to reach room temperature and stirred for 1 h. Compound **S9** (314 mg, 0.94 mmol, 1.0 eq.) was added dropwise and the mixture was refluxed overnight. After cooling to room temperature, the reaction was quenched by the addition of saturated NH_4Cl solution and extracted with Et_2O for three times. The combined organic phases were dried with $MgSO_4$ followed by removing of the solvent under reduced pressure. **S10** (190 mg) was obtained by repeated column chromatography on silica gel (cyclohexane/ethyl acetate = 10/1) as a mixture of stereoisomers. Then **S10** (1.12 g, 3.3 mmol, 1.0 eq.) was dissolved in EtOH (10 mL) and a solution of KOH (556 mg, 9.9 mmol) in water (3.5 mL) was added. The reaction mixture was refluxed for 3 h, before it was cooled to room temperature and slowly acidified with 2 M HCl solution until CO_2 developed. The suspension was extracted with pentane for three times and the combined organic phases were dried with $MgSO_4$, before concentration under reduced pressure. The residue was applied to column chromatography on silica gel (cyclohexane/ethyl acetate = 10/1) to give the ketone **S11** (800 mg, 3.0 mmol, 55% over 2 steps).

S11. TLC (cyclohexane/ethyl acetate = 10/1): R_f = 0.27. HR-MS (APCI): calc. for $[C_{18}H_{33}O]^+$ *m*/*z* = 265.2526; found *m*/*z* = 265.2521. IR (diamond ATR): ṽ = 2925 (s), 2855 (m), 1719 (s), 1452 (m), 1376 (m), 1360 (m), 1165 (w) cm^{-1}. ^1H-NMR (C_6D_6, 500 MHz): δ = 5.26 (m, 2H, 2xCH), 2.19 (m, 2H, CH_2), 2.05 (m, 4H, 2xCH_2), 1.92 (m, 2H, CH_2), 1.69 (d, $^3J_{H,H}$ = 1.3 Hz, 3H, CH_3), 1.66 (s, 3H, CH_3), 1.63 (s, 3H, CH_3), 1.57 (s, 3H, CH_3), 1.49 (m, 2H, CH_2), 1.38 (m, 2H, CH_2), 1.18 (m, 2H, CH_2), 1.00 (m, 1H, CH), 0.87 (d, $^3J_{H,H}$ = 6.5 Hz, 3H, CH_3) ppm. ^{13}C-NMR (C_6D_6, 125 MHz): δ = 206.2 (C_q), 134.8 (C_q), 131.2 (C_q), 125.4 (CH), 125.0 (CH), 43.7 (CH_2), 40.3 (CH_2), 37.4 (CH_2), 36.8 (CH_2), 32.7 (CH), 29.3 (CH_3), 27.3 (CH_2), 25.9 (CH_2), 25.9 (CH_3), 21.6 (CH_2), 19.7 (CH_3), 17.8 (CH_3), 16.1 (CH_3) ppm. EI-MS (70 eV): *m*/*z* (%) = 264 (1), 249 (1), 221 (21), 203 (8), 177 (2), 163 (2), 151 (2), 137 (5), 123 (39), 109 (25), 95 (34), 81 (32), 69 (100), 55 (20), 43 (77). GC (HP-5MS): *I* = 1910.

Preparation of (2*E*,10*E*)-3,7,11,15-tetramethylhexadeca-2,10,14-trien-1-ol (S13)
The ketone **S11** (370 mg, 1.40 mmol) was elongated by HWE reaction with triethyl phosphonoacetate using the same procedure as for **S2** (page 50 of SI), affording the isomeric mixture of ethyl esters **S12** (253 mg). The mixture was reduced with DIBAl-H as described for **S3** above (page 51 of SI). Repeated column chromatography (cyclohexane/ethyl acetate = 7/3) yielded **S13** (212 mg, 0.73 mmol, 52% over 2 steps).
S13. TLC (cyclohexane/ethyl acetate = 1/1): R_f = 0.67. HR-MS (APCI): calc. for $[C_{20}H_{37}O]^+$ *m*/*z* = 293.2839; found *m*/*z* = 293.2838. IR (diamond ATR): $\tilde{\nu}$ = 3321 (w), 2963 (s), 2928 (s), 2859 (m), 1261 (s), 1090(s), 1017 (s), 798 (s) cm^{-1}. ^{1}H-NMR and ^{13}C-NMR data were identical to those reported in the literature.[31] EI-MS (70 eV): *m*/*z* (%) = 292 (1), 274 (3), 259 (0.4), 249 (1), 231 (2), 203 (1), 189 (1), 175 (2), 163 (2), 149 (6), 136 (7), 123 (30), 109 (16), 95 (24), 81 (38), 69 (100), 55 (25). GC (HP-5MS): *I* = 2177.

Preparation of 6,7-dihydro-GGPP trisammonium salt (S14)
S13 (150 mg, 0.51 mmol) was converted into its pyrophosphate with $(NBu_4)_3HP_2O_7$ using the same procedure as for **S4a**–**S4c** mentioned above to yield 6,7-dihydro-GGPP (**S14**).
S14. Yield: 225 mg (0.45 mmol, 88%). HR-MS (ESI–): $[M–H]^-$ calc. for $[C_{20}H_{37}O_7P_2]^-$ *m*/*z* = 451.2020; found *m*/*z* = 451.2019. ^{1}H-NMR (D_2O, 700 MHz) δ = 5.42 (brs, 1H, CH), 5.09 (dt, *J* = 22.7, 6.9 Hz, 2H, CH_2), 4.44 (brs, 2H, 2x CH), 1.99 (m, 8H, 4x CH_2), 1.70 (s, 3H, CH_3), 1.63 (s, 3H, CH_3), 1.57 (s, 3H, CH_3), 1.55 (s, 3H, CH_3), 1.36 (m, 5H, 2x CH_2, 1x CH), 1.12 (m, 2H, CH_2), 0.88 (m, 3H, CH_3) ppm. ^{13}C-NMR (D_2O, 175 MHz) δ = 142.3 (C_q), 134.2 (C_q), 131.9 (C_q), 125.0 (CH), 124.9 (CH), 124.5 (CH), 62.3 (CH_2), 39.9 (CH_2), 39.7 (CH), 37.3 (CH_2), 36.9 (CH_2), 32.4 (CH_2), 26.6 (CH_2), 25.5 (CH_2), 25.4 (CH_3), 25.3 (CH_2), 19.2 (CH_3), 17.3 (CH_3), 15.8 (CH_3), 15.7 (CH_3) ppm. ^{31}P-NMR (D_2O, 162 MHz) δ = –6.26 (d, *J* = 21.9 Hz, 1P), –9.98 (d, *J* = 20.3, 1P) ppm.

Incubation experiments with 6,7-dihydro-GGPP and recombinant CaCS
A preparative scale incubation was performed by dissolving 6,7-dihydro-GGPP (220 mg, 0.47 mmol) in substrate buffer (22 mL). The reaction mixture was split into 220 equal volumes and to each sample (100 µL) a protein preparation of recombinant CaCS (66 µL, 50 µg mL^{-1}) and incubation buffer (4 mL) was added. The reaction mixtures were incubated overnight at 28 °C and pooled. The products were extracted with pentane (200 mL), the extract was concentrated in vacuo and the residue (<1 mg) was analysed by GC/MS. Purification of the main product was tried, but the amount of pure compound obtained was too low for structure elucidation by NMR.

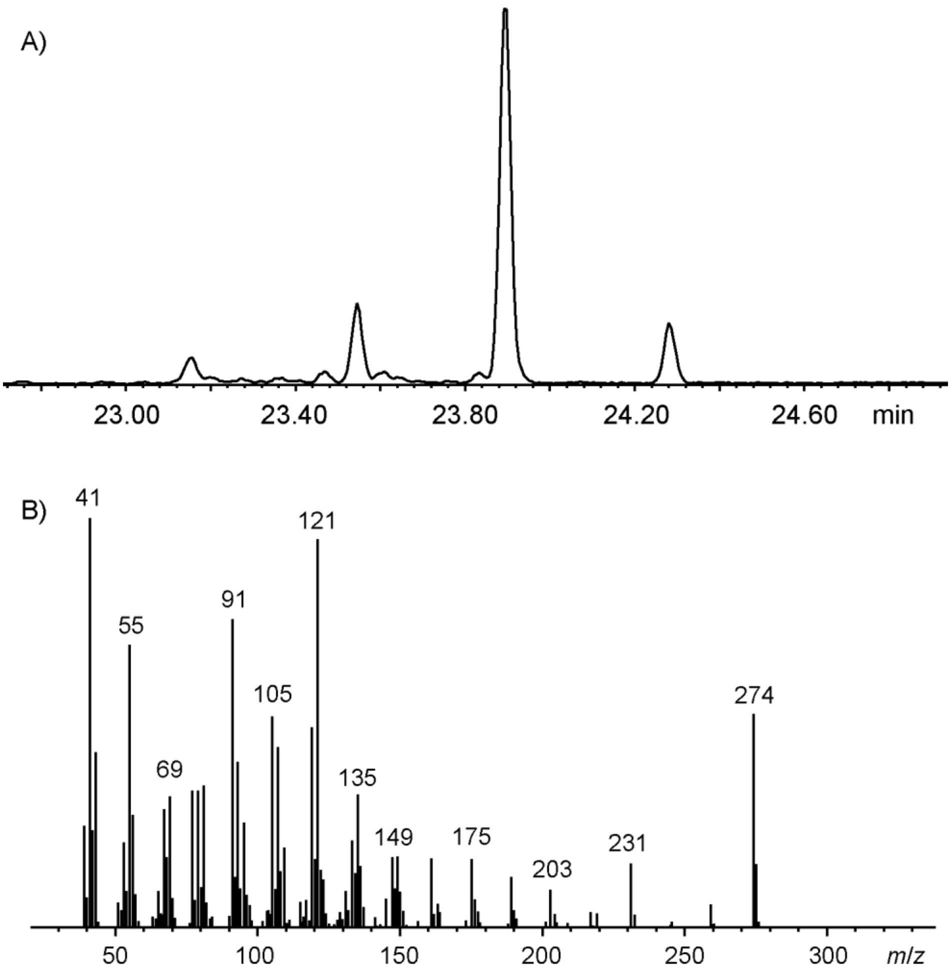

Figure S85. A) Total ion chromatogram of the products obtained from 6,7-dihydro-GGPP with CaCS. B) Mass spectrum for the major compound.

References

[1] K. Grob, F. Zürcher, *J. Chromatogr.* **1976**, 117, 285.
[2] C. M. Starks, K. Back, J. Chappell, J. P. Noel, *Science* **1997**, *277*, 1815.
[3] E. Y. Shishova, L. Di Costanzo, D. E. Cane, D. W. Christianson, *Biochemistry* **2007**, *46*, 1941.
[4] P. Baer, P. Rabe, K. Fischer, C. A. Citron, T. A. Klapschinski, M. Groll, J. S. Dickschat, *Angew. Chem. Int. Ed.* **2014**, *53*, 7652.
[5] J. S. Dickschat, *Nat. Prod. Rep.* **2016**, *33*, 87.
[6] J. Rinkel, J. S. Dickschat, *Org. Lett.* **2019**, *21*, 2426.
[7] S.-Y. Kim, P. Zhao, M. Igarashi, R. Sawa, T. Tomita, M. Nishiyama, T. Kuzuyama, *Chem. Biol.* **2009**, *16*, 736.
[8] C. Nakano, T. Tezuka, S. Horinouchi, Y. Ohnishi, *J. Antibiot.* **2012**, *65*, 551.
[9] P. Rabe, J. S. Dickschat, *Angew. Chem. Int. Ed.* **2013**, *52*, 1810.
[10] W. K. W. Chou, I. Fanizza, T. Uchiyama, M. Komatsu, H. Ikeda, D. E. Cane, *J. Am. Chem. Soc.* **2010**, *132*, 8850.
[11] D. E. Cane, J. K. Sohng, C. R. Lamberson, S. M. Rudnicki, Z. Wu, M. D. Lloyd, J. S. Oliver, B. R. Hubbard, *Biochemistry* **1994**, *33*, 5846.
[12] P. Rabe, M. Samborskyy, P. F. Leadlay, J. S. Dickschat, *Org. Biomol. Chem.* **2017**, *15*, 2353.
[13] P. Rabe, J. Rinkel, T. A. Klapschinski, L. Barra, J. S. Dickschat, *Org. Biomol. Chem.* **2016**, *14*, 158.
[14] P. Rabe, J. Rinkel, E. Dolja, T. Schmitz, B. Nubbemeyer, T. H. Luu, J. S. Dickschat, *Angew. Chem. Int. Ed.* **2017**, *56*, 2776.
[15] C. Nakano, S. Horinouchi, Y. Ohnishi, *J. Biol. Chem.* **2011**, *286*, 27980.
[16] J. Rinkel, J. S. Dickschat, *Org. Lett*. **2019**, *21*, 9442.
[17] X. Lin, R. Hopson, D. E. Cane, *J. Am. Chem. Soc*. **2006**, *128*, 6022.
[18] B. Neumann, A. Pospiech, H. U. Schairer, *Trends Genet.* **1992**, *8*, 332.
[19] G. R. Fulmer, A. J. M. Miller, N. H. Sherden, H. E. Gottlieb, A. Nudelman, B. M. Stoltz, J. E. Bercaw, K. I. Goldberg, *Organometallics* **2010**, *29*, 2176.
[20] P. Rabe, L. Barra, J. Rinkel, R. Riclea, C. A. Citron, T. A. Klapschinski, A. Janusko, J. S. Dickschat, *Angew. Chem. Int. Ed.* **2015**, *54*, 13448.
[21] G. Bian, J. Rinkel, Z. Wang, L. Lauterbach, A. Hou, Y. Yuan, Z. Deng, T. Liu, J. S. Dickschat, *Angew. Chem. Int. Ed.* **2018**, *57*, 15887.
[22] T. Mitsuhashi, J. Rinkel, M. Okada, I. Abe, J. S. Dickschat, *Chem. Eur. J.* **2017**, *23*, 10053.
[23] J. Rinkel, L. Lauterbach, J. S. Dickschat, *Angew. Chem. Int. Ed.* **2019**, *58*, 452.
[24] J. Rinkel, L. Lauterbach, P. Rabe, J. S. Dickschat, *Angew. Chem. Int. Ed.* **2018**, *57*, 3238.
[25] J. Rinkel, P. Rabe, X. Chen, T. G. Köllner, F. Chen, J. S. Dickschat, *Chem. Eur. J.* **2017**, *23*, 10501.
[26] P. Rabe, J. Rinkel, B. Nubbemeyer, T. G. Köllner, F. Chen, J. S. Dickschat, *Angew. Chem. Int. Ed.* **2016**, *55*, 15420.
[27] T. A. Klapschinski, P. Rabe, J. S. Dickschat, *Angew. Chem. Int. Ed.* **2016**, *55*, 10141.
[28] L. Lauterbach, J. Rinkel, J. S. Dickschat, *Angew. Chem. Int. Ed.* **2018**, *57*, 8280.
[29] J. Rinkel, L. Lauterbach, J. S. Dickschat, *Angew. Chem. Int. Ed.* **2017**, *56*, 16385.
[30] A. Hou, J. S. Dickschat, *Angew. Chem. Int. Ed.* **2020**, doi:10.1002/anie.202010084.
[31] Y. Suhara, A. Wada, Y. Tachibana, M. Watanabe, K. Nakamura, K. Nakagawa, T. Okano, *Bioorg. Med. Chem.* **2010**, *18*, 3116.
